# Microgravity Induces Changes in Microsome-Associated Proteins of Arabidopsis Seedlings Grown on Board the International Space Station

**DOI:** 10.1371/journal.pone.0091814

**Published:** 2014-03-11

**Authors:** Christian Mazars, Christian Brière, Sabine Grat, Carole Pichereaux, Michel Rossignol, Veronica Pereda-Loth, Brigitte Eche, Elodie Boucheron-Dubuisson, Isabel Le Disquet, Francisco Javier Medina, Annick Graziana, Eugénie Carnero-Diaz

**Affiliations:** 1 Laboratoire de Recherches en Sciences Végétales, Université de Toulouse UPS, CNRS UMR5546, Castanet-Tolosan, France; 2 Institut de Pharmacologie et de Biologie Structurale IPBS CNRS, Fédération de Recherche 3450 Agrobiosciences Interactions et Biodiversités Plateforme Protéomique Génopole Toulouse Midi Pyrénées, Toulouse, France; 3 GSBMS, Université de Toulouse, Toulouse, France; 4 UR5-PCMP-EAC 7180 CNRS, Université Pierre et Marie Curie-Sorbonne Universités, Paris, France; 5 Centro de Investigaciones Biologicas CSIC, Madrid, Spain; University of Firenze, Italy

## Abstract

The “GENARA A” experiment was designed to monitor global changes in the proteome of membranes of *Arabidopsis thaliana* seedlings subjected to microgravity on board the International Space Station (ISS). For this purpose, 12-day-old seedlings were grown either in space, in the European Modular Cultivation System (EMCS) under microgravity or on a 1 g centrifuge, or on the ground. Proteins associated to membranes were selectively extracted from microsomes and identified and quantified through LC-MS-MS using a label-free method. Among the 1484 proteins identified and quantified in the 3 conditions mentioned above, 80 membrane-associated proteins were significantly more abundant in seedlings grown under microgravity in space than under 1 g (space and ground) and 69 were less abundant. Clustering of these proteins according to their predicted function indicates that proteins associated to auxin metabolism and trafficking were depleted in the microsomal fraction in µg space conditions, whereas proteins associated to stress responses, defence and metabolism were more abundant in µg than in 1 g indicating that microgravity is perceived by plants as a stressful environment. These results clearly indicate that a global membrane proteomics approach gives a snapshot of the cell status and its signaling activity in response to microgravity and highlight the major processes affected.

## Introduction

Earth’s gravity is a permanent stimulus that influences living organisms. Among the eukaryotes, plants probably best display the effects induced by this permanent constraint, especially if we consider their diversity of shape. Thus, roots are forced to sink in the soil to extract minerals and water, while shoots grow upwards experiencing a negative gravitropism to optimally access the light necessary for carbon dioxide assimilation. This permanent stimulus imposes on land plants a mechanical load which is one thousand times stronger than that experienced by plants living in water [1]. To withstand these constraints, plants have evolved, strengthening their shoots mainly by stiffening the cell walls with a crosslinked network of lignins, cellulose and hemicelluloses. Together, these responses imply the coordinated activity of the enzymes involved in the synthesis of the building bricks of cell wall and of the enzymes involved in bridging and crosslinking these building units [2]–[7]. Obviously therefore, gravity plays a crucial role on the development and shape of plants on earth and to understand the mechanisms involved, morphological and molecular changes induced by this permanent stimulus have been studied for years on the ground [8]–[11]. This research area is currently experiencing a renewed interest in the context of future long-term space missions where plants are envisioned as food and fiber supply, ambient air purifiers, human waste and water recyclers, and also as factors contributing to the well being of the crew by attenuating the possible side effects of long-term missions such as depression. The reasons for studying plant biology in space as well as the main lessons drawn from the last space missions including plant payloads have been recently reviewed [12]. However the opportunities of space missions are scarce and they need detailed and robust preparation on ground. For this purpose, scientists have set up various devices allowing them to modulate the gravity stimulus either by increasing it, generally using centrifuges that mimic hypergravity, or by artificially changing the orientation of the plant within the gravity field to mimic the conditions encountered in space. Such conditions can be generally achieved using a 2-D-clinostat, a random positioning machine (RPM, 3D clinostat) or magnetic levitation [13]. From these seminal studies a quantity of important information has been extracted and conceptual models have been proposed to understand how the physical stimulus generated by gravity is perceived in roots and shoots. One widely accepted model is based on the involvement of specialized elements, the starch-statoliths: it has been shown that the perception of gravity in Arabidopsis roots occurs in specialized cells located in the columella of the cap in the root tip or within the endodermis of shoots [14]. These cells, called statocytes [15], [16], contain dense amyloplasts (statoliths) that sediment upon gravistimulation, initiating the generation of an auxin gradient responsible for the graviresponse, i.e downwards curvature of roots and upward curvature of shoots [17]. Both differentiated and non-differentiated cells are able to perceive this gradient and react to changes in the acceleration stimulus. This perception leads to the differential distribution of auxin that will induce asymmetric cell elongation [18]. But cells devoid of statoliths or Arabidopsis mutants deficient in starch synthesis [19] are still responsive to gravity but less so [20]. This peculiarity forced new theories to emerge which have been recently reviewed in [21]. An important issue in graviperception concerns the concept of presentation time, i.e. the minimal duration of stimulation in the gravitational field required to induce a gravitropic response such as amyloplast sedimentation in plants. This duration is estimated to be very short (less than 10 s) [22], [23], but it is long enough to cause the redistribution of auxin that will induce gene reprogramming leading to an adaptive plant response.

Numerous studies have focused on gene expression either on the ground using plant material challenged with the devices mentioned above or during space missions [12], [24]–[29]. Although they can be informative, these approaches highlight neither the behavior of the encoded proteins (subcellular location, increased or decreased abundance) nor the activities and functions that they perform (e.g. modulation of specific targets, enzyme activity). These functions are of paramount importance in ensuring the adaptive response. Some of these molecules can be determined using proteomics approaches but only few studies have been performed to investigate the response to gravity changes [30]–[34].

In the context of a space experiment called GENARA A standing for Gravity regulated genes in *Arabidopsis thaliana*(ESA-RA-LS-01-ILSRA-2001-001) and performed in 2010 on board the International Space Station (ISS) we took advantage of the access to microgravity conditions to evaluate the global qualitative and quantitative changes of the membrane –associated proteome produced in microgravity. It is well known that membranes and more specifically the plasma membrane, which contain receptors, are the starting point for signaling pathways initiated by the perception of environmental cues. Membranes constitute an interface between the extracellular environment and the cell but also an exchange platform allowing communication between compartments and organelles within the cell. A better knowledge of protein trafficking events occurring through membranes will help to better understand the way plants respond to microgravity, taking into account that to generate or maintain the auxin gradients necessary for the graviresponse, protein trafficking of auxin efflux transporters is critical [35]. The auxin transporters such as PIN auxin efflux facilitators cycle between the plasma membrane and endosomal compartments. Their polar distribution requires the endocytic pathways [36], [37]. Several lines of evidence point to a role of phosphatidylinositol signaling in these pathways that involve membrane-associated enzymes such as PLC or PIP5K to generate IP3 or PIP2 respectively [38]. Similarly, shoot gravitropism (sgr) mutants encoding proteins involved in the machinery of the vesicle trafficking pathway highlight the numerous protein exchanges that can occur between the plasmalemma and the endomembranes [39]–[42]. In addition, the recent demonstration that signaling complexes are associated with rafts in signaling pathways transducing external cues such as the elicitin cryptogein [43] highlights the importance of membranes in hosting signaling complexes. All together, such a crucial role of membranes in signaling pathways and more specifically in graviresponses pushed us to set up a quantitative proteomic approach to measure the relative abundance of membrane-associated proteins in Arabidopsis seedlings grown either under microgravity in space or under 1 g in space or on the ground. We present here the results obtained from this large-scale proteomics study which reveals clusters of proteins that have been identified as being up or down represented in the microsomal fractions and we discuss these data in the context of the current knowledge of gravity signaling.

## Materials and Methods

### Plant Materials and Growth Conditions

*Arabidopsis thaliana* (Col O ecotype) seeds were surface sterilized by soaking them in a sterilizing solution mixture containing 0.4% available chlorine made up of 1 vol of diluted commercial bleach (250 ml of 9.6% available chlorine (36° chlorometric degrees) in 750 ml of distilled water), 5 vol of 100% ethanol and 10 drops of Triton X100.

A volume of about 10–50 µl of Arabidopsis seeds were soaked in 2 ml of the sterilization solution in a 2 ml Eppendorf tube with occasional agitation for 10 min. The seeds were then quickly rinsed with two washes of 95% ethanol, and remaining ethanol of the last wash was carefully removed with a pipette. The seeds were then dispersed on the surface of the tube and left to dry for 2 hours under a laminar flow hood. The sterile seeds were sown as indicated below.

### GENARA A Hardware

**Figure 1:**
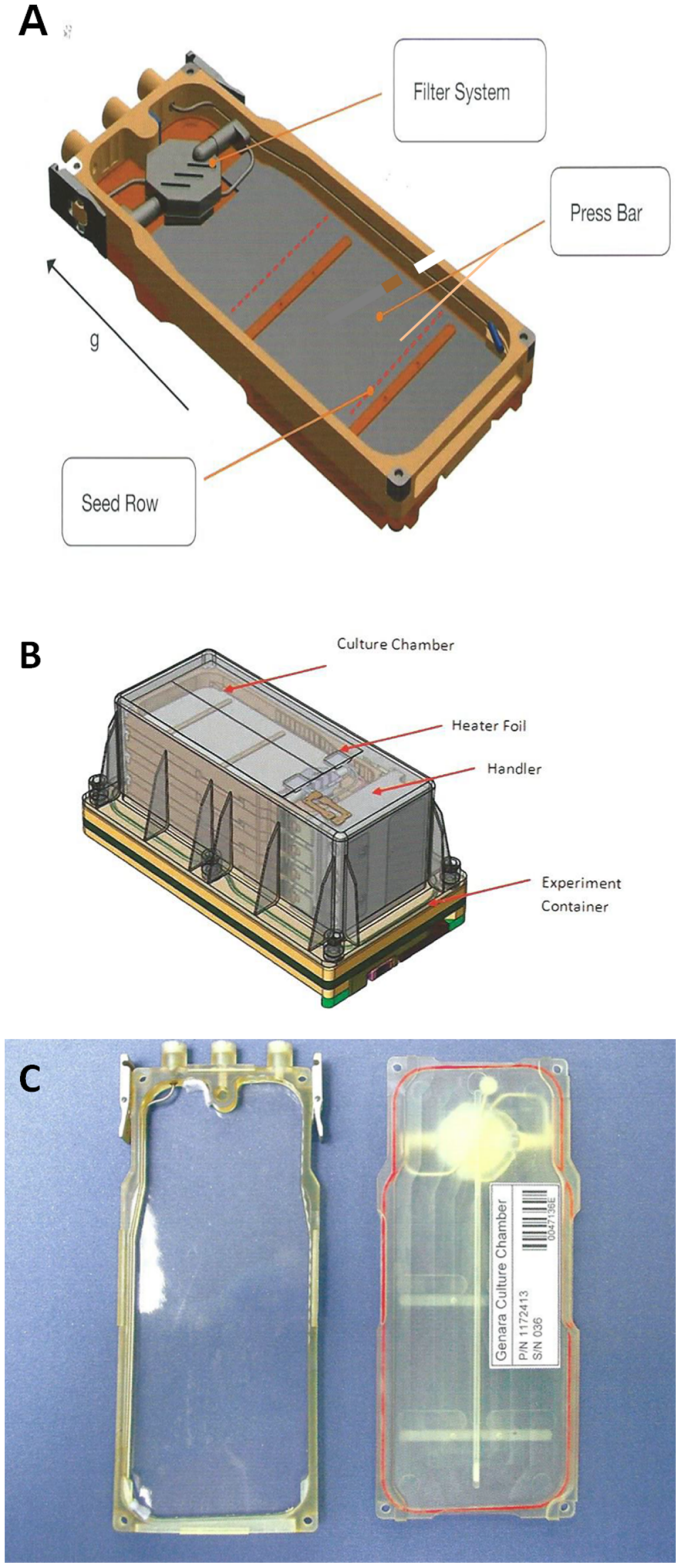
Design and views of the culture chamber and of the experiment container used during the GENARA-A experiment. (A, C) View of a culture chamber, B. View of an experiment container housing four culture chambers. The grey part on the scheme corresponds to the filter pad with a nylon mesh sewed on top (A). Empty culture chamber (C-left) and closed culture chamber with the Biofoil (C-right). (Adapted from the Space Biology Product Catalog of ASTRIUM company).

Specific polysulfone culture chambers (CC) were designed and built by ASTRIUM company according to our specifications (Fig. 1a–c). The CC contained cellulose paper (Whatman 17 CHR) soaked in (5.6 ml) of half-strength Murashige and Skoog medium covered with a nylon Nitex membrane (Sefar AG,Heiden Switzerland) of 37 µm mesh size sewn on top of it. This seed support was fixed to the cassette with two press bars (Fig. 1a). After drying under a laminar flow hood, the support was mounted inside the CC equipped with a sterilizing 0.22 µm filter to avoid any contamination during automatic hydration (Fig. 1a). After sterilization of the cassette, 200 sterilized seeds of Columbia ecotype (Col0) were layered in humid conditions on two rows. The seeds were then dried out overnight under a laminar flow hood and the cassettes were sealed with a Biofoil membrane allowing air exchange (Fig. 1c). The prepared cassettes were then stored in the dark at 4°C until launching.

### Experimental Procedures during the Spaceflight

The GENARA A experiment (ESA-RA-LS-01-ILSRA-2001-001) was launched on space shuttle mission STS -132 using an Atlantis shuttle on May 14^th^ 2010 and was back on earth with a Discovery Shuttle on space mission STS-133 on March 9^th^ 2011. The experiment was performed on the ISS from July 9^th^ until July 22^nd^ 2010. Upon arrival at ISS the samples were stored in the Columbus module (COL1A2) at ambient temperature until experiment starts.

**Figure 2:**
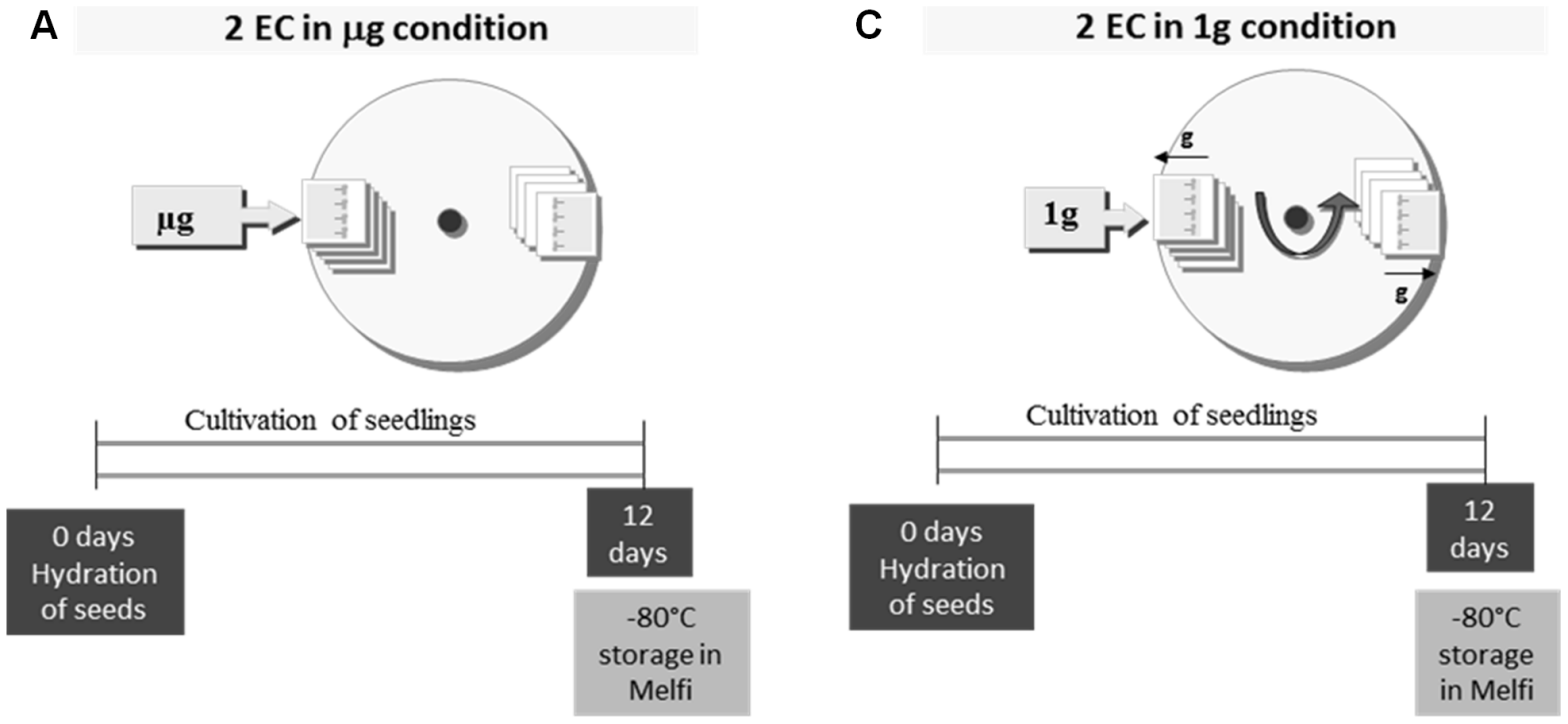
Schematic view of the experiment scenario of Arabidopsis culture on board the ISS.

Each experimental container (EC) held four cassettes (Fig. 1b). The upper cassette of each EC contained a black nylon mesh for image capture. Two ECs were placed on the centrifuge to mimic 1 g conditions whereas two other ECs were not centrifuged in order to be directly exposed to microgravity conditions within the European Modular Cultivation System (EMCS) (Fig. S1) [44], [45]. All the cassettes were hydrated by the EMCS before the beginning of the experiment. Hydration of the upper cassette of each EC was followed by taking images of seedlings once a day. The illumination cycle (16 h light/8 h dark) was provided within the EMCS by an array of 75 W m^−2^ photosynthetically active radiation (PAR) lights in the center of the EC. The space experiment followed the conditions shown in the scheme depicted in Fig. 2. At the end of the experiment each EC was manually placed by an astronaut in the MELFI 2 freezer at -80°C on board the ISS. During the return journey of the shuttle on earth, the samples were packed in a common ziplock bag and stored at −80°C on shuttle’s Glacier freezer. Upon arrival at Kennedy Space Center the bags containing the samples were packed in dedicated dry-ice containers with temperature loggers at a temperature oscillating between −80°C and −75°C. Four weeks after the end of the space experiment, a Ground Reference Run (GRR) was performed from August 19^th^ until September 1^st^ 2010 at N-USOC in Trondheim (Norway) using the same batch of seeds. ECs were placed in a second EMCS apparatus to provide a 1 g ground control. The conditions were those defined by the initial procedure and not by the actual flight conditions. The main change concerned manual hydration instead of EMCS hydration.

### Harvesting the Seedlings

Upon reception of the cassettes and storage at -80°C, harvesting was performed in a cold room placing the cassettes on a plastic support to allow a flow of cold air around the CCs, and a gentle thawing (Fig. S2A). The Biofoil sheet was cut off with a razor blade (Fig. S2B). Cassettes were carefully inspected (e.g. growth of seedlings, water status) and after thawing seedlings were carefully harvested (Fig. S2C). To minimize and average putative CC side effects, plants from three different CCs were collected and mixed together and immediately ground up in liquid nitrogen with a pestle and mortar (Fig. S2D). The powder obtained was then freeze dried and stored at –80°C until protein extraction.

### Microsome Preparation

**Figure 3:**
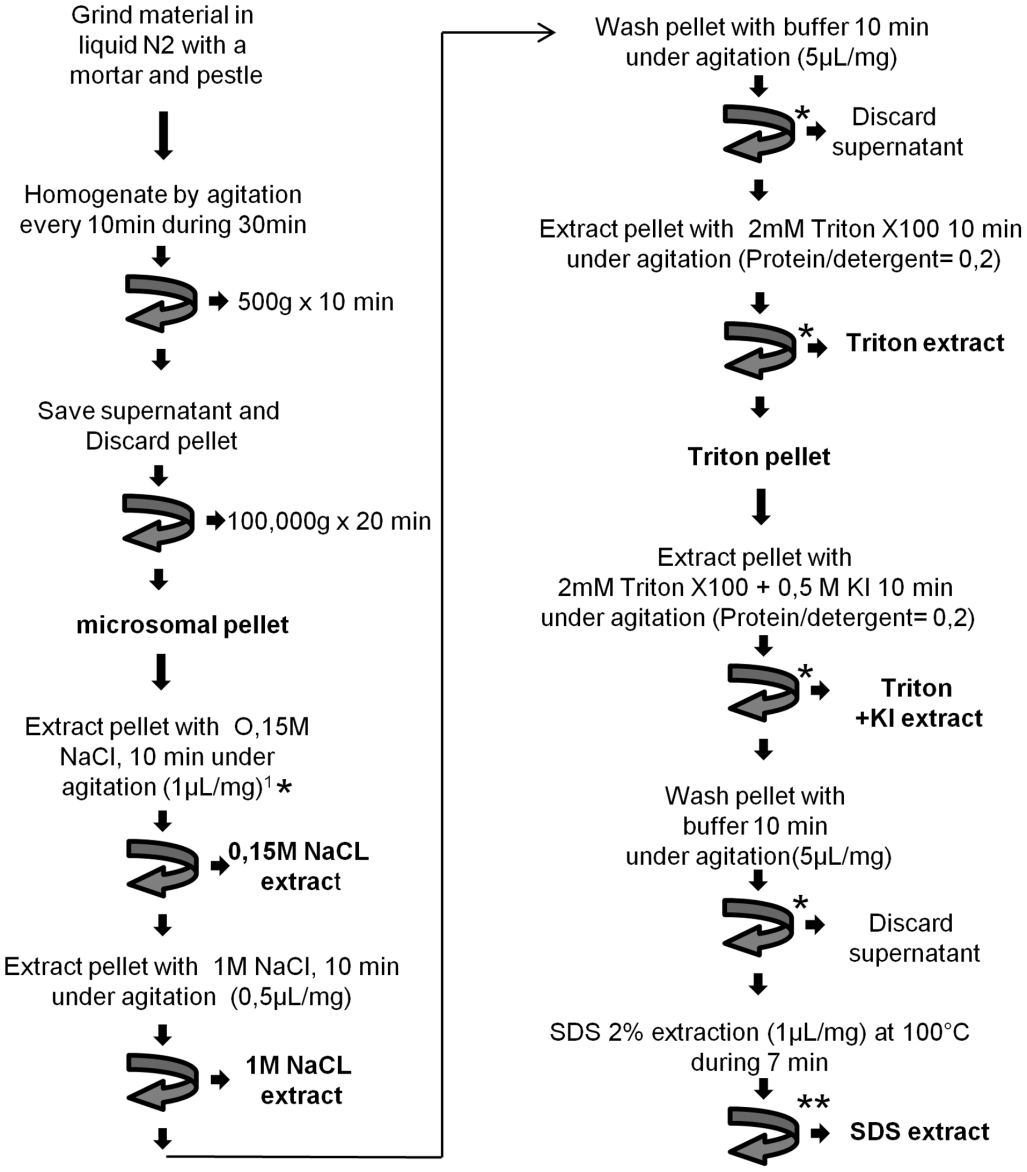
Workflow of microsome preparation and sequential extraction of proteins with salts and detergents. ^1^ Volumes of buffer are expressed as µl per mg of initial fresh material used. * Centrifugation 100 000 g, 20 min; ** Centrifugation 15 000 g 15 min.

The general workflow for microsome preparation is described in Fig. 3. Briefly, an amount of freeze-dried powder corresponding to 200 to 600 mg of frozen material was suspended in 1 ml of ice-cold buffer A containing 50 mM Tris-HCl pH 7.5, 10 mM DTT, 0.1 mM AEBSF. The suspension was homogenized 4 times on ice every 10 min using a Dounce homogenizer with a small clearance pestle for 30 min.

The suspension was centrifuged at 500 g for 10 min to separate the debris and dense material (cell walls, nuclei) from the supernatant containing the microsomes and soluble material. The resulting supernatant was further centrifuged at 100,000 g for 20 min. The resulting microsomal pellet was subsequently extracted according to the workflow illustrated in Fig. 3.

### Preparation of Samples for Mass Spectrometry Analysis

The microsomal fractions were boiled for 30 min at 95°C in Laemmli buffer to reduce and denature the proteins which were subsequently alkylated with 90 mM iodoacetamide for 30 min at room temperature in the dark. The protein concentration was determined using a detergent-compatible assay (Bio-Rad Protein Assay Kit, INTERCHIM France) according to the manufacturer’s instructions. Fifty micrograms of proteins from each microsomal extract were concentrated in a single band on a 12% acrylamide SDS-PAGE gel and visualized by colloidal Coomassie Blue staining. The single band, containing the whole sample, was cut out from the top of the resolving gel and washed in 50 mM ammonium bicarbonate for 15 min at 37°C followed by a second wash in 50 mM ammonium bicarbonate, acetonitrile (1∶1) for 15 min at 37°C. Trypsin (Promega, Charbonnières, France) digestion was performed overnight at 37°C. The resulting peptides were extracted from the gel in three steps: a first incubation step in 50 mM ammonium bicarbonate for 15 min at 37°C and two incubations in 10% formic acid, acetonitrile (1∶1) for 15 min at 37°C. The three extracts were pooled with the initial digestion supernatant, dried under vacuum in a SpeedVac, and resuspended with a solution containing 2% acetonitrile, and 0.05% trifluoroacetic acid.

### Detailed LC-MS/MS Analysis, Data Search and Validation

The peptide mixtures were analyzed by nano-LC-MS/MS using an Ultimate3000 system (Dionex) coupled to an LTQ-Orbitrap Velos mass spectrometer (Thermo Fisher Scientific, Bremen, Germany) according to the workflow described in Fig. S3. Five microliters of each sample were loaded on a C18 precolumn (300 µm inner diameter ×5 mm; Dionex) at 20 µl/min in 5% acetonitrile, 0.05% trifluoroacetic acid. After 5 min of desalting, the precolumn was switched on line with the analytical C18 column (75-µm inner diameter × 15 cm; in-house packed) equilibrated in 95% solvent A (5% acetonitrile, 0.2% formic acid) and 5% solvent B (80% acetonitrile, 0.2% formic acid). Peptides were eluted using a 5–50% gradient of solvent B for 110 min at a 300 nl min^−1^ flow rate. The LTQ-Orbitrap was operated in data-dependent acquisition mode with Xcalibur software. Survey scan MS spectra were acquired in the Orbitrap in the 350–2000 *m*/*z* range with the resolution set at 60,000. The twenty most intense ions per survey scan were selected for collision-induced dissociation fragmentation, and the resulting fragments were analyzed in the linear trap (LTQ). Dynamic exclusion was used within 60 s to prevent repetitive selection of the same peptide. Mascot Daemon software (version 2.3.2; Matrix Science, London, UK) was used to perform database searches, using the Extract_msn.exe macro provided with Xcalibur (version 2.0 SR2; Thermo Fisher Scientific) to generate peaklists. The following parameters were set for the creation of the peaklists: parent ions in the mass range 400–4500, no grouping of MS/MS scans, and threshold set at 1000. A peaklist was created for each fraction analyzed, and individual Mascot (version 2.3.2) searches were performed for each fraction. The data were searched against Arabidopsis entries in the Swiss-Prot TrEMBL database (53809 sequences*).* Carbamidomethylation of cysteines was set as a fixed modification and oxidation of methionine was set as a variable modification. Specificity of trypsin digestion was set for cleavage after Lys or Arg, and one missed trypsin cleavage site was allowed. The mass tolerances in MS and MS/MS were set to 10 ppm and 0.8 Da, respectively, and the instrument setting was specified as “ESI-Trap.” Mascot results were parsed with the in-house developed software Mascot File Parsing and Quantification (MFPaQ) version 4.0 [46]. To evaluate false positive rates, all the initial database searches were performed using the decoy option of Mascot, i.e. the data were searched against a combined database containing the real specified protein sequences and the corresponding reversed protein sequences (decoy database). MFPaQ used the same criteria to validate decoy and target hits, calculated the false discovery rate (FDR; FDR = number of validated decoy hits/[number of validated target hits+number of validated decoy hits] ×100) for each sample analyzed.

Protein hits were validated automatically using MFPaQ with less than 1.3% FDR (minimum length of eight amino acid peptide). All the validated proteins containing at least two peptides were imported in the ProteinCenter software (Proxeon Bioinformatics, Odense, Denmark, www.proxeon.com. Accessed 2014 February 18) in which statistical, comparative and sorting analysis was carried out.

### Relative Quantification of Proteins

Proteins were quantified using the label-free module implemented in the MFPaQ v4.0.0 software (http://mfpaq.sourceforge.net/. Accessed 2014 February 18). For each sample, the software uses the validated identification results and extracts ion chromatograms (XIC) of the identified peptide ions in the corresponding raw nano LC-MS files, based on their experimentally measured retention time (RT) and monoisotopic *m*/*z* values. The time value used for this process is retrieved from Mascot result files, based on an MS2 event matching to the peptide ion. If several MS2 events were matched to a given peptide ion, the software checked the intensity of each corresponding precursor peak in the previous MS survey scan. The time of the MS scan that exhibited the highest precursor ion intensity was attributed to the peptide ion and then used for XIC extraction as well as for the alignment process. Peptide ions identified in all the samples to be compared were used to build a retention time matrix to align LC-MS runs. If some peptide ions were sequenced by MS/MS and validated only in some of the samples to be compared, their XIC signal was extracted in the nanoLC-MS raw file of the other samples using a predicted RT value calculated from this alignment matrix by a linear interpolation method. Quantification of peptide ions was performed based on calculated XIC area values. To perform a relative quantification in different samples, the abundance of a protein was defined as the sum of XIC area values for all the tryptic peptides identified for this protein. The quantification results were taken into account if at least two peptides could be quantified. To perform normalization of a group of comparable samples, the software computed the XIC area ratios for all the extracted signals between a reference run and all the other runs of the group and used the median of the ratios as a normalization factor.

### Statistical Analysis of MS/MS Data

Statistical analysis was performed using R [47]. Before analysis, a logarithmic transformation of the abundance data was performed to reduce the skewness of the distribution. For each of the 1484 proteins quantified in the three conditions a mean abundance value was calculated from the values measured in the fractions where the protein was quantified. In order to test the statistical significance of the abundance ratios (0 g space/1 g space, 0 g space/1 g ground, 1 g space/1 g ground), we assumed that the abundance values followed the same distribution within each condition. Parameters of this distribution were estimated from three control samples taken from 1 g samples grown on board the ISS which were extracted and analyzed by MS/MS in exactly the same conditions as the samples grown in µg in space or 1 g on ground. The log-ratios between control samples were then calculated. Good repeatability of the data was observed between replicates (Fig. S4A). As expected, higher variability was observed between samples 0 g and 1 g from the space experiment (Fig. S4B). The histogram of the log-ratios showed a centered and symmetrical bell-shape distribution (Fig. S5). Q-Q plots revealed that the distribution of log-ratios was closer to a Laplace distribution than to a normal distribution (Fig. S6). Therefore, using the R package VGAM [48], a Laplace distribution was fitted to the log-ratio distribution in control samples and then used to calculate p-values, i.e. probabilities that 1 g/0 g ratios observed in the space experiment were due to chance rather than to gravity effects (Fig. S7). In order to control the false discovery rate in the lists of selected proteins, a Benjamini & Hocheberg correction was applied to the p-values using the Multitest R-package [49]. A protein was considered as significantly over- or under-represented in one condition compared to another when the corrected p-value associated to the corresponding ratio was lower than 0.05.

## Results

### Phenotypes of Space-grown Seedlings do not Significantly Differ from 1 g-grown Seedlings

Observations made upon receipt of samples from space indicate that most of the *Arabidopsis* seeds germinated correctly with a germination rate close to 100% and gave seedlings that apparently grew similarly whatever the gravity conditions (data not shown). This apparent homogeneity in the biomass production of plants whatever the conditions, allowed us to compare samples in terms of distribution of proteins extracted from similar amount of total protein in either conditions.

### Microsomal Proteins were Identified in all the Tested Conditions

In order to successfully analyze membrane-associated proteins in the different samples, a selective extraction of membrane proteins from microsomes was performed using a sequential extraction protocol with salts and detergents. This sequential extraction method separated proteins from a given membrane compartment into much less complex molecular mixtures, thus simplifying the sample for subsequent analysis by mass spectrometry, while maintaining the diversity and plurality of the proteome, and thus increasing the efficiency of protein identification and quantification (see experimental procedures section).

**Figure 4:**
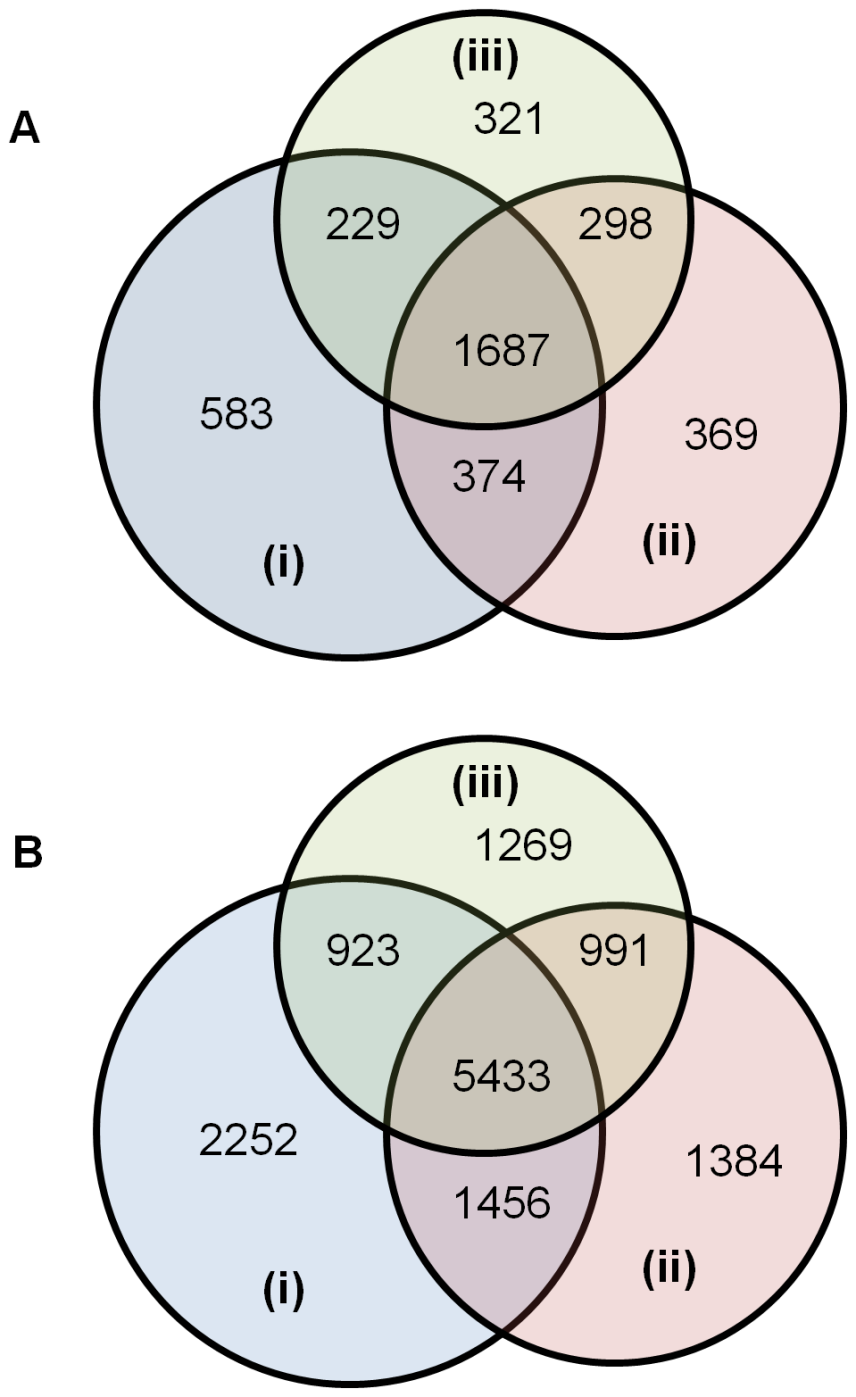
Venn diagrams of proteins identified on the basis of at least two peptides. A. Number of proteins, B. Number of corresponding sequenced peptides from microsomal samples isolated from seedlings grown on board the ISS in microgravity (i), 1 g on centrifuge (ii) or 1 g on Earth (iii). Total number of proteins identified is 3861 and total number of corresponding peptides is 13 708.

Based on this protocol we sequenced 13 708 peptides corresponding to 3861 proteins which were identified each with at least two validated peptides: 1687 proteins (43.7%–5433 peptides) were found in the 3 conditions, 583 proteins (15.1%–2252 peptides) were specific to the µg condition whereas 321 proteins (8.3%–1269 peptides) and 369 proteins (9.6%–1384 peptides) are specific to 1 g condition on ground and ISS respectively (Fig. 4 ab).

Among the 3861 proteins identified, 1267 of them are associated to the GO term “membrane” (GO:0016020) in the Gene Ontology “Cellular Component”, and 2188 (56.6%) have at least one transmembrane segment. This large proportion of membrane related proteins validates our approach although we are aware that some proportion of proteins not associated to this GO term could be soluble proteins artifactually co-purified with the microsomes, but also soluble proteins which have undergone post-translational modifications directing them to membranes under microgravity condition.

**Figure 5:**
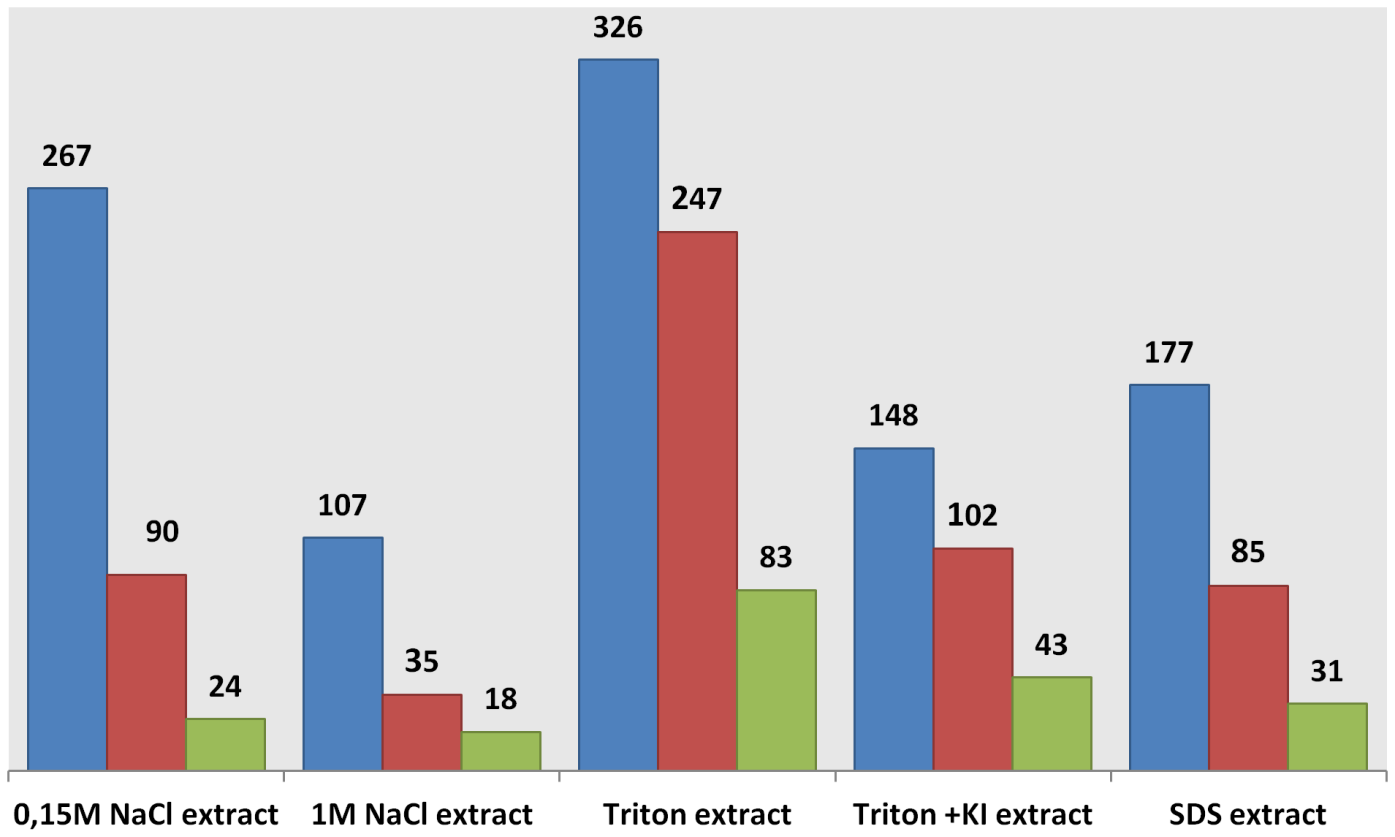
Proteins specifically identified in one extraction fraction and common to the three gravity conditions (i.e. 1 g ground, 1 g space and µg space). Blue bars indicate the total number of proteins identified. Red bars show the number of proteins with at least one transmembrane segment and the green bars the number of proteins which are annotated as “membrane” in Gene Ontology (The number of proteins is indicated at the top of the bars).

Fig. 5 shows the distribution of the set of 1025 proteins that were identified in only one of the 5 extraction conditions (i.e. specific to the extraction method used). By using the ProteinCenter software, data analysis shows that the GO term “membrane” (GO:0016020) is associated to numerous proteins in each extract. Even in the salt extract assumed to contain hydrophilic proteins, up to 33% of the proteins have a transmembrane segment. In a similar way, proteins with at least one transmembrane segment are distributed throughout the five extracts. As expected, the percentage of membrane proteins is much higher and close to 100% in the extracts obtained with Triton detergent or chaotropics such as potassium iodide salts (Fig. 5).

### Relative Quantification of Microsomal Proteins

The relative quantity of membrane-associated proteins from µg and 1 g from the EMCS centrifuge was evaluated using a label-free mass spectrometry method based on the measurement of the precursor signal intensity as described in the “Methods” section. In order to determine the threshold ratios above which proteins are considered as significantly “over” or “under”-represented in µg, statistical analysis of the mean abundance values was performed in the fractions where the proteins were detected.

The unavailability of true biological repetitions in space prevented us from using standard statistical tests to determine the significance of the ratios calculated, e.g. microgravity (µg) vs simulated gravity in space (1 g), or simulated gravity vs ground gravity. To circumvent this problem we made two assumptions: 1) the error distribution of abundance measurements was identical for each protein, and 2) this distribution was the same in all conditions (µg, 1 g space, or 1 g ground). This distribution was thus estimated from control samples and used to compute p-values associated to the ratios (see Materials & Methods section Figures. S4–S7).

**Only 69 proteins in the microsomal fraction were found to be significantly less abundant and 80 were found to be more abundant under microgravity conditions than under gravity (1 g in space or on ground).**

Among the 1687 proteins identified with at least 2 peptides, 1484 (97%) were quantified and are listed in Table S1. The statistical method described in experimental procedures was then applied to determine the level of proteins whose abundance was significantly changed between µg and 1 g conditions obtained both in space and on the ground. Furthermore, in order to be more specific in terms of microgravity effect, we considered here only proteins whose abundance did not change significantly (BH >0.05) between the two 1 g conditions (in space and on the ground).

The number of microsomal proteins that significantly underwent quantitative changes in microgravity as compared to 1 g conditions was very low i.e. only 5% of the total number of proteins quantified in the 3 conditions (µg space, 1 g space and 1 g ground). Using these conditions, the proteins that reached the threshold for a significant change are listed in Table 1 and 2. Table 1 displays the 69 proteins significantly (BH<0.05) under-represented (i.e. less abundant in microsomes) and Table 2 displays the 80 proteins significantly over-represented (i.e. more abundant in microsomes), with an average fold change of about 2.1 and 2.3 respectively. Analysis of the statistical distributions of ratios shows that in each group about half of the fold changes were greater than 2 (Fig. S8 and S9).

**Table 1:** List of membrane proteins significantly under-represented in microgravity conditions on board the ISS.

UniProt	AGI	Ratios	Protein names
**Signalling**			
P93025	AT5G58140	0.51024	Phototropin-2 (EC 2.7.11.1) (Defective in chloroplast avoidance protein 1) (Non-phototropic hypocotyl 1-like protein 1) (AtKin7) (NPH1-like protein 1)
Q39192	AT3G50500	0.55803	Serine/threonine-protein kinase SRK2D (EC 2.7.11.1) (OST1-kinase-like 3) (Protein ATHPROKIN A) (SNF1-related kinase 2.2) (SnRK2.2)
Q39253	AT2G38170	0.36764	Vacuolar cation/proton exchanger 1 (Ca(2+)/H(+) antiporter CAX1) (Ca(2+)/H(+) exchanger 1) (Protein CATION EXCHANGER 1) (Protein RARE COLD INDUCIBLE 4)
Q96262	AT4G20260	0.73542	Plasma membrane-associated cation-binding protein 1 (AtPCAP1) (Microtubule-destabilizing protein 25)
**Miscellaneous**			
O04309	AT3G16470	0.62001	Myrosinase-binding protein-like At3g16470
P15459	AT4G27160	0.47098	2S seed storage protein 3 (2S albumin storage protein) (NWMU2-2S albumin 3)
P38666	AT3G53020	0.42956	60S ribosomal protein L24-2 (Protein SHORT VALVE 1)
Q42586	AT3G54470	0.53952	Uridine 5′-monophosphate synthase (UMP synthase)
Q8L7S8	AT5G26742	0.55758	DEAD-box ATP-dependent RNA helicase 3, chloroplastic (EC 3.6.4.13) (Protein EMBRYO DEFECTIVE 1138)
Q9C5M3	AT1G78970	0.57192	Lupeol synthase 1 (AtLUP1) (EC 4.2.1.128) (EC 5.4.99.41) (Lupan-3-beta,20-diol synthase)
Q9CAU9	AT3G04830	0.51415	Contains similarity to O-linked GlcNAc transferases (Expressed protein) (Prenylyltransferase-like protein) (Putative uncharacterized protein T9J14.22)
Q9FZ47	AT1G16880	0.5611	ACT domain-containing protein (At1g16880) (F6I1.12 protein) (Putative uncharacterized protein F6I1.12) (Uridylyltransferase-related protein)
Q9LKA6	AT3G14990	0.51174	Class I glutamine amidotransferase domain-containing protein (Emb|CAA17570.1)
Q9LTB2	AT5G49810	0.53697	Methionine S-methyltransferase (EC 2.1.1.12) (AdoMet:Met S-methyltransferase)
Q9LUJ7	AT3G22640	0.53207	AT3g22640/MWI23_1 (Cupin family protein)
Q9SUR3	AT4G23630	0.45225	Reticulon-like protein B1 (AtRTNLB1) (VirB2-interacting protein 1)
Q9T076	AT4G27520	0.41046	Early nodulin-like protein 2 (Phytocyanin-like protein)
**Lipid metabolism**			
F4HYI6	AT1G48600	0.56281	Putative phosphoethanolamine N-methyltransferase 2
O04983	AT5G35360	0.52901	Biotin carboxylase, chloroplastic (EC 6.3.4.14) (Acetyl-CoA carboxylase subunit A) (ACC) (EC 6.4.1.2)
Q9LD43	AT2G38040	0.33612	Acetyl-coenzyme A carboxylase carboxyl transferase subunit alpha, chloroplastic (ACCase subunit alpha) (EC 6.4.1.2)
**Chloroplast**			
P10798	AT5G38410	0.35817	Ribulose bisphosphate carboxylase small chain 3B, chloroplastic (RuBisCO small subunit 3B) (EC 4.1.1.39)
Q944I4	AT1G80380	0.3499	D-glycerate 3-kinase, chloroplastic (AtGLYK) (EC 2.7.1.31)
Q41932	AT4G05180	0.51641	Oxygen-evolving enhancer protein 3-2, chloroplastic (OEE3) (16 kDa subunit of oxygen evolving system of photosystem II) (OEC 16 kDa subunit)
**Proteases**			
Q9LEW2		0.36835	Nucleoid DNA-binding protein cnd41-like protein
Q9XEC4	AT4G04460	0.4244	Aspartic proteinase A3 (EC 3.4.23.-)
**Unknown**			
Q8L9T0		0.52193	Putative uncharacterized protein
Q94EI1		0.52207	At1g44575/T18F15
Q9LYE7	AT5G11420	0.36003	Putative uncharacterized protein At5g11420 (Putative uncharacterized protein F15N18_10)
Q9SIL0		0.5859	Putative uncharacterized protein At2g12400 (Putative uncharacterized protein At2g12400; F24C20.8)
Q9SLV3	AT1G58270	0.53874	At1g58270/F19C14_8 (F19C14.11 protein) (Putative uncharacterized protein At1g58270) (TRAF-like protein) (ZW9 protein)
Q9STW1	AT4G24330	0.35208	At4g24330 (Putative uncharacterized protein AT4g24330) (Putative uncharacterized protein T22A6.160) (Uncharacterized protein)
Q9XI93	AT1G13930	0.53415	At1g13930/F16A14.27 (F16A14.14) (F7A19.2 protein) (Uncharacterized protein)
**Respiratory chain**			
O49313	AT2G33220	0.50406	NADH dehydrogenase [ubiquinone] 1 alpha subcomplex subunit 13-B
P56774	ATCG00730	0.39674	Cytochrome b6-f complex subunit 4 (17 kDa polypeptide)
**Nucleosome**			
O23629	AT3G45980	0.36438	Histone H2B.6 (H2BAt) (HTB9)
P26568	AT1G06760	0.46852	Histone H1.1
Q94F49	AT5G27670	0.21652	Probable histone H2A.5 (HTA7)
Q9FJE8	AT5G59870	0.23441	Probable histone H2A.7 (HTA6)
Q9LQQ4	AT1G07790	0.34323	Histone H2B.1 (HTB1)
**Cell wall metabolism**			
Q9FM65	AT5G55730	0.3768	Fasciclin-like arabinogalactan protein 1
O22126	AT2G45470	0.87579	Fasciclin-like arabinogalactan protein 8 (AtAGP8)
Q39099	AT2G06850	0.53895	Xyloglucan endotransglucosylase/hydrolase protein 4 (At-XTH4) (XTH-4) (EC 2.4.1.207)
Q9LIS3	AT3G23820	0.45798	UDP-glucuronate 4-epimerase 6 (EC 5.1.3.6) (UDP-glucuronic acid epimerase 6) (AtUGlcAE2)
Q9SG80	AT3G10740	0.58384	Alpha-L-arabinofuranosidase 1 (AtASD1) (EC 3.2.1.55) (Beta-D-xylosidase) (EC 3.2.1.-)
**Glycolysis**			
O65581	AT4G26530	0.56419	Fructose-bisphosphate aldolase (EC 4.1.2.13)
Q9LF93		0.1573	Pyruvate kinase (EC 2.7.1.40)
**Transport**			
A1L4Y1	AT3G10350	0.5503	Anion-transporting ATPase family protein (At3g10350)
A8MR14	AT1G64650	0.56728	General substrate transporter-like protein (Uncharacterized protein At1g64650.2)
O23482	AT4G16370	0.29141	Oligopeptide transporter 3 (AtOPT3)
P26587	AT1G73190	0.48634	Aquaporin TIP3-1 (Alpha-tonoplast intrinsic protein) (Alpha-TIP) (Tonoplast intrinsic protein 3-1) (AtTIP3;1)
Q8L856	At4G25570	0.53011	Transmembrane ascorbate ferrireductase 1 (EC 1.16.5.1) (Cytochrome b561) (Artb561-1) (AtCytb561) (Tonoplast Cyt-b561) (TCytb)
Q9LJX0	AT3G28860	0.29547	ABC transporter B family member 19 (ABC transporter ABCB.19) (AtABCB19) (Multidrug resistance protein 11) (P-glycoprotein 19)
Q9M817	AT1G52190	0.39512	Probable peptide transporter At1g52190
Q9SMV6	AT5G17020	0.35214	Exportin 1A (Exportin1 (XPO1) protein) (Putative Exportin1 (XPO1) protein) (Putative exportin1 protein XPO1)
**Cytoskeleton**			
B9DGT7	At1G50010	0.6263	Tubulin alpha-2 chain
P29513	AT1G20010	0.47936	Tubulin beta-5 chain (Beta-5-tubulin)
P53492	AT5G09810	0.6687	Actin-7 (Actin-2)
Q0WV25	At1G04820	0.6263	Tubulin alpha-4 chain
Q56YW9	At5G62690	0.43921	Tubulin beta-2 chain
Q96292	AT3G18780	0.53391	Actin-2
Q9ASR0	At5G62700	0.43921	Tubulin beta-3 chain
**Oxidoreductases**			
Q8LCQ9		0.41902	NADP-dependent malate dehydrogenase
Q94B78	AT4G33010	0.44161	Glycine dehydrogenase [decarboxylating] 2, mitochondrial (EC 1.4.4.2) (Glycine cleavage system P protein 2) (Glycine decarboxylase 2)
Q9SAE3	AT1G13090	0.47906	Cytochrome P450 71B28 (EC 1.14.-.-)
Q9SGS4	AT1G76080	0.55624	Thioredoxin-like protein CDSP32, chloroplastic (Chloroplastic drought-induced stress protein of 32 KDa) (AtCDSP32)
**Membrane trafficking**			
O23140	AT5G46630	0.59061	AP-2 complex subunit mu-1 (AP47/50p)
O80977	AT2G14740	0.55033	Vacuolar-sorting receptor 3 (AtVSR3) (BP80-like protein a’) (AtBP80a’) (Epidermal growth factor receptor-like protein 2a) (AtELP2a)
Q56WK6	AT1G72150	0.58578	Patellin-1
Q9XFM6	AT5G52240	0.55566	Membrane steroid-binding protein 1 (AtMP1)

**Table 2:** List of membrane proteins significantly over represented in microgravity conditions on board the ISS.

UniProt	AGI	Ratios	Protein names
**Signalling**			
Q08466	AT3G50000	2.8638	Casein kinase II subunit alpha-2 (CK II) (EC 2.7.11.1)
Q9SRQ7	AT3G03530	3.2845	AT3g03530/T21P5_5 (At3g03530/T21P5_5) (Phosphatidylglycerol specific phospholipase C) (Phospholipase C) (T21P5.5 protein)
Q8VZG1	AT2G18730	2.1248	At2g18730/MSF3.11 (Diacylglycerol kinase 3)
P25071	At2G41100	3.679	Calmodulin-like protein 12 (Touch-induced calmodulin-related protein 3)
**Lipid metabolism**			
O48917	AT4G33030	2.1263	UDP-sulfoquinovose synthase, chloroplastic (EC 3.13.1.1) (Sulfite:UDP-glucose sulfotransferase) (Sulfolipid biosynthesis protein)
Q8L5Z1	AT1G33811	2.5715	GDSL esterase/lipase At1g33811 (EC 3.1.1.-) (Extracellular lipase At1g33811)
Q8LPS1	At3G05970	2.7547	Long chain acyl-CoA synthetase 6, peroxisomal (EC 6.2.1.3)
Q9M7Z1	AT3G06850	1.7488	2-oxoisovalerate dehydrogenase E2 component (Dihydrolipoyl transacylase) (AT3G06850 protein) (Branched chain alpha-keto acid dehydrogenase E2 subunit)
Q9SJD4	At2G04350	1.7513	Long chain acyl-CoA synthetase 8 (EC 6.2.1.3)
**Stress Defence**			
P50700	AT4G11650	2.4094	Osmotin-like protein OSM34
Q06327	AT1G55020	4.1369	Linoleate 9S-lipoxygenase 1 (EC 1.13.11.58) (Lipoxygenase 1) (AtLOX1)
Q683G1		2.1047	Similar to senescence-associated protein
Q8VZG8	AT4G08850	2.9398	Probable LRR receptor-like serine/threonine-protein kinase At4g08850 (EC 2.7.11.1)
Q941L3	AT5G61900	1.7347	Protein BONZAI 1 (COPINE 1)
Q94AJ8		2.3775	Putative beta-1,3-glucanase
Q9LSP5	AT3G17020	1.9398	AT3g17020/K14A17_14 (Gb|AAF26101.1) (Universal stress protein (USP) family protein)
Q9SRH6	AT3G01290	2.7526	Hypersensitive-induced response protein 3 (AtHIR3)
**Amino-acid synthesis**			
Q1EBW2	AT5G19550	1.7205	Aspartate aminotransferase (EC 2.6.1.1)
Q9SZJ5	AT4G37930	1.512	Serine hydroxymethyltransferase, mitochondrial (SHMT) (EC 2.1.2.1) (Glycine hydroxymethyltransferase) (Serine methylase)
**Chloroplast**			
O22773	AT4G02530	1.5287	Thylakoid lumenal 16.5 kDa protein, chloroplastic
Q1H537	AT5G18660	1.9528	At5g18660 (Rossmann-fold NAD(P)-binding domain-containing protein)
Q2HIV2	AT3G25760	3.8866	At3g25760
Q9FYC2	AT3G44880	2.5419	Pheophorbide a oxygenase, chloroplastic (AtPaO) (Pheide a oxygenase) (EC 1.14.12.20) (Accelerated cell death 1) (Lethal leaf-spot 1 homolog) (Lls1)
Q9LW57	AT3G23400	1.4798	Probable plastid-lipid-associated protein 6, chloroplastic (Fibrillin-6) (AtPGL30.4) (Harpin-binding protein 1) (HrBP1)
**Proteases**			
O49607	AT4G34980	1.7715	Putative subtilisin serine protease (Subtilisin proteinase) (Subtilisin-like serine protease 2)
Q9STL8	AT3G48380	1.9345	Probable Ufm1-specific protease (UfSP) (EC 3.4.22.-)
**Unknown**			
O04551	AT1G27020	2.1419	Putative uncharacterized protein At1g27020 (T7N9.8) (Uncharacterized protein)
O04616	AT4G01150	2.3716	Uncharacterized protein At4g01150, chloroplastic
O23601		1.8203	Putative uncharacterized protein AT4g17600 (Putative uncharacterized protein dl4835w)
Q8H0W8		1.9158	Putative uncharacterized protein At5g38660
Q8VYV1		2.3485	AT5g08050/F13G24_250 (At5g08050/F13G24_250)
Q93VA8	AT1G76010	1.7247	Alba DNA/RNA-binding protein (At1g76010/T4O12_22)
Q93W02	AT5G24690	1.9446	AT5g24690/MXC17_8 (Putative uncharacterized protein At5g24690) (Putative uncharacterized protein At5g24700) (Uncharacterized protein)
Q94CD9		1.7983	Putative uncharacterized protein At4g33360 (Fragment)
Q9LIL4	AT3G22845	2.9425	Transmembrane emp24 domain-containing protein p24beta3 (p24 family protein beta2) (p24beta2) (p24 family protein beta3) (p24beta3)
Q9LTB7		2.1824	Lil3 protein
Q9LVM3	AT5G58250	2.2543	Uncharacterized protein
Q9M1J1	AT3G57090	2.1494	AT3g57090/F24I3_170 (Mitochondrial fission protein AtFIS1a) (Protein BIGYIN1) (Putative uncharacterized protein F24I3.170)
Q9SH90		1.7123	Putative uncharacterized protein At2g37970 (Putative uncharacterized protein At2g37970; T8P21.12)
**Tricarboxylic cycle**			
P57106	AT5G43330	2.0606	Malate dehydrogenase, cytoplasmic 2 (EC 1.1.1.37)
Q94A28	AT4G26970	1.7543	Aconitate hydratase 3, mitochondrial (Aconitase 3) (EC 4.2.1.3) (Citrate hydro-lyase 3)
**Hormone biosynthesis**			
P38418	AT3G45140	2.8506	Lipoxygenase 2, chloroplastic (AtLOX2) (EC 1.13.11.12)
Q9LS02	AT3G25770	2.961	Allene oxide cyclase 2, chloroplastic (EC 5.3.99.6)
Q9M0X9	AT4G05160	1.7386	4-coumarate–CoA ligase-like 7 (EC 6.2.1.-) (4-coumarate–CoA ligase isoform 6) (At4CL6)
**Miscellaneous**			
B9DHS6		2.0934	AT3G01480 protein (Fragment)
Q38924	AT2G27190	2.2812	Fe(3+)-Zn(2+) purple acid phosphatase 12 (PAP) (EC 3.1.3.2) (Iron(III)-zinc(II) purple acid phosphatase 12)
Q56WN1	AT5G37600	2.0578	Glutamine synthetase cytosolic isozyme 1-1 (EC 6.3.1.2) (Glutamate–ammonia ligase GLN1;1) (GLN1;1)
Q94AF6	AT5G20160	2.9457	AT5g20160/F5O24_50 (Ribosomal protein L7Ae-like) (U4/U6 small nuclear ribonucleoprotein SNU13)
Q9FVQ1	AT1G48920	1.781	Nucleolin 1 (Protein NUCLEOLIN LIKE 1) (AtNUC-L1) (Protein PARALLEL 1) (AtPARL1)
Q9LSE4	AT3G26710	1.8275	At3g26710 (Cofactor assembly of complex C) (Emb|CAA66820.1) (Expressed protein) (Putative uncharacterized protein At3g26710)
Q9M5P3	AT5G54290	2.3778	Cytochrome c-type biogenesis ccda-like chloroplastic protein (Cytochrome b6f biogenesis protein CCDA)
Q9SIV9	AT2G16430	1.8662	Purple acid phosphatase 10 (EC 3.1.3.2)
Q9SR40	AT3G09220	2.1287	Laccase-7 (EC 1.10.3.2) (Benzenediol:oxygen oxidoreductase 7) (Diphenol oxidase 7) (Urishiol oxidase 7)
Q9SRT9	AT3G02230	2.2652	UDP-arabinopyranose mutase 1 (EC 5.4.99.30) (Reversibly glycosylated polypeptide 1) (AtRGP1) (UDP-L-arabinose mutase 1)
Q9SX77	AT1G47420	1.7168	Uncharacterized protein At1g47420, mitochondrial
Q9ZVA2	AT1G78830	1.9959	At1g78830/F9K20_12 (Curculin-like (Mannose-binding) lectin-like protein) (F9K20.12 protein) (Putative uncharacterized protein At1g78830)
**Protein synthesis**			
A8MRC4	AT1G30230	2.435	Elongation factor 1-delta 1 (Uncharacterized protein At1g30230.2)
Q9FVT2	AT1G57720	2.0131	Probable elongation factor 1-gamma 2 (EF-1-gamma 2) (eEF-1B gamma 2)
Q9SCX3	AT5G19510	3.4476	Elongation factor 1-beta 2 (EF-1-beta 2) (Elongation factor 1-beta’ 2) (EF-1-beta’ 2) (Elongation factor 1B-alpha 2) (eEF-1B alpha 2)
**Transcription**			
Q84WU9	AT5G56900	2.0231	Zinc finger CCCH domain-containing protein 64 (AtC3H64)
Q9SRM4	AT3G11200	1.8897	PHD finger protein ALFIN-LIKE 2 (Protein AL2)
**Oxydoreduction**			
B1GV36		3.5288	Cinnamate-4-hydroxylase
O49340		2.7992	Cytochrome P450 71A12 (EC 1.14.-.-)
O65782	AT4G31500	1.879	Cytochrome P450 83B1 (EC 1.14.-.-) (Protein ALTERED TRYPTOPHAN REGULATION 4) (Protein RED ELONGATED 1) (Protein SUPERROOT 2)
O65787	AT2G24180	2.0196	Cytochrome P450 71B6 (EC 1.14.-.-)
O80852	AT2G30860	1.7945	Glutathione S-transferase F9 (AtGSTF9) (EC 2.5.1.18) (AtGSTF7) (GST class-phi member 9)
O81816	AT4G38540	2.2396	FAD/NAD(P)-binding oxidoreductase family protein (Monooxygenase) (Monooxygenase 2 (MO2))
Q94BV7	AT4G05020	2.4173	NAD(P)H dehydrogenase B2, mitochondrial (EC 1.6.-.-)
Q9SMU8	AT3G49120	7.3136	Peroxidase 34 (Atperox P34) (EC 1.11.1.7) (ATPCb)
Q9SRY5	AT1G02920	4.6163	Glutathione S-transferase F7 (EC 2.5.1.18) (AtGSTF8) (GST class-phi member 7) (Glutathione S-transferase 11)
Q9SU63	AT3G48000	1.7102	Aldehyde dehydrogenase family 2 member B4, mitochondrial (ALDH2a) (EC 1.2.1.3)
**Glycolysis**			
Q8RWN9	AT3G13930	1.7859	Dihydrolipoyllysine-residue acetyltransferase component 2 of pyruvate dehydrogenase complex, mitochondrial (EC 2.3.1.12 2)
Q96536		2.1072	Pyruvate decarboxylase (EC 4.1.1.1)
Q9LZR9	AT5G03690	2.4066	Fructose-bisphosphate aldolase (EC 4.1.2.13)
Q9SJQ9	AT2G36460	2.0721	Fructose-bisphosphate aldolase (EC 4.1.2.13)
**Transport**			
O80725	AT2G47000	2.5087	ABC transporter B family member 4 (ABC transporter ABCB.4) (AtABCB4) (Multidrug resistance protein 4) (P-glycoprotein 4)
Q8LE12		1.8569	Outer membrane lipoprotein-like
Q94EG9	AT1G55910	1.7651	Zinc transporter 11 (ZRT/IRT-like protein 11)
Q96303	AT2G38940	2.6498	Inorganic phosphate transporter 1–4 (AtPht1;4) (H(+)/Pi cotransporter)
Q9SAF5	AT1G13210	1.7937	Putative phospholipid-transporting ATPase 11 (AtALA11) (EC 3.6.3.1) (Aminophospholipid flippase 11)

**Figure 6:**
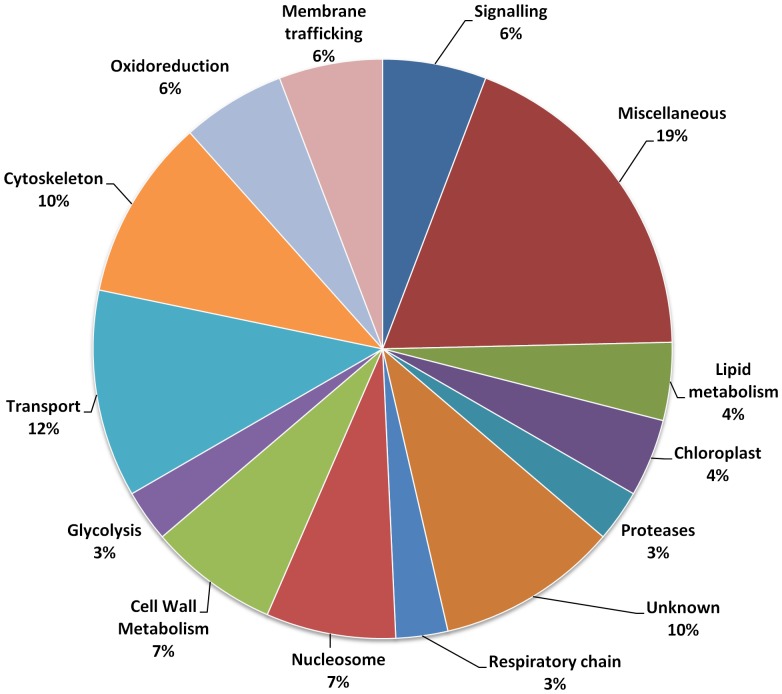
Distribution according their putative functions of the 69 proteins under-represented in microgravity (percentage of the total).

**Figure 7:**
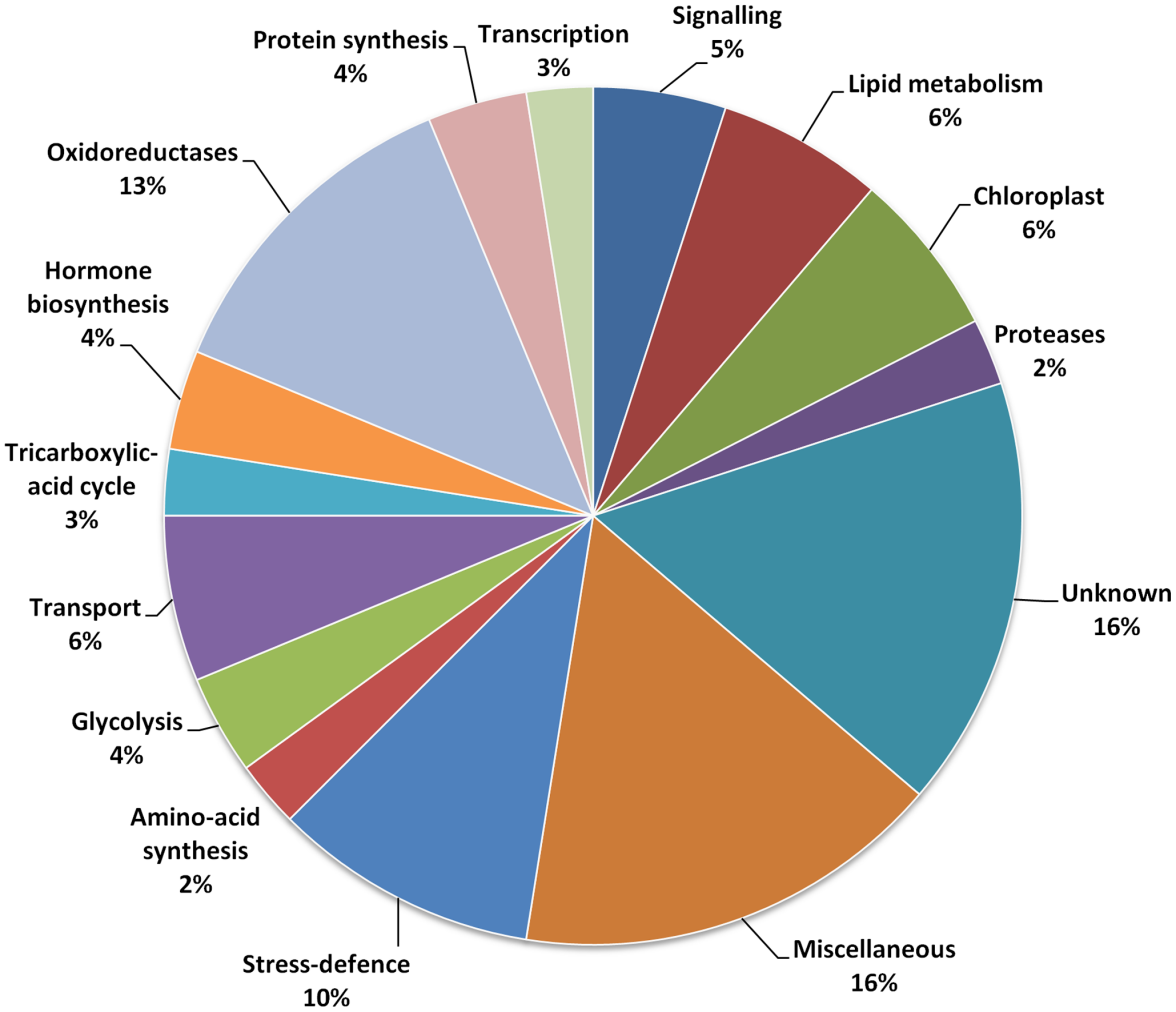
Distribution according to their putative functions of the 80 proteins over-represented in microgravity (percentage of the total).

We used functional annotations available in the UNIPROT database to manually cluster these proteins into functional categories. In each group, the largest protein cluster was the miscellaneous one, which groups protein categories represented by only one member (Fig. 6 and 7). This cluster can account for up to 20% for the under–represented proteins and up to 16% for the over represented group. The cluster of “unknown proteins” represented a similar number of proteins in the latter case, but was only half of that of the “miscellaneous” cluster in the under-represented group.

**Figure 8:**
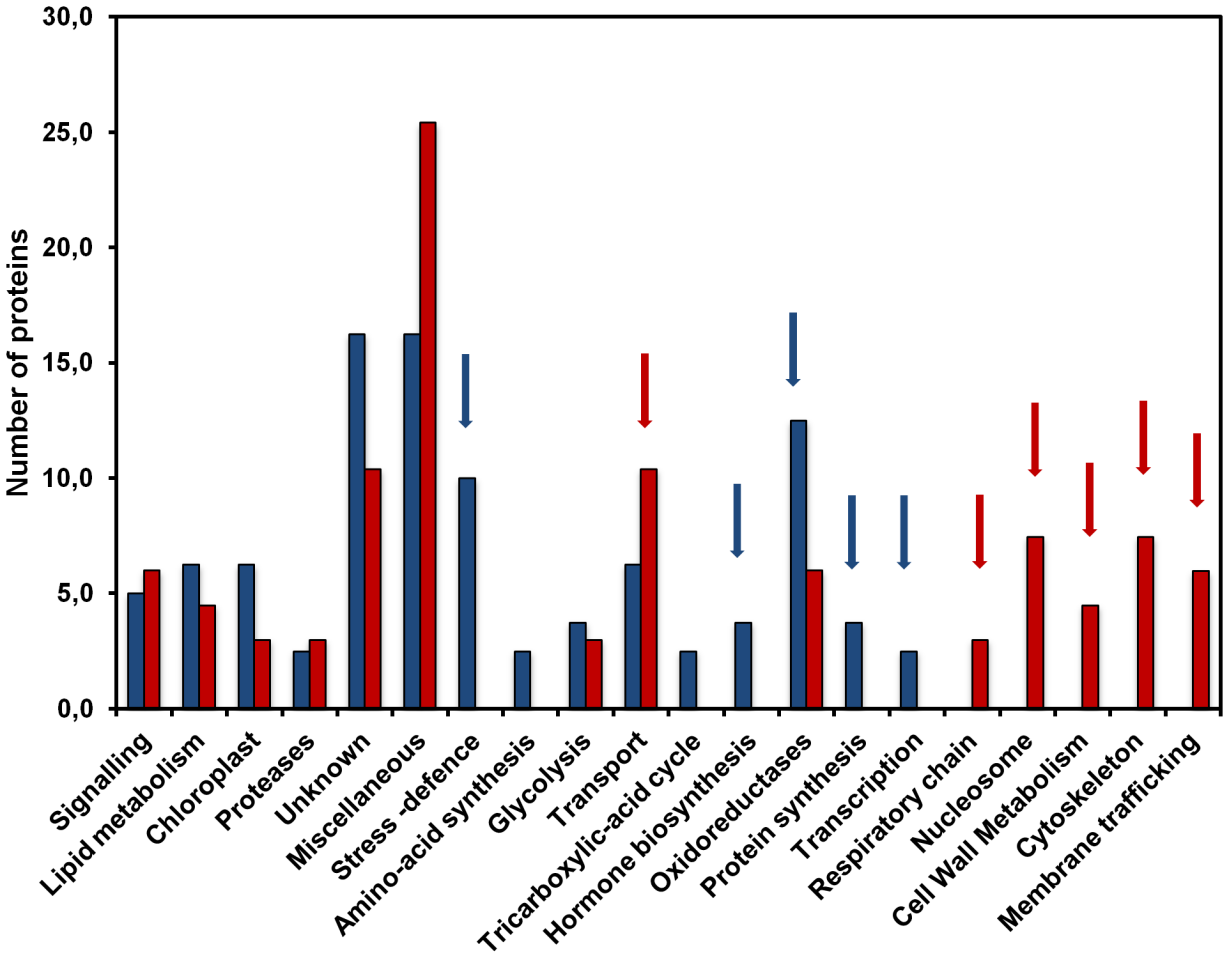
Comparative distribution of under and over-represented proteins according to functional categories. Black bars = over-represented, Grey bars = under-represented.

Considering the under-represented group (Fig. 6), proteins belonging to the cytoskeleton and transport clusters accounted for about 10% each and together represented one fifth of the total number of quantified proteins. Other important functional classes correspond to proteins involved in membrane trafficking, signaling, nucleosome, oxidoreduction and cell wall metabolism (Fig. 6). In the case of over-represented proteins (Fig. 7), the main functional classes concerned oxidoreduction and stress-defence proteins. A comparative analysis between the two groups (Fig. 8) pointed out some specificities in each group (see red and blue arrows): thus, nucleosome, respiratory chain, cell wall metabolism, cytoskeleton, membrane trafficking and to a lesser extent transport categories are specific to the group of under-represented proteins (Fig. 8 red arrows) whereas stress-defence, amino-acid and protein synthesis, TCA-cycle, hormone biosynthesis, transcription, and oxidoreduction categories are more specific to the group of proteins over-represented in microgravity conditions (Fig. 8 blue arrows).

## Discussion

Experiments in space imply several constraints. In particular, the amount of biological material available postflight is limited and it is difficult to increase the number of biological experiments in space. We developed a sequential extraction protocol using small amounts of young Arabidopsis seedlings in order to analyze the membrane proteins and those which are associated with the microsomal fraction by using mass spectrometry technology. With this specific protocol we were able to positively identify 3861 proteins, a third of them (1267) having a “membranes” annotation in Gene Ontology, and 2188 of them with at least one transmembrane segment. Others can only be considered associated with this cellular compartment either through stimulus-induced post-translational modifications such as prenylation, acetylation or myristoylation, or non-specific binding, in which case, the proteins would likely be soluble.

**Membrane-associated proteins related to auxin transport and metabolism, and protein trafficking are less abundant in microgravity conditions than in normal gravity.**

Careful analysis of 69 microsomal proteins significantly less abundant in space conditions (µg) indicate that several of them can be linked to one of the major processes already shown to be involved in gravity responses such as protein trafficking, signaling, cell wall modifications, auxin transport and metabolism, and stress-defence.

Thus, if we consider the functional group of “signaling proteins”, three proteins are particularly interesting: PHOT2, CAX1 and TOUCH3 (Table 1). The level of phototropin2 (PHOT2) is halved with respect to the control (1 g). This protein is encoded by the NON PHOTOTROPIC HYPOCOTYL 2 *(NPH2) locus* and belongs to the AGC kinase group and more specifically to the AGC4 group [50]. PHOT2 is a homolog of PHOT 1 and both are blue light receptors. PHOT1 is the major player in the phototrophic growth of Arabidopsis seedlings in low intensity light conditions, whereas PHOT2 has been shown to trigger a calcium-mediated phototrophic response under high light intensity [51]. This rise in calcium could have its importance in activating calcium sensors such as TOUCH3 and PINOID BINDING PROTEIN1 (PBP1), which are known to interact with another AGC kinase, namely PINOID (PID), recognized as a regulator of polar auxin transport [52]. Such calcium mediation is suspected to take part in the blue-light regulation of polar auxin transport (PAT) [53]. Interestingly TOUCH3 was increased almost 4-fold at membranes (see Over-represented proteins Table 2). Although speculative, a reason for that, could be a calcium-dependent regulation of PAT in microgravity conditions. Such calcium-regulation is consistent with the drop of the amount of CAX1 associated to membranes. CAX1 is known to drive calcium influx from the cytosol to the vacuole and proton influx from the vacuole to the cytosol [54]. Indeed, a decrease in the abundance of such exchanger at the vacuolar membrane should result in an increase of calcium in the cytosol that could be decoded by calcium binding proteins such as TOUCH3. The consequences of lowering the amount of CAX1 at the vacuolar membrane could also be viewed with respect to the phenotype observed in *cax1* mutants. In a working model with the Arabidopsis *cax1* mutant [54] speculates that H^+^ export into the apoplasm should be reduced both by the reduction of H^+^ export from vacuole to the cytosol and also because of the down-regulation of the plasma membrane proton ATPase AHA1 that leads to alkalization of the apoplast. This alkalization should down-regulate the auxin influx carrier AUX1, thus lowering the auxin concentration inside the guard cells. We can also hypothesize that the increase of calcium due to CAX1 impairment can lead to a possible calcium-dependent activation of PID by TOUCH3 which would finally impact on PIN1 trafficking [55]. This hypothesis is supported by the fact that we found concomitant down-regulation of the ABC transporter ABCB19 which is known to associate with PIN1 [56].

Interestingly, another signaling protein, microtubule-destabilizing protein 25 (MDP25) is also significantly less abundant at the membranes. MDP25 has been shown to be associated to the plasma membrane via N-myristoylation [57], and similarly, cortical microtubules have been shown to be associated to membranes through linker proteins [58]; furthermore MDP25 binds microtubules [59]. Using genetic approaches, the same group indicated that MDP25 negatively regulates cell elongation in the hypocotyl and that calcium induces the dissociation of MDP25 from the plasma membrane and migration towards the cytosol [59]. So, a lower abundance of the protein at the membranes in microgravity could result from migration of the protein to the cytosol, which would lead to cytoskeleton disorganization. Such a hypothesis is coherent with the observation that several subunits considered as building blocks of both microtubules and microfilaments were also found to be less associated to membranes under microgravity (see the cytoskeleton functional group Table1). It is worth noting that the two protein clusters related to cytoskeleton and membrane trafficking, and possibly linked to polar auxin trafficking [60] are specific to the group of proteins under-represented at membranes in microgravity conditions. Proteins demonstrated to be actors in membrane trafficking are also less abundant in membranes and this suggests that membrane trafficking is disturbed in microgravity conditions.

Interesting also is the lower abundance of methionine S-methyl transferase (SAM) (Table 1, miscellaneous group) which is involved in the biosynthesis of polyamines [61]. Polyamines being mainly involved in osmotic and drought stress and because SAM is much less represented in microgravity compared to 1 g indicates that gravity is perceived as a stress by the plant cells. More related to gravitropic responses, polyamines have been shown to be important since one of them i.e. spermine is able to rescue a gravitropic defect associated with the *adk* mutation [62]. In that perspective, our results suggest that microgravity controls the abundance of positive regulators of gravity.

Overall analysis of the functional role of proteins whose amounts appear significantly lowered at the membrane level suggests a working model where polar auxin transport would be reduced through dismantling of the cytoskeleton network, and mislocalisation and/or down-regulation of auxin transporters.

**Membrane-associated proteins related to metabolism and defence are more abundant in microgravity conditions.**

Proteins that are over-represented in microgravity, except for those already mentioned above, are mainly associated to metabolism or defence. It can be speculated that the over representation of proteins involved in glycolysis, the TCA cycle or amino acid and protein synthesis, will participate in the activated metabolism required for fueling the growth of seedlings in microgravity conditions. At the same time, we observe that some over-represented proteins are also markers of root starvation such as phosphate transporters and purple phosphatases [63]. The profile of proteins found to be increased at membranes in microgravity conditions suggests a physiological adaptation to phosphate deprivation. For instance the AtPHT1.4 phosphate transporter was over-represented in microgravity seedlings. This membrane-localized protein, preferentially expressed in roots, plays a major role in inorganic phosphate (Pi) uptake enhancement under Pi deprivation conditions [64]. Orthophosphate is required for most plant processes, being essential for development and metabolism. Higher plants use a series of adaptive morphological and biochemical strategies to increase the acquisition of poorly available Pi [65]. A common feature of the plant responses to Pi-starvation (PSR) is the up-regulation of both high-affinity phosphate transporters and purple acid phosphatases [63]. Consistent with the over-expression of AtPHT1.4 are the over-representation of two phosphatases among the 29 putative purple acid phosphatase (PAP) isozymes encoded by the Arabidopsis genome. AtPAP10 was recently characterized, and conclusive evidence proved its important role in plant tolerance to Pi limitation. This phosphatase is predominantly associated with the root surface after its secretion. AtPAP12 is phylogenetically closely related to AtPAP10, and its secretion has also been demonstrated to be Pi starvation induced. However, so far, the function of AtPAP12 remains elusive [66], [67].

The lack of a gravity stimulus has an impact in primary and lateral roots of seedlings grown under microgravity conditions see reviews [68], [69]. The protein profile observed in microgravity conditions suggests that plants adopt an adaptive strategy to respond to possible Pi-starvation that could be explained by the partial loss of contact of the roots with the culture medium. This nutrient stress can also apply for other nutrients or for water supply especially for those roots which are not in contact with the medium. Such water deficiency for some lateral roots could explain the increase of stress proteins such as osmotin-like protein (At4g11650) [70]. Other stress and defence proteins also appear to be over-represented, such as LOX1 (At1g55020) [71], [72]. LOX1 is a crucial enzyme in the oxylipin pathway but has also been shown to play a role in lateral root formation [72]. The amount of copine protein BON1 is also increased at the membrane level. Copine proteins are evolutionarily conserved proteins involved in stress defence. The Arabidopsis gene *BON1* was one of the first to be isolated on the basis of a strong resistant phenotype giving a hypersensitive response (HR) linked to strong expression of pathogenesis-related (PR) genes and resistance to virulent strains of *Pseudomonas syringae* and *Perenospora parasitica* [73]. However, it was shown later, using mutant combinations that the BON/CPN family also promotes cell growth in Arabidopsis [74]. An interesting feature is the over representation in membranes of 3 enzymes that participate in jasmonate biosynthesis [75], [76]. Indeed 4-coumarate-CoA ligase-like 7 has been shown to have a good activity with jasmonate precursors such as 12-oxo-phytodienoic acid (OPDA [77] whereas chloroplastic allene oxide cyclase2 also increased in membranes and could fuel 4-coumarate-CoA ligase-like 7 with this precursor [53]. Chloroplastic lipoxygenase 2 has also been demonstrated to participate in wound-induced jasmonate biosynthesis [54]. We can imagine that these 3 enzymes function in a microgravity situation to increase the level of jasmonate. It has also been shown that JA at higher concentrations (50 µM), reduces accumulation in the plasma membrane of the auxin efflux facilitator PIN2 [78] also involved in gravitropism [36]. This observation is also in agreement with the down regulation of proteins involved in trafficking and auxin transport discussed above. Tryptophan conjugates of JA cause agravitropic root growth and inhibit auxin in Arabidopsis [79]. It can thus be speculated that in microgravity conditions an increase of these conjugates is required to maintain the agravitropic responses encountered in space through regulation of the action of auxin.

In microgravity conditions an increase in the level of enzymes associated with lipid metabolism was also observed (Table 2). Because some of them can be involved in both synthesis and catabolism it is difficult to predict if their larger association to membranes in microgravity is associated to lipid synthesis or not. However, UDP–sulfoquinivose synthase is known to be crucial for the synthesis of sulfolipids which are components of thylakoid membranes. Such synthesis could be related to the known effect of space conditions on the ultrastructure of chloroplasts [80] and on the efficiency of the photosystems [81].

Among the membrane-associated proteins over-represented in microgravity and belonging to the oxidoreduction cluster (Table 2) there are several Cyt-P450 dependent mono-oxygenases such as the cinnamate-4 hydroxylase. This membrane-associated enzyme is 3.5-fold more abundant in microgravity than in 1 g suggesting a possible effect of microgravity on lignification since this enzyme belongs to the lignin biosynthesis pathway. Interestingly, we can also note the strong accumulation of peroxidase 34 at membranes with a >7-fold increase in microgravity compared to 1 g conditions. Although this enzyme has been clearly shown to be involved in MAMP immune responses by contributing to the generation of half the ROS produced and associated with callose deposition in plant**s** [82] we cannot exclude that this enzyme also contributes to the crosslinking of phenolic compounds and function in coordination with cinnamate-4 hydroxylase. Laccase 7, another enzyme whose level is increased at membranes, can also contribute to lignin formation through oxidative coupling of monolignols. The suggested reinforcement of the cell wall by lignification is also supported by the over-representation of UDP-arabinopyranose mutase 1 which has been shown to be involved in the biosynthesis of non-cellulosic cell wall polysaccharides [83].

Most of the enzymes classified in this oxidoreduction category can play a dual role: detoxification by conjugation such as gluthatione-S-transferase and/or defense such as cytochrome P450 CYP83B1 which can be involved in glucosinolate synthesis in response to pathogens but can also regulate auxin biosynthesis. A mutation in the *SUR_2_* gene, which encodes CYP83B1, was shown to induce accumulation of indole-3-acetic acid (IAA) in mutated plants [84]. The mutants formed higher amounts of adventitious roots and had epinastic cotyledons. The authors suggest from their results that this gene regulates the Trp–IAOx–IAN–IAA pathway in Arabidopsis reviewed in [85]. It has also been shown that the product of this gene catalyzes the first step of glucosinolate synthesis from IAOx (indole-3-acetaldoxime) [86]–[88].

The effect of gravity or microgravity on the proteome of *Arabidopsis thaliana* seedlings or cells has been studied using various experimental set up: gravitational stimulation of root apices [32], effect of simulated microgravity on callus cells [34] effect of hypergravity or simulated microgravity on callus cells [89], early effects of gravitational stimulation on inflorescence stems [90]. Only few proteins showed gravity-dependent expression or trafficking in these previous works. Many factors may explain such a discrepancy. The different experimental conditions (e.g. simulated microgravity vs space experiment) or organs under study (e.g. callus cells vs seedlings), the hardware used, but may be most importantly, the proteome which was analyzed: our analysis focused on membrane proteins while only soluble proteins were considered in the previous works. Although, soluble proteins can undergo post-translational modifications that redirect them from the cytosol to membranes or from membranes to cytosol, it is difficult to compare these various experiments. Similarly, it is also difficult to compare the results to the numerous transcriptomic data that have been collected to study the response to gravity stimulus. Such a comparison has been made by the group of Hampp who performed both approaches (transcriptomics and proteomics). They compared the results obtained from the proteomic approach performed on callus cell cultures of *Arabidopsis thaliana* exposed to 8 g (centrifugation) [89] to the results obtained from a gene expression analysis of an array containing 4100 genes hybridized with mRNAs coming from cells exposed to hypergravity [91]. Among the 200 genes shown to be significantly up-regulated after 60 min of exposure to hypergravity, only 9 genes were found to correspond to the proteins identified after 23 h of exposure to the same stimulus. These 9 proteins were associated to detoxification and metabolism.

Our global proteomic approach performed with seedlings grown in space gives a snapshot of the status of the cell and of its “on” and “off” signaling pathways. The cell activity profile obtained fits pretty well with previous observations made on specific aspects of either metabolism, signaling or organelle function such as cytoskeleton status, auxin transport, or cell wall metabolism. Overall, the comparative analysis of the proteome of microsomal fractions of Arabidopsis seedlings grown in microgravity or 1 g conditions points out that the effect of microgravity in space is strongly related to a deregulation of auxin metabolism, possibly through disturbed transport processes and membrane trafficking.

Such a model provides a perfect fit with previous observations of a lack of auxin relocalization in microgravity conditions [92], [93]. Interestingly, several proteins that are more abundant at membranes in microgravity are known to be involved in stress defence responses. To some extent, microgravity appears to be perceived by plant cells as a stress, and part of the changes observed in the membrane proteome may correspond to the setting up of an adaptative response of the cell to the stress.

## Supporting Information

Figure S1 **Frontal view of EMCS.**
(TIF)

Figure S2 **View of the various steps involved in the harvesting of seedlings.** Thawing of CCs (a), tearing up of the Biofoil (b), harvest of thawed seedlings (c), grinding seedlings in liquid nitrogen (d).
(TIF)

Figure S3 **Workflow of mass spectrometry analysis.**
(TIF)

Figure S4 **Scatterplots of the log-transformed intensities of proteins.** (a) in two control samples, and (b) in microgravity and 1 g space samples of the space experiment.
(TIF)

Figure S5 **Distributions of the log-transformed ratios, (a) between two control samples, and (b) between the 0** **g and 1** **g samples in the space experiment.**
(TIF)

Figure S6 **Q-Q plots of log-transformed ratios. (a) for a Normal distribution, and (b) for a Laplace distribution.**
(TIF)

Figure S7 **Distribution of calculated raw P-values (upper panel) or adjusted P-values (lower panel) in control and space samples.**
(TIF)

Figure S8 **Intensity ratio distribution for proteins under–represented in microgravity condition versus 1** **g space (adjusted p-value<0.05), but showing no significant difference of abundance between 1** **g space and 1** **g ground (adjusted p-values >0.05).**
(TIF)

Figure S9 **Intensity ratio distribution for proteins over–represented in microgravity condition versus 1** **g space (adjusted p-value<0.05), but showing no significant difference of abundance between 1** **g space and 1** **g ground (adjusted p-values >0.05).**
(TIF)

Table S1 **List of proteins identified with at least two peptides and quantified.**
(PDF)
