# Supplementary figures and images for: Microgravity Induces Changes in Microsome-Associated Proteins of Arabidopsis Seedlings Grown on Board the International Space Station
Source: PLoS One. 2014 Mar 11;9(3):e91814. doi: 10.1371/journal.pone.0091814 (PMC3950288; doi:10.1371/journal.pone.0091814)

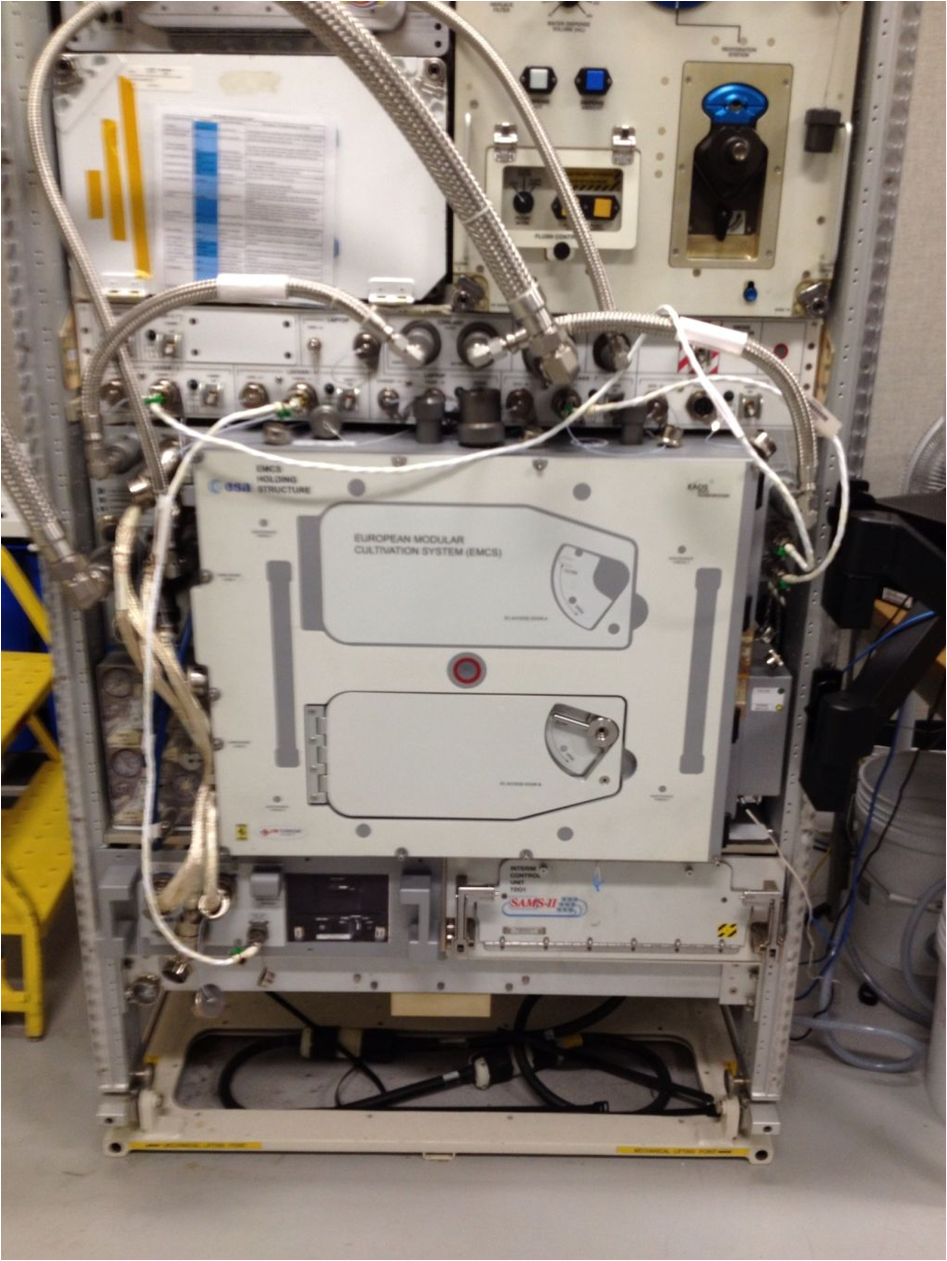

Supplement: Figure S1 — Frontal view of EMCS. (TIF) [file pone.0091814.s001.tif]

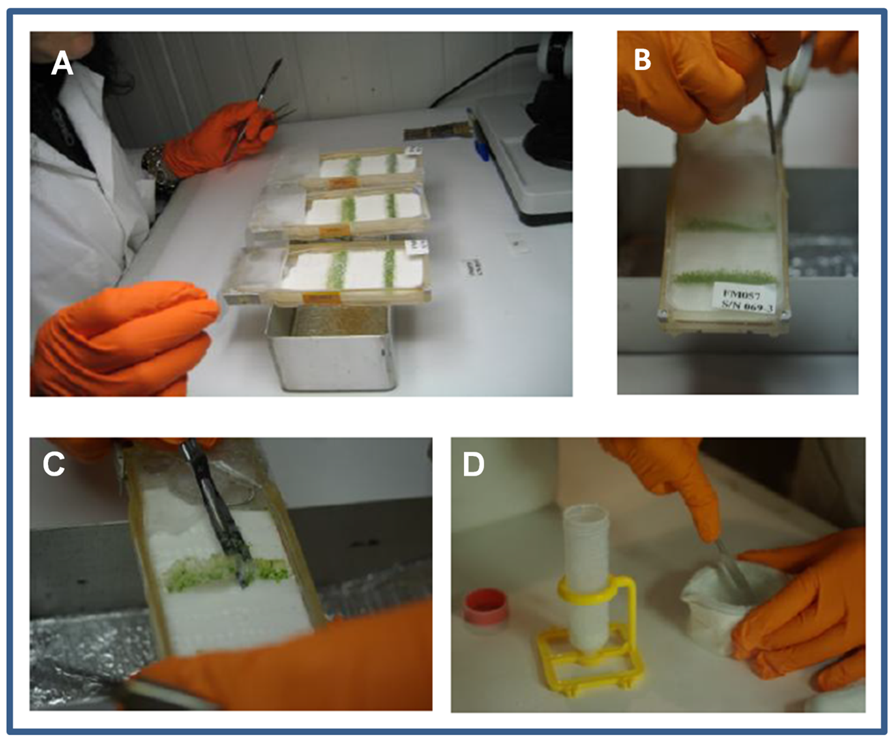

Supplement: Figure S2 — View of the various steps involved in the harvesting of seedlings. Thawing of CCs (a), tearing up of the Biofoil (b), harvest of thawed seedlings (c), grinding seedlings in liquid nitrogen (d). (TIF) [file pone.0091814.s002.tif]

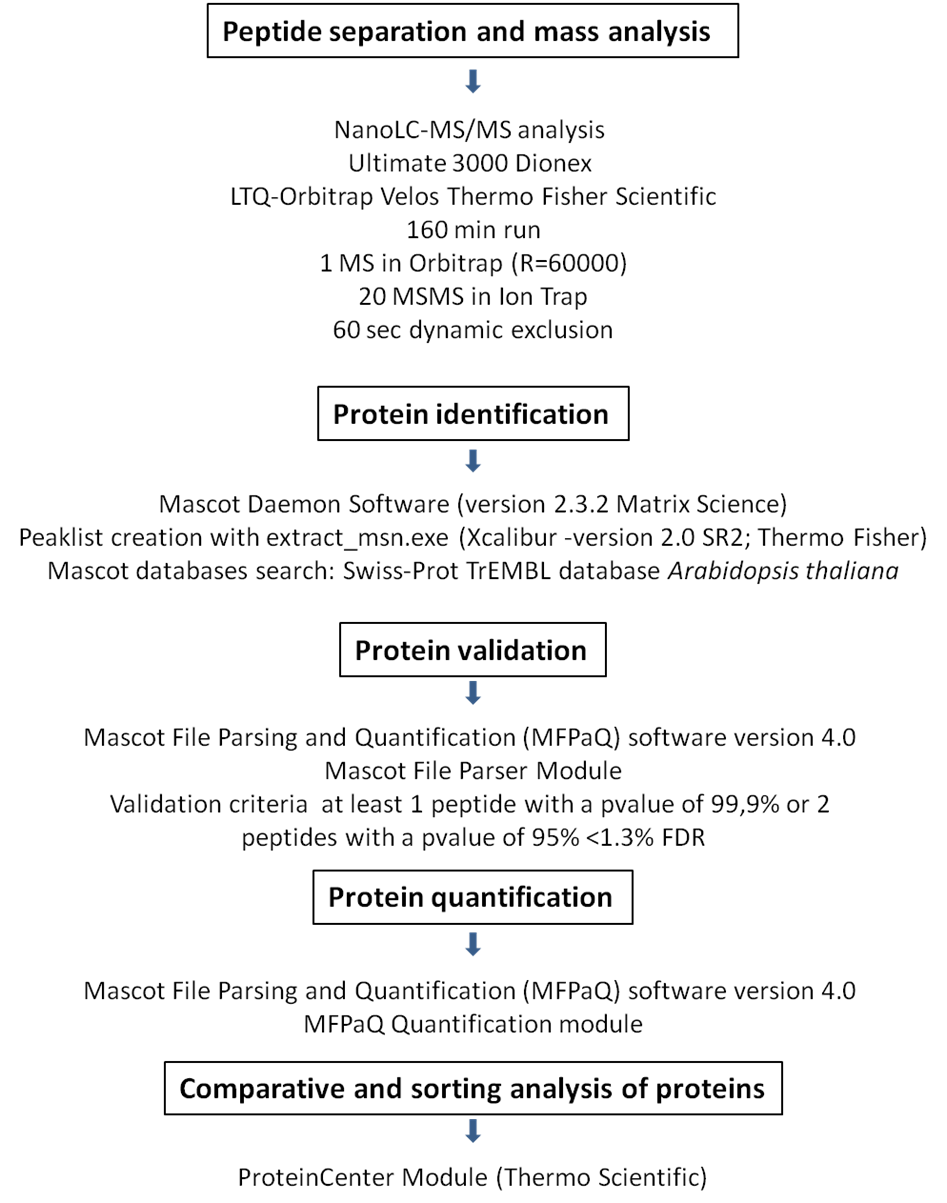

Supplement: Figure S3 — Workflow of mass spectrometry analysis. (TIF) [file pone.0091814.s003.tif]

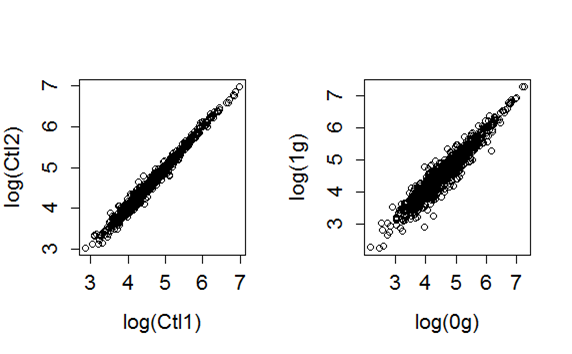

Supplement: Figure S4 — Scatterplots of the log-transformed intensities of proteins. (a) in two control samples, and (b) in microgravity and 1 g space samples of the space experiment. (TIF) [file pone.0091814.s004.tif]

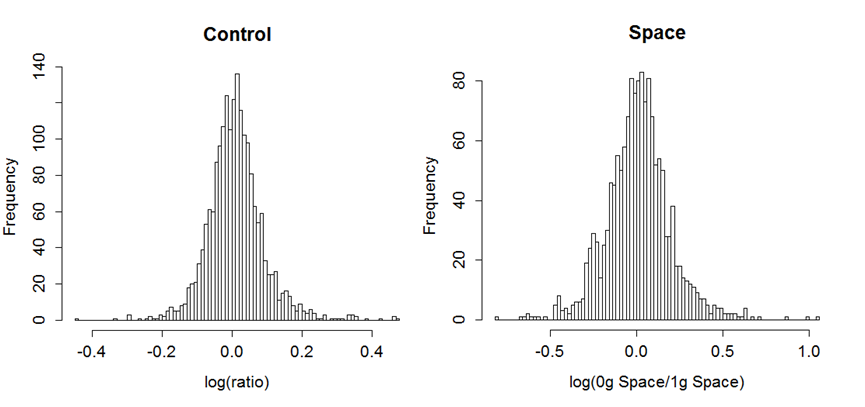

Supplement: Figure S5 — Distributions of the log-transformed ratios, (a) between two control samples, and (b) between the 0 g and 1 g samples in the space experiment. (TIF) [file pone.0091814.s005.tif]

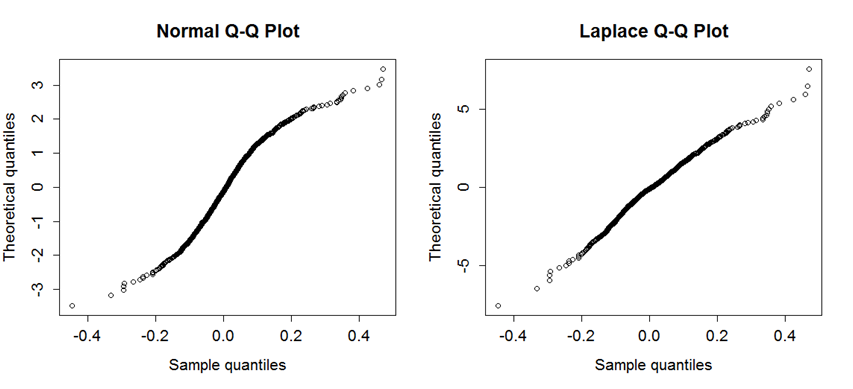

Supplement: Figure S6 — Q-Q plots of log-transformed ratios. (a) for a Normal distribution, and (b) for a Laplace distribution. (TIF) [file pone.0091814.s006.tif]

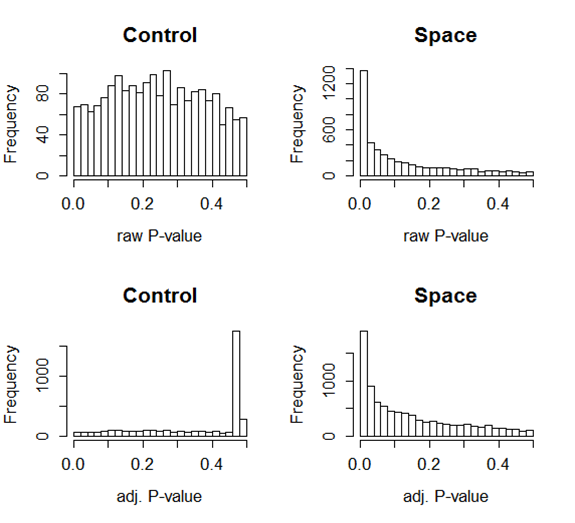

Supplement: Figure S7 — Distribution of calculated raw P-values (upper panel) or adjusted P-values (lower panel) in control and space samples. (TIF) [file pone.0091814.s007.tif]

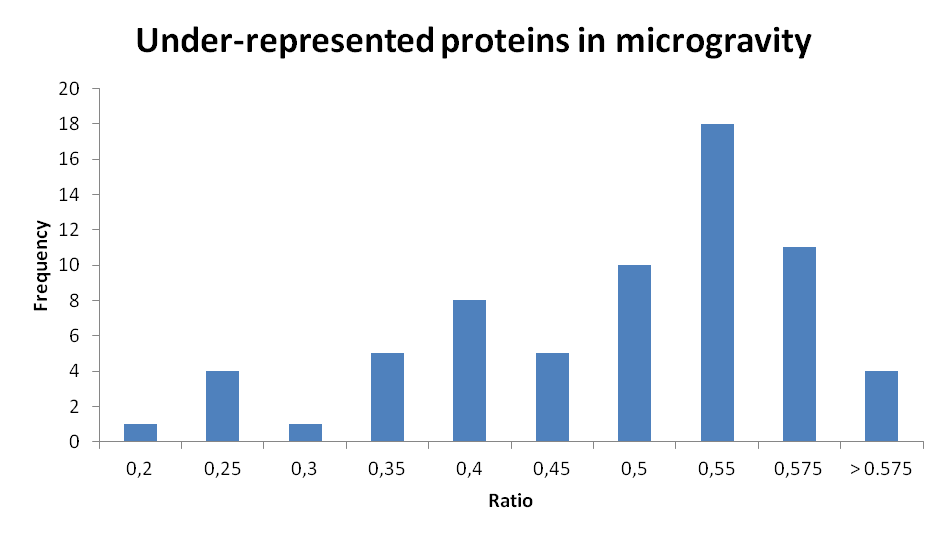

Supplement: Figure S8 — Intensity ratio distribution for proteins under–represented in microgravity condition versus 1 g space (adjusted p-value<0.05), but showing no significant difference of abundance between 1 g space and 1 g ground (adjusted p-values >0.05). (TIF) [file pone.0091814.s008.tif]

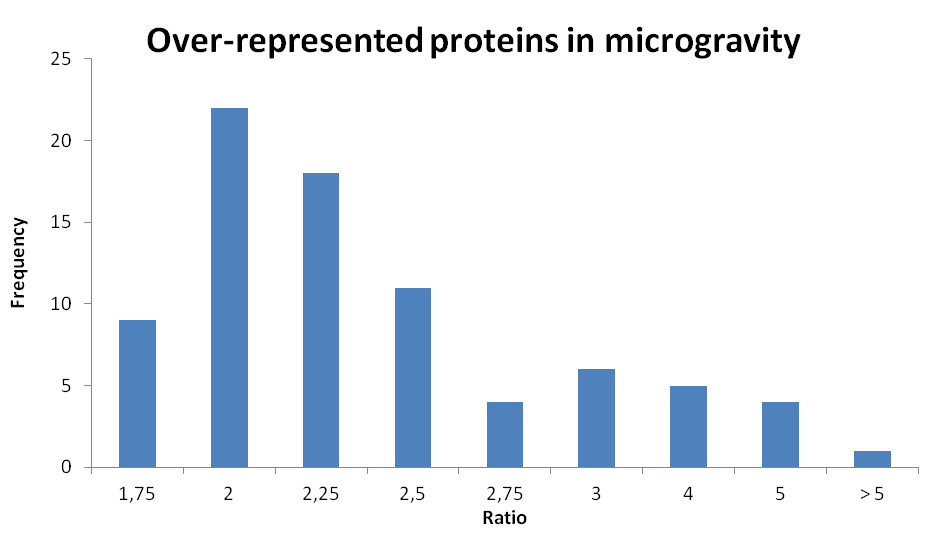

Supplement: Figure S9 — Intensity ratio distribution for proteins over–represented in microgravity condition versus 1 g space (adjusted p-value<0.05), but showing no significant difference of abundance between 1 g space and 1 g ground (adjusted p-values >0.05). (TIF) [file pone.0091814.s009.tif]
