# Supplementary material for: Microgravity Induces Changes in Microsome-Associated Proteins of Arabidopsis Seedlings Grown on Board the International Space Station
Source: PLoS One. 2014 Mar 11;9(3):e91814. doi: 10.1371/journal.pone.0091814 (PMC3950288; doi:10.1371/journal.pone.0091814)
Supplement: Table S1 — List of proteins identified with at least two peptides and quantified. (PDF) [file pone.0091814.s010.pdf]

UNIPROT	TAIR	Number of quantified peptides	PEPTIDES	DESCRIPTION	Ratio 0g/1g space	Ratio 0g/1g ground	Ratio 1g space/1g ground*	P value 0g/1g	P value 0g/1g	P value 1g space/1g ground**
Q9LRS0	AT3G14415.3	2	ILIDVNK,VPVFLDGGVR,DIQWLQTITNMPILVK,IDMATTVLGFK	(S)-2-hydroxy-acid oxidase	0,7108	0,6723	0,98226	0,12853533	0,05815837	0,30423363
P15455	AT5G44120.3	12	GQRPQEEEEEGR,CSGVSFAR,GLYLPSFFNTAK,VIPGCAETFQDSSEFQPR,IDLQTAQQLQNQDDNR,CTD	12S seed storage protein CRU4	0,71707	0,91158	1,2799	0,12549222	0,43085806	0,12852101
P42643	AT4G09000.1	1	EAAPAAAKPADEQQS	14-3-3-like protein GF14 chi	0,88536	0,60562	0,71477	0,31567926	0,05672548	0,11672133
P48347	AT1G22300.1	4	VCNDILSVIDK,AAVAAAENGLAPTHPVR,EAADQSLEAYK,QAFDDAIAELDSLNEESYK	14-3-3-like protein GF14 epsilon	1,3677	0,85347	0,67668	0,23930232	0,41661672	0,20293002
P48348	AT5G65430.1	1	AAQDVAVADLAPTHPIR	14-3-3-like protein GF14 kappa	1,05	0,83001	0,79172	0,40976466	0,21561358	0,19894185
P48349	AT5G10450.1	1	LLDSHLIPSAGASESK	14-3-3-like protein GF14 lambda	1,3355	0,98837	0,74006	0,13687527	0,46478545	0,12424607
Q96299	AT2G42590.1	1	AYEIATTAAEAK	14-3-3-like protein GF14 mu	1,1811	0,67759	0,5934	0,32110536	0,07487734	0,06697837
Q96300	AT3G02520.1	1	TVDTDELTVEER	14-3-3-like protein GF14 nu	1,02	1,3764	1,3373	0,45449884	0,09995659	0,13180052
Q01525	AT1G78300.1	3	VSAAVDGDELTVEER,GNDDHVTAIR,SAQDIANAELAPTHPIR	14-3-3-like protein GF14 omega	1,1963	0,73555	0,6319	0,29291621	0,12232474	0,08876987
P46077	AT1G35160.1	1	GNDDHVTTIR	14-3-3-like protein GF14 phi	1,0097	0,85005	0,84192	0,47480938	0,20011661	0,21523942
P42644	AT5G38480.1	2	QAFDDAIAELDTLGEESYK,SASDIATAELAPTHPIR,ICDGILNVLEAHLIPSASPAESK	14-3-3-like protein GF14 psi	1,3422	1,0344	0,80809	0,23526536	0,24920493	0,41886759
P42645	AT5G16050.1	1	ICDGILNLLEAHLIPAASLAESK	14-3-3-like protein GF14 upsilon	0,71787	0,88912	1,2386	0,11867247	0,25867952	0,17419534
Q9SZP6	AT4G38690.1	2	VICVWKPR,DNWIDTDLPSTK	1-phosphatidylinositol phosphodiesterase-related protein	0,88802	0,49177	0,55378	0,28627108	0,01421162	0,03648279
Q39227	AT1G20330.1	4	LEEVYAEIYR,ANVVGITINEYQVNR,AYVDIAETAK,ILDVGCGVGGPMR	24-methylenesterol C-methyltransferase 2	1,092	1,5556	1,4246	0,32793817	0,04919945	0,09913303
Q0WQQ6	AT5G23540.1	2	AVAVVVDPIQSVK,AGVPMEVMGLMLGEFVDEYTVR	26S proteasome non-ATPase regulatory subunit 14	0,88701	2,0787	2,3435	0,28530327	0,01280421	0,01457109
Q9SGW3	AT1G64520.1	1	FTSLPPLFENSPNAAK,IPQSPQENLILGLNLLR	26S proteasome non-ATPase regulatory subunit RPN12A	1,1152	1,3931	1,2478	0,30160883	0,12365761	0,236846
Q8W4H4		2	YVDSVVGGQIAPDNNIGR,FIADAVASLPK	26S proteasome regulatory subunit	0,85626	1,1334	1,3237	0,23819306	0,24627457	0,13309989
Q9SIV2	AT2G20580.1	2	QNLAATFVNAFVNAGFGQDK,YIPLSPILEGFIILK	26S proteasome regulatory subunit N1	0,78704	0,83217	1,1284	0,1463643	0,06266646	0,27044118
Q6EMB6	AT1G20200.1	1	LTGSVLSSFLDFALVPGSEAHSR	26S proteasome regulatory subunit N3	0,74346	1,6479	2,2165	0,13337707	0,03674982	0,01836847
Q6EMB5	AT4G24820.2	3	AFGVSVDFIDQELSR,VVDAPEILTVLGK,SEVLDFIR,IADAEENLGESEVR	26S proteasome regulatory subunit N7	1,0737	1,2771	1,2329	0,35129763	0,17947472	0,14540456
O24412	AT5G05780.1	2	AEDSKPVAIPATS,VVVHPLVLLSIVDHYNR	26S proteasome regulatory subunit N8	0,76326	1,4286	1,8717	0,1468236	0,0757436	0,03254768
Q6XJG8	AT4G28470.1	1	YIPLSPILEGFVILK	26S proteasome regulatory subunit S2 1B	0,73062	1,5469	2,1173	0,1258008	0,05048437	0,02060502
Q6XJG4		4	VEIAHIAELIGLPLDHVEK,EYVEALLSTLVK,LRPFFSLIPK,LAALLMENK	26S proteasome subunit RPN6a	3,6768	1,0236	0,96251	0,06347689	0,49686901	0,05897559
Q96291	AT3G11630.1	3	SFGVLIHDQGIALR,TLQALQYIQENPDEVCPAGWKPGEK,SGGLGDLNYPLISDVTK	2-Cys peroxiredoxin BAS1	1,0295	1,0699	1,022	0,29028546	0,18721117	0,37115731
Q9SQI8	AT3G25860.1	6	AAGMALAQHPVVNASCK,ITASDVETAAGIAPSK,LDLYLLSQK,IVYGADLAAFLQTFAK,NMIESLSVPTFR	2-oxoacid dehydrogenases acyltransferase family protein	0,82153	0,93345	1,1517	0,16183983	0,38681559	0,18121507
Q9M2T8		3	LLECGEVSQQDIDR,LAWSTQFENFLATK,NHPSTLQIYHK	2-oxoglutarate dehydrogenase, E1 subunit-like protein	1,0249	1,6447	1,6047	0,43991914	0,0369508	0,06026399
Q9SMU8	AT3G49120.1	5	NQCQFILDR,NVGLDRPSDLVALSGGHTFGK,ETIVNELR,TVSCADMLTIAAQQSVTLAGGPSWR,SALVFD	peroxidase 34	7,3136	8,6168	1,1782	9,40E-05	8,34E-06	0,22475865
Q9ASV6	AT3G15190.1	1	LIGEAYSAIDK	30S ribosomal protein S20	1,0322	0,95883	0,92894	0,4245653	0,38748556	0,34689155
Q9XJ27	AT1G74970.1	4	VPLVTLGYENSYDIFVK,EYLQGNPLWLQYVK,AHGGGLSGQAQAITLGVAR	30S ribosomal protein S9	1,0076	1,0764	1,0682	0,48028519	0,32482596	0,36005984
A9X4U2	AT2G43420.1	1	LVLYVFSLLK	3beta-hydroxysteroid-dehydrogenase/decarboxylase isoform 3	1,2878	1,3822	1,0734	0,15784004	0,08954494	0,35110449
Q9C920	AT1G53000.1	1	ALQVTPDAVFSTAVTSLKPEDGLDPNR	3-deoxy-manno-octulosonate cytidylyltransferase (CMP-KDO sy	0,6064	0,73179	1,2068	0,05897859	0,09424463	0,19895015
Q8VZB2	AT5G10480.1	1	YSFFGFK	3-hydroxyacyl-CoA dehydratase PASTICCINO 2	0,78486	0,7993	1,0184	0,16588096	0,14483867	0,45382493
Q9LKJ1	AT5G65940.1	1	TVEEIISALER	3-hydroxyisobutyryl-CoA hydrolase 1	1,1964	1,7598	1,4709	0,21738085	0,026391	0,08506375
Q9ZUZ0	AT2G46720.1	1	VWQLAFGSGFK	3-ketoacyl-CoA synthase 13	0,87368	1,0205	1,1681	0,26346102	0,44164823	0,23397099
Q56WD9	AT2G33150.1	7	GLPVLGVFR,QEQDQAAVDSHR,TGDEKPITVSVDDGIRPTTTLASLGK,DTYPDDLLAPVLR,TNLNPSEVGDI	3-ketoacyl-CoA thiolase 2	1,6412	1,9819	1,3263	0,12161525	0,00367439	0,0277367
P33207	AT1G24360.1	6	ILGTIPLGR,WGTIDVVVNNAGITR,VESPVVVITGASR,VLVNYAR,QIEEYGGQAITFGGDVSK,IINISSVVGLIG	3-oxoacyl-[acyl-carrier-protein] reductase	1,6022	1,8407	1,3201	0,22774775	0,00787023	0,0277367
P52410	AT5G46290.1	3	ALESANLGGDK,AGVLVGTGMGGLTVFSEGVQNLIEK,LLSGESGISLIDR	3-oxoacyl-[acyl-carrier-protein] synthase I	0,92834	0,61783	0,73544	0,172787	0,03690382	0,14132648
Q9SIE3	AT2G22230.1	2	FPAFPTVMDINQIR,VIEYTPGVSAVAIK	3R-hydroxymyristoyl ACP dehydrase	1,7672	3,0398	1,7202	0,04574243	0,00249169	0,04547361
Q9LX13	AT5G10160.1	3	YEAFPTVMDINK,SNFFFAGIDK,VIEYTAGVSAVAIK	3R-hydroxymyristoyl ACP dehydrase	1,6295	2,1996	1,3498	0,06099168	0,01030389	0,12443244
P16181	AT3G48930.1	2	FNVLK,ILAGTCHSAK	40S ribosomal protein S11-1	1,5438	1,4938	1,108	0,26346102	0,23380176	0,1374723
P42733	AT5G23740.1	1	EAIDGAYIDSK	40S ribosomal protein S11-3	1,1209	2,0109	1,794	0,29291621	0,0148253	0,03772105
Q9S9P1	AT1G15930.2	2	VAQLVVLAEDCNQPDYVK,DFGEETTALSIVNK	40S ribosomal protein S12-1	1,2622	1,6658	1,3513	0,22213442	0,01738877	0,0555293
Q9SKZ3	AT2G32060.3	3	VVGCSCLVIK,NAQLCVLAEDCNQPDYVK,DFGEETTALNIVK	40S ribosomal protein S12-2	0,6544	1,2999	1,9864	0,08067319	0,12167154	0,0266429
P59224	AT4G00100.1	2	GLTPSQIGVILR,LILVESR	40S ribosomal protein S13-2	0,80416	0,76148	1,0026	0,10907499	0,10861875	0,35101078
Q9SIH0	AT2G36160.1	1	VDVVTLGPSVR	40S ribosomal protein S14-1	0,8316	1,1061	1,3301	0,21328864	0,2808519	0,13050741
Q9CAX6	AT3G11510.1	2	IEDVTPIPTDSTR,VETVTLGPSVR,TPGPGAQSALR	40S ribosomal protein S14-2	0,95331	1,1762	1,2453	0,30754911	0,32903377	0,21316325
P42036	AT3G52580.1	1	TPGPGAQSALR,VENVTLGPAVR	40S ribosomal protein S14-3	0,84551	1,0769	1,3699	0,2218635	0,38756356	0,17419534
Q2V4R0	AT1G04270.2	1	EAPQGEKPEPVR	40S ribosomal protein S15-1	0,9208	1,2783	1,4258	0,29409355	0,1307783	0,09745137
Q9FY64	AT5G09510.1	1	EAPAGEKPEPVR	40S ribosomal protein S15-4	0,74316	1,1065	1,4889	0,13337707	0,28048336	0,08149266
P42798	AT5G59850.1	2	HGYIGEFEYVDDHR,ISVLNDALK,EIEGWTAR	40S ribosomal protein S15a-1	1,1343	1,6244	1,6343	0,45831573	0,09593216	0,11267335
Q9SK22	AT2G09990.1	1	IFEPILLGK	40S ribosomal protein S16-1	1,096	1,2967	1,1878	0,31685402	0,30839936	0,20968388
Q42340	AT5G18380.1	1	IFEPVLLGK	40S ribosomal protein S16-3	1,0172	0,95563	0,93951	0,45776795	0,38074277	0,36664065
Q9SQZ1	AT3G10610.1	1	ILEEVAIIPSK	40S ribosomal protein S17-3	1,332	1,8846	1,4149	0,13889133	0,01992393	0,10264234
Q5PNZ9	AT4G09800.1	4	YSQVVSNALDMK,IPDWFLNR,IMFALTSIK,AGELSAAEIDNLMTIVANPR,VLNTNVDGK	40S ribosomal protein S18	0,88166	1,1122	1,3068	0,20935831	0,26037722	0,12230716
Q9FNP8	AT5G61170.1	1	IELPLWTDIVK	40S ribosomal protein S19-3	0,83479	0,93133	1,1142	0,21322366	0,31921491	0,2908149
Q8H189		1	VIDLFSSPDVVK	40S ribosomal protein S20	0,76081	1,2576	1,6529	0,14551713	0,14137613	0,05407754
P49200	AT5G62300.2	1	VIDLFSSPDVVK	40S ribosomal protein S20-1	0,9563	0,78714	0,82311	0,39968787	0,13401989	0,191671
P49688	AT2G41840.1	3	TYGFLTPEFWK,LSVVPVR,VLQFAGIDDVFTSSR,SPYQEHTDFLASK,QIEQIYLHSLPVK,EYQIIDMLIGPTLK	40S ribosomal protein S2-3	0,9914	1,2171	1,2993	0,32843915	0,14192721	0,11725341
P49201	AT5G02960.1	2	IGIEAK,VSGVSLLALFK	40S ribosomal protein S23-2	1,0781	1,0067	0,96229	0,3847187	0,36216877	0,49515082
Q9SS17	AT3G04920.1	2	SSGFGLIYDTVESAK,DPNAIFVFK	40S ribosomal protein S24-1	0,93525	0,98567	1,0582	0,29576483	0,45807104	0,30776669
P49206	AT2G40590.1	3	GHVNPIR,NIVEQAAIR,DVQEASVYEGYTLPK	40S ribosomal protein S26-1	0,89045	0,81803	1,0308	0,09196159	0,13190576	0,235938
Q9SR73	AT5G03850.1	1	EGDILTLLESER	40S ribosomal protein S28-1	1,1971	0,89559	0,74811	0,21721359	0,26763162	0,12852101
Q9SIP7	AT2G31610.1	1	TPLPDVVIIHAPK	40S ribosomal protein S3-1	0,85458	1,5526	1,8168	0,23630779	0,04945274	0,03615671
Q9M339	AT3G53870.1	2	EYIDSAVR,VMLDWDPK	40S ribosomal protein S3-2	0,85906	1,6148	1,8797	0,24130467	0,04112409	0,03225315
Q9CAV0	AT3G04840.1	2	LFCIAFTK,TTDSYTLR	40S ribosomal protein S3a-1	1,1535	1,886	1,7186	0,21902323	0,0184179	0,05965669
Q42262	AT4G34670.1	1	TTDGYTLR	40S ribosomal protein S3a-2	1,1393	1,6991	1,5223	0,19262079	0,01878338	0,06924373
P49204	AT5G07090.1	5		40S ribosomal protein S4-2	1,0824	1,4237	1,34	0,29525989	0,30557396	0,18997493
O48549	AT4G31700.1	1	ISQEVSGDALGEEFK	40S ribosomal protein S6-1	0,89676	0,94823	1,162	0,10212603	0,20834825	0,17307293
P51430	AT5G10360.1	1	LSQEVSGDALGEEFK	40S ribosomal protein S6-2	0,89676	0,94823	1,162	0,10212603	0,20834825	0,17307293
Q9M885	AT3G02560.2	2	AVVIYVPFR,GVAPTEFEEQVTQALFDLENTNQELK	40S ribosomal protein S7-2	0,92662	0,78566	0,85919	0,36636313	0,24563713	0,36723385
Q93VG5	AT5G20290.1	7	VLDVVYNASNNELVR,DGEEGEEAAVAAPEEVK,ADGYILEGK,SLDSHIEDQFASGR,LDTGNYSWGSEATTR,	40S ribosomal protein S8-1	1,0484	1,3184	1,2655	0,34220693	0,026391	0,05756286
Q9LXG1	AT5G15200.1	2	VQYSLSR,YGLLDESQNK,IFEGEALLR	40S ribosomal protein S9-1	1,2712	1,5036	1,2458	0,33405095	0,07196559	0,1311499
Q08682	AT1G72370.1	6	LLILTDPR,FAQYTGANAIAGR,EGALGNIPIIAFCDTDSPMR,HSIGCLFWLLAR,FVDIGIPANNK,MMCAAEV	40S ribosomal protein Sa-1	0,97172	1,2942	1,358	0,3146579	0,02479861	0,02760656
Q9LF93		1	VFNQDLYFK	pyruvate kinase-like protein	0,1573	0,21505	1,3672	0,00019966	0,00033184	0,11842417
Q9LTH1		1	VYVIGEEGILK	4-nitrophenylphosphatase-like	1,0202	0,62512	0,61273	0,45095774	0,0428636	0,05683749
Q9LY66	AT3G63490.1	8	GTGQTVIVAVLAQGEK,TGIVHIPFGK,VNFTEEDLLINFLAAVK,ATVSLPK,YNDQQLR,AGTVTANIPQAIEEF	50S ribosomal protein L1	1,1981	0,82755	0,81057	0,33906565	0,17392991	0,13880363
Q9MAP3	AT1G32990.1	4	AGYIIPVEITVFDDK,TPPASVLLLK,SFTFILK,VGVITIDQLR	50S ribosomal protein L11	1,1379	0,768	0,69949	0,38648576	0,15860329	0,14531065
P36210	AT3G27830.1	2	ILVDYLQDK,IGSEISSLTLEEAR	50S ribosomal protein L12-1	1,1772	0,86642	0,73602	0,23075357	0,22292779	0,12198653
P36212	AT3G27850.1	2	ILVDYLQDK,IGSEISSLTLEEAR	50S ribosomal protein L12-3	1,2426	0,81294	0,64248	0,20230136	0,04179326	0,0277367
Q9SYL9	AT1G78630.1	3	GPDHPHEAQKPLDLPIR,IVEHAVR,LASTIANHIR	50S ribosomal protein L13	0,81505	0,85112	1,0209	0,21790356	0,24573956	0,38172228
Q9SX68	AT1G48350.1	1	QKPISEEFDYTSGPTIEVAK	50S ribosomal protein L18	1,1803	1,1115	0,94173	0,22774775	0,2742757	0,36998444
Q3EDH2	AT1G07320.2	1	TLNLFDILNADK	50S ribosomal protein L4	0,62132	0,41474	0,67826	0,0523846	0,00938781	0,12993994
O04603	AT4G01310.1	4	LLALMGMPFR,IVVNCGIGDAAQNDK,DQGVFPEIR,DIALITGQKPIK,EGGGGSTGAIVR	50S ribosomal protein L5	1,0115	0,75443	0,76592	0,37591169	0,34596681	0,4888616

O50008	AT5G17920.2	12	IPSSEEIADR,WFDTNYHYIVPELGPEVNFSYASHK,ALGVDTVPVLVGPVSYLLLSK,SFELLSLLPK,NIWANDF	5-methyltetrahydropteroyltriglutamate--homocysteine methyltr	1,0596	1,4192	1,3204	0,42619086	0,26070743	0,2423675
Q42112	AT3G09200.1	4	VGLVAPIDVVVQPGNTGLDPSQTSFFQVLNIPTK,IHSENTGNTAILNLLPLLQGNVGLIFTK,NALAIAVATEYT	60S acidic ribosomal protein P0-2	1,033	0,94602	0,9145	0,25045	0,37566743	0,30784206
P57691	AT3G11250.1	3	NALAIAVATDYTFPQAEK,IHSENSGNTAILNLLPLLQGNVGLIFTK,FVVAAAAVSADAGGGSAQAGAAAK	60S acidic ribosomal protein P0-3	0,96868	1,314	1,5368	0,11399376	0,14912661	0,04132934
Q8LEQ0	AT5G47700.2	2	AAGVTIESYWPMLFAK,NVTDLIMNVGAGGGGGAPVAAAAPAAGGGAAAAAPAAEEK	60S acidic ribosomal protein P1-3	1,9187	2,83	2,2499	0,05945217	0,012231	0,15355836
P51407	AT2G27720.1	3	LASVPSGGGGGVAVASATSGGGGGGGAPAAESK,VVAAFLLAVLSGK	60S acidic ribosomal protein P2-1	1,1251	1,431	1,4325	0,2962133	0,03851025	0,08836791
B9DGN3	AT2G27710.3	3	TILGSVGAETEDSQIELLLK,LASVPSGGGGGVAVASATSGGGGGGGASAAESK,VVAAYLLAVLSGK	60S acidic ribosomal protein P2-2	1,4365	2,1477	1,8225	0,24031994	0,01057178	0,03254768
B3H4N7	AT5G57290.3	3	GVFSFVCK,QHEGDLEASASSTYDLQR	60S acidic ribosomal protein P3-2	1,2599	1,7919	1,5467	0,2523643	0,02366639	0,0680739
Q93VT9	AT1G14320.1	2	ENVSSEALEAAR,DAHGHHAQEALR,VAIGQVLLSVR	60S ribosomal protein L10-1	1,2162	1,5361	1,339	0,09380015	0,06538476	0,48313394
Q8VZB9	AT1G08360.1	1	EAITTITGK,NFVETIELQIGLK	60S ribosomal protein L10a-1	1,0014	1,1865	1,5769	0,16588096	0,18098215	0,07439308
P59230	AT2G27530.2	3	FPTLVSHQESLEAK,NFVETVELQIGLK	60S ribosomal protein L10a-2	1,332	1,8984	1,791	0,42810099	0,01649625	0,02474635
Q0WQZ1	AT2G42740.1	4	VLEQLSGQTPVFSK,AMQLLESGLK,YEGVILNK,IACYVTVR	60S ribosomal protein L11-1	1,0878	1,4658	1,3652	0,30758306	0,20850578	0,14531065
Q0WW72	AT3G53430.1	4	EILGTCVSVGCTVDGK,HNGNISFDDVTEIAR,VTVVPSAAALVIK	60S ribosomal protein L12-2	1,0104	1,4276	1,5101	0,42004279	0,17988632	0,2413593
P41127	AT3G49010.3	4	AGDSTPEELANATQVQGDYLPIVR,SLEGLQTNVQR,LAPTIGIAVDHR	60S ribosomal protein L13-1	0,72696	1,0416	1,4076	0,15727102	0,12693655	0,05337672
Q9FF90	AT5G23900.1	1	SLEGLQSNVQR	60S ribosomal protein L13-3	0,7629	1,3176	1,7271	0,1468236	0,11312893	0,04475628
Q9LRX8	AT3G24830.1	2	VFEGVPPPYDK,ELLNGQSVVVVR	60S ribosomal protein L13a-2	0,79247	1,5157	1,8789	0,17788236	0,06756386	0,03539219
Q9T043	AT4G27090.1	3	ANLNDFDR,LSLTDIVIDINR,LVVIVDVVDQNR	60S ribosomal protein L14-2	0,80169	1,2159	1,602	0,12978731	0,2413764	0,07124351
Q8VYF1	AT4G17390.1	2	GIVYGKPTNQGVTQLK,YYEIILVDPAHNAVR	60S ribosomal protein L15-2	0,95126	2,0374	2,0569	0,33667925	0,05089621	0,04997613
Q93VI3	AT1G27400.1	2	YSQEPDNITK,SAQFVLDLLK	60S ribosomal protein L17-1	1,077	1,1787	1,1553	0,49571374	0,21781433	0,23990571
Q0WRE5	AT1G67430.1	1	SAQFVLDLLK	60S ribosomal protein L17-2	0,677	0,89836	1,4102	0,06347689	0,33540753	0,07814145
P42791	AT3G05590.1	1	IAVLVGTITDDLR,HFGPAPGVPHSHSKPYVR	60S ribosomal protein L18-2	1,111	1,3849	1,3785	0,40976466	0,08227413	0,08836791
Q940B0	AT5G27850.1	3	IAVLVGTITDDLR,HFGPAPGVPHSNTKPYVR,APLGQNTVLLR,APLSLSR,SNSNFNAVILK	60S ribosomal protein L18-3	1,1043	1,5313	1,4169	0,49925283	0,12988833	0,15236678
P51418	AT2G34480.1	2	FWYFLR,LWATNEVR	60S ribosomal protein L18a-2	0,85851	1,4713	1,6102	0,48554397	0,02420318	0,03633011
Q9SRX2	AT1G02780.1	2	GPGGDVAPVAAPAPAATPAPTAAVPK	60S ribosomal protein L19-1	0,51946	0,9298	1,7899	0,03506043	0,32630692	0,03801615
Q0WSS0	AT1G57860.1	2	VGDYVDVK,GYIPLSTYLR	60S ribosomal protein L21-2	0,74089	1,0413	1,4055	0,13264411	0,38984503	0,10547876
Q9M9W1	AT3G05560.3	1	AGALGDSVTITR	60S ribosomal protein L22-2	0,57848	0,89212	1,5422	0,04823352	0,26260914	0,07155399
Q8LD46	AT2G39460.2	3	LTPDYDALDVANK,VNTLIRPDGTK,LDHYQILK	60S ribosomal protein L23a-1	0,88828	1,4961	1,6737	0,29525989	0,05151081	0,04504245
Q94F49	AT5G27670.1	1	LLHGVTIASGGVLPNINPVLLPK	histone H2A 7	0,21652	0,23759	1,0973	0,00119159	0,0005183	0,31412586
P51414	AT3G49910.1	5	VNGTTVNVGIQPSK,VVQVYR,VIMSSPLSTDLR,AHFTASSSER,DDEVQIVR	60S ribosomal protein L26-1	1,1595	2,228	1,9508	0,24242069	0,06237995	0,15355836
P51419	AT4G15000.1	1	EVATLDALQSK	60S ribosomal protein L27-3	1,0671	1,2207	1,2172	0,41311163	0,0746239	0,08183355
Q9LR33	AT1G23290.1	2	FFCPIVNLDK,LWSLVPEDVK	60S ribosomal protein L27a-2	0,89221	1,0814	1,2825	0,21876223	0,31921491	0,14661987
P49637	AT1G70600.1	1	LWSLVPEDVK	60S ribosomal protein L27a-3	1,0565	1,5135	1,4326	0,3814661	0,05630528	0,09745137
Q0WSA0	AT1G77940.1	1	LILISTNCPPLR	60S ribosomal protein L30-2	1,0738	1,5146	1,4704	0,45504834	0,03843823	0,06026399
Q9LSA3	AT3G18740.1	1	YNGNNVDLGTACGK,LILISSNCPPLR	60S ribosomal protein L30-3	0,99749	1,6172	1,6983	0,48362139	0,07912739	0,10547876
P17094	AT1G43170.9	2	VDFAYSFFEK,GIQAQLEK	60S ribosomal protein L3-1	1,5427	1,4803	0,95951	0,07772805	0,0631033	0,40701926
O80626	AT2G39390.1	1	SIAQVLTVISQK	60S ribosomal protein L35-2	1,1753	1,4314	1,2432	0,24220458	0,07226174	0,17307293
Q9FZH0	AT1G41880.1	1	SNQYPNTSLIQIEGVNTQEEVNWYK	60S ribosomal protein L35a-2	1,0194	1,6883	1,6562	0,45267591	0,03224572	0,05362229
Q9M352	AT3G53740.4	1	EVAGQAPYEK	60S ribosomal protein L36-2	0,90412	1,8892	2,0896	0,31054298	0,01973783	0,02145069
Q8LEM8	AT3G16080.1	1	CSACAYPAAR	60S ribosomal protein L37-3	1,8453	1,3789	0,74724	0,0390965	0,09056879	0,12852101
Q8RXU5	AT3G60245.1	2	YFCEFCGK,AGGAYTMNTASAVTVR	60S ribosomal protein L37a-2	1,0411	1,0213	0,98094	0,40832398	0,43978357	0,45165109
O22860	AT3G59540.1	1	YLYTLCVFDQEK	60S ribosomal protein L38	1,0669	1,2062	1,1306	0,36464091	0,17467063	0,27044118
Q9SF40	AT3G09630.1	1	IINSDEIQSVVNPIK	60S ribosomal protein L4-1	0,45316	0,71534	1,5786	0,02012287	0,08425049	0,06536678
P49691	AT5G02870.1	1	IINSDEVQSVVNPIK	60S ribosomal protein L4-2	0,62445	0,74342	1,2813	0,03241734	0,13279871	0,07040057
Q8LBI1	AT3G25520.1	3	SNAYFK,ALLDVGLIR,DIVAQIVSASIAGDIVK	60S ribosomal protein L5-1	0,88884	1,0555	1,2087	0,19367066	0,4125894	0,21268144
P49227	AT5G39740.2	2	DIVAQIVSASIAGDIVK	60S ribosomal protein L5-2	1,213	1,0465	0,86272	0,20541826	0,38074277	0,2413593
Q9FZ76	AT1G18540.1	1	QLSSGLLLVTGPFK	60S ribosomal protein L6-1	1,1814	1,2841	1,0869	0,22702952	0,12836822	0,32998295
Q9C9C6	AT1G74060.1	3	QLASGLLLVTGPFK,VDAPVEKPPK,VDISGVTLDK	60S ribosomal protein L6-2	1,0917	0,97028	0,88875	0,32814661	0,4146112	0,27726892
Q9C9C5	AT1G74050.1	1	QLASGLLLVTGPFK	60S ribosomal protein L6-3	0,84318	1,5699	1,8619	0,22317613	0,04687855	0,03345105
P60040	AT2G01250.1	2	IALTDNSIVEQALGK,HGIICTEDLIHEILTVGPHFK	60S ribosomal protein L7-2	0,95362	1,4775	1,7717	0,14287184	0,0428636	0,02071524
Q3EBG6	AT2G44120.2	1	IALTDNSIVDQALGK	60S ribosomal protein L7-3	1,178	1,4388	1,3823	0,43206157	0,09311739	0,12974288
P49692	AT2G47610.1	2	VSNPLFER,LGAVVHQK,TASCLCLTTVK	60S ribosomal protein L7a-1	0,79152	1,5622	1,9819	0,13996266	0,04398296	0,02060502
Q9LZH9	AT3G62870.1	1	TAAALCLTTVK	60S ribosomal protein L7a-2	1,1102	1,6379	1,5806	0,4368568	0,02481085	0,03543749
P46286	AT2G18020.1	3	AMIGQVAGGGR,SIPEGAVVCNVEHHVGDR,ASGDYAIVIAHNPDSDTTR	60S ribosomal protein L8-1	1,0071	1,2807	1,3741	0,28627108	0,0615253	0,04995052
P49209	AT1G33140.1	6	VIEVEGPR,HLNLDFQLIK,IDSWFGTR,FVYAHFPINASIGGDGK,TALSHVDNLISGVTR,SCALINQK	60S ribosomal protein L9-1	0,984	1,3176	1,4452	0,18877558	0,42312681	0,20220305
Q9C5J7	AT5G56630.1	1	VAVVGIPK	6-phosphofructokinase 7	1,3041	2,7363	2,0981	0,14974463	0,00354507	0,02145069
Q9FWA3	AT3G02360.2	1	DSGPCVTYIGK,AGSPVDQTIK	6-phosphogluconate dehydrogenase	0,87767	1,3445	1,5372	0,27014962	0,26404347	0,15390372
A8MS34	AT1G50430.2	1	IVASYTTTSGETK	7-dehydrocholesterol reductase	0,94106	1,2976	1,323	0,42165346	0,16898384	0,16356589
Q9SRY5	AT1G02920.1	1	FTLVDLHTIPVIQYLLGTPTK	glutathione S-transferase 11	4,6163	7,3786	1,5984	0,00119159	2,40E-05	0,0615125
Q8LPQ6	AT4G25450.1	4	IIELGTHSELVAQK,VGELTGLLTSDLGALNSIVNDNISR,NAPILILDEATSALDAVSER,LVQSALNR,STIVQLLA	ABC transporter B family member 28	1,0177	1,5783	1,4602	0,44615942	0,41028807	0,37921453
O23482	AT4G16370.1	2	ISTVHALSFLADLK,ILTPQFDLDIGAYNNYGK	Oligopeptide transporter 3	0,29141	0,55966	1,7647	0,00119159	0,00271908	0,07476504
Q9C8G9	AT1G30400.2	4	LEEVLSTEER,LASLAENSLNSVER,VLGIIPQAPVLFSGTVR,ILIDECDIGR	ABC transporter C family member 1	1,2903	1,9655	1,5977	0,36944641	0,02064468	0,04132934
Q9SKX0		1	SSILNALFR	ABC transporter C family member 13	0,99957	1,9426	1,9074	0,46447374	0,0179601	0,03119634
Q94FB9	AT4G39850.1	1	DLLALVATVVFR	ABC transporter D family member 1	1,5884	1,061	0,99434	0,06099168	0,29797719	0,03801615
Q9FJH6	AT5G60790.1	3	LPPPVLQFVEVSFGYTPDYLIYK,AAEILFGLGFDK,VIFAWLAYK	ABC transporter F family member 1	1,3866	1,6754	1,2083	0,11932159	0,03350051	0,19779495
Q7PC88	AT2G29940.1	1	LNILK	ABC transporter G family member 31	0,69493	1,0034	1,4439	0,10545331	0,48948178	0,09313267
Q7PC87	AT2G36380.1	1	LVEYFEAIEGVPK	ABC transporter G family member 34	1,2242	0,89492	0,731	0,19718068	0,2670029	0,11838808
Q9XIE2	AT1G59870.1	13	SLPTLLNVVR,YEHLTIK,NSLVTDYTLK,VQETFAR,DISGVIKPGR,NAFFYVFK,LSVDFAELYNQSALHQR,MSI	ABC transporter G family member 36	1,2522	1,2868	1,0413	0,13264411	0,13401989	0,36854968
Q7PC84	AT1G66950.1	1	IQILK	ABC transporter G family member 39	1,2219	1,4094	1,1781	0,14224503	0,09018468	0,40507275
A0NAA8	AT2G01320.2	3	QFFLLLK,TTLLNVLAGQLSLSPR	ABC transporter G family member 7	0,91247	1,3754	1,5073	0,32194046	0,09131721	0,07809191
Q8W496	AT4G25650.2	1	LSLIFICIPVSPGR	ACD1-like protein	1,0537	1,1736	1,1139	0,3847187	0,20271416	0,2908149
Q38971	AT1G36160.2	2	DLFEGILQAGSTIVENLR	acetyl-CoA carboxylase 1	0,74916	0,89041	1,2052	0,15424716	0,27514969	0,23559881
Q2HIV2	AT3G25760.1	2	ALKPSGVVSNFTN,GLANDLPLELIGTPVPPSK,FGLGDLVPFTNK	allene oxide cyclase 1	3,8866	4,6524	1,3975	0,00119159	0,00033184	0,36345423
P56765		2	ISSVLCDYQSSK,GLLDAIVPR	Acetyl-coenzyme A carboxylase carboxyl transferase subunit bet	0,68371	0,71569	1,0372	0,10907499	0,15491052	0,24459912
Q9FJE8	AT5G59870.1	2	HLLLAIR,LLSGVTIAHGGVLPNINSVLLPK	histone H2A 6	0,23441	0,27302	1,1647	0,00119159	0,00104436	0,23682267
Q9SIB9	AT2G05710.1	1	SGEDADTLGLTGHER	aconitate hydratase 2	1,6289	2,7985	1,718	0,06099168	0,00325483	0,04559256
Q3E6W5	AT2G31810.2	1	VEDISSEPQVER	ACT domain-containing small subunit of acetolactate synthase p	1,1183	1,155	1,0328	0,29525989	0,22191953	0,42536905
Q9SRQ7	AT3G03530.1	5	LPNYVVVEQR,SNTVSSSDTNSLR,LLLEGFPQK,QDCPETLSTPIK,LYVHSATSHGATSNDK	phospholipase C	3,2845	2,0092	0,60677	0,00119159	0,00431771	0,05963097
O23140	AT5G46630.1	2	PVAASAIYFLNLR,SGYNTVEWVR,TNFQVTTGR	AP-2 complex subunit mu-1	0,59061	0,73883	1,2873	0,00121527	0,0022783	0,13614308
O22832	AT2G43710.1	1	DYADILEFLVGR	acyl-[acyl-carrier-protein] desaturase	1,503	1,9059	1,3012	0,07123949	0,03462055	0,37626434
Q9M879	AT3G02630.1	1	DYADILEFLVER	acyl-[acyl-carrier-protein] desaturase	1,0607	0,93867	0,88497	0,37451204	0,34358374	0,27044118
Q9LSQ0	AT3G16910.1	1	IPANYTALTPLWFLDR	acyl-activating enzyme 7	1,0574	1,4107	1,6053	0,11940032	0,0985513	0,03254768
Q8LDU8		1	AQSIATVEQAAALIEELLLEK	acyl-carrier protein ACP, putative	1,1481	1,8719	1,6305	0,25951247	0,02052223	0,05707974
O04619	AT4G01100.1	7	SLFAGGVAGGVSR,STASLEYTGMVDAFR,VVPSIAIAFVTYEMVK,ENPYGLVENNELTVVTR,TESAAVSTIV	adenine nucleotide transporter 1	1,3642	2,3245	1,7039	0,12801913	0,00779529	0,04671641
Q9SUW2	AT4G22570.1	1	GFLFGPPIALAIGAK	adenine phosphoribosyl transferase 3	1,0563	1,175	1,0918	0,20847942	0,24125082	0,36871976
P31166	AT1G27450.1	3	GFIFGPPIALAIGAK,AIIIDDLIATGGTLAAAIR	adenine phosphoribosyltransferase 1	1,0815	0,8969	0,86846	0,14224503	0,07231508	0,32512179

B3LFB3	AT3G09820.1	1	AGCYASNVVIQR	adenosine kinase 1	1,3425	0,84806	0,6317	0,13416284	0,19802596	0,06464314
O82514	AT5G63400.1	5	AVLTNIQAEK,APQEVTSEVK,TPLGVK,TPGVDDITGEPLIQR,LAAFHSQTQPVIDYYAK,VLNFAIDDAILEER	adenylate kinase 1	1,2245	1,3169	1,161	0,14901598	0,24705869	0,08325311
Q2V2W1	AT5G63400.2	1	VLNFAIDDAILEER	adenylate kinase 1	0,9878	0,93108	0,94258	0,46816001	0,32795148	0,37098283
Q8VYL1	AT5G35170.1	4	AEVSSGTDIGK,YVLLATYAERPTPEQIDDAFSGK,LVTRPDDTEEK,RPVVLINPR	adenylate kinase family protein	1,0224	0,82805	0,80987	0,44591736	0,17392991	0,17667834
Q0WVD8	AT3G08580.2	5	TDGIAGLYR,GFNISCVGIIVYR,GFTNFALDFLMGGVSAAVSK,QFDGLVDVYR	ADP,ATP carrier protein 1	1,1053	1,2739	1,1802	0,18646308	0,11452519	0,37086758
Q0WW68	AT1G80300.1	2	LLTTLGPR,IFGVEVATLK	ADP,ATP carrier protein 1	0,96836	0,87294	0,91374	0,44298898	0,15233262	0,19551153
P40941	AT5G13490.2	3	LLIQNQDEMLK,GFNISCAGIIVYR,SDGIAGLYR	ADP,ATP carrier protein 2	1,0562	3,4331	3,5799	0,42165346	0,05301688	0,08303118
P92935	AT1G15500.1	3	IFGVEVTTLK,LLATLGPR,IPVVSQEDAPSGETTSQLSEK	ADP,ATP carrier protein 2	0,54633	0,81468	1,4912	0,04040215	0,15959709	0,08075935
Q9LQC8	AT1G70490.3	6	MLNEDELR,ILMVGLDAAGK,LGEIVTTIPTIGFNVETVEYK,QDLPNAMNAAEITDK,NISFTVWDVGGQDK,D	ADP-ribosylation factor 2	0,69804	0,89463	1,2816	0,10766085	0,26660992	0,15212336
Q6ID97	AT1G10630.1	5	MLNEDELR,ILMVGLDAAGK,LGEIVTTIPTIGFNVETVEYK,QDLPNAMNAAEITDK,NISFTVWDVGGQDK,D	ADP-ribosylation factor A1F	1,128	1,1416	1,0277	0,274315	0,28375281	0,43420114
Q8VY57	AT3G49870.1	1	AVSAIVYVVDAADPDNLSVSK	ADP-ribosylation factor-like A1C	0,84779	0,61578	0,72634	0,22830814	0,03966131	0,11535213
Q06327	AT1G55020.1	1	LPITSSLDIYVPR	lipoxygenase 1	4,1369	3,0705	0,74223	0,00121527	0,00243954	0,12540988
Q2V412	AT2G41100.3	1	LTDDQITEYR	calmodulin-like protein 12	3,679	3,6934	0,99799	0,00240024	0,00134791	0,39452092
Q56YU0	AT3G24503.1	1	EAGIPDGVLNIVTGFGSTAGAAIASHMDVDK	aldehyde dehydrogenase 2C4	1,9349	3,6348	1,8786	0,03424101	0,0010594	0,03225315
Q70E96	AT4G36250.1	5	LPLLFYPAK,SFFGENPK,TATVAINCLDK,CPTIVDHHTISK,IIMAAAAQHLTPVTLELGGK	aldehyde dehydrogenase 3F1	1,1082	1,1628	1,0718	0,39151124	0,19802596	0,27971321
Q70DU8	AT1G44170.2	5	LLEQYLDPSAVR,DDLGKPELESSVYEVSLLR,SPVVVDSDTDLK,ALVDSNIFDLFK,VVEGAVTETSALLEQK	aldehyde dehydrogenase 3H1	1,7896	2,1368	1,1566	0,08337039	0,0448421	0,40070282
Q9STT3	AT3G47800.1	4	YGGLCLETQGFPDSVNHK,IHELPGGYDINYVIDGPIGK,GFSDVIWSVQK,LIPTGEITSITGTPYDFLEPR	aldose 1-epimerase	0,80321	0,63953	0,7888	0,09929699	0,0194921	0,14396051
Q940G5	AT4G25900.1	2	VITFNAQLDR,FVTVESAAVAKPITVNPGK	aldose 1-epimerase family protein	1,1906	2,0017	1,6813	0,22069077	0,01516721	0,04946517
B1GV36		1	NEIDTVLGPGVQVTEPDLHK	cinnamate-4-hydroxylase	3,5288	2,5677	0,72763	0,00287954	0,00489045	0,11591242
Q9LJX0	AT3G28860.1	1	NPTVLLLDEATSALDAESECVLQEALER	ABC transporter B family member 19	0,29547	0,34809	1,1781	0,00342053	0,00296739	0,22475865
Q9SCX3	AT5G19510.1	4	GVEMPGLFWGASK,VYAAVPVKPSDAFPNASK,SVEEHLAGK,AVTFSDLHTEEGVK	Elongation factor 1-beta 2	3,4476	3,6971	1,1344	0,00444978	0,00096352	0,247568
Q9LS01	AT3G25780.1	1	IQELNVYELNEGDR	allene oxide cyclase 3	1,1347	1,2649	1,1147	0,2749585	0,13666172	0,29002757
Q96242	AT5G42650.1	20	SNGGELTMGAIEK,DFVVLVAR,NLLFFLLK,SVVYECLR,ADFGGSSDGTAFNFLAR,LFVIEIFR,AFYGTNPADT	allene oxide synthase	0,87966	3,4924	3,3957	0,48920473	0,01120702	0,02006469
Q9SJI7	AT2G42690.1	1	NWDTILDPLDQSLR	alpha/beta-hydrolase domain-containing protein	2,4595	1,2936	0,52678	0,01523118	0,14483867	0,03184562
Q8VZ57	AT1G52510.1	2	IPLFGEFTCQNAILAER,LAILNSPLTVSSPVPGLFK	alpha/beta-hydrolase domain-containing protein	1,1143	1,386	1,2221	0,31578971	0,13614272	0,24929874
Q93Z70	AT2G19940.2	1	AIIISVIDNLVK	alpha/beta-hydrolase domain-containing protein	1,0157	1,3948	1,3732	0,4602126	0,08520734	0,11641753
A4IJ41	AT3G10840.1	1	FLGNAFGGSQQQDVDLK	alpha/beta-hydrolase domain-containing protein	0,9476	1,0905	1,1508	0,38323589	0,30327825	0,24929874
Q9FFW9	AT5G38520.1	3	LLPWLAQLSST,LIPEITKPVLVLWGDQDGLTPLDGPVGK,GIASALFNR	alpha/beta-hydrolase-like protein	1,4072	1,5043	1,069	0,11445402	0,05810662	0,35838427
Q9SGH6	AT3G01420.1	15	FLLLIVHGVDK,LPVFLGLTYLEVR,RPDPMVVATK,IHTIDWTVQLLK,GLEWVNTTESLK,LVTSAVVAK,TPW	alpha-dioxygenase	1,636	0,95316	0,6217	0,0385024	0,2804106	0,02339742
Q8VXZ7	AT3G56310.1	1	EIIAVNQDPLGVQGR	alpha-galactosidase	1,2146	1,1024	0,90761	0,20402177	0,28570603	0,30776669
Q9LS02	AT3G25770.1	2	GLANDLPLELTGTPVPPSK,VQELSVYEINELDR,NAFSLMFGLGDLVPFTNK	allene oxide cyclase 2	2,961	2,5132	0,9533	0,00514795	0,00976953	0,10264234
Q9SPE6	AT3G56190.1	12	AVNIFCEIGR,VDTNEAASCLER,HSLNNNLLK,YQDLDPTFTGTR,ADVVSITNALEK,FLADLASAIDEEDIAK,H	alpha-soluble NSF attachment protein 2	1,0243	0,96785	1,0614	0,28236567	0,24575719	0,48940992
Q9S7Y7	AT1G68560.1	12	EYDAHSIYGFSETIATHK,QELYQWDTVADSAR,DFTLNPVAYPR,IPLDVIWNDDDHMDGHK,QCPSGEGPG	alpha-xylosidase 1	1,0517	0,72992	0,71877	0,39110214	0,27850583	0,38080967
Q2V367	AT5G19140.2	2	DEIFCLFQGSLDNLGSLK	aluminum induced protein with YGL and LRDR motifs	1,1191	1,4104	1,2602	0,29497091	0,08050429	0,16012956
Q41931	AT1G62380.1	2	DSEYPSFVFDDYMK,HLPQSNLNDISDVSDEYR	aminocyclopropanecarboxylate oxidase	1,2031	1,047	0,87032	0,21322366	0,37990607	0,25155844
O65396	AT1G11860.3	11	TGYTGEDGFEISVPDEHAVDLAK,GGAIDDSVITK,VTDEHIYLVVNAGCR,SLLALQGPLAAPVLQHLTK,VGFF	aminomethyltransferase	1,1299	1,0562	0,97581	0,31684455	0,32358376	0,4497903
P38418	AT3G45140.1	13	GYITAQEEFLEGITWSR,GLDDIADIR,YGGEFYVPR,LPGGSITFTCESWVAPK,LTIPDYPFANDGLILWDAIK,A	lipoxygenase 2	2,8506	3,2054	1,1384	0,00594029	0,00335257	0,31160325
Q9SYT0	AT1G35720.1	12	STIQCLTRPELYFVDVLR,VSDSVPAPSDDAEQLR,TAFEGWGTNEDLIISILAHR,AILLWTLEPGER,TSTQLLHA	annexin D1	1,9527	0,99534	0,52273	0,03514917	0,30101402	0,04132934
Q9XEE2	AT5G65020.1	14	VPSNVPLPEDDAEQLHK,YEGDDVNMMLAR,SVYAATYNEDLLK,ELSSDFER,LLLPLVSTFR,AFSGWGTNEK	annexin D2	2,4095	1,2226	0,50782	0,01446762	0,0980089	0,04033278
Q9SE45	AT2G38760.1	6	VPNEVPSPAQDSETLK,LLVTLASTFR,VAIFCIDTPEK,DLIDVLSSELSGDFMK,DSIEGFGTDEDSLTR,DFIITLL	annexin D3	1,5622	0,67963	0,48706	0,32110536	0,04938623	0,04671641
Q9ZVJ6	AT2G38750.1	3	HFNEIK,LLVGLVSAYR,DFLLTLLSK,SSLLNEALICLLKPALYFSK	annexin D4	0,81904	0,68008	0,88517	0,36464091	0,09018468	0,14531065
Q9LD43	AT2G38040.2	1	IEALEQQIK	acetyl-coenzyme A carboxylase carboxyl transferase subunit alpl	0,33612	0,39591	1,1779	0,00630893	0,00536658	0,22481978
Q8L7A9	AT1G31730.1	2	DLVSSFVSILK,LSAVESYLQLISEPK	AP-4 complex subunit epsilon	0,75327	0,94089	1,2491	0,14059531	0,34770623	0,16725321
Q94IE2		7	IVEGDAEDLPFPTDYADR,EEEYIEWFK,FFSDVWMLFPK,YVSAGSIEYWPDPQR,VVDVGGGTGFTTLGIVK,	APG1	1,1483	1,1557	1,0154	0,20274946	0,16604258	0,49362608
P61837	AT3G61430.2	3	QYQALGGGANTVAHGYTK,EPPPAPFFEPGELSSWSFWR	aquaporin PIP1-1	1,1359	0,60508	0,51733	0,41328858	0,00354507	0,00652833
A8MRW1	AT2G45960.3	3	QYQALGGGANTIAHGYTK,EPPPAPLFEPGELASWSFWR,SLGAAIIFNK	aquaporin PIP1-2	0,90336	0,48445	0,63273	0,06713121	0,00667631	0,07809191
B9DFR9	AT2G45960.2	1	QYQALGGGANTIAHGYTK	aquaporin PIP1-2	0,53146	0,75493	1,4205	0,0374089	0,11058961	0,100525
Q08733	AT1G01620.1	3	QPIGTSAQTDK,EPPPAPFFEPGELSSWSFYR	aquaporin PIP1-3	0,84199	0,25775	0,30612	0,2218635	0,00078311	0,00351289
P43286	AT3G53420.2	3	DVEAVPGEGFQTR,DYQDPPPAPFIDGAELK,ALLYIIAQCLGAICGVGFVK	aquaporin PIP2-1	0,71276	0,6127	0,95468	0,08351316	0,01007099	0,08876987
P43287	AT2G37170.1	3	AFQSSYYDR,DVEGPEGFQTR,DYEDPPPTPFFDADELTK	aquaporin PIP2-2	1,8601	0,82066	0,43324	0,35207156	0,00488204	0,01392798
C0SVL5	AT4G35100.2	2	DYVDPPPAPLLDMGELK,DSHIPVLAPLPIGFAVFMVHLATIPITGTGINPAR	aquaporin PIP2-7	0,62361	0,4622	0,74117	0,06440019	0,01107518	0,12465876
P25818	AT2G36830.1	2	AALAEFISTLIFVVAGSGSGMAFNK,NIAIGRPDEATRPDALK	aquaporin TIP1-1	0,75368	0,44179	0,55934	0,21902323	0,04119178	0,12396969
Q41963	AT3G26520.1	2	NIAIGGVQEEVYHPNALR,AALAEFISTLIFVFAGSGSGIAFNK	aquaporin TIP1-2	0,58958	0,41026	0,69996	0,16741981	0,02878188	0,12164339
Q41951	AT3G16240.1	3	GSLGTIAPLAIGLIVGANILAAGPFSGGSMNPAR,AGVAFGSFDDSFSLASLR	aquaporin TIP2-1	0,80974	0,37563	0,4378	0,35896271	0,00296739	0,00839164
Q56WK6	AT1G72150.1	11	IDELVESGEEVSEFEK,EGHVVIYSSYGEFQNK,SADVAAAPVVK,AIDFSNPEAK,QFEDNYPEFAAK,IVITIDNG	patellin-1	0,58578	0,40104	1,7006	0,00630893	0,00931231	0,13614308
Q9SZX3	AT4G24830.1	2	GVYETPGGTILFAAVQELESLTLDR,ALSPATLLAELNTIGGK	argininosuccinate synthase	0,71952	1,1589	1,5595	0,11207126	0,43888698	0,09169192
O23247	AT4G26300.1	1	VIFLEGFDIPLMVVK	Arginyl-tRNA synthetase, class Ic	0,71966	0,69052	0,95951	0,11932159	0,07113495	0,40701926
Q93VS8	AT5G19820.1	6	EGFFPWIDQVAPTLVPLLK,ILIVFAEVLTGK,LNVVIQLPNAR,IAALNAVISFVQCLANSTER	armadillo/beta-catenin-like repeat-containing protein	0,61712	0,91799	1,5415	0,09843317	0,17420063	0,19256419
Q9FJD4	AT5G53480.1	3	QFQEQNLAGFLLSLAGELANDEKPVDSR,SVKPPIFSCFGDIALAIGEDFDK,QALPGLVPLLLETLLK	armadillo/beta-catenin-like repeat-containing protein	0,56849	0,71742	1,262	0,04635602	0,08538335	0,15909481
Q9FX95	AT1G49670.1	4	IIYAGVNASDVNFSSGR,FHQGLSFPSAIFVK,LPFDAGFEGVGLIAAVGESVK	ARP protein	1,23	1,9185	1,5597	0,19251025	0,01843986	0,06904298
O04947		2	LPSSPPPVTPR,LILLNTQNLIDGYTK	ascorbate oxidase	1,4347	2,2293	1,5538	0,10667054	0,00968629	0,06932445
P82281	AT4G09010.1	12	QLAVMSAFLGPDQAATEQLLATDPQVAPWVQK,FTYLASAIR,SDATEADPEGR,FIAVGLGPR,ETVSQTDYE	ascorbate peroxidase 4	1,4606	1,3908	1,0285	0,13462476	0,0791647	0,38151565
P46644	AT5G11520.1	2	IFTLAGLTVK,LILGADSPAIR	aspartate aminotransferase	1,7898	1,3783	0,77004	0,04306948	0,09060668	0,14369822
Q94AF6	AT5G20160.1	1	AYPLADSQLSITILDLVQQATNYK	U4/U6 small nuclear ribonucleoprotein SNU13	2,9457	4,2155	1,4311	0,00630893	0,0005183	0,09745137
P46248	AT4G31990.2	1	ISLAGLSLAK	aspartate aminotransferase	1,6086	1,0878	0,67628	0,06347689	0,30606248	0,08325311
Q38934		5	AAPVWYNMLK,FTVVFDTGSSNLWVPSSK,TVVDQYGQTILDLLLSETQPK,CYFSLACLLHPK,FDGILGLGFQ	aspartic proteinase	1,2215	1,4964	1,236	0,32141383	0,09223695	0,16545095
Q9LI73	AT3G25700.1	1	LFFTPLLTNPLSPTFYYVK	aspartyl protease family protein	1,1651	0,86832	0,7453	0,24063742	0,22601448	0,12730146
Q9M2U7	AT3G54400.1	2	GPLSLISQSQNLYQSTFSYCLPNSK,AIVQSPTYIVR	aspartyl protease family protein	0,86992	0,79575	0,91474	0,25804393	0,14149413	0,32009677
O04496	AT1G09750.1	1	QNANAVLNVIANLQQQNLR	aspartyl protease-like protein	1,1658	0,96012	0,85155	0,23050855	0,39651228	0,18571584
Q9M356	AT3G61820.1	3	VAYDLVGSR,LDDSSECVTR,LTQPAYVALR	aspartyl protease-like protein	0,95196	0,90592	0,95163	0,39110214	0,28375281	0,38863231
Q9ZVS5	AT1G03230.1	4	ISSVNPYTVLESSIYK,IPNLIFSCGSTSLLK,TPLLINPGTTVFEFSK,EFWVDCDQGYVSTTYR	aspartyl protease-like protein	0,73461	0,93552	1,2821	0,0390965	0,0779512	0,14132648
Q8VZF8		1	GGDNQSEVISK	At1g02120/T7I23_26	0,80275	0,7667	0,95509	0,18321838	0,11959875	0,39714947
B0FV99		1	EAPQGEKPEPVR	At1g04270	0,49762	2,1827	4,3862	0,02976225	0,01057178	0,00054729
Q94KD4		1	LLTSASDDVR	At1g09270/T12M4_2	0,74608	0,81671	1,0947	0,13472408	0,16177995	0,31762474
B9DGT0		4	IVSFLDPDGWK,IVNQELGGK,GGGSVIAFVK,DPDGYTFELIQR	AT1G11840	1,7168	1,2688	0,7629	0,0753558	0,099675	0,25156677
B9DGZ4		7	SDLDIVSNASCTTNCLAPLAK,AASFNIIPSSTGAAK,FGIVEGLMTTVHSITATQK,YDSVHGQWK,TLLFGEKPV	AT1G13440	1,1737	0,72218	0,57326	0,18621086	0,07578276	0,03974861
Q9FPI2		1	STIGSPPDELDEISAGK	At1g17620	1,1345	0,87716	0,77315	0,27510854	0,2397083	0,14531065
Q94BW0		1	AAQDIANAELAPTHPIR	At1g35160/T32G9_30	1,3163	0,70317	0,53419	0,14457138	0,077212	0,03254768
Q9LIL4	AT3G22845.1	1	EALESVVAEQK	emp24/gp25L/p24 family/GOLD domain-containing protein	2,9425	3,0082	1,0223	0,00630893	0,00259189	0,44596687
B9DGR7		2	YLQEVLFLNNK,LPLNLGSITALYLTFANNR	AT1G49750	0,78032	0,75619	0,96908	0,16183983	0,11129187	0,42700519
Q5XF82		16	VIGPIHGASGR,VGSCIDGIGVYYAPLPPSPPPPEK,VIGSETGVITMLR,TYDPIGYNEGAR,GFDDGGDYDGIR,I	At1g52100	1,0073	1,3084	1,2784	0,3719914	0,36369854	0,48185096
C0Z2H8		1	CSACAYPAAR	AT1G52300	1,1028	1,2807	1,1613	0,31436427	0,12988833	0,23990571
Q94F41		1	ELLEELASLK	At1g80030/F18B13_37	1,2341	1,2747	1,0329	0,18917409	0,13221115	0,42536905
B9DFH2		1	GVPDFWLIALK	AT2G19480	1,4258	0,90402	0,63404	0,10942681	0,2808519	0,06562021

C0Z3G4		1	VVAAYLLAVLSGK	AT2G27710	1,4248	1,2534	0,8909	0,18321838	0,24306048	0,31493804
B9DHR0		3	ETIGGDEFR,GVLLIGPPGTGK,SLVAYHEVGHAVCGTLTPGHDAVQK,LVEVLLEK,VPSSTTTTPASAPTPAAV	AT2G30950	1,8849	1,5537	1,0508	0,07214592	0,07412095	0,32052402
C0Z300		1	ALFTQVTTK	AT2G37660	1,1086	1,0335	0,93225	0,30758306	0,40910075	0,35218597
Q8VZG8	AT4G08850.1	1	LLTNLEYLDLSSNR	Leucine-rich repeat-containing protein	2,9398	2,4104	0,81992	0,00630893	0,00647983	0,18816785
Q9ASV4		1	FVEFLTR	AT3g06720/F3E22_14	0,84143	1,2699	1,5092	0,2218635	0,13401989	0,07799316
C0Z3A0		2	FGSPLLNLPEVAIIALGR	AT3G06850	1,0003	1,7936	1,7931	0,49925283	0,02441009	0,03772105
B9DHP6		1	CTGLLLAR	AT3G25520	0,91659	1,587	1,7137	0,42165346	0,02438436	0,03345105
B9DGQ8		1	FEQEISSR	AT3G27570	1,3881	2,3239	1,6742	0,11932159	0,00779529	0,05008108
C0Z391		1	LGALGCTFPEILSK	AT3G27690	0,84556	0,7938	0,88106	0,4029344	0,28402752	0,37737857
B9DHN8		8	VIVADLK,AGNAVIGNGIDDVLK,LINHENAEVTK,SSFVQADGVK,GVPIAISCLSSLLK,LFHDESLANEDTYEPF	AT3G42050	0,92624	1,696	2,2553	0,45396265	0,13669608	0,17307293
Q94C38		5	VIVADLK,VVILTFR,LINHENAEVTK,SSFVQADGVK,ENVTCFEENDFQILR	AT3g42050/F4M19_10	0,75129	0,76181	1,014	0,13922032	0,11536542	0,46466908
Q93ZL3		3	QQFFLCSASLQDIISR,FSPWEIVR,QLMNILGVVYR	AT3g46970/F13I12_20	0,94847	1,3805	1,4555	0,3847187	0,09018468	0,08876987
Q93ZE7		7	GDVTVPELIFEFK,TPGLTYTPFR,GPVSLPSQMNLK,YLCSGCDFSGLDPTLIPR,AFLEYYYLNLR,LDFPDLTVP	AT3g52500/F22O6_120	1,4817	1,0342	0,698	0,09361953	0,4075447	0,09745137
Q8LPR0		4	ESSLPVSVFEFTMSNLGK,FVLQVYR,LVAGDEEEESNLLLR,GFQTASGIYEAIWSDR	At4g10060/T5L19_190	1,3403	0,92062	0,68687	0,13472408	0,30839936	0,08876987
B9DHH1		2	GDLAVTVGR,GLISEIISR,LIGATDPQK	AT4G11010	1,3014	2,1383	1,6698	0,16168813	0,01183842	0,05337672
Q9FPJ3		3	ISATSIYFESLPYK,LLICGGSAYPR,NAVFGDSSALAPGGVR	AT4g13930	1,0025	0,82247	0,82858	0,42303608	0,27470657	0,34816126
Q84WJ3		1	VIYIDDLADWIK	At4g29690	0,36739	0,74161	2,0186	0,0076488	0,10051964	0,02511127
Q94JQ3		1	DFVVVADFIK	At4g32520/F8B4_220	0,92906	1,6982	1,8279	0,35150194	0,03113571	0,0355902
B9DI06		1	ATDLNQGVVYGVK	AT4G33030	1,0586	1,3011	1,2291	0,3767835	0,12137488	0,18070565
Q8L891		1	VLLLVWDDK	AT4g35790/F4B14_60	0,48326	1,2553	2,5976	0,02590559	0,14196313	0,00995155
Q9FL43		4	VPTITFMFK,LAKPVYEAVR,VKPTTAVVTSLGGFDTCYSGQVK,LGPTSQPQR	At5g07030	1,066	0,78262	0,75725	0,37451204	0,09725565	0,15236678
Q9LQQ4	AT1G07790.1	1	TAAERPVEENK	histone H2B	0,34323	0,37037	1,0791	0,00641506	0,00367439	0,34243189
Q8L749		1	VLGVLGIVYSAAQYVISR	AT5g13390/T22N19_40	0,54039	1,0955	2,0273	0,0390965	0,29593287	0,02474635
Q93ZK3		5	IPSSEEIADR,ALGVDTVPVLVGPVSYLLLSK,SFELLSLLPK,NIWANDFAASLSTLQALEGIVGK	AT5g17920/MPI7_60	0,57071	0,70011	1,2171	0,04274392	0,05863512	0,20220305
B9DGU9		1	GLSLEEIESK	AT5G59250	1,2938	1,129	0,87262	0,15508788	0,25211857	0,25387857
Q8LCP3		1	DVIQLALDLAR	ATMRK1	0,76016	0,48804	0,64202	0,14481507	0,01377556	0,06904298
P56758	ATCG00150.1	2	IIQLPQGELAAPTNDINTTVALALLTSVAYFYAGLSK,NPQTIPTDGQNFFEFVLEFIR,YIQPTPILLPINILEDFTK	ATP synthase CF0 A subunit	0,6612	1,531	2,3164	0,08503666	0,10185261	0,02474635
P56759	ATCG00130.1	6	ILNTIR,EGAIQQLENAR,GVLNDLLDNR,NETILFEQQR,VNGYSEIER,VFQQALQGAIGTLNSCLSNELHLR	ATP synthase CF0 B subunit	1,199	1,1682	1,0045	0,2218635	0,04787149	0,13576487
P56757	ATCG00120.1	15	EVTIVNTGTVLQVGDGIAR,IAQIPVSEAYLGR,TLTAEAESFLK,SVYEPLQTGLIAIDSMIPIGR,FLVQLR,QSQS	ATP synthase CF1 alpha subunit	0,99271	1,2348	1,2009	0,49417787	0,13388272	0,1545229
P19366	ATCG00480.1	30	VGLTALTMAEYFR,GFNLILSGEFDSLPEQAFYLVGNIDEATAK,ELQDIIAILGLDELSEEDR,EGNDLYMEMK,	ATP synthase CF1 beta subunit	1,007	1,3243	1,259	0,36165493	0,07071045	0,12344714
P09468	ATCG00470.1	2	IGNNEITILVNDAEK,NSDIDPQEAQQTLEIAEANLR	ATP synthase CF1 epsilon subunit	1,1258	1,3959	1,1987	0,15102512	0,02701153	0,12724546
Q9SUI9		11	VALVVVTGDR,RPYIPVDK,YLEAGTLPTAK,FVSLVK,ALQESLASELAAR,GEICDINGTCVDAAEDEFFR,GNS	ATP synthase gamma chain, chloroplast precursor	0,99889	1,2343	1,1685	0,30758306	0,09424463	0,17803691
A7KNI5		1	LATLTNAISSR,ESLVVLR	ATP synthase protein MI25	1,5346	2,8419	1,8519	0,21818685	0	0
P93303	ATMG00480.	3	AVDLLGK,NQLLSHR,ITLISCFGEISGSR,DPNSLEDLLR	ATP synthase protein YMF19	0,93219	2,1891	2,7259	0,23747711	0,02479861	0,07421815
P60112		1	LIGAGAATIALAGAAIGIGNVFSSLIHSVAR	ATP synthase subunit 9, mitochondrial	0,39106	2,7759	7,0984	0,0106699	0,00335257	3,20E-05
P92549		3	AILNSVKPELLQALK,MELDAFLK	ATP synthase subunit alpha, mitochondrial	0,86321	1,6196	1,8151	0,27014962	0,04476232	0,03640683
P83484	AT5G08690.1	18	YDDLSEQSFYMVGGIDEVVAK,EGNDLYR,TEHYLPIHR,QISELGIYPAVDPLDSTSR,VLNTGAPITVPVGR,IP	ATP synthase subunit beta-2	1,143	2,5589	2,2387	0,26501876	0,004953	0,01740826
Q9C5A9	AT5G08680.1	14	YDDLSEQSFYMVGGIDEVVAK,TEHYLPIHR,QISELGIYPAVDPLDSTSR,IPSAVGYQPTLASDLGALQER,VLN	ATP synthase subunit beta-3	0,90886	1,3444	1,4753	0,22421051	0,1140068	0,06904298
Q9FT52	AT3G52300.1	5	EAYDSIEIPK,FSQEPEPIDWDYYR,AFDEVNTQLQTK,EIADVQEISK,FDALLVELK	ATP synthase subunit d	1,0596	1,3528	1,2758	0,18663468	0,04520429	0,15355836
Q96252	AT5G47030.1	3	LASATTDLEK,LTVNFVLPYTSELTGK,GLAEFQQK	ATP synthase subunit delta'	0,96184	1,5641	1,6288	0,40683029	0,03931545	0,04767472
Q96251	AT5G13450.1	11	IDPSIYGGLIVEFQQK,DPSVPR,ELTETLQEIIGAGK,NFLSLLAENGK,VLDMSIR,IETDLSEMIEAMK,VLVTTV	ATP synthase subunit O	0,94862	1,07	1,129	0,44832611	0,23372411	0,28053259
Q3ZVE1		1	TAVATDTIINQQGQNVICVYVAIGQK	ATPase alpha subunit	1,1015	1,1591	1,0523	0,31540618	0,21714644	0,38556112
Q9C522	AT3G06650.1	2	EVTPPQIPEDLSSAIK,DLVSSLVSGLLTIGPR	ATP-citrate lyase B-1	1,3284	1,0635	0,86347	0,38437408	0,40708656	0,45165109
Q9FI56	AT5G50920.1	5	VPEPTVDETIQILK,VVQILGR,VITLDMGLLVAGTK,YTDESLVAAAQLSYQYISDR,IIGQDEAVK,EIADILLK,M	ATP-dependent Clp protease ATP-binding subunit ClpC	0,73126	0,96222	1,2916	0,08941395	0,088429	0,34243189
P56772		1	SPGEGDTSWVDIYNR	ATP-dependent Clp protease proteolytic subunit 1	0,66695	0,62144	0,93176	0,08819529	0,04179326	0,35110449
Q94B60	AT5G45390.1	2	ADVSTIALGIAASTASIILGAGTK	ATP-dependent Clp protease proteolytic subunit 4	1,1317	0,98692	0,93	0,14408762	0,05865764	0,26061733
Q9S834	AT1G02560.1	1	FQSIISQLFQYR	ATP-dependent Clp protease, protease subunit	0,81046	0,86129	0,99857	0,11940032	0,03089351	0,17890785
Q94BT2		1	LAFDFWNLR	Auxin-induced in root cultures protein 12	0,40817	1,4602	3,6468	0,01347706	0,08709742	0,00279604
Q9LVA0	AT5G62390.1	4	SMVDELEAMLDVVDPQPQGK,LDLIDPYTCTPLIVR,SYNWTTEVK,ENGEVSHTYIIK	BCL-2-associated athanogene 7	0,58528	0,83615	1,4286	0,04967535	0,18306382	0,09812982
Q94CE4	AT1G70410.2	3	ADLGNVAAAK,HSGVGAAVEYAVVHLK,ELDSSNSDAIER,VCPSHILNFQPGEAFVVR,NSTLFNHLAK	beta carbonic anhydrase 4	0,92683	0,65818	0,68336	0,21184587	0,01050728	0,03632066
Q9SE50	AT1G52400.3	10	YYIQR,GSTDYVGMNYYTSVFAK,DLNTDAFR,SPSWTTDSLVDWDSK,HWITFNEPWVFSR,VLDFILGWHL	beta glucosidase 18	0,76993	0,95244	1,2492	0,29525989	0,31597334	0,19615166
Q9FIW4	AT5G36890.1	1	FGLVYVDYK	beta glucosidase 42	1,0067	0,79263	0,79116	0,47480938	0,14294724	0,15782318
Q9M649	AT4G23460.1	1	GFLEALK	beta-adaptin-like protein C	0,9081	0,96427	1,0619	0,3155282	0,40046469	0,36998444
Q43866	AT3G13790.1	3	ISLDDTK,SGLGPFGLMVLASK,QLIQWPVR,GWSGIQTIPR,NQQVQNIAEPK	beta-fructofuranosidase	1,5131	2,1804	1,474	0,11932159	0,01158974	0,07150049
Q9FN08	AT5G63810.1	3	CLTGCGEPSQR,IWTENWPGWFK,HYMESFTTYIVNLLK	beta-galactosidase 10	0,74278	0,6933	0,9334	0,13337707	0,07231508	0,35485881
Q9C525	AT1G66270.1	10	DSNDAPTVSR,EAYLVSHNLLNAHAEAVEVFR,DFLAQGVRPSALK,QDSLVSWEPK,LGTTDSVDVGTADHN	beta-glucosidase 21	1,0051	2,3147	2,342	0,3146579	0,00697307	0,02006469
Q9C8Y9	AT1G66280.1	7	QDSLVAWEPK,DFLGQGVRPSALK,GPALWDIFCK,CSGHNADVAVDFFHR,NVDHSAIGSQPLTAALPVYAK	beta-glucosidase 22	0,89849	1,8684	2,0865	0,34411499	0,01255004	0,01684929
Q9SR37	AT3G09260.1	25	DFLSQGVRPSALK,HWITFNEPWVFSHAGYDVGK,YANPEIMIMENGYGEELGASDSVAVGTADHNR,SGYE	beta-glucosidase 23	0,87152	0,84118	0,96606	0,26113057	0,2397083	0,48558152
Q9LV33	AT3G18080.1	7	DFADYAEFCYK,DFHIGWFIHPLVYGEYPK,LNFDAYR,DLGYQQDWNVEFGFAK,GFVFGTATSAYQVEGET	beta-glucosidase 44	1,0448	0,84237	0,83035	0,41535462	0,20112602	0,18846418
Q93Y07	AT3G06510.1	3	LVETDEYSESGR,FGLVAVDR,TLLIWPLIMK	beta-glucosidase-like SFR2	1,2403	1,6809	1,3154	0,11932159	0,01354858	0,08303118
Q8L7S6	AT1G65590.1	2	VIEILMTPWNIIDQPR,LSHVISGDR	beta-hexosaminidase 3	0,57	0,70622	1,239	0,04661118	0,07885575	0,17416837
Q8L9C4	AT1G67730.1	6	GLNLILVAR,AFAFQLAQK,ASFLVASPEGYAK,YFHEVDEELINNLIK,YGSWAIITGPTDGIGK	beta-ketoacyl reductase 1	0,96486	0,93551	0,98511	0,2218635	0,44357684	0,19459657
Q9FGY1	AT5G49360.1	1	LGIGGYEWWSEALHGISDVGPGAK	beta-xylosidase 1	0,53568	0,2499	0,4665	0,03827781	0,00069138	0,02006469
Q9SKI0	AT2G10940.2	4	ALDLNLYVPVALQLLLTCGK,LGACVDLLGGLVK,IGLGDPAVNK	bifunctional inhibitor/lipid-transfer protein/seed storage 2S albu	0,73488	0,49141	0,68195	0,14959475	0,012231	0,05963097
Q1JPL7	AT1G11580.1	4	ITASSDLAPVK,EAEQFTVAK,DATIITGSLNVIDGSTTFR,VTANVVVAK	bifunctional pectinesterase 18/rRNA N-glycosylase	0,87615	2,0185	2,3038	0,26767321	0,01461739	0,01568383
Q9LZ82	AT5G04430.1	3	FLVSNAAAGSVIGK,NQEFFPGTTDR,LVVPNSSCGGIIGK	binding to TOMV RNA 1L (long form) protein	0,77046	1,3583	1,763	0,15250811	0,09725565	0,04052356
Q8H765		1	VLGTDELNAYLNK	Casein kinase II subunit alpha-2	2,8638	2,1468	0,74962	0,00649404	0,01140342	0,12919191
Q944A7	AT4G35230.1	5	VLFDEDGDPR,DLVATLAPLQTK,VTPESVTYSFGTVLLDLLSGK,FSTEEATVVVELASQCLQYEPR,SLCYLLCD	BR-signaling kinase 1	0,99275	0,81621	0,84032	0,34524541	0,13401989	0,11720508
Q9SZR1	AT4G29900.1	3	SDIIILDDNFESVVK,NEQGGPQFIGGK,SESGEIQVSGSPTER	Ca2+-transporting ATPase	1,3537	1,5247	1,095	0,23630779	0,14691503	0,36629974
O22218	AT2G41560.1	2	ITSISDIIEGFASEALR,INVFK,NIAGQSVYQLIVLGILNFAGK	Ca2+-transporting ATPase	1,0593	0,80419	0,75447	0,3767835	0,13272107	0,1252794
Q9M2L4	AT3G57330.1	3	VVDSNGESVPLSEEK,IASISDVIEGFASEALR	Ca2+-transporting ATPase	0,96406	1,1949	1,2461	0,36922949	0,16711671	0,14437568
Q8LEM7	AT4G26570.1	1	ETVFSVSEIEALYELFK	calcineurin B-like 3	0,91798	0,78295	0,8529	0,33099788	0,13166135	0,2293484
Q9FN48	AT5G23060.1	6	NCYIVTDGFSGGR,VISIPLEELPNK,TVTDVAQQTSK,LGTDSYNFSFAQVLSPSR,GDLTPAQTLDLLCTK,VFQ	calcium sensing receptor	0,9916	1,4411	1,495	0,46816001	0,10533992	0,12344714
Q9XIR0	AT1G64850.1	1	ANLTFEELYIAVLLVYNDINK	calcium-binding EF-hand-containing protein	11,368	1,9263	0,16944	1,06E-05	0,01800598	9,25E-05
O23052	AT1G05150.1	1	ILALLDDSGSGR	Calcium-binding tetratricopeptide family protein	0,66759	0,86331	1,2932	0,08839531	0,21781433	0,14531065
Q8L706	AT1G05500.1	1	LLPPEFYPSWVVFSER	calcium-dependent lipid-binding domain-containing protein	1,6475	1,3902	0,8438	0,05919942	0,08674062	0,21770989
Q9ZVF1	AT2G01540.1	1	SSDPYIVLNVADQTLK	calcium-dependent lipid-binding domain-containing protein	1,2346	0,96281	0,77984	0,18917409	0,39651228	0,15169864
Q9LEX1	AT3G61050.2	3	AVGGSLTAIPGLSDMIDDTVDTIVK,VIFQLADEIPCISAVVVALLAEPKPR,LTLGNVAPK,WGGDPNIVLGVTA	calcium-dependent lipid-binding domain-containing protein	0,81713	0,63215	0,77204	0,20057034	0,04843564	0,15236678
Q944I4	AT1G80380.2	5	AVDPQLEVVNK,AYLPTLYAEGPSGSDPDR,TTLVFALDYLFK,LSVETLEALSK,LFQLNELK	D-glycerate 3-kinase	0,3499	0,40534	1,1585	0,00649404	0,00603337	0,24165941
Q9LY30	AT5G07300.1	7	TIALELMR,LIVHAEESLASK,DSIVSASDLPLSILIVGVGGADYK,LDEQQFLGEATCTLSEVVTK,NDPNPLWKP	calcium-dependent phospholipid-binding copine-like protein	1,444	1,3541	0,9377	0,10367477	0,0985513	0,3637027
Q9SLE2	AT2G16760.1	1	LFLAELSGQSK	calcium-dependent phosphotriesterase-like protein	3,3862	1,7716	0,52316	0,00342053	0,02584859	0,03075562
Q9ZSA2	AT4G04720.1	1	SGTITYEELK,EVISEVDTDNDGR	calcium-dependent protein kinase 21	1,1316	1,0081	0,893	0,27345331	0,46478545	0,27951679
Q42479	AT4G23650.1	3	EDGEASDKPLDNAVLSR,LTAAEVLNHPWIR,ISEAEIR	calcium-dependent protein kinase 6	1,1173	0,93503	0,8743	0,38238029	0,35677107	0,28646833
Q0WV19	AT5G57110.2	6	WVLPEDDLILLAIVGIK,DSVVLCQNAGVK,GASEIVLASCR,AIALECGILSSDADLSEPTLIEGK,LGMNFETAR,	calcium-transporting ATPase 8	1,1923	1,2564	0,97891	0,11793516	0,02228089	0,13650515

Q9SFU6	AT3G07160.1	2	LELVQWPLFLLSSK,VLGSVLEQLAK	callose synthase	0,62121	1,0036	1,6361	0,04823352	0,38370679	0,05476911
Q9SJM0	AT2G36850.1	2	ALIEVLEVLSR,IDENAVNEVFLK	callose synthase 10	0,85454	1,1252	1,3273	0,21721359	0,2395332	0,11896564
Q9ZT82	AT4G03550.1	1	VVNVLQSLYEIATR	callose synthase 12	1,2132	1,4219	1,1721	0,20541826	0,077212	0,2297029
Q9STW1	AT4G24330.1	4	NFSLLGVGEGEDSPLLLK,LIALIPYYIDLIGR,ENENLALAWASK,FALPDIK	uncharacterized protein	0,35208	0,31726	0,90109	0,00649404	0,00229085	0,29503313
P29402	AT5G61790.1	11	SEGHEDYGLLVSEK,ELDEPLNLK,YLRPQEAGWTPQGFDSESPYSIMFGPDK,AEEEAAGSADGLK,ITELIEK,N	calnexin 1	1,9041	2,7192	1,3059	0,12801913	0,10453019	0,45590666
Q38798	AT5G07340.1	2	VFDLLYK,GNLLSAEDFEPPLIPSK	calnexin2	1,1985	1,0493	0,87552	0,21662245	0,37511822	0,2580432
O04151	AT1G56340.1	4	YVGVELWQVK,QTGSLYSDWDLLPAK,LLSDDVDQTK,EAEETDAAHDEL,SGSLFDNVLVSDDPEYAK	calreticulin-1	1,2027	1,2806	1,1102	0,36417609	0,1318416	0,20888524
O04153	AT1G08450.1	2	IEQDIECGGAYIK,AGSIFDNILICDDPAYAR	calreticulin-3	1,7971	2,1114	1,2817	0,17788236	0,02004869	0,07873399
Q9LZQ4	AT3G62360.1	8	IGSANNQIER,ISFVPQDSR,SSIDVNVGTEDIK,VLGAVGGESCLIK,VALLSGQPQEGVAIEAR,TVVLTDESSQF	carbohydrate-binding-like fold-containing protein	1,0282	1,3017	1,2594	0,35896271	0,09255401	0,15245963
P27140	AT3G01500.2	6	VISELGDSAFEDQCGR,DVATILHWK,GGYYDFVK,AFDPVETIK,VCPSHVLDFQPGDAFVVR,YGGVGAAIEY	carbonic anhydrase 1	0,99926	0,77796	0,82159	0,21520133	0,09060668	0,25175877
Q3E9H5	AT5G14740.1	5	VLAESESSAFEDQCGR,YAGVGAAIEYAVLHLK,VCPSHVLDFHPGDAFVVR,ITAELQAASSSDSK	carbonic anhydrase 2	0,84275	0,65196	0,7736	0,22262747	0,05301425	0,14531065
P42737	AT5G14740.2	4	VLAESESSAFEDQCGR,YAGVGAAIEYAVLHLK,ITAELQAASSSDSK	carbonic anhydrase 2	0,78156	0,56745	0,76332	0,09951543	0,01688601	0,12730146
P32826	AT3G10410.1	1	FLNLQSVR	carboxypeptidase	0,64127	0,88586	1,3814	0,07413148	0,25211857	0,11381838
Q27GI8	AT5G23210.2	1	DSYNFLVNWFK	carboxypeptidase D	0,95937	0,88461	0,92207	0,40683029	0,25032609	0,33339265
Q0WPR4	AT5G23210.1	1	GLMIGNALLDDETDQK	carboxypeptidase D	0,88953	0,59419	0,66798	0,28952208	0,0325878	0,07927577
O65572	AT3G63520.1	9	FGVLPR,GIYDLGEGR,LLDLLER,DELMIR,LSASAVDFPR,INECYTGK,VPYGFHALFVTEEQLQEQTLI,MLEV	carotenoid cleavage dioxygenase 1	1,1154	0,94168	0,84429	0,29841028	0,34935543	0,21796349
Q42267		1	LIIQNQDEMIK	Carrier protein	1,0231	5,5722	6,3463	0,49709532	0,05872587	0,07871651
Q9SMV6	AT5G17020.1	1	NYISEVIVQLSSNEASFR	exportin 1A	0,35214	0,47649	1,3531	0,00649404	0,01223283	0,12375524
Q9LF37	AT5G15450.1	1	AIDLVDEAAAK,VNLEIQQAER	casein lytic proteinase B3	0,87636	1,5869	1,9179	0,32843915	0,0693958	0,06387722
P25819	AT4G35090.1	4	YPTPPAVCSGK,GPILLEDYHLVEK,ITHEIR,EGNFDLVGNNFPVFFIR,TWPEDILPLQPVGR	catalase 2	1,3015	1,0249	0,80224	0,17677818	0,4495652	0,17667834
Q42547	AT1G20620.1	10	VANFTR,DEEINYYPSK,TNIQEYWR,WVEILSEPR,LTHEIR,HMEGFGVHTYTLIAK,DGIQFPDVVHALKPNPK	catalase 3	1,7492	1,0835	0,60221	0,03918435	0,25069453	0,0571781
Q9C5D6	AT1G05940.1	2	SLASYAVALLELFPALK	cationic amino acid transporter 9	0,53185	1,7047	3,2053	0,0374089	0,03085824	0,00380368
Q9SI85	AT1G62630.1	1	VATVEDIIITLLR	CC-NBS-LRR class disease resistance protein	0,53964	1,0573	1,9593	0,0390965	0,35819229	0,0277367
Q8LBA6	AT1G68000.1	1	DSTSWLFR	CDP-diacylglycerol--inositol 3-phosphatidyltransferase	0,98436	1,1785	1,1972	0,45993579	0,1985399	0,20692895
B9DI55	AT3G09840.1	1	DFSTAILER	cell division control protein 48-A	1,0655	1,9464	1,8268	0,36754088	0,01738877	0,0355902
Q9FGM0	AT5G53170.1	1	LVSDTEVSELETNDR	cell division protease ftsH-11	0,87713	1,6911	1,928	0,26938059	0,03205345	0,03006281
Q84WU8	AT2G29080.1	2	FAPTLLLLGTLIYGAR	cell division protease ftsH-3	0,79778	2,0318	2,7142	0,13560953	0,01421162	0,0079484
Q9FH02	AT5G42270.1	2	DGSVLQLTAVDNR,GGQGGAGGPGGLGGPMDFGR	cell division protease ftsH-5	1,1769	1,112	0,93925	0,24243794	0,32151554	0,34438321
Q8W585	AT1G06430.1	1	AYEIALSQIR	cell division protease ftsH-8	1,0708	1,6857	1,5743	0,36149069	0,03240953	0,0659409
Q0WWL4	AT3G19820.3	12	TWVDYFVK,CLYWEGK,AIGTFMSVYYK,GQIYPEPGFEYENR,YLLGWLMPPK,LILPFGDQFWFR,VGDALEV	cell elongation protein DIMINUTO	0,85823	0,87345	1,0346	0,36257523	0,30424444	0,45316148
O48946	AT4G32410.1	1	INALVAK	cellulose synthase A catalytic subunit 1 [UDP-forming]	1,2318	1,5215	1,2365	0,19348061	0,0543413	0,17307293
Q8VZK9	AT1G55850.1	3	ALASCTYEENTQWGK,EGNTIAIPTLVYLSR,ANCLDSAAEEVAK,EIAFVQFPQCFDNVTR	cellulose synthase-like protein E1	0,86635	1,2656	1,464	0,21738085	0,16177995	0,08680049
P41088	AT3G55120.1	1	LLAEAVLESIIGK	chalcone--flavonone isomerase 1	1,2456	5,1023	4,0962	0,18321838	0,00024074	0,0008235
Q84VQ4	AT5G21430.1	1	NCSYDQVTIGYK	chaperone DnaJ-domain containing protein	1,1643	1,095	0,94044	0,2412962	0,29657824	0,36785181
Q7XAR9	AT5G42480.1	1	ILAAGVAIGLISLFSQK	chaperone DnaJ-domain containing protein	0,98186	0,3395	0,34577	0,45449884	0,00271908	0,00626206
Q39079	AT2G35720.1	1	AQQLLQTVATR	chaperone protein dnaJ 13	1,0873	1,5643	1,4386	0,33405095	0,04778386	0,09532433
Q3EAQ4	AT3G44110.2	1	SNPGEVVKPDSYK,VSLEDVYLGTMK	chaperone protein dnaJ 3	1,3247	1,497	1,282	0,20006354	0,07284323	0,22202096
Q94AW8	AT3G44110.1	1	SNPGEVVKPDSYK	chaperone protein dnaJ 3	0,93521	0,96805	1,0351	0,36304362	0,40965488	0,42018922
Q9SH87	AT2G38000.1	1	ETQVLVPNSETVEK	chaperone protein dnaJ-like protein	0,58047	0,74156	1,2775	0,04823352	0,10051964	0,15355836
Q9SIF2	AT2G04030.1	2	GVVDSDDLPLNVSR,NYSQFVGFPIYTWQEK	Chaperone protein htpG family protein	0,83827	0,8447	1,0077	0,21912883	0,19358982	0,48115087
O65282	AT5G20720.3	4	TAGGLLLTETTK,YAGTEVEFNDVK,ITPLPVSTGSTVLYSK,EDDIVGILETEDIK	chaperonin 20	1,4349	1,4188	0,98879	0,10667054	0,0779512	0,47086949
P29197	AT3G23990.1	6	IGGASEAEVGEK,NVVIEQSWGAPK,CELDDPLILIHEK,TALVDAASVSSLLTTTEAVVVDLPK,GYTSPYFITNQ	chaperonin CPN60	1,6382	1,6303	1,1373	0,18321838	0,0118808	0,04699898
Q8L7B5	AT2G33210.1	4	TALVDAASVSSLLTTTEAVVTEIPTK,CELEDPLILIHEK,AAVEEGIVPGGGVALLYASK,LSTANFDQK	chaperonin CPN60-like 1	1,9602	3,4675	1,8746	0,06099168	0,00087078	0,01546244
Q93ZM7	AT3G13860.1	3	AAMLQGVSEVAEAVK,VTDALNATR,ALDNLQTENEDQR	chaperonin CPN60-like 2	1,0403	1,8426	1,7712	0,40976466	0,02168291	0,03974861
P21238	AT2G28000.1	15	HGLLSVTSGANPVSLK,LADCVGLTLGPR,VGAATETELEDR,AALQAGIDK,AVASISAGNDDLIGSMIADAIDK	chaperonin-60alpha	1,1778	0,94399	0,7666	0,13996266	0,07596297	0,35059684
Q96282	AT5G49890.1	1	APVGGVLFALEEAASWWR	chloride channel protein CLC-c	0,25646	0,881	3,4353	0,00177495	0,24489993	0,00266707
Q7M1L1		1	LGALGCVFPELLAR	Chlorophyll a/b-binding protein type I	0,8256	0,93899	1,1696	0,36417609	0,39518157	0,4365101
P04777	AT1G29920.1	5	YLGPFSGESPSYLTGEFPGDYGWDTAGLSADPETFAR,GPIENLADHLADPVNNNAWAFATNFVPGK,VAGN	chlorophyll a-b binding protein 2/3	0,82214	0,84068	1,0223	0,22731052	0,20911579	0,46793219
P27521	AT3G47470.1	9	WFVQAELVNGR,IGIINVPEWYDAGK,LAMLAFLGFVVQHNVTGK,GEVGYPGGIFNPLNFAPTQEAK,WAM	chlorophyll a-b binding protein 4	1,312	1,3559	0,99988	0,17134952	0,11764001	0,42312141
Q9XF89	AT4G10340.1	18	YGANCGPEAVWFK,SEIPEYLNGEVAGDYGYDPFGLGK,WAMLGAAGFIIPEALNK,HLSDPFGNNLLTVIAGT	chlorophyll a-b binding protein CP26	2,2263	2,7796	1,0642	0,36226386	0,27777756	0,41886759
Q07473	AT5G01530.1	6	STPFQPYSEVFGIQR,TAQLQLAEIK,LAMVAFLGFAVQAAATGK,NLAGDVIGTR,FFDPLGLAADPEK	chlorophyll a-b binding protein CP29.1	1,231	1,1711	0,92647	0,41872923	0,3657488	0,45590666
Q9XF88	AT3G08940.2	5	AQLQLAEIK,FFDPLGLASDPVK,TVISDRPLWFPGAK,NLYGEVIGTR,LAMVGFLGFAVQAAATGK	chlorophyll a-b binding protein CP29.2	1,0306	1,0165	0,93528	0,29763493	0,09798439	0,19116842
Q9S7W1	AT2G40100.1	2	IYPGGYFDPLGLAADPEK,GPVSFLATFNN,GFDPFGLGKPAEYLQYDFDGLDQNLAK	chlorophyll a-b binding protein CP29.3	1,2247	1,5401	1,2216	0,23646531	0,10801739	0,26095203
Q38833	AT3G51820.1	2	YQASAQPFLVLGIFVTALASQH,ALGLQSLPVAFGTETAK	chlorophyll synthase	1,1594	1,2013	1,0362	0,245877	0,17847855	0,41886759
O22527	AT1G19670.1	2	LLPPGGQVEVDDAGSVINWASENLK,SFVGGIVVAFLK	chlorophyllase 1	1,0869	0,95428	0,94794	0,08482301	0,19026959	0,15627224
Q9STW6	AT4G24280.1	1	VVDWLAAEFK	chloroplast heat shock protein 70-1	1,1808	1,2699	1,0719	0,24318492	0,23708007	0,45982422
Q93WG3	AT1G08640.1	1	SFEAPSSPIYNEEGEESGR	Chloroplast J-like domain 1-containing protein	1,4074	1,7242	1,2251	0,11445402	0,02912464	0,18361577
O80565	AT2G43950.1	2	HVSVLYDVEEK,VTSEFDSDSLLFLNK	chloroplast outer envelope protein 37	0,99442	0,94141	0,94669	0,48619109	0,34889454	0,37789765
Q9LYA9	AT3G63140.1	10	ELLSAGHAVTILTVGDESSEK,ILEALK,DLSSMLTSAVANPEAASGNIFNCVSDR,DLDTVRPVVDWAK,DLLGV	chloroplast stem-loop binding protein-41	1,1033	0,96815	0,8775	0,31373782	0,40972098	0,26059071
Q9SRH6	AT3G01290.1	11	LNLDDVFEQK,LQQLDVQCETK,ASDAFYR,DVLDMVMMTQYFDTMR,VLNPGLQFVPWVIGDYVAGTLTL	Hypersensitive-induced response protein 3	2,7526	2,0264	0,83725	0,00688603	0,00741687	0,20692895
O49340		1	ICPGINLALGLVEVTVANLVGR	Cytochrome P450 71A12	2,7992	4,0257	1,4382	0,00688603	0,00068018	0,09537544
P48523	AT3G19450.1	2	LADSLDYIIDTVPVFHPLDPYLACLK,GLTSTIETVK	cinnamyl alcohol dehydrogenase 4	1,2203	1,1496	0,94212	0,20006354	0,22817556	0,37046625
Q0WUX6	AT2G42790.1	1	NLADEVFSIVGK	citrate synthase 3	1,24	1,5055	1,2081	0,18561901	0,06361783	0,21316325
P20115	AT2G44350.2	2	VPVVAAYVYR,VLPTAQSGAEPLPEGLLWLLLTGK	citrate synthase 4	0,86999	1,4051	1,6859	0,18877558	0,09297249	0,04773426
Q9FPF0	AT3G14990.1	4	ATTHPVVSDK,LVAEVLLDEVAEK,LAATCATAVESR,TVLIPIAHGTEPLEAVAMITVLR	class I glutamine amidotransferase domain-containing protein	0,96163	0,81947	0,88916	0,3847187	0,30839936	0,43108996
P10798	AT5G38410.1	1	QVQCISFIAYKPPSFTEA	ribulose bisphosphate carboxylase small chain 3B	0,35817	0,35232	0,98367	0,00688603	0,00310132	0,45820061
Q0WLB5	AT3G08530.1	1	WLVLIGIAPGSPER	Clathrin, heavy chain	1,1051	1,9267	1,7435	0,31147744	0,01800598	0,04246453
Q0WNJ6	AT3G11130.1	5	VEEDAVWSQVAK,IIYAFISNWAK,ADDTTQFLEVIR,ASEDTNVYDDLVR,WLVLIGIAPGSPERPQLVK	Clathrin, heavy chain	1,0252	1,2509	1,2582	0,3155282	0,08821698	0,16132595
Q94A40	AT1G62020.1	2	VWDIGALR,TGLQTFR	coatomer subunit alpha-1	0,62578	0,80797	1,2911	0,06532174	0,15341453	0,14567479
Q9SJT9	AT2G21390.1	6	GPPALVFDFSQLEAK,TGIQTFR,LAIELGDNVPSLPEGK,SLLDTNPTIESQAR,VFLSILQTIPLVVVESR	coatomer subunit alpha-2	0,9735	1,0041	1,0604	0,15102512	0,30146007	0,21194888
Q9CAA0	AT1G79990.3	1	SFDVTELPVR	coatomer subunit beta'-1	1,2276	1,6121	1,3132	0,19432574	0,04138367	0,13614308
Q9SV21	AT4G31480.2	3	NAILAIMSIYK,LLDTFYQIR,AVNYLLSNVDK	coatomer subunit beta-1	1,6288	1,866	1,1456	0,06099168	0,02063113	0,25418836
B9DGK9	AT5G05010.2	2	QSNILEDLATLTLLSK	coatomer subunit delta	1,2609	2,2945	1,6539	0,11207126	0,35095504	0,12852101
B9DHT9	AT4G34450.1	10	QITNFMSDIADEFK,VEATEVFFSVTK,ELTPAITVLQLFLSSPRPVLR,IIDGTLLTQIER,GAVLQEAR,AIVDSIVT	coatomer subunit gamma	0,8538	1,1965	1,3766	0,15302081	0,42403285	0,15909481
Q5XEU8	AT3G15000.1	1	TLAQIVGSEDEAR	cobalt ion binding protein	0,96413	2,9406	3,05	0,4153556	0,00271908	0,00479953
Q9FM65	AT5G55730.2	1	VGFGPDGGDLSSFFVK,IVDTLIDEQPLAIYATDK	fasciclin-like arabinogalactan protein 1	0,3768	0,50994	1,343	0,00688603	0,012231	0,13178257
O64603		1	IAVGGSIAR	Contains similarity to equilibratiave nucleoside transporter 1 gb|	1,3071	1,4195	1,1365	0,16227175	0,07190364	0,27224867
O04645		1	WSVLNTILLLR	contains similarity to Synechococcus PCC7942 chromosomal reg	1,0329	1,2173	1,1785	0,42303608	0,16711671	0,22455086
Q9ZUX4	AT2G27730.1	2	VAGATASASAESGPK,QGPGEQAAGSASEAK	copper ion binding protein	1,1493	2,8793	2,5679	0,22007147	0,01044196	0,03470465
Q93VM8	AT5G20650.1	2	SSSGVSAPLIPK,ATILFDFWK	copper transporter 5	0,80564	0,8447	1,0295	0,10834393	0,03104125	0,21472133
P15456	AT1G03880.1	2	QNNIFNGFAPEILAQAFK,FVIEPQGLFLPTFLNAGK,TNENAQVNTLAGR	cruciferin 2	0,87787	1,5509	1,8742	0,31014605	0,12726868	0,09959821
Q96318	AT4G28520.1	15	YVIEQGGLYLPTFFTSPK,QGQQGQPWEGQGQQGQQGFR,NLWSGFDAQVIAQALK,TNENAMISTLAGR,I	cruciferin 3	0,77762	1,0876	1,4234	0,1468236	0,33893255	0,1072668
Q8L5Y6	AT2G02560.1	1	LSSIILQQLDDVAGDVSGLAVK	cullin-associated NEDD8-dissociated protein 1	0,54758	0,8314	1,5183	0,04046287	0,17732078	0,07594912
O23629	AT3G45980.1	2	KPAAEKPVEEK	histone H2B	0,36438	0,35631	0,97786	0,00745712	0,00324569	0,44559591

Q9LEW2		2	LSFPSQTATAYNK,LPIPSTVFSTPGALIDSGTVITR	nucleoid DNA-binding protein cnd41-like protein	0,36835	0,48857	1,3264	0,00766485	0,01383439	0,13180052
P38605	AT2G07050.1	9	IAEGGSPWLR,NSQVLEDCPGDLNYWYR,TAIEHIHYEDENTR,QQPSGGWGESYLSCQDK,VYSNLDGNR,F	cycloartenol synthase	1,0481	1,1124	1,0613	0,39433221	0,27314825	0,37046625
Q9LM02	AT5G13710.2	2	EIFTPMYFFLAR,VSNFLEQAAEGLVDGGR,VLDVGCGIGGPLR	cycloartenol-c-24-methyltransferase	1,1673	2,4011	2,0892	0,21184587	0,00759839	0,02908101
P55217	AT3G01120.1	1	ADILQALEAI	cystathionine gamma-synthase	1,1664	1,5948	1,3673	0,23936463	0,04359913	0,11842417
Q0WWF3	AT4G39090.1	1	APILPTENLPEDFDWR	cysteine proteinase RD19a	0,76638	1,2617	1,6464	0,14959475	0,13839266	0,05485971
P43297	AT1G47128.1	5	SWGESGYLR,CGIAIEPSYPIK,AVAHQPISIAIEAGGR,GVDGTCDQIR,VVTIDSYEDVPTYSEESLK	cysteine proteinase RD21a	1,096	1,0959	0,98491	0,21902323	0,16408394	0,44368492
P47999	AT2G43750.2	1	YLSTQLFQSIR	cysteine synthase	0,82736	0,77727	0,93946	0,20935831	0,12693655	0,36664065
Q9S757	AT3G61440.1	3	YLSSVLFDELR,TPLVFLNK,SFGAELVLTDPAK,VTEGCEAYVAAK	cysteine synthase C1	1,4384	1,351	0,92966	0,11816398	0,16136326	0,2610145
Q8GYA4	AT4G23180.1	1	YSNQNILSTLITTGGVILVNTR	cysteine-rich receptor-like protein kinase 10	1,4499	1,3333	0,91958	0,1020706	0,10726857	0,32893826
Q9LRJ9	AT3G22060.1	2	CSDIEGSFTSK,SCLETAIPELR	cysteine-rich repeat secretory protein 38	1,1952	1,4565	1,2187	0,21818685	0,06898957	0,1886216
Q9LV60	AT5G48540.1	1	VYGLAQCR	cysteine-rich repeat secretory protein 55	1,4889	3,7318	2,5064	0,09180655	0,0009503	0,01166119
P42792		3	DVEGGWLLR,IAFYPYFYVK,GIPNSYTDETDHT	Cytochrome b	1,0194	1,3228	1,2976	0,45267591	0,11129187	0,1439429
Q42342	AT5G53560.1	4	YFIGEIDSSSVPATR,TYVAPQQPAYNQDK,VLSFEEVSK,DATNDFEDVGHSDTAR	cytochrome b5 isoform 1	1,6709	1,7218	1,1567	0,32343528	0,20024699	0,14773663
Q9FDW8	AT1G26340.1	1	YFIGELDESSLPEIPELK	cytochrome B5 isoform A	0,65433	0,85683	1,3095	0,08067319	0,20850578	0,13799311
Q9ZWT2	AT5G48810.1	2	DCWIVIDGK,FLDDHPGGDEVILTSTGK,AMLDEYYVGDIDTATVPVK	cytochrome B5 isoform D	0,72579	0,89547	1,1844	0,11399376	0,14149413	0,28053259
P56779		3	SFADIITSIR,QGIPLITGR,FDSLEQLDEFSR	Cytochrome b559 subunit alpha	1,4599	1,9388	1,3269	0,23075357	0,15817724	0,40213088
Q9SWS1	AT5G38630.1	1	VTFLQVNQVITR	cytochrome b-561	1,5101	1,7073	1,1306	0,08466166	0,0307626	0,27044118
Q96303	AT2G38940.1	2	EQLQVLNALDVAK,VLQVEIEPEQQK	inorganic phosphate transporter 1-4	2,6498	1,8514	0,6987	0,00902119	0,02133519	0,09745137
P56773	ATCG00720.1	6	IVTGVPDAIPVIGSPLVELLR,GSASVGQSTLTR,VYDWFEER,LEIQAIADDITSK	cytochrome b6	1,9176	1,8822	1,0316	0,14577531	0,07488229	0,32505166
O80725	AT2G47000.1	5	FYDPDSGEITLDGVEIK,FYDPQAGDVLIDGINLK,QQTGLVSQEPILFNETIR,LSADAATIR,DVYFTYPARPDEC	ABC transporter B family member 4	2,5087	2,7649	1,102	0,01009523	0,00296739	0,30638073
Q9ZR03	AT4G03280.1	4	FLCPCHGSQYNAQGR,DALGNDVVAAEWLK,GPAPLSLALAHADIDEAGK	cytochrome b6-f complex iron-sulfur subunit	1,5362	1,541	1,0278	0,0599686	0,03587438	0,48027602
Q9S7L9	AT1G22450.1	2	FPTTNQTR,EVAPEVTTQAEEVK,QDTSAKPVEVK	cytochrome C oxidase 6B	1,0485	2,082	2,0798	0,36464091	0,01738877	0,0355902
P93285		2	IIVTSADVLHSWAVPSLGVK	Cytochrome c oxidase subunit 2	1,1546	2,0697	1,6491	0,33500193	0,05807164	0,05625382
Q9SSB8	AT1G80230.1	1	SFECPVCTQYFK	cytochrome c oxidase subunit Vb	1,078	2,6622	2,4697	0,34845914	0,00395658	0,01197314
Q9FKT8	AT5G56090.1	1	LALPVSLIVGITAISGAFVAGNDAGR	cytochrome c oxidase subunit XV assembly protein	0,79222	2,0156	2,5442	0,172787	0,01472171	0,01090936
O23138	AT1G22840.1	1	ADLIAYLK	cytochrome C-1	2,0458	4,6709	2,2831	0,02734734	0,00033184	0,01625845
Q8L5Z1	AT1G33811.1	2	AYTYPINIQELANL,TYVDALAQILGFR	GDSL esterase/lipase	2,5715	1,5595	0,60646	0,0106699	0,04862524	0,05476911
P56771	ATCG00540.1	10	GGYEITIVDASNGR,LDQPLTSNPNVGGFGQGDAEIVLQDPLR,YSEITFPILAPDPATNK,IGNLSFQNYRPNK,	cytochrome f	2,068	2,0736	0,94297	0,09425809	0,10833354	0,29315211
Q9FI39	AT5G47990.1	7	LFVNVYAIMR,FFNFPIVLVSSASTAYEIFK,LGISLFAK,LVQETDLPNLPYLQAIVK,GLVTESIFLTK	cytochrome P450 705A5	1,7128	0,92139	0,54372	0,04306948	0,15790714	0,01867507
Q8L7D5	AT5G48000.5	3	ESTSSVTSLAIK,APLPYFPK,IISNLKPETQATLVDNIMALGSEWFQSPLK	cytochrome P450 708A2	1,7396	1,0125	0,58204	0,04741018	0,46269536	0,04559256
Q9FYC2	AT3G44880.1	3	VESSGPWGFQGANDDSPR,GAYNSFQILK,LVLAGLSLISAASAYALHEQEK	pheophorbide a oxygenase	2,5419	2,5875	1,0179	0,01098118	0,00470436	0,45500202
Q9M817	AT1G52190.1	1	SLFTGLAQAIVAAYK	putative peptide transporter	0,39512	0,28481	0,7208	0,01117442	0,00128936	0,11195408
Q9LIP6	AT3G26300.1	1	IIDLLTGLQGR	cytochrome P450 71B34	1,3168	1,0153	0,77104	0,14437791	0,45545505	0,14396051
Q9LIP3	AT3G26330.1	1	EGVEDFVDLLLK	cytochrome P450 71B37	0,78839	1,0912	1,354	0,10367477	0,30606248	0,13180052
Q8L6Z6		1	LTNFFELVIDDHLK	cytochrome P450 71B5	0,65413	0,38127	0,58286	0,08067319	0,00431771	0,04560558
P56774	ATCG00730.1	2	KPDLNDPVLR,LLGVLLMVSVPAGLLTVPFLENVNK	cytochrome b6/f complex subunit IV	0,39674	0,49732	1,2535	0,01135077	0,0148253	0,1644441
Q96514	AT1G13110.1	6	FGPVMLLHFGFVPVVVISSK,QVSENPDVVDVMLDLMK,IQGYDIPVK,TQDLECCSRPETVATR,TLFTLVASI	cytochrome P450 71B7	1,0196	1,6744	1,5331	0,2339919	0,34358374	0,30776669
Q9T076	AT4G27520.1	2	GADSVLEVNK,VDDGDSEISLDR	early nodulin-like protein 2	0,41046	0,47787	1,1642	0,01347706	0,01244755	0,236846
O22203	AT2G40890.1	3	VQEEFDR,LGASLSIAEHIPWLR,DCNLPENR	cytochrome P450 98A3	0,78558	1,0676	1,3591	0,16645652	0,33973072	0,12190558
O49650		4	IFINVWAIHR,FDFQGLVK,EGSPVNLGEQIFLSIFNVVTR,FGLVLK	cytochrome P450 like protein	1,4126	1,0654	0,75422	0,11371281	0,34358374	0,13180052
Q9SI49	AT2G14100.1	1	IIVEHEEK	cytochrome P450, family 705, subfamily A, polypeptide 13	1,1705	1,2152	1,0381	0,23630779	0,16863695	0,41453009
O23391	AT4G15350.1	1	ILGPQALER	cytochrome P450, family 705, subfamily A, polypeptide 2	0,73834	1,0148	1,3745	0,13072322	0,4568332	0,11591242
O49652	AT4G22710.1	1	EGSPVNLGEQIFLSIFNVVTR	cytochrome P450, family 706, subfamily A, polypeptide 2	0,83679	0,97377	1,1637	0,21818685	0,42440272	0,23725372
Q9STI1	AT4G12300.1	3	LLSEPNVSDFFPWLAR,IPLVALPIPR,LTIVVNSPSLAR	cytochrome P450, family 706, subfamily A, polypeptide 4	0,82907	1,7559	2,1179	0,21184587	0,02663721	0,02060502
Q9STI0	AT4G12310.1	2	LTVVVNSPSLASEILK,TPLVALPIPR	cytochrome P450, family 706, subfamily A, polypeptide 5	0,74601	0,85455	1,1455	0,13472408	0,20570812	0,25418836
Q9LUC9	AT3G14650.1	1	IFELQAELAQLIIQTVR	cytochrome P450, family 72, subfamily A, polypeptide 11	0,63641	1,3635	2,23	0,07543942	0,49740325	0,06968708
Q9ZNT1	AT5G17770.1	8	FVFELPTSTSVLGLPIGQHISCR,DGQGEDVIKPYTPTTLDSDVGR,FELVIK,EMIQTHCPAPASDIQILR,AFGMI	cytochrome-b5 reductase	1,2647	1,6615	1,232	0,18353589	0,12620998	0,42018922
Q0WLF5		1	LFVAIFPSFGER	cytosolic O-acetylserine(thiol)lyase	1,562	0,85582	0,5479	0,07358752	0,20779961	0,0355902
O49485	AT4G34200.1	7	AHAIGVDLVSFDEALATADFISLHMPLTPTTSK,NVAQADASVK,VLLDGSPESPLETITVQLSNVESK,ALDAGI	D-3-phosphoglycerate dehydrogenase	1,018	1,7115	1,5404	0,12985308	0,11009919	0,44382762
O04130	AT1G17745.1	2	LAVQLASGGK,EAQEGVAIEIAEAVAGALK	D-3-phosphoglycerate dehydrogenase	0,57294	1,0078	1,759	0,04700634	0,47673687	0,04077339
A8MRC4	AT1G30230.2	1	WYNHIDALLR	elongation factor 1-delta 1	2,435	2,2173	0,91057	0,01347706	0,00985441	0,31242979
Q93ZG7	AT3G53110.1	2	FTGITAELAVPDSTR,VNLVVNYNLPTK	DEAD-box ATP-dependent RNA helicase 38	1,0605	1,0041	0,9468	0,37451204	0,48770663	0,37791345
Q9M2F9	AT3G58570.1	1	TPILVATDVAAR	DEAD-box ATP-dependent RNA helicase 52	0,94954	0,70973	0,74745	0,38583106	0,08084458	0,12852101
Q56XG6	AT5G11200.1	1	ILVATDLVGR	DEAD-box ATP-dependent RNA helicase 56	0,69513	1,7252	2,4819	0,10545331	0,02907051	0,01197314
Q8LEG8		4	YGDYIGIR,CSYQSFDVEQWLASDTSPYELSAVR,NLEAWGGLMGPGYNYYER,GNLDIFSGK	dehydration stress-induced protein	1,8203	1,1761	1,0031	0,41634694	0,16954179	0,230691
P42759	AT1G20450.1	1	TVEEENQGVMDR	dehydrin ERD10	0,96415	0,6616	0,6862	0,4153556	0,05670342	0,08876987
P42763	AT1G76180.2	4	VHISEPEPEVK,HESLLEK,DETKPEETPIASEFEQK,KPEDGSAVAAAPVVVPPPVEEAHPVEK	dehydrin ERD14	0,92392	0,39418	0,42451	0,33123166	0,00445771	0,01390553
Q9SLJ2	AT1G54410.1	1	IGDALHIGGGNK	dehydrin family protein	1,6579	0,83158	0,5016	0,05725506	0,17733666	0,02638708
Q9S9W2	AT4G05530.1	1	FGLEGASVVVSSR	dehydrogenase/reductase SDR family member 4	1,4099	3,4528	2,449	0,11399376	0,00136455	0,01225561
Q9SFH9	AT1G69740.2	1	HGLVQEVAK	delta-aminolevulinic acid dehydratase	1,3356	1,0898	0,81596	0,13687527	0,30424444	0,1833606
Q9ZS88	AT4G04860.1	1	LVTNFLYFR	derlin-2.2	1,0591	0,91847	0,86724	0,37638256	0,30531068	0,2471388
Q94BV7	AT4G05020.1	3	VMEDVSAIFSK,NLNNSQYEVQIISPR,VVLLGTGWAGTSFLK	NAD(P)H dehydrogenase B2	2,4173	2,968	1,2278	0,01404151	0,00269651	0,18165562
A8MQE0	AT1G80380.3	1	TTLVFALDYLFK	D-glycerate 3-kinase	0,2682	0,63284	2,3595	0,00210428	0,04546667	0,01400248
P50700	AT4G11650.1	1	TNCNFDSSGR	osmotin-like protein OSM34	2,4094	3,9173	1,6258	0,01413533	0,00074766	0,05779597
Q39077		1	VVSNFVAQALR	diamide resistance gene	1,0905	1,266	1,1609	0,3299506	0,13614272	0,23990571
Q9FMF7	AT5G64290.1	6	SLSLSAGSKPNDSSSR,LGSYLIQSQFQCAGNSSALFLTAAAQNLLCLK,AASLPAIISLLCTPLILYK,DTPEAPGIA	dicarboxylate transport 2.1	1,2304	1,8351	1,2697	0,40683029	0,17392991	0,2413593
Q9LXV3	AT5G12860.1	3	QTIGWTDWAK,YGLIEWFSQTVVK,AGGIFLPLVK,SLCVACGSNVGDGTEHR	dicarboxylate transporter 1	2,1292	2,8743	1,3093	0,07507334	0,01662694	0,15858849
Q9LUA6		5	AAGMALAQHPVVNASCK,VGYPVNTDALDALYEK,ITASDVETAAGIAPSK,IVYGADLAAFLQTFAK,NMIESL	dihydrolipoamide S-acetyltransferase	1,2483	0,81106	0,65618	0,16884263	0,175255	0,07417003
Q9M5K3	AT1G48030.2	2	HSFANHGIK	dihydrolipoyl dehydrogenase 1	1,3538	2,4416	1,8035	0,13092819	0,00626693	0,0366822
Q9LZR9	AT5G03690.2	1	YAVICQENGLVPIVEPEILVDGSHDIEK	fructose-bisphosphate aldolase, class I	2,4066	1,6038	0,66643	0,01413533	0,04253299	0,07873399
Q9ZRQ1	AT5G55070.1	2	ISTSADAVSHVAPSEK,DAFLEK	dihydrolipoyllysine-residue succinyltransferase component of 2-	1,4438	1,6076	1,2098	0,31371371	0,04917429	0,10386097
Q9SZ31	AT4G26910.1	1	SEDTASQVTPSQK	dihydrolipoyllysine-residue succinyltransferase component of 2-	1,426	1,8071	1,2673	0,10942681	0,02365712	0,15741367
Q0WRX8	AT5G23300.1	2	LAVSAAAR,RPDPAILGLEVWGR	dihydroorotate dehydrogenase	1,5625	3,0791	1,9707	0,07358752	0,00243954	0,02760656
Q5XPY9		2	LAFLNNDYSQFGTGTK,LTSTELSTYASTK	DING protein	1,1933	1,2296	1,0305	0,21902323	0,15817724	0,43108996
Q9LSB4	AT3G15950.1	4	EASQAYEEYHEAVHK,QLNLDDFSEGDDSAESAR,FTSLTTSLSELLK,EFEAAHAGFEQLK	DNA topoisomerase-like protein	1,0022	1,0836	1,0772	0,42754007	0,41077942	0,36785181
Q05212	AT3G04880.1	1	TSPETALEIFDAWIK	DNA-damage-repair/toleration protein DRT102	1,0292	1,3007	1,2638	0,42903391	0,12138972	0,15812838
Q93ZH5	AT1G21080.2	1	VCEQVLQDPTAK	DNAJ heat shock N-terminal domain-containing protein	1,087	1,2762	1,1741	0,33443041	0,1318416	0,22896149
Q93ZY3	AT5G19690.1	2	SLSLLDPTYASK,AAWEIFNSLDVK,GSGAGLAAAALLAMVPSYISR,DGLHVFDDFR	dolichyl-diphosphooligosaccharide--protein glycosyltransferase	1,3478	1,4288	1,141	0,15606633	0,06908648	0,27374873
Q9LVR2		2	VPDVYGVFQFK,VLVLLDDLSLK	dolichyl-di-phosphooligosaccharide-protein glycotransferase (oli	1,0644	1,7482	1,6424	0,36922949	0,02701153	0,05550763
Q8LDT6		9	YGQYLYDGLIIFAPSTER,GFDLDFK,DLILSADTAASDLIR,VPDVYGVFQFK,VLVLLDDLSLK,LSSPPQLTGSSIS	dolichyl-di-phosphooligosaccharide-protein glycotransferase (oli	0,96622	1,1847	1,1987	0,42588881	0,09573237	0,13380921
Q9SAU2	AT5G61410.2	1	GVNPWIEVDGGVTPANAYK	D-ribulose-5-phosphate-3-epimerase	0,59215	0,4356	0,73563	0,05198642	0,00826368	0,12188209
Q6AWX0	AT5G17010.3	2	GLTLEEIEAK,VSILLGLLK	D-xylose-proton symporter-like 2	1,0265	2,5281	2,2199	0,271836	0,07731179	0,05864327
Q9SE83	AT1G10290.1	1	LGEQLVNSAEGTR	dynamin-2A	0,72229	0,81006	1,1215	0,11940032	0,15530305	0,28080724
Q9LQ55	AT1G59610.1	1	LGEQLVSSAEGTR	dynamin-2B	1,4219	1,2981	0,91295	0,10989013	0,12251207	0,31667451

P42697	AT5G42080.1	8	GTDAVEILEGR,LGKPIAADAGGK,YNDSYLR,IPGIQSLINK,LFDQIFK,VYNVFDNQLPAALK,LIESSIVSIR,SIV	dynamin-related protein 1A	1,2678	1,2418	0,99819	0,42619086	0,43978357	0,38489721
Q8LF21	AT1G14830.1	6	IPSIVALINK,LIDGSISYFK,SIDEINAELDR,SLLNFFYAQVGR,FPTLASDIAAAANEALER	dynamin-related protein 1C	1,2167	1,3811	1,1022	0,31567926	0,29123244	0,49362608
Q9FNX5	AT3G60190.1	3	LALLNYFYSQISK,IPSILSLINK,LIEGALGYFR	dynamin-related protein 1E	1,5274	1,2857	0,84269	0,1468236	0,27906516	0,22026775
Q8S944	AT4G33650.1	1	FIFEELIK	dynamin-related protein 3A	1,1556	1,7957	1,5551	0,29684583	0,02802609	0,06910349
Q8LFT2	AT2G14120.1	1	FIFDELVK	dynamin-related protein 3B	1,4402	2,9325	2,0361	0,10523429	0,00271908	0,0244815
Q9SRU2	AT3G02260.1	1	VLNFLVDLLSVAQSPDLR	E3 ubiquitin-protein ligase UBR4	0,73448	1,8148	2,4709	0,12853533	0,02343683	0,01197314
Q8LCQ9		1	LASGEVFGPDQPIALK	NADP-dependent malate dehydrogenase	0,41902	0,27408	0,65408	0,01466101	0,00105177	0,07421815
Q9C8G5	AT1G30360.1	15	GTFSQLDNLSMANITK,AEAILAATNNRPTNK,GLEPWEGTSLTR,SLVATENSK,EIYPETFYR,LDMIINLLATSL	Early-responsive to dehydration stress protein (ERD4)	0,6549	0,68714	1,0125	0,11940032	0,02686113	0,15775323
Q8LF70		6	IPNLIFDCGATFLLK,DQSTLQYTTVINQR,LGYAVPEIELVLHSK,ISSVNPYTVLESSIYNAFTSEFVK,GYVSSTYC	EDGP precursor	1,0282	1,1931	1,0963	0,12948732	0,21392055	0,23176472
Q9LS47	AT3G18430.2	1	DFVAFLSAFSAK	EF-hand, calcium binding motif-containing protein	0,75617	0,73748	0,97527	0,14224503	0,09801687	0,44098082
Q9M4T7		6	HLVFPIFEFLQER,WIVNLIR,LNLNYEEAER,SLEEAAAPLVSFLLNPNAVQELR,YQIGPDQIEALYQYAK,YLAT	eIF3e	0,58586	1,0652	1,8182	0,04967535	0,34358374	0,03610505
Q8VYP0	AT3G08950.1	1	AAIGGPFSLIR	electron transport SCO1/SenC-like protein	1,6392	4,1141	2,5098	0,06009101	0,00059583	0,01166119
Q9M5P3	AT5G54290.1	1	VSAWINPISGALLLGGGLYTFLDR	cytochrome c-type biogenesis ccda-like chloroplastic protein	2,3778	1,5874	0,66761	0,01470554	0,04460158	0,07919991
Q94AJ8		1	IFDVNPDILR	putative beta-1,3-glucanase	2,3775	3,4004	1,4302	0,01470554	0,00146374	0,09769307
Q9FVT2	AT1G57720.2	3	ALIAAEYAGVK,AAPVAEAPKPAEEEEAPKPK,QTEAVPPVPTK	elongation factor EF-1 gamma subunit	2,0131	2,3243	1,4115	0,01553351	0,00385866	0,25907467
O04487	AT1G09640.1	5	EAPVAEAPK,LAEEEEAPKPK,TPAFLK,QTEAVPPIASK,VPVLETPEGSVFESNAIAR	elongation factor EF-1 gamma subunit	1,888	2,0763	1,1781	0,06347689	0,02159247	0,25750992
Q9SGT4		18	IRPVLTVNK,GVQYLNEIK,FGVVESK,NMSVIAHVDHGK,FTADELR,LLEPVYMVEIQAPEGALGGIYSVLNQK	elongation factor EF-2	1,006	1,2333	1,2515	0,31381465	0,10082244	0,18681848
Q9SI75	AT1G62750.1	2	VALTGDIIALAGLK,VVDSLVPLAEMFQYVSTLR	elongation factor EF-G	0,75371	0,8029	1,0639	0,17134952	0,19576087	0,35550787
Q0WUV8	AT4G02930.1	3	FPGDDIPIIR,VGEEVEILGLR,GSALSALQGTNDEIGR	Elongation factor Tu	1,3398	2,0394	1,5546	0,11399376	0,04359913	0,27044118
P17745	AT4G20360.1	13	EDQVDDAELLELVELEVR,FEAIIYVLK,QTELPFLLAVEDVFSITGR,IVVELIVPVACEQGMR,EHILLAK,TTLTA	Elongation factor Tu	1,1079	0,68169	0,63845	0,42314706	0,04684104	0,07543622
Q8VYV1		1	AGLLSLAEK	At5g08050/F13G24_250	2,3485	1,6306	0,69431	0,01553351	0,03862535	0,09470631
Q8LCP6	AT1G75680.1	3	LALQFFDIQK,ENPMQNEPTTYNNALLVGLLSSLVTTSSTLQSLK,IDVDTPGTEVAAETAAAMASASLVFK	endoglucanase 10	0,84419	0,65328	0,78739	0,22452411	0,03690382	0,1112501
Q38890	AT5G49720.1	2	LAGAQLLLSR,LFLSPGYPYEEILR	endoglucanase 25	1,1239	1,3849	1,2264	0,36417609	0,12693655	0,20110453
Q9C5N2	AT5G25100.1	2	EAQMCNVLGR,HEYEGQWSEK	endomembrane family protein 70	0,8976	1,0263	1,0953	0,3931149	0,17096144	0,24444701
Q9FYQ8	AT5G35160.2	1	YEEAANVAR	endomembrane family protein 70	0,84352	0,9468	1,1224	0,22317613	0,36083543	0,28034618
Q8LDH5		5	YLEELVK,AVSEASSSFGAGYVAGPVTFIFEK,VSVFLPEEVK,VVETYEATSAEVK	endomembrane-associated protein	1,6007	0,92566	0,58216	0,07833955	0,27470657	0,04618817
Q9LM16	AT1G22200.1	1	LAFGDFFPGVVNPLDGVQWNQGK	Endoplasmic reticulum vesicle transporter protein	0,94083	0,88932	0,94525	0,37157152	0,25889002	0,37598399
Q9STX5	AT4G24190.1	5	LADTPCVVVTSK,ETVYEWELLNDVK,GLVDSDTLPLNVSR,APHDLYESYYNSNK,DEAGEYLEESK,ELISNASI	endoplasmin-like protein	1,1242	1,261	1,1843	0,33405095	0,13826204	0,23990571
Q9M2U2	AT3G55360.1	3	DLGAQVSYR,DKPVVLNSK,DPSGAGGYQIPR,ILETFFVHR	enoyl reductase	0,77385	0,72492	0,93263	0,2218635	0,1553022	0,41363758
Q9ZPI6	AT4G29010.1	5	FSGGFDINVFQQVHK,SPMTELLLK,VAVIGGGLMGSGIATALLLSNIR,ASDLDIASVLGMSFPSYR	enoyl-CoA hydratase/3-hydroxyacyl-CoA dehydrogenase	1,642	1,5284	0,93787	0,09856805	0,02777531	0,21383931
Q9ZPI5	AT3G06860.1	9	FLEAGIGR,VAIIGGGLMGSGIATALILSNYPVILK,DVDMVIEAVIENISLK,GLIHVFFSQR,LCDLVGFGVAIATA	enoyl-CoA hydratase/3-hydroxyacyl-CoA dehydrogenase	1,2991	2,1638	1,7794	0,13996266	0,00938781	0,04854766
Q8L860	AT4G32760.1	1	AQLLALTLLETIVK	ENTH/VHS/GAT family protein	1,0825	0,95462	0,9773	0,34220693	0,34357193	0,46344543
Q9LJG3	AT3G14210.1	14	IGPMLNEFAK,ISTSPYGFQFTVFDFYNAVLR,ANPNADASAQQAFVTNVINR,TGNECYELLNDLAK,GVSFAV	epithiospecifier modifier 1	2,1273	1,2371	0,59507	0,02976225	0,074121	0,1072668
P42762	AT5G51070.1	1	TAIAEGLAISIAEASAPGFLLTK	ERD1 protein	1,3595	1,2557	0,92365	0,12948732	0,14192721	0,33655818
Q94II1		2	FFSLLPGEVILASLDGFNK,IVQGENVVAVLASVSDEIQWPLTK	ERD7 protein	1,2577	1,4824	1,2184	0,36317676	0,18669993	0,1545229
Q9ASW5	AT2G18360.1	2	AELLEALVISNK,SSADLLLPTSVK	esterase/lipase domain-containing protein	2,1034	1,1142	0,5297	0,0246647	0,27108039	0,03184562
Q9ZVI9	AT2G38670.1	2	IVANHEAYQK,IVGSCIVGGAAFAVGASFLHLFLK,TEGVSSTDIVGR	ethanolamine-phosphate cytidylyltransferase	3,281	0,95543	0,89287	0,00119159	0,05865764	6,11E-05
Q3E9K8	AT5G05740.2	3	LGVPFFVPSWQIGSFGAITR,LFLLTNPEDDKPVAVVVPR	ethylene-dependent gravitropism-deficient and yellow-green-lik	0,72229	0,82346	1,1241	0,13041656	0,20536785	0,24165941
Q8L9C2		2	QPETTTEAEAPSLTTK,TETLVLEGEAK	ethylene-responsive protein, putative	1,0946	1,0719	1,2615	0,15554632	0,29733638	0,10264234
Q9SIZ2	AT2G40290.1	3	VSEEDIQTCEER,ILVTDPDSVLGPLTR,EVGPDGQEVTK,NAEAAGNEDCPVK	Eukaryotic translation initiation factor 2 subunit 1	0,92666	1,2371	1,3094	0,49571374	0,0631033	0,08325311
Q8RVQ5	AT5G12370.2	1	AIFTCLLDQVSVYITEGLER	exocyst complex component 5	2,7292	0,80673	0,29559	0,00763307	0,1521335	0,00279604
P38666	AT3G53020.1	1	SIVGATLEVIQK	60S ribosomal protein L24-2	0,42956	0,59232	1,3789	0,01630164	0,03224572	0,11454667
Q9ZPY7	AT2G46520.1	1	YCLDNFAAPLTEIFLK	exportin-2	0,68077	0,757	1,112	0,09731746	0,11197347	0,29277568
Q8L7C3		1	DLNVVGFGLIGWLAPSSIPAINGK	expressed protein	3,3713	1,7651	0,52355	0,00342053	0,02611824	0,03075562
O48702		3	SQLGTALTALDSLLQTVPSQVLDK,AVAQYASDDGFSK,SASEEDAGSDDLESSEIK	expressed protein	1,4877	2,3533	1,6299	0,08503666	0,01894149	0,15812838
Q9MAH9		8	LLDDEGQPDEHVIR,FLDNFHVQTK,DSIAVNNQDTK,HVISGILR,TLDEQVDQEEFVR,QWLIQAMGGAPSG	F12M16.12	1,0453	1,0617	1,0381	0,32416414	0,43085806	0,28089052
Q9LN36		1	IPSGEVWGGNPAR	F18O14.34	1,0463	1,1122	1,063	0,3986578	0,27332023	0,36814976
Q9LNS1		1	GFGFVSFR	F1L3.2	0,59394	1,5314	2,5784	0,0523846	0,05301688	0,01034931
Q94BX1		4	SWGESGYLR,CGIAIEPSYPIK,VVTIDSYEDVPTYSEESLK,GVDGTCDQIR	F2G19.31/F2G19.31	1,1858	0,76128	0,642	0,22317613	0,11488251	0,06904298
Q9SF45	AT3G09580.1	1	LFDFVFR	FAD/NAD(P)-binding oxidoreductase domain-containing protein	1,4636	1,2886	0,88044	0,09856805	0,12620998	0,26397186
Q9XEC4	AT4G04460.1	1	FDGILGLGFK	phytepsin	0,4244	0,46383	1,0964	0,01746757	0,01183842	0,34321568
Q9SA85	AT1G30700.1	2	ISSTVTPFPYR,LPDELFIR	FAD-binding and BBE domain-containing protein	0,93945	1,7742	1,8885	0,36922949	0,02569306	0,03184562
Q9FKV0	AT5G44380.1	2	TVTMTYLGQFLGEK,EPIPVIGLK	FAD-binding and BBE domain-containing protein	0,66738	1,2075	1,8093	0,08839531	0,17392991	0,03633011
Q38924	AT2G27190.1	5	FWFFIPPK,NQDGNAVAADSVWLLNR,QAEATVNTYR,EASFGHGLLEIK,TVQYWCENEK	Fe(3+)-Zn(2+) purple acid phosphatase 12	2,2812	2,0361	0,89256	0,01779099	0,01416146	0,28221319
Q9LZX4	AT3G60900.1	1	TFANLLVSSGVIK	fasciclin-like arabinogalactan protein 10	2,4503	0,76031	0,31029	0,01340805	0,1142361	0,00376622
Q9FFH6	AT5G44130.1	3	GGQFVTLIR,FYTLEDLLSVSNPVR	fasciclin-like arabinogalactan protein 13	1,3488	0,86816	0,72379	0,42221674	0,28606641	0,26059071
Q9SU13	AT4G12730.1	3	VAFGVQDDDSK,SGNGAVNTLATEGNNK,NILSLHVLVDYFGTK,TFQDTVDGGLTVFCPSDSAVGK	fasciclin-like arabinogalactan protein 2	1,3271	0,98278	0,7797	0,49684902	0,41256047	0,42500305
Q9SIL7	AT2G20520.1	2	QQFPLAVYVVDSVLLPEELFGTK,YYSLSDLLLASNPVR	fasciclin-like arabinogalactan protein 6	0,6975	0,61647	0,87448	0,11399376	0,0428636	0,26466223
Q9SJ81	AT2G04780.2	2	VSSSVFSTDPVAVYQVNR,FTDVSGTVR	fasciclin-like arabinogalactan protein 7	0,60293	0,7852	1,2978	0,04661118	0,13025477	0,1183661
Q9SRT9	AT3G02230.1	1	GIFWQEDIIPFFQSAK	reversibly glycosylated polypeptide 1	2,2652	1,6689	0,73675	0,01854757	0,03426426	0,12230716
B3LF88	AT1G03870.1	2	YYSMDDLLSVSNPVR,QQRPLAVYVVDMVLLPGEMFGEHK	fasciclin-like arabinogalactan protein 9	0,82205	0,68613	0,87566	0,17831287	0,06174874	0,21547333
Q7XJJ7	AT5G64440.1	13	LFVNLLEAPLIGSLIVDYLK,VVEIVVPELEEMR,IISIIEEFGYDKPPTPFLIR,KPAIFYDILNTN,TSFAIFR,AAHVISI	fatty acid amide hydrolase	1,4672	1,4897	1,0154	0,09779852	0,06123844	0,46175807
Q9LVM3	AT5G58250.1	2	VLYDSFEATSLDEALASNPTTLEFDKPK,YEPGWWDTFLPK	uncharacterized protein	2,2543	1,8875	0,8373	0,01870364	0,01981269	0,20945838
Q9ZNZ7	AT5G04140.2	4	ISGLTFDELAR,VTAPAGELQLK,VEPAVAIVQR,FPGVPGDLVNYFLYVAEEVR,IPTVTIEQAQK,SGSQLLVLSI	ferredoxin-dependent glutamate synthase 1	0,66153	1,0624	1,4523	0,04901431	0,06404894	0,23920679
Q9FKW6	AT5G66190.1	11	MAEYAEELWELLK,GIDDIMVSLAAK,LVYTNDGGEIVK,DGIDWLEYK,LYSIASSAIGDFGDSK,MFFEEHEDY	ferredoxin--NADP+ reductase	1,2376	1,2374	1,0407	0,2164228	0,07394455	0,2064367
Q8W493	AT1G20020.1	8	LYSIASSALGDLGNSETVSLCVK,APENFR,ITADDAPGETWHMVFSHQGEIPYR,DPNATVIMLATGTGIAPFR	ferredoxin--NADP+ reductase	1,1975	1,256	1,0663	0,23142653	0,12329048	0,30776669
Q39161	AT2G15620.1	2	VTELVPLVAEILIK	Ferredoxin--nitrite reductase	1,4542	1,1978	0,82364	0,10072273	0,18127293	0,19205589
Q9C4Z9		2	LVSETADLQNSIK,VQQELAEVVGLDR	ferulate-5-hydroxylase	1,1411	1,443	1,2646	0,26790162	0,07230791	0,15800496
Q9LSQ5	AT5G54500.1	4	SESPIITPNELAEADGFVFGFPTR,QPTELELQQAFHQGQYIASITK,GAASVEGVEAK	flavodoxin-like quinone reductase 1	1,3822	1,4108	1,0396	0,11940032	0,10019382	0,44115476
P52839	AT2G03760.1	1	ALVFALLNR	flavonol sulfotransferase-like protein	4,2366	10,244	2,418	0,00119159	2,88E-06	0,01330089
Q9S7E4	AT5G14780.1	2	NPNFLGCVENALGIR,ILILMR	Formate dehydrogenase	1,4383	1,062	0,74803	0,18621086	0,45242566	0,16012956
Q9SPK5	AT1G50480.1	11	FLYPLDIGIK,SFSDIMFR,SYGASGVEYSDQAEK,IFHETSQSDK,LVGPGGFVVTEAGFGSDIGTEK,STTTVGLC	Formate--tetrahydrofolate ligase	1,1095	2,378	2,1433	0,30717238	0,00692025	0,02006469
P25851	AT3G54050.2	1	SGYELQTLTGWLLR	fructose-1,6-bisphosphatase	1,1136	1,0133	0,90989	0,30079473	0,46084219	0,31160325
P29512	AT5G62700.1	1	YTGDSDLQLER	tubulin beta-2/beta-3 chain	0,43921	0,67194	1,5186	0,01870364	0,03089351	0,13380921
O04616	AT4G01150.1	3	ELAEDIESLK,VMELVGLGYTGWFVYR,ASSEETSSIDTNELITDLK	uncharacterized protein	2,3716	3,5541	1,4952	0,01870364	0,00167679	0,08303606
Q9LF98	AT3G52930.1	6	VSPEVIAEHTVR,LASINVENVETNR,IGENEPSEHSIHENAYGLAR,AAQEALYVR,GTVELAGTDGETTTQGLD	fructose-bisphosphate aldolase, class I	1,2556	1,4509	1,1605	0,10942681	0,02186864	0,14562255
Q3E716	AT4G38970.2	3	GLVPLVGSNNESWCQGLDGLSSR,VLLLR	fructose-bisphosphate aldolase, class I	1,1479	0,83035	0,75806	0,46382572	0,29558239	0,29315211
Q9ZU52	AT2G01140.1	4	ASPETVADFTLTMLK,GLSPLAGSNEESWCQGLDGLASR,YSAEGENEDAK,GILAIDESNATCGK	fructose-bisphosphate aldolase, class I	1,0394	2,2064	2,1227	0,41088283	0,01011811	0,02060502
Q9SJU4	AT2G21330.1	5	ATPEQVASYTLK,LASIGLENTEANR,YAAISQDSGLVPIVEPEIMLDGEHGIDR,AAQDILLAR,TIASPGHGIMA	fructose-bisphosphate aldolase, class I	0,90014	0,8694	0,9442	0,33500193	0,49591147	0,32834711
Q944G9	AT4G38970.1	2	TWGGRPENVNAAQTTLLAR	fructose-bisphosphate aldolase, class I	0,86632	0,84276	0,90484	0,33099788	0,19026959	0,32507871
O65581	AT4G26530.2	2	VAPEVIAEYTVTALR,IGATEPSELSIQENAK	fructose-bisphosphate aldolase, class I	0,56419	0,33584	0,59527	0,04550156	0,00269595	0,04960883
Q42139	AT4G32260.1	4	AEIAAALNK,ALDSQIAALSEDIVK,ELDEQAAAVMR,ETQVEVEEK	F-type H+-transporting ATPase subunit b	1,0479	1,3295	1,2538	0,31578971	0,08945559	0,16326125
Q9SSS9	AT4G09650.1	6	NDTMELTVTDIEK,TVIDASLVAGFTIR,QVIDDIVK,QLEDIASQLELGEIQLAT,LEQVFSDPQVLNFFANPTITV	F-type H+-transporting ATPase subunit delta	1,3448	1,6243	1,1983	0,12245672	0,01183842	0,07124351
Q1KPV0	AT1G03160.1	3	LLSSVEALVR,LSSLDLAISYVFK,ADLLVFVLSADRPLTESEVAFLR	FZO-like protein	1,6149	1,3108	0,8117	0,06347689	0,11648072	0,1782611

Q9C6B3	AT1G47260.1	5	GDVNNISVGSGTNIQDNTLVHVAK,NYINLAQIHASENSK,SFEQIEVER,VGSLLQGSHR,IEEHLSR	gamma carbonic anhydrase 2	0,77765	0,79717	1,0251	0,15926174	0,14254923	0,44115476
Q94AU7	AT5G66510.1	2	DTEYDSVLDDLTLPENVPK,VFFSSSAVEYSNLAQAHATENAK	gamma carbonic anhydrase 3	0,71617	0,69674	0,97287	0,11793516	0,07412095	0,43547164
Q9SMN1	AT3G48680.1	1	SQVTPSPDR	gamma carbonic anhydrase-like 2	1,3225	1,3337	1,0084	0,14224503	0,10717064	0,47972522
Q9SPE5	AT4G20410.1	2	AYLSAIILYLYAHDLQQAEK,LSIWNEVADFYR	Gamma-soluble NSF attachment protein	1,398	1,6364	1,1963	0,11399376	0,04240089	0,26059071
Q8VZB4		1	VSGVTSEGETAK	GDP dissociation inhibitor	1,6493	3,591	2,1773	0,05897859	0,00113564	0,0192438
Q8LBY8		5	VSGVTSEGETAK,VLIHTDVTK,AVDGSYVFVQGK,VEFDEEGK,SPLMGIFEK,VVCDPSYLTNK,THDGMDLR	GDP dissociation inhibitor	1,0878	1,4683	1,378	0,3146579	0,15521562	0,12117762
O81816	AT4G38540.1	2	YRPPWELLWSNITK,VLVGCDGVYSVVGK	FAD/NAD(P)-binding oxidoreductase family protein	2,2396	2,9323	1,3093	0,01908925	0,00271908	0,13799311
Q9C7N4	AT1G29670.1	12	SNYFPYGIDFGGPTGR,SYNAQSASDAYPMDISR,YSTQLNALYNYGAR,NAGQITCLPGQRPCR,QILSGVNY/	GDSL esterase/lipase	1,4577	1,1983	0,81256	0,14266898	0,47687266	0,13699913
Q7XA74	AT1G54030.1	7	QASGNVQECVK,NVCAKPEEYIFFDGK,LLQLLVELSR,VDASFPPYGVTVGQATGR,FAFQNLAPLGCLPAVK,'	GDSL esterase/lipase	0,96082	1,2768	1,3317	0,39793891	0,10864464	0,11012598
Q8W4H8	AT1G54010.1	14	NDISLLYSSGASK,TAPASAPFQFTVFDFYNAILTR,QEFNTGMDQCYEK,TLLPQTFWPYGK,GASFAVADATLI	GDSL esterase/lipase	0,89641	0,98918	1,1231	0,2138852	0,16604258	0,08680049
Q9LJP2	AT3G14220.1	7	LLVAITVPLLYR,NAYGCGYSNVHAK,WSDGHIVPDFVGR,FAVQNLGPLGCLPIVR,EPIPPVLDPK,INEPSLHG	GDSL esterase/lipase	0,85432	1,4592	1,677	0,21322366	0,13656973	0,07518436
Q1H583	AT1G54000.1	13	GASFAVEGATLLGAPVESMTLNQQVK,QEYNTGMDQCYEK,TLVAQGFWPYGK,IGPMLNEMAR,FSDGLIT	GDSL esterase/lipase	0,62792	0,87604	1,4169	0,07846703	0,36514363	0,05824542
Q9FIA1	AT5G55050.1	2	YGHPTEAAAR	GDSL esterase/lipase	0,5278	1,129	2,1391	0,03655796	0,25211857	0,02008934
Q9SZW7	AT4G30140.1	3	IYLTQLYVLGAR,NVALFGIGK,VNYLPYGIDYFQGPTGR	GDSL esterase/lipase	0,43786	1,0954	2,5194	0,03514917	0,08577227	0,00847069
O65787	AT2G24180.1	4	FVDSNVETK,FAAADIFPGFGWILDR,SFFYDGLGLGFTK,YGPVITVYLGSVR,GIVTYDDLEALVYMK	cytochrome P450 71B6	2,0196	3,58	1,745	0,02011774	0,00183813	0,08303118
Q9CA67	AT1G74470.1	10	MIDEGDLR,VEAHPIPEHPRPR,VAVIGGGPAGGAAAETLAQGGIETILIER,SIDAGDYDYAIAFQER,FQLATR,	geranylgeranyl diphosphate reductase	1,8628	1,2978	0,71195	0,01908925	0,07348877	0,06910349
Q9FMA8	AT5G38940.1	3	APAVAFAALSSQNPGVITIANTVFGANPAINPTILAK,IDYAVNGQNPPHTHPR	germin-like protein subfamily 1 member 11	1,1394	0,82984	0,75652	0,34845914	0,14624026	0,24216816
P94072	AT5G20630.1	4	GPQSPSGYSCK,GDSMVFPQGLLHFQLNSGK,AAVTPAFAPAYAGINGLGVSLAR	germin-like protein subfamily 3 member 3	0,80519	0,65081	0,83772	0,13416284	0,04414617	0,21796349
P55229	AT5G19220.1	1	EILLNLLR	glucose-1-phosphate adenylyltransferase large subunit 1	1,1038	0,85384	0,77358	0,31342308	0,20480329	0,14531065
P55228	AT5G48300.1	1	DVMLDLLR	glucose-1-phosphate adenylyltransferase small subunit	0,9766	1,3933	1,4055	0,44740793	0,12620998	0,13576487
Q9M5A9	AT5G54800.1	3	SEPHPIGDDAAAAETK,TPVQPVNALGAAIAILGTFLYSQAK,TDFDFWK	glucose-6-phosphate/phosphate translocator 1	1,5285	3,733	2,2571	0,1468236	0,01651631	0,07848598
Q8LB04	AT5G46110.1	6	FISDLFWVGMFYHLYNQLATNTLER,LLNHGFADAIAK,VGFLAK,VAPLTHAVGNVLK,APIDSNLLK,ISTQTG	Glucose-6-phosphate/phosphate translocator-like protein	0,77964	1,0728	1,3244	0,18561901	0,26738732	0,29277568
Q42521	AT5G17330.1	1	ELDELPSR	glutamate decarboxylase 1	0,92183	1,0421	1,1304	0,33667925	0,38883617	0,27044118
Q38946	AT5G07440.2	4	TMCHTHSCNLR,TAVADIPYGGAK,FIVEAANHPTDPDADEILSK,GVVFATEALLAEYGK	glutamate dehydrogenase 2	0,67733	0,60603	0,92321	0,17681787	0,12530114	0,43513114
Q9LR30	AT1G23310.1	3	NVVCNFTEGAMYSFPQIR,ALDYDTLNENVK,QVVALCQAPFLLDDPNVGMLFPADAIAR	glutamate:glyoxylate aminotransferase	0,62441	0,60725	0,85227	0,08686679	0,02346359	0,19895015
Q42522	AT3G48730.1	1	DNGALLIFDEVMTGFR	glutamate-1-semialdehyde 2,1-aminomutase 2	1,4579	1,7692	1,2135	0,09951543	0,02592086	0,19256419
P42799	AT5G63570.1	1	SVGGQPVLIDSVK	glutamate-1-semialdehyde-2,1-aminomutase	2,1919	1,4838	0,67694	0,02094976	0,06237995	0,08325311
B9DGD1	AT5G35630.3	17	HETASIDQFSWGVANR,VSGEVPWFGIEQEYTLLQQNVK,WPLGWPVGAFPGPQGPYYCGVGADK,GGNN	glutamine synthetase	0,71733	0,51192	0,71924	0,24130467	0,05301425	0,13576487
Q9SUR3	AT4G23630.1	1	AMIELK	reticulon-like protein B1	0,45225	0,40301	0,89111	0,02012287	0,00593853	0,28053259
Q0WWL2		2	HAAAEIFANPDVIAEVPWYGIEQEYTLLQK	glutamine synthetase like protein	1,15	2,1374	1,8586	0,25753301	0,01162354	0,03345105
P48641	AT3G24170.3	1	VISSHGEEFVADVVLFATGR	glutathione reductase, cytosolic	0,80612	0,91953	1,1407	0,18621086	0,30624202	0,25907467
Q9LIS3	AT3G23820.1	1	NPQSYIASNIAGFVNLLEVAK	UDP-D-glucuronate 4-epimerase 6	0,45798	0,644	1,4062	0,02122885	0,04945274	0,10547518
Q94BY2	AT1G65820.1	3	YNVPYPTLYAIESENK,LFNCVQR,AAITEFLPK	glutathione S-transferase	1,2126	1,4734	1,2311	0,23630779	0,24156639	0,43386879
Q9LTB7		1	TDWDSVIVSEAK	Lil3 protein	2,1824	1,88	0,86141	0,02122885	0,02016917	0,23990571
Q9FWR4	AT1G19570.1	3	TPAEFASVGSNIFGTFGTFLK,WVTDSDVIVGILEEK,VSAVDLSLAPK	glutathione S-transferase DHAR1	1,4996	1,3318	0,96805	0,17134952	0,05225261	0,19116842
P42761	AT2G30870.1	5	HVSAWWDK,IPVLVDGDYK,VLTIYAPLFASSK,NEYLAGDFVSLADLAHLPFTEYLVGPIGK,LAEVLDVYEAQL	glutathione S-transferase ERD13	1,3414	1,4387	1,0387	0,05241001	0,02515116	0,37267026
Q9FHX0	AT5G42150.1	1	AALYDAAETWVDALK	Glutathione S-transferase family protein	1,5464	2,8547	1,846	0,07677157	0,00303049	0,03423451
P46422	AT4G02520.1	3	LAFEQIFK,YENQGTNLLQTDSK,SIYGLTTDEAVVAEEEAK	glutathione S-transferase PM24	2,6651	1,183	0,427	0,00603378	0,15817724	0,01197314
Q9M9F4	AT1G78370.1	3		glutathione S-transferase TAU 20	1,3829	1,5704	1,1356	0,11940032	0,04684104	0,26397186
Q9LPW0	AT1G12900.1	2	YDSTLGIFDADVKPSGDSALSVDGK,VLITAPGK	glyceraldehyde-3-phosphate dehydrogenase (NADP+) (phosphor	0,85003	0,8691	1,0194	0,2378156	0,32795148	0,16416895
P25856	AT3G26650.1	6	DSPLDIIAINDTGGVK,NPSLLPWK,IIQVVSNR,VIITAPGK,YDSTLGIFDADVKPSGETAISVDGK	glyceraldehyde-3-phosphate dehydrogenase A	0,67593	0,52109	0,69647	0,15240911	0,04253299	0,1763451
P25857	AT1G42970.1	11	AVSLVLPQLK,VIITAPAK,YDSMLGTFK,GILDVCDAPLVSVDFR,DSPLEVVVLNDSGGVK,VVDLAHLVASK,I\	glyceraldehyde-3-phosphate dehydrogenase B	0,65349	0,52935	0,72352	0,1258008	0,02686113	0,15236678
P25858	AT3G04120.1	10	AASFNIIPSSTGAAK,FGIVEGLMTTVHSITATQK,YDSVHGQWK,IGINGFGR,DAPMFVVGVNEHEYK,TLLFG	glyceraldehyde-3-phosphate dehydrogenase, cytosolic	1,4285	0,90749	0,5735	0,10942681	0,1080633	0,03164068
Q9LMM0	AT1G01610.1	1	TALDPIIIAIALGR,VIGGVLGFECTELTR	glycerol-3-phosphate acyltransferase	0,68428	0,57311	0,80433	0,11399376	0,02592086	0,16356589
Q8GWG0	AT5G60620.1	3	IFVDAFWNSR,DLLDISPTLTEAAGAIVDDSFTR	glycerol-3-phosphate acyltransferase 9	1,3927	1,9083	1,3703	0,11867247	0,01888222	0,11731952
Q9SS48	AT3G10370.1	6	LIHGGVR,SMAEDAVDAAIK,LGDPTASVPSR,VVEILASEHK,VVFMLPWLGR,LNVGLACTAALAGAAVLNH/	glycerol-3-phosphate dehydrogenase SDP6	1,0493	1,4952	1,4249	0,3931149	0,05998603	0,09913303
Q9M1J1	AT3G57090.1	2	GIAMLEASLESSAPPLEDR,LYLLAVGYYR	protein BIGYIN1	2,1494	2,4187	1,1253	0,02311352	0,00639157	0,27726892
Q9SLV3	AT1G58270.1	8	FLSFADIK,YQISQETEAQR,TRPPNSYCVK,NWALK,DVYAEITFLAYK,VIDQIQSNNFEK,DPIFEWR,FLDSYTS	TRAF-like protein	0,53874	0,42752	0,80081	0,02311352	0,00354507	0,15554888
Q3EBX0	AT2G21660.2	1	GFGFVTFK	glycine-rich RNA-binding protein 7	1,2684	2,3498	1,8526	0,16916149	0,00738458	0,03363603
O22797	AT2G33470.2	1	AMDFLVELFR	glycolipid transfer protein 1	0,78776	1,2912	1,6391	0,16885097	0,12530114	0,0555293
Q9ZVL0	AT1G54860.1	1	LTNYQDILSECK	Glycoprotein membrane precursor GPI-anchored	0,34698	0,87906	2,5335	0,00649404	0,24236245	0,01108888
Q8W112	AT5G20950.2	10	GFVISDWQGIDR,IGQMVQIER,NTVAPTTQVVYSQNPDANFVK,ELVTGFLK,FTMGLFEEPLADLSFANQLG	Glycosyl hydrolase family protein	0,6219	0,92197	1,5215	0,09951543	0,22648565	0,15741367
Q9C7F7	AT1G27950.1	1	VTLCLDFATGK	glycosylphosphatidylinositol-anchored lipid protein transfer 1	0,46123	0,74006	1,6045	0,02208201	0,099675	0,06026399
Q9LTR9	AT5G57020.1	1	SALKPAELGLVLL,DIGDTSLPEGPIEPATPLSEVK	glycylpeptide N-tetradecanoyltransferase 1	0,8491	1,1607	1,3914	0,18917409	0,17874159	0,08303118
Q0WWW1		2	LEFLMFPR,LFYIPSFK	glycyl-tRNA synthetase	0,91153	1,1132	1,2212	0,32084694	0,27199465	0,18698463
O23627	AT1G29880.1	2	AGDEQLNLFR,LFYIPSFK	glycyl-tRNA synthetase 1	1,1839	1,418	1,1978	0,22469517	0,07812003	0,20655705
Q84L08	AT5G19980.1	1	SLTPLLVAIADTVFR	golgi nucleotide sugar transporter 4	1,6212	4,6559	2,5732	0,13337707	0,00243954	0,01568383
Q9FMH8	AT5G43060.1	3	WGESGYIK,VVTIDSYEDVPENSEASLK,NGGIDTEADYPYK	Granulin repeat cysteine protease family protein	0,95676	1,2166	1,2716	0,40082814	0,16767353	0,15599286
Q9LT78	AT3G19390.1	2	VVTIDGYEDVPQNDEK,ALANQPISVAIEAGGR	Granulin repeat cysteine protease family protein	0,87942	0,96799	1,1007	0,27231812	0,40965488	0,30883009
Q9LK96	AT3G15090.1	3	FGGPEVFELR,VGQEVFGALHPTALR,IGINFLR,YGFVVGLPLATSLLMK	GroES-like zinc-binding alcohol dehydrogenase family protein	0,78626	1,3722	1,7731	0,13996266	0,10019382	0,03772105
Q9LXZ4	AT3G56460.1	4	ELLSWLSR,LGDPTATNPGSPESPVEVSK,YQEKPPLPFIPGSDYSGIVDAIGPAVTK,VIATSLNYANYLQILGK	GroES-like zinc-binding alcohol dehydrogenase-like protein	1,2411	1,5367	1,2382	0,18615349	0,05241017	0,17419534
Q9ZR12	AT4G03190.1	1	LNPAVTLDGLVSLLR	GRR1-like protein 1	0,71555	1,1208	1,5663	0,11756599	0,26260914	0,0680051
Q8H156	AT5G55190.1	4	LVIVGDGGTGK,VCENIPIVLCGNK,SNYNFEKPFLYLAR,NLQYYEISAK	GTP-binding nuclear protein Ran-3	1,1582	1,1225	0,98126	0,30314377	0,13614272	0,26061733
Q9SA73	AT1G30580.1	3	TGFSAINLIYFFTAGPDEVK,TYVVQDGDIIFFK	GTP-binding protein	1,3184	2,1388	1,6223	0,14408762	0,01161912	0,05824542
Q42067		1	ILFLGIDNAGK	GTP-binding protein (Fragment)	1,0678	1,1138	1,0431	0,36417609	0,27146028	0,40522387
Q9FNM5	AT5G08650.1	1	EGIGITEILDAIVQR	GTP-binding protein LepA	0,6245	0,95416	1,5279	0,06470951	0,37805955	0,07434431
O24456	AT1G18080.1	13	LWDLAAGVSTR,DGVVLLWDLAEGK,AYGVAQR,AHTDMVTAIATPIDNADIIVSASR,STLAGHTGYVSTVA\	guanine nucleotide-binding protein subunit beta-like protein	1,1627	1,3417	1,1867	0,45852089	0,04414617	0,05864327
Q9LXK4		3	GEIGIFFPIIVLR,NPASVAQFLR,YAFLTSLVR	guanine nucleotide-exchange-like protein	0,87662	1,1571	1,3236	0,11810843	0,38397552	0,12852101
P20649	AT2G18960.1	8	AWASLFDNR,EDVNIFPEK,ESPGGPWEFVGLLPLFDPPR,TALTYIDSDGNWHR,GAPEQILDLANARPDLR	H(+)-ATPase 1	1,2499	1,6783	1,4673	0,43781083	0,43293578	0,49431307
Q53XH7	AT5G62670.1	2	ETVDLENVPIEEVFESLR,SLAVAYQEVPEGTK	H(+)-ATPase 11	1,2527	0,69216	0,55255	0,17831287	0,07196559	0,03633011
Q9FNC4	AT5G44020.1	3	FEEWQNSGK,TWLTSLGYR,SATVENLIEAGYHSWSNLLLR	HAD superfamily, subfamily IIIB acid phosphatase	1,1071	0,82673	0,75372	0,29409355	0,2028412	0,13614308
Q8VZ10	AT1G56500.1	8	QLLNSPWDVCFEPVNEK,ILGVPTSECVVIEDALAGVQAAQAANMR,ALDLQTGGSR,SPSFTQNQGEGR,QT	haloacid dehalogenase-like hydrolase domain-containing protei	0,91443	0,91832	1,0043	0,32505317	0,30531068	0,48838174
Q3E700		1		HD2B (HISTONE DEACETYLASE 2B)	1,3437	1,4035	1,0445	0,13416284	0,08232493	0,40289158
P22953	AT5G02500.1	7	STVHDVVLVGGSTR,EFAAEEISSMVLIK,SINPDEAVAYGAAVQGAILSGEGNEK,IQAGPADKPMIYVEYK,FS	heat shock 70kDa protein 1/8	1,0502	1,078	1,0363	0,33397761	0,13019327	0,22571128
Q9M637	AT5G49910.1	1	VVDWLASTFK	heat shock protein 70-2	1,3364	0,99565	0,745	0,13677514	0,48702348	0,12730146
Q93VK7	AT5G14910.1	1	ALQDIDGVSNLK	heavy metal transport/detoxification domain-containing protein	1,4136	1,2328	0,87215	0,11363801	0,15651457	0,25335726
Q9FF10	AT5G07830.1	1	QTLVGGFYGLLEK	Heparanase-like protein 1	0,64843	1,3202	2,036	0,07772805	0,11216109	0,0244815
Q42525	AT4G29130.1	6	GFSIEEAVGQDVVGALNK,IAALVNDTVGTLAGGR,MAEDAAFFGDTVPSK,AFEEDCATPISK,GLFYALDLGG	hexokinase 1	0,98848	1,2981	1,2592	0,40965549	0,10589214	0,15004595
P93834	AT2G19860.1	2	SHELFDFIVDVLAK,AFEEDCATPIAK,MAEEAAFFGDIVPPK	hexokinase 2	0,8327	1,1576	1,4028	0,20871781	0,21890917	0,1072668
O64644	AT2G45640.1	1	SGGHHTSEDYAVR	histone deacetylase complex subunit SAP18	1,265	1,0089	0,79751	0,17134952	0,47363597	0,16437754
O04551	AT1G27020.1	1	GGSETFLQNVFESILK	uncharacterized protein	2,1419	2,9904	1,3962	0,02336118	0,00264297	0,10811884
P26569	AT2G30620.1	2	TTSSHPTYEEMIK,LLLVNLK	histone H1.2	0,65646	0,66083	1,0067	0,08154038	0,05630528	0,48313394
Q3EBK6	AT2G38170.3	1	DFLSNLQEVILGTK	vacuolar cation/proton exchanger 1	0,46704	0,36764	0,78717	0,02336118	0,00362132	0,1565329
P26568	AT1G06760.1	1	LLLLNLK	histone H1.1	0,46852	0,52658	1,1239	0,02370508	0,0192227	0,27837031

Q9SII0	AT2G38810.3	1	AGIQFPVGR	histone H2A 8	1,0172	1,0919	1,1231	0,09182467	0,08944023	0,34755475
Q9FFC0	AT5G22880.1	1	TPAAEPAAAAEK	histone H2B	0,60159	1,133	1,8991	0,03379681	0,07609794	0,11842417
Q9SR40	AT3G09220.1	4	GVIHYGGASK,NTLAVPVGGWAVIR,LPSFFDTLTAYR,GIFTADFPDQPPVK	laccase 7	2,1287	1,6824	0,79035	0,02379383	0,0325878	0,15788845
O48917	AT4G33030.1	1	FCVQAAVGHPLTVYGK	UDP-sulfoquinovose synthase	2,1263	2,4452	1,15	0,02379383	0,00626693	0,25049101
P59259	AT5G59970.1	6	ISGLIYEETR,DAVTYTEHAR,IFLENVIR,TVTAMDVVYALK,DNIQGITKPAIR	histone H4	0,89978	1,1206	1,2159	0,2218635	0,22657176	0,42700519
Q9LM25	AT1G18260.1	1	LPVTLALASLWLR	Hr.3 like protein	0,79429	1,4188	1,7863	0,17500456	0,0779512	0,0380425
Q8L7U4	AT4G02450.1	1	IFLTVVLADTK	HSP20-like chaperone	0,88513	0,8649	0,97714	0,28236567	0,22043189	0,44412223
Q9C9W5	AT1G68010.1	8	GPVIDEAALVEHLK,YGIAVGNTPGVLTETTAELAASLSLAAAR,GGLYEGWLPHLFVGNLLK,GQTVGVIGAGF	hydroxypyruvate reductase	0,71761	0,71009	0,99437	0,05045175	0,05546255	0,28320703
Q9FM19	AT5G62740.1	2	DSVLGFAVNVPGTTAK,LLLDDVFEQK	Hypersensitive-induced response protein 1	1,4672	1,8397	1,261	0,06099168	0,01461739	0,17416837
Q9CAR7	AT1G69840.7	3	AMNEINAASR,NSVLAFSESVPGTSSK,LDLDSTFEQK	Hypersensitive-induced response protein 2	1,0557	1,2091	1,1454	0,38252051	0,1728863	0,25419492
Q8VZG1	AT2G18730.1	1	YGLGCLEK	diacylglycerol kinase 3	2,1248	1,8355	0,86386	0,02379383	0,02212846	0,24216816
Q9FHM7	AT5G51570.1	1	ASADDAFYELQNPK,EQIQAYVFDVVR	Hypersensitive-induced response protein 4	1,3843	1,6317	1,1947	0,14877313	0,02686113	0,12761338
P15459	AT4G27160.1	1	QEEPVCVCPTLK	seed storage albumin 3	0,47098	0,56721	1,2043	0,02379383	0,02619155	0,20110453
Q0WPB8		1	LEDSDDLLSSLSLLAEDLTFHTPAFTEK	hypothetical protein	1,5932	3,4492	2,165	0,06642235	0,00136455	0,01957823
Q712H0		1	FTSLTTSLSELLK	hypothetical protein	1,2426	1,318	1,0606	0,18555418	0,11310742	0,37115731
Q9FW47		2	GIESLDLSYNQFHLNTIPK,LSGTIPNFLSNFK,LSGTLPANIGALSQLEAFSLEGNR	hypothetical protein	1,225	1,2288	1,0283	0,36304362	0,19026959	0,29405411
Q9C9C7		1	IIIGLYGDDVPAGTAR	hypothetical protein	1,2031	0,90332	0,7508	0,21322366	0,27999526	0,12993117
Q0WLE7		9	YATVIR,VDFAYSFFEK,VGTEAHTAMTEYDR,FQTSLEK,FIDTASIFGHGR,HGSLGFLPR,VLAHTQIR,AGMTH	hypothetical protein	1,1772	1,4341	1,3935	0,17831287	0,06951783	0,03590064
Q9SVP5		1	NSQLGNSIPDTISNIAALLQVLK	hypothetical protein	0,92085	0,66474	0,72187	0,3350995	0,05810662	0,11243933
Q0WL65		1	LLPLLASSLEFGSAAAPALTALLK	hypothetical protein	0,76992	1,359	1,7651	0,15214608	0,09703449	0,04033278
Q0WST9		1	NSQTGSGIFAPPEISGSR	hypothetical protein	0,70198	0,7843	1,1173	0,10942681	0,13220957	0,28646833
Q9M2L9		1	ESITEPVEEDVATQPIR	hypothetical protein	0,59391	0,8389	1,4125	0,0523846	0,18610806	0,10311528
Q0WQ33		2	NAASAIASDEK,LLAHSSPK	hypothetical protein	0,52418	0,76893	1,4669	0,03554246	0,12138972	0,08629145
Q9SL52		1	IELLQLATLLLK	hypothetical protein	0,45257	1,2869	2,8435	0,02012287	0,12693655	0,00652833
P56785		6	SFLWPNFK,FLFDIVR,YIILPLLIIIK,NTLYFISTIK,ILNFWFTK,TVIWEFFQAK	hypothetical protein Arthcp087	0,88332	1,8575	2,1176	0,37844445	0,02119669	0,0277367
Q8VYM2	AT5G43350.1	3	FPAPTYAVNR,VDAGYPPGIGVK,STCHGISAAAGK	inorganic phosphate transporter 1-1	3,1162	1,2511	0,6392	0,03269544	0,21561358	0,04767472
Q96536		1	VSNQIGLDAAVEAAAEFLNK	pyruvate decarboxylase	2,1072	2,9535	1,4016	0,0246647	0,00271908	0,10708804
Q38954	AT3G26570.2	5	ITELTPTR,GFAAEFAAASVVLFASK,IFPIALSQALACGVAGAIVFDR	inorganic phosphate transporter 2-1	0,79185	1,1344	1,0819	0,12948732	0,09507086	0,48836072
Q9ASX8	AT1G27760.1	1	IAAGEALAVIYELGTLEK	Interferon-related developmental regulator domain-containing p	1,0868	2,1895	2,0146	0,33459764	0,01050728	0,02532286
Q9SRZ6	AT1G65930.1	6	LITPFVELDIK,AFADASMNTAYEK,DTYLNTEEFIDAVAAELK,LIDDMVAYALK,LEAACVGTVESGK	isocitrate dehydrogenase	1,453	1,0451	0,73762	0,13687527	0,2634429	0,21160857
Q945K7	AT5G03290.1	1	TDGLFLK	Isocitrate dehydrogenase [NAD] catalytic subunit 5	0,7445	1,0642	1,4294	0,13416284	0,34544696	0,09794415
Q9LQ31	AT1G33790.2	4	IYVQGGQEGIQYVK,TSPPFGLETTSNFVLGK,AAAAVDAIGAYFSR,IFGSGGGVITMLK	jacalin lectin family protein	0,57381	1,5773	2,7489	0,04730835	0,04567457	0,00775371
O04310	AT3G16460.2	31	IIGFHGFAGNHVNSIGAYFVPK,SSTTPVPSTPLK,SLSTQEVITALTFTTNK,SAEFTLAPDEYITALSAYGK,TSYGI	jacalin-like lectin domain-containing protein	0,87881	0,8484	1,1212	0,42165346	0,2622811	0,33321787
O04311	AT3G16450.3	13	QTSAPFGLEAGTAFELK,VYVGQGQDGISAVK,ASELLHQFGVHVMPLTN,TSPPYGLETQK,VEAGGGAGGA:	jacalin-like lectin domain-containing protein	0,75136	0,5958	0,79327	0,13264411	0,09018468	0,4460835
O80950	AT2G39310.3	20	SVEATYQKPNIFSNTAITSLK,FEYVQEDGEVVTTEYGTTNQHPK,IVGFHGQASDVVHSIGVTIVPITTTE,VWD	jacalin-related lectin 22	0,50624	0,73386	1,471	0,01691131	0,05562979	0,07493888
O04313	AT3G16430.2	4	QTSPPFGLEAGTAFELK,IGVHVRPVSN,GGNQWDDGSDHDAVTK,NGQTEQTPLR	jacalin-related lectin 31	0,62107	0,81641	1,3145	0,06347689	0,16177995	0,13614308
Q9ZTZ7	AT1G01790.1	3	GGIGFQAIAEALGLAAVK,VGQIIAQLLSER,AAVAITAIIAGGR	K(+) efflux antiporter 1	0,64198	0,97129	1,4596	0,11940032	0,30606248	0,07814145
O65272	AT4G00630.1	5	SLDLPVYFGDAGSR,IGQIIAQLLSER,ACAAAIALDTPGANYR,GGIGFQAIAEALGLAAIK	K(+) efflux antiporter 2	1,4805	3,2531	2,0477	0,37462917	0,0369508	0,06999134
Q05758	AT3G58610.3	3	APVSLDFETSVFK,NISVVAVCPK,NTVECITGTISR,VSLAGYEEYIVR,GILLGAVHGIVESLFR	ketol-acid reductoisomerase	0,9682	0,87458	0,8834	0,19991482	0,077212	0,23682267
Q8VYC5	AT1G18270.1	1	GSTCIVNAASER	ketose-bisphosphate aldolase class-II-like protein	0,71168	0,72604	1,0202	0,11533731	0,09077271	0,45058148
Q9LMU2	AT1G17860.1	2	LALSDVPLK,SLLTGVNYYILPVIR	kunitz type trypsin and protease inhibitor domain-containing pro	1,1264	1,1955	1,0613	0,28627108	0,18336696	0,37046625
Q683G1		1	LGDYSTWLQK	similar to senescence-associated protein	2,1047	2,102	0,99869	0,0246647	0,012231	0,49515082
Q6NQI8	AT1G13580.3	1	VLPLFAVFFPSIR	LAG1 longevity assurance homolog 3	0,74228	1,6436	2,2143	0,13337707	0,03699437	0,01836847
Q42593	AT1G77490.1	2	TGPGEAGGQSWTVK,FDPPEGIVIENVPEK	L-ascorbate peroxidase	1,2916	0,90789	0,70294	0,15594361	0,28678651	0,09959821
Q42564	AT4G35000.1	5	SGFDGPWTQEPLK,LAWHDAGTYDAQSK,IALDLCEGVK,GESEGLLK,LSELGFNPNSSAGK,TLLEDPEFR	L-ascorbate peroxidase	1,2874	1,7579	1,3889	0,12759718	0,02168291	0,12443244
Q05431	AT1G07890.8	6	LAWHSAGTFDCQSR,NCAPIMVR,YAADEDAFFADYAEAHMK,EGLLQLVSDK,EDKPQPPPEGR,FDAEQAH	L-ascorbate peroxidase	1,13	1,0836	1,0023	0,28995258	0,35214127	0,37333458
Q9M386	AT3G54200.1	5	RPTTTIDSVTVDR,IGFSYDSSSALLNYR,LQASVNPLLLK,GQVIGEAPLPANR,LLSETQLLSDVMAGVIPLNTF\	late embryogenesis abundant hydroxyproline-rich glycoprotein	0,77524	0,71073	1,0164	0,27014962	0,03232666	0,08303118
O80576	AT2G44060.2	4	FSLEETVAILHVR,IPLTLIYDDIK,DFGSALWDMIR,KPDVDIEK	Late embryogenesis abundant protein, group 2	0,95465	0,77031	0,86297	0,37011401	0,17732078	0,15245963
Q9FZI8	AT1G27480.1	2	YGDGDGTVNLASLAALK,TFASGNTLGVPLVNPLLVR	Lecithin-cholesterol acyltransferase-like 1	0,78246	1,0734	1,3719	0,16337716	0,32855467	0,11688919
Q39205		6	AGYWVQTLVGK,AWIEFR,SENNLIFIEFDTFK,DETPFSHGQGLYINPIQFK,RPLIQGSR,TSFTFSITPR	lectin like protein	1,8193	1,2161	0,68576	0,10942681	0,30531068	0,13880363
Q9LK72	AT3G16530.1	4	EFLDISGNHVGVNINSMTSLVAEK,VLNEVLLQNMYAGFAGSMGR,TNDGKPENHILAIEFDTFQNK,DSTITV	legume lectin-like protein	1,7526	2,2208	1,3461	0,09843317	0,00595597	0,04801067
P30184	AT2G24200.2	3	LSSVSALASGIVLGLFEDGR,SGTGFGVATLVEWVQK	leucine aminopeptidase 1	2,3382	1,8632	0,85948	0,06099168	0,03371261	0,44115476
Q944P7	AT4G30920.1	1	AATGFGVATLVEWVQNHSSS	leucine aminopeptidase 3	1,0235	1,2104	1,1826	0,44321714	0,17190195	0,22004005
Q8GZ99	AT5G49760.1	6	CTDNFSEANDVGGGGYGK,LLGFCFDR,SLYDLQELLDTTIIASSGNLK,LPADISFLSELR,AIADFFK,ENPTDHC	leucine-rich repeat protein kinase-like protein	1,3354	1,2373	0,91402	0,23630779	0,44164823	0,25204488
Q9LSI9	AT3G28450.1	3	IAETFDENIR,GDVYGLGVVLLELATGLK,IPVFFSSPSYSSDDFSGNK,FSVANNDLSGR	leucine-rich repeat protein kinase-like protein	1,1168	1,0004	0,89661	0,38247193	0,36514363	0,29237547
Q9LH71	AT3G14840.2	3	LVIQASGLVGPIPSAIGLLGTLTDLR,LYGCCVEGDQLLLVYEYLENNSLAR,LSAISLTYQALCLGK	Leucine-rich repeat transmembrane protein kinase	1,3178	1,0415	0,79032	0,14408762	0,38957674	0,15788845
B9DHS6		1	TPFYGSTLEELGLYK	AT3G01480	2,0934	2,1566	1,0302	0,02516926	0,01117388	0,43108996
Q9FLK9	AT5G61240.1	2	HLDVGNNHLVGTIR,IPAELGTLQNLR	leucine-rich repeat-containing protein	1,0839	1,1166	1,0302	0,33751783	0,26763162	0,43108996
Q9LT39	AT3G20820.1	7	SYFTVLDLSYNNLK,LSAITIADWK,LPFLR,TLDLIGNQISGGIPYDIGR,ALLAFR,LAVLNVADNR,NNLISGVIPS	leucine-rich repeat-containing protein	1,0033	0,59974	0,62172	0,1863095	0,01220392	0,04671641
Q9FW48	AT1G33600.1	1	LYLLNLGDNLLTGTIPLGLANLK	leucine-rich repeat-containing protein	0,95985	0,8211	0,85545	0,40716384	0,16688934	0,23285827
Q9SU56	AT3G47930.1	3	LLALDPLNDVHVAK,NFNQPENLADLEALVK,VTVQAGIR	L-galactono-1,4-lactone dehydrogenase	0,87841	0,88038	1,0022	0,27075063	0,24381661	0,49362608
Q9M320	AT3G61470.1	10	LTGTDVGYPGGLWFDPLGWGSGSPAK,IGILNTPSWYTAGEQEYFTDK,WNVQAEIVHCR,WAMLGAAGIFI	Lhca2 protein	1,2451	1,1622	0,88159	0,11910663	0,12502722	0,34749418
Q9XF85		2	FAMLGVAGILFTDLLR	Lhca5 protein	3,1138	1,1422	0,36683	0,00535041	0,2377417	0,0079484
Q6LAC7		1	LGFVVQHNVTGK	Light harvesting chlorophyll a/b binding protein	0,75594	0,47549	0,62901	0,14224503	0,012231	0,06355894
Q9LMQ2	AT1G15820.1	9	DPAFLK,TAENFANYTGDQGYPGGR,FFDPLGLAGK,SWIPAVK,EAELIHGR,WVDFFNPDSQSVEWATPWS	light harvesting complex photosystem II subunit 6	2,0669	2,1649	1,0023	0,44153264	0,49686901	0,44412411
Q8LB46		1	WGISIANIADFAKPPEK	light induced protein like	1,6147	2,8407	1,7593	0,06347689	0,00310132	0,04077339
Q9S7M0	AT5G54270.1	8	WAMLGAFGCITPEVLQK,GPLENLLDHLDNPVANNAWAFATK,ALEVIHGR,YLGPFSVQTPSYLTGEFPGDY	light-harvesting chlorophyll B-binding protein 3	0,85607	1,0567	1,3443	0,49305322	0,23361516	0,25123305
Q3EAJ6	AT3G54890.3	1	WAMLAVPGILVPEALGYGNWVK	light-harvesting complex I chlorophyll a/b binding protein 1	2,5627	6,08	2,3725	0,0106699	7,42E-05	0,01398742
Q9C5R7	AT3G54890,1	4	YPGGAFDPLGYSK,ESELIHCR,WAMLAVPGILVPEALGYGNWVK	light-harvesting complex I chlorophyll a/b binding protein 1	1,1703	1,9156	1,6369	0,23630779	0	0
Q39142	AT2G34430.1	2	ASKPTGPSGSPWYGSDR	light-harvesting complex II chlorophyll a/b binding protein 1	0,75935	0,7467	0,95367	0,2218635	0,34941796	0,28320703
Q9SAE3	AT1G13090.1	1	EIITGNEVALDLVQVFLH	cytochrome P450 71B28	0,47906	0,3676	0,76733	0,02516926	0,00362132	0,14132648
O65660	AT4G39730.1	3	NLQAWAGLMGPDYNYFER,GNLDIFSGR	Lipase/lipooxygenase, PLAT/LH2 family protein	1,4965	2,6636	2,0277	0,11908981	0,00639157	0,04246869
A8MS68	AT3G16950.2	3	AIGVDILTGFGSVLGPQK,LAVHAHPTLSEVLDELFK,ALAENEGEGIAK	lipoamide dehydrogenase 1	0,79359	0,77207	1,0613	0,25045	0,14624026	0,34085338
P29513	AT1G20010.1	2	YSGDTADLQLER,EVDEQILNIQNK	tubulin beta-5 chain	0,47936	0,37566	0,78367	0,02516926	0,00395658	0,15355836
O04983	AT5G35360.1	2	LILDVEDFK,LLEEAPSPALTAELR,LIVWAPTR,SEAAAAFGNDGCYLEK	biotin carboxylase	0,52901	0,43217	0,83407	0,02590559	0,00626693	0,22571128
Q9SR93	AT3G08930.1	1	QCSAFTANPTSEK	LMBR1-like membrane protein	1,1925	0,95983	0,80491	0,21936801	0,38889733	0,17305111
Q9M028	AT5G01460.1	1	AADALHQEER	LMBR1-like membrane protein	0,68682	0,95595	1,3918	0,10012196	0,3814227	0,11012598
Q9T0A0	AT4G23850.1	5	LLIEDLAELKPTIFCAVPR,EIVDGKPGK,VYSGLQK,GVMISNESIVTLIAGVIR,IILSGAAPLASHVESFLR,VIEECI	long chain acyl-CoA synthetase 4	0,82227	1,0102	1,2467	0,28514415	0,29005356	0,17383183
Q9CAP8	AT1G77590.1	6	GDVTALKPTIMTAVPAILDR,LLASPWTPESGLVTAALK,LLWDVLVFR,QGIDFANFEELCTK,MIPYAAGVIVP	long chain acyl-CoA synthetase 9	1,0275	1,3396	1,2652	0,42754007	0,15574943	0,15627224
Q8W471	AT4G14070.1	1	FLILLWGEK	long-chain acyl-CoA synthetase	1,0633	0,75376	0,70889	0,37103852	0,10971414	0,10386097
Q9SJQ9	AT2G36460.1	2	VAPEVIAEHTVR,LASINVENVESNR	fructose-bisphosphate aldolase, class I	2,0721	3,0965	1,4944	0,02590559	0,00243954	0,07999502
Q8LKS5	AT5G27600.1	1	GTGVTIVSYQK	long-chain acyl-CoA synthetase 7	1,7658	3,0241	1,7126	0,04575845	0,00255677	0,04589544
Q39043	AT5G42020.1	1	VEIESLFDGVDLSEPLTR	Luminal-binding protein 2	1,4051	2,0439	1,5151	0,14551713	0,01655114	0,07919991
Q8LKS6	AT3G05970.1	5	ELGASDPSANR,VVSYSVLLNQGR,GVVLTHANLIANVAGSSFSVK,VDGTVGDYK,EVIDEDGWLHTGDIGLW	long-chain acyl-CoA synthetase 6	2,7547	3,8604	1,3903	0,02590559	0,00325483	0,1237541

Q9LZX1	AT5G01750.2	2	VLLDGSGTPVVTLR,SCVVYAGESDAIVAQMHR	LURP-one-related 15 protein	1,5262	0,87829	0,64234	0,12191578	0,24306048	0,0680739
Q9C942	AT1G52760.1	1	LFTQSFLPLDGEIK	lysophospholipase 2	1,1134	1,5372	1,2775	0,18917409	0,03847704	0,13375719
Q9ZPI1	AT3G11710.1	4	FELFINK,KPEIWVPGQTR,LVGEFLEPTCVNPTFIINQPEIMSPLAK,AVAADDEEMDATQYYENR	Lysyl-tRNA synthetase	1,3428	1,9788	1,4736	0,13416284	0,01634816	0,08427683
Q9C7N2	AT1G29690.1	1	SLALVGYFIPLYDVK	MAC/Perforin domain-containing protein	1,0003	1,7627	1,7622	0,49925283	0,02619155	0,04052356
B3H4K6	AT4G25080.4	2	AYLHSEADVER,IYGETDEVNR	magnesium-protoporphyrin IX methyltransferase	1,2055	1,3071	1,0842	0,21184587	0,11811992	0,33349195
Q9M591	AT3G56940.1	7	EIQESLLTPR,NLNEAEFEALLQEFK,LLAIGETDDASFIK,TNPVVAEIFSLMSR,GLSDFNLALDLGFLTK,TDYNC	magnesium-protoporphyrin IX monomethyl ester [oxidative] cyc	1,8002	1,5221	0,8493	0,03792779	0,06011687	0,16545095
Q9FMT8	AT5G14120.1	1	IGGGYFSELVVR	major facilitator protein	2,0262	0,84896	0,419	0,02873593	0,19911192	0,01392798
P57106	AT5G43330.1	1	TSVGEKPVR	malate dehydrogenase	2,0606	1,9336	0,93839	0,02652238	0,01783933	0,36483812
P93819	AT1G04410.1	3	VQTSSGEKPVR,NGDWSIVQGLPIDEVSR,MELIDAAFPLLK	malate dehydrogenase	1,3409	1,163	0,9127	0,11364212	0,07704558	0,48201906
Q9SN86	AT3G47520.1	8	GVAFANKPAAAAAN,VAVLGAAGGIGQPLSLLIK,TLVEAVAENCPNAFIHIISNPVNSTVPIAAEVLK,DVNVV	malate dehydrogenase	1,132	1,3816	1,2257	0,33405095	0,0523105	0,10264234
Q0WUS4	AT2G22780.1	3	AGAGSATLSMAYAAVEFADACLR,AIVNIISNPVNSTVPIAAEVFK	malate dehydrogenase	0,98054	1,4338	1,4565	0,4586391	0,05810662	0,07421815
Q2V389	AT5G09660.2	10	VAILGAAGGIGQSLSLLMK,QLEDALTGMDLVIIPAGIPR,LLGVTTLDVAR,GDANVVECSFVASQVTELAFFA	malate dehydrogenase	0,75008	1,0163	1,3265	0,16885097	0,19996677	0,08003804
Q9LYG3	AT5G11670.1	3	DAHYLTGLLPPVILSQDVQER,FAESSMYSPVYR,LLIDNVEELLPVVYTPTVGEACQK,ATGQEYAEFLHEFMC	malate dehydrogenase (oxaloacetate-decarboxylating)(NADP+)	1,5132	0,80454	0,56181	0,17691773	0,16898384	0,07464304
Q9ZP06	AT1G53240.1	7	DDLFNINAGIVK,VAILGAAGGIGQPLALLMK,ALEGADLVIIPAGVPR,SEVVGYMGDDNLAK,LNPLVSSLSLY	malate dehydrogenase 1	1,1992	1,5788	1,3166	0,2161792	0,04557805	0,13576487
Q9LW85	AT3G16000.1	3	INLLSLELK,NSAIEELNTR,LTSETQAAADAELISR,DLVTELGR	MAR-binding filament-like protein 1	1,2406	1,1867	0,96275	0,10806312	0,09124049	0,4108775
Q9LVD9	AT3G21690.1	1	DVLSYAFTEGK	mate efflux domain-containing protein	0,6315	0,65652	1,0396	0,06871065	0,05462372	0,4121936
Q9LXQ2	AT3G44100.1	1	ISPDPVVSGAAATFK	MD-2-related lipid recognition domain-containing protein	0,8098	0,65264	0,80593	0,18917409	0,05301425	0,17307293
Q8VZL4	AT4G00290.1	2	SFAELTIGCNLIR,LLPVGFSNEDVPYEK	Mechanosensitive ion channel protein	3,7866	1,6628	0,43912	0,00197437	0,03484347	0,01640344
Q56WN1	AT5G37600.1	1	VFSNPDVAAEVPWYGIEQEYTLLQK	glutamine synthetase cytosolic isozyme 1-1	2,0578	1,7701	0,86021	0,02654178	0,02592086	0,23882268
Q9M2Z4	AT3G48890.1	2	TASAEGLSTNTGEEASAITHDETSR,GQIYDVSQSR	membrane steroid-binding protein 2	1,6254	1,8253	1,0968	0,06494351	0,02907051	0,34243189
Q1ECE0	AT5G54110.1	1	SCYMRPPGGVLAPGESVFATVFK	membrane-associated mannitol-induced protein	1,3883	0,99384	0,71587	0,11932159	0,48179407	0,10811884
O64471	AT2G19080.1	1	DGIVNLDSQLQSLSDYLSLK	metaxin-related protein	0,98771	1,4593	1,4775	0,46816001	0,06833032	0,08325311
P26587	AT1G73190.1	1	ADEATHPDSIR	aquaporin TIP3-1	0,48634	0,63088	1,2972	0,02654178	0,0448421	0,14396051
Q93ZT6	AT5G57870.1	1	GVITLIFDK	MIF4G domain and MA3 domain-containing protein	1,6083	1,744	1,0844	0,06347689	0,02743583	0,33339955
O81062	AT2G03120.1	2	FANLALAGLTLAPLVVR	minor histocompatibility antigen H13	0,89579	1,753	1,8122	0,46447374	0,02366639	0,03547632
Q8RXF8	AT5G27540.2	13	LPIEFFPDGIPVTIVDTSSRPEDR,EIETCIECSALK,DDLDSSPISIQESTR,LSEYWLPELR,APDQSFELTNAAIDFL	MIRO-related GTP-ase 1	1,456	2,6264	1,7771	0,2492873	0,01740184	0,04946517
Q93XM7	AT5G46800.1	5	EVPGNATMFAAYEAFK,GMGAPLATVAAFNAVLFTVR,SVLQVDDYK,LQAQGALAGASTTSSVVAAVK	mitochondrial carnitine/acylcarnitine carrier-like protein	1,0955	1,3484	1,195	0,38022547	0,49591147	0,37519001
Q9SVB2		2	QYQGIVDCVQTIVR,ELSDPENALIGAFAGALTGAVTTPLDVIK	mitochondrial carrier-like protein	1,0286	1,4876	1,4462	0,43049918	0,0616115	0,09238405
Q8L7F2		13	GLWQPFTALLGDNPSIDVK,NDIVLSVTELNK,SVVVTLSSDK,LGELDSYEIEGGETK,EVQFVIVGEK,FHSVVA	mitochondrial F1-ATPase, gamma subunit (ATP3_ARATH)	1,0209	1,527	1,4653	0,39394212	0,09682281	0,09913303
P82873	AT1G27390.1	2	WGGALLELSQFQPIPEAK,LEEALTINPGK	mitochondrial import receptor subunit TOM20-2	1,5126	2,0417	1,3336	0,12948732	0,02947581	0,15788845
Q9FNC9	AT5G43970.1	1	ISNSEIVSQGR	mitochondrial import receptor subunit TOM22-2	1,5387	3,3219	2,1501	0,08215201	0,00271908	0,03043684
Q9LHE5	AT3G20000.1	1	SDNFEGLR	mitochondrial import receptor subunit TOM40-1	0,81619	1,0423	1,277	0,19663579	0,38857216	0,15355836
Q9SRH5	AT3G01280.1	8	DLLYK,VNSAGIASALIQHEWKPK,INAGLSFTK,VCTDSTFLITATVDEAAPGLR,DSTITVGTQHSLDPLTSVK,F	mitochondrial outer membrane protein porin 1	1,117	1,8295	1,5346	0,16637969	0,0133854	0,05825772
Q9SMX3	AT5G15090.2	9	FSVTTYSSTGVAITTTGTNK,VSTDSSLLTTLTFDEPAPGLK,NNNFTADVK,HFNAGFNFTK,ENAITVGTQHAL	mitochondrial outer membrane protein porin 2	1,905	3,2338	1,6846	0,02516926	0,00084721	0,03547632
O04308	AT3G16480.1	2	VITKPLTMATFGDVLNVPSYDSVSK,SAILMNLESR	mitochondrial processing peptidase	0,87751	1,2104	1,3793	0,26998831	0,17190195	0,11454447
Q9C5M0	AT5G19760.1	8	MQADNTLPLAQR,IAPHVMMTWIFLNQITK,FYSGFPVYCVR,ALCGLTAGAIGACVGSPADLALIR,GCGPTV	Mitochondrial substrate carrier family protein	1,1365	1,6619	1,3968	0,28320985	0,07434363	0,15691705
Q9FHX2	AT5G42130.1	2	DGILGLYAGYSATLLR,AIIGAGAGGLAGAFTYVTLLPLDAIK	Mitochondrial substrate carrier family protein	0,5708	0,23012	0,40316	0,04661118	0,00044965	0,01197314
Q9SSK7	AT1G70850.3	2	HGGSGSVVHWHFEYEK,INEEVAHPETLLQFAVEVSK	MLP-like protein 34	1,1709	0,25492	0,21771	0,23630779	0,00074766	0,00046994
Q8GUM2	AT4G37910.1	1	IPAEIASEIETAVSDLR	molecular chaperone DnaK	2,8786	17,504	6,0807	0,00649404	6,16E-08	7,94E-05
Q940V1	AT4G39960.1	1	TCGGQGQVVASTR	molecular chaperone Hsp40/DnaJ family protein	1,4329	1,3229	0,91742	0,09929699	0,08169761	0,41363758
Q9SU40	AT4G12420.2	3	RPLTGPAK,TENLDSWYLGQETYVR,ASGGFGSFVVNPR,STIQVYPGAWSAILISLDNPGAWNLR,DQIGSFFV	Monocopper oxidase-like protein SKU5	0,53843	0,99842	1,827	0,03647136	0,19921999	0,05963097
Q0WUV6	AT3G52880.1	3	EFANQGVQPGELAVISK,VVGAFMEGGSGDENK,TSVPDVYAVGDVATFPLK	monodehydroascorbate reductase (NADH)	1,2129	1,2288	1,0131	0,20541826	0,15860329	0,46697019
Q9LK94	AT3G27820.1	1	TGDFDYLPFFYSR	monodehydroascorbate reductase (NADH)	0,72151	0,74916	1,0383	0,11932159	0,10677205	0,41453009
Q96290	AT1G20840.1	4	EDVTDEMALLVEGLDIGGEK,IAASGPLWSALLEPGVK,VLQQLCGR	monosaccharide-sensing protein 1	0,90248	0,94709	1,0494	0,30758306	0,36137505	0,39188196
Q8LPR8	AT5G22640.1	1	EPEPDPNDPSK	MORN (Membrane Occupation and Recognition Nexus) repeat-c	1,2773	2,2339	1,7489	0,16384434	0,00960744	0,04164865
Q8LGU1	AT3G21250.2	1	ILVLDEATASIDSATDAIIQR	multidrug resistance-associated protein 6	0,91782	0,41841	0,45587	0,33099788	0,00677896	0,01900348
Q8GUN1	AT4G31340.1	2	ALESQIDEK,IASLQTEVSSLQK	myosin heavy chain-related protein	1,1533	1,3148	1,1567	0,42619086	0,19703166	0,19053138
P37702	AT5G26000.1	8	NWITINQLYTVPTR,GFIFGVASSAYQVEGGR,LPEFSETEAALVK,GLNVWDSFTHR,IFFHGWFMGPLTEGK,	myrosinase 1	0,55368	0,66208	1,1958	0,04274392	0,05672548	0,20845254
Q9C5C2	AT5G25980.2	6	FSFAWSR,EFFLGWFMEPLTK,DLDVMEELGVK,ASGLWYQSFLR,GGADLGNGDTTCDSYR,GINEDGINYY	myrosinase 2	1,0368	1,1419	1,175	0,1897806	0,28097411	0,11983455
O80998	AT2G25980.1	1	ASPELYQIGVTVAPITK	myrosinase-binding protein-like protein	0,58113	1,7513	3,0136	0,04823352	0,02686113	0,00499103
Q9SZJ5	AT4G37930.1	13	GFVEEDFAK,SATLFRPK,HEVEEFAK,DFVSAMESSSTIQSEIAK,LDESTGYIDYDQMEK,QLNAPLEEVDPEIA	glycine hydroxymethyltransferase	1,512	1,3343	0,84645	0,02805837	0,01386543	0,48975834
Q8H124	AT2G34460.1	6	VDNFGTVNLVDACR,IVEQLLSR,DLVAEVAVEALLQEESSFK,VFVAGATGQTGK,LAEVIGDDSQAVICATGF	NAD(P)-binding Rossmann-fold-containing protein	1,5136	0,74779	0,49682	0,06347689	0,09293496	0,01867507
Q84WU9	AT5G56900.2	1	EVLAGLLK	zinc finger CCCH domain-containing protein 64	2,0231	1,8241	0,90162	0,02884344	0,02286788	0,29607696
Q949S6	AT1G14345.1	1	VTVISGLTGDDR	NAD(P)-linked oxidoreductase-like protein	1,1968	0,44457	0,37148	0,21729317	0,00931231	0,00830443
Q94A68	AT1G06690.1	4	GIPLASNQVNYSLIYR,AVGVSNYSEK,TPTQIALNWLVAQGNVIPIPGAK,FAALPWR	NAD(P)-linked oxidoreductase-like protein	0,84012	0,99777	1,2214	0,29525989	0,44699663	0,26059071
Q1JPL4	AT4G28220.1	1	AADADNSGTLTMEELEGVVDDIIVR	NADH dehydrogenase	0,63611	0,73825	1,1606	0,07142542	0,09853101	0,23990571
O49313	AT2G33220.1	2	VGENVYNSGR,AILPILQAEEDER	NADH dehydrogenase	0,50406	0,32803	0,65078	0,03198338	0,0024715	0,07277915
Q9FLX7	AT5G52840.1	3	TLEGLIAESK,AVESFTR,AVLIDLYSK	NADH dehydrogenase (ubiquinone) 1 alpha subcomplex 5	0,76352	0,93127	1,2197	0,1468236	0,32795148	0,18816785
Q9LHI0	AT3G12260.1	2	VIDLLIFK,VFDFFR	NADH dehydrogenase (ubiquinone) 1 alpha subcomplex 6	0,67863	0,92521	1,2962	0,10907499	0,30839936	0,13614308
Q9SK66	AT2G20360.1	7	LALVAK,SSVSGIVATVFGATGFLGR,ANVVINLIGR,YIQVSCLGASVSSPSR,FQPVYVVDVAAAIVAALK,VPFF	NADH dehydrogenase (ubiquinone) 1 alpha subcomplex 9	0,80593	0,75951	1,0087	0,13922032	0,0553681	0,25837114
Q42577	AT5G11770.1	1	AAEFVISK	NADH dehydrogenase (ubiquinone) Fe-S protein 7	0,87808	1,1136	1,2682	0,27025302	0,27146028	0,1573503
Q8LAL7	AT5G08530.1	6	EAYAAGLLGK,LKPPFPANAGLYGCPTTVTNVETVAVSPTILR,ASAAYIYIR,LEEIDMLQEVTK,GGWDNLLAIII	NADH dehydrogenase (ubiquinone) flavoprotein 1	0,77271	0,85821	1,1196	0,10959147	0,20400226	0,19205589
O22769	AT4G02580.1	3	DIESALLDHLGVK,EILSYYPSNYK	NADH dehydrogenase (ubiquinone) flavoprotein 2	0,85959	1,3265	1,5545	0,1441488	0,18306382	0,06581376
P93306		6	AAPYDVYDQLDFDVPVGTR,VLFCEITR,LLEFYER,LVDIGTVTAQQAK,APGFAHLQGLDFMSK,LLNCEVPLR	NADH dehydrogenase [ubiquinone] iron-sulfur protein 2	0,75694	0,89698	1,1875	0,1505452	0,18015681	0,33072432
Q95748		5	YFDFASPWEQR,FDTNTDYLFQLLCFLK,ISSVVSLFPSAGWWER,VVSEPIEMTQEFR,VQTSADEVTR,EVWD	NADH dehydrogenase [ubiquinone] iron-sulfur protein 3	0,85884	1,2046	1,3588	0,0599686	0,12510572	0,15691705
Q37165	ATCG01100.1	3	YSFLGGLR,IGPEYAGPLGILQALADGTK	NADH dehydrogenase subunit 1	0,801	1,2644	1,5992	0,18877558	0,19651045	0,08876987
B5TM94		1	NEILAESEFAAPTIIK	NADH dehydrogenase subunit 5	0,66966	0,91042	1,2034	0,0753558	0,04295245	0,43108996
B5TM95		5	VLFCEITR,AAPYDVYDQLDFDVPVGTR,LLEFYER,LVDIGTVTAQQAK,LLNCEVPLR	NADH dehydrogenase subunit 7	0,99822	0,85033	0,85185	0,49599304	0,20024699	0,22896149
P56753	ATCG01110.1	6	VIMLELSR,AIIQYLPYVTR,LSEMTESIK,IRPPGFINLQILPELVK,LIVTLDGEDVVDCEPILGYLHR,IGGIAADLP	NADH dehydrogenase subunit 7	0,83034	0,88987	1,0721	0,27863986	0,35677107	0,35685866
P56755	ATCG01090.1	1	LPVTIQYPYEK	NADH dehydrogenase subunit I	1,1273	1,0843	0,96191	0,28530327	0,31133327	0,4121936
P56754	ATCG00420.1	1	IPSVFWVWK	NADH dehydrogenase subunit J	0,89526	1,1645	1,3328	0,27394655	0,21549831	0,13546214
Q29Q36	AT5G20080.1	2	IFYTVDNPTK,GYFDLLIK	NADH-cytochrome b5 reductase-like protein	1,6684	1,4957	0,95915	0,12948732	0,0693958	0,35646171
P92558		1	GPDVVGSFGLLQPLADGLK	NADH-ubiquinone oxidoreductase chain 1	1,1199	1,4079	1,2572	0,29409355	0,0811332	0,16263148
O05000		2	ISIFANILR,ILTGYEITGAR	NADH-ubiquinone oxidoreductase chain 2	0,99415	2,079	2,0912	0,48554397	0,01280421	0,02145069
Q9FGI6	AT5G37510.2	9	ATETIDVSDAVGSNIR,GSGEEIGTYVEK,VGYVGPPAEFNYDCK,LPYNSIEGVR,LMTSELSGNVIDICPVGALT	NADH-ubiquinone oxidoreductase subunit	0,69943	0,77092	1,1858	0,09231447	0,12263034	0,25419849
Q1WIQ6	AT2G24270.3	6	IPLGVVLAIPPFNYPVNLAVSK,LTVGPPEENSDITAVVSESSANFIEGLVMDAK,DSVTEVVR,VVLVMESVADI	NADP-dependent glyceraldehyde-3-phosphate dehydrogenase	0,98365	0,76359	0,77892	0,4256023	0,17336581	0,23023138
Q96262	AT4G20260.3	6	EPETTTTAPVAEPPKP,YLEELVK,AVSEASSSFGAGYVAGPVTFIFEK,VSVFLPEEVK,VVETYEATSAEVK	plasma-membrane associated cation-binding protein 1	0,73542	0,57636	0,79118	0,03198338	0,00617954	0,17553034
Q3E9U3	AT4G30210.1	1	VVDDILVEQGAQR	NADPH-ferrihemoprotein reductase	0,97143	2,0566	2,1171	0,42854583	0,01354858	0,02060502
Q9LHG9	AT3G12390.1	2	APDLSNVISK,AADGDIVSAIMELTT	Nascent polypeptide-associated complex subunit alpha-like prot	0,78012	1,2494	1,5723	0,18561901	0,04644231	0,02815539
A8MQP6	AT4G10480.2	1	NVLFVISKPDVFK	Nascent polypeptide-associated complex subunit alpha-like prot	0,60204	3,1797	5,2816	0,05682275	0,00225196	0,00018626
Q9S9N6	AT1G15980.1	4	DKPCIGLFSSEEK,FIVIHGIESDSK,NAMQVFEGSLALV,GDADSLLSLEK	NDH-dependent cyclic electron flow 1	0,89073	0,90019	1,0106	0,29028546	0,27470657	0,47278989
B0ZVY9		1	LVGGQLVLLDGGER	NHL3, partial	1,9619	1,2797	0,65225	0,03309547	0,13025477	0,07367035
B0ZVS1		3	LTEFTLDPTNNLR,DLNEDVNSQIYR,LVGGQLVLLDGGER	NHL3, partial	1,3392	1,3977	1,0436	0,13511137	0,08425049	0,40431654
Q3E6U9	AT3G52640.2	2	FLWNFLADK,SFGADSPISGLVALLGAVDALSR	nicastrin	0,89091	1,3858	1,5555	0,29043827	0,08829857	0,06910349

Q05085	AT1G12110.1	3	DQEAGVTSNVFNK,LIGSPMTQVAAVIVAAWR,ASVSGFGSDQFDETEPK	nitrate transporter 1.1	1,086	1,4107	1,2676	0,40185013	0,13221115	0,18794286
C0SVD5	AT3G44310.3	3	YIVEAASK,GAELVLFPEGFIGGYPR,VTIVQSSTVYNDTPATIDK	nitrilase 1	1,3235	2,3276	1,8284	0,11661671	0,00617954	0,04132934
P46010	AT3G44320.1	1	VTIVQSSTVYNDTPATLDK	nitrilase 3	1,6891	1,7803	1,054	0,05198642	0,02535268	0,38205092
Q9SDM9	AT3G16400.2	2	VNDMTSEMITFLSIK,GWTASTTATIDGK	Nitrile-specifier protein 1	1,2309	0,70105	0,57243	0,17831287	0,06809682	0,03547632
O04316	AT3G16410.1	5	YNPEGLILGLTFK,GWTASTTATIGGK,STDVLDSLGAYISLSPTPNLHGK,NGQTEEAPLR,HLYVFDIESR	Nitrile-specifier protein 4	0,5217	1,3511	2,7091	0,04771522	0,09147846	0,01392798
Q9SRQ6	AT3G03520.1	6	SNPLSTSDPNSAQIFFGK,VEETSSGGGSSASPIK,ELVQEFAVCDR,ELNPEIDGVSESEPR,LPNYVVIEPR,DIDI	non-specific phospholipase C3	1,0477	0,96652	0,94785	0,2218635	0,38558762	0,26059071
Q9S816	AT3G03540.1	1	LLIEGFPQK	non-specific phospholipase C5	5,2065	2,6571	0,51034	0,00070441	0,00398083	0,0277367
P29510	AT1G50010.1	3	LVSQVISSLTASLR,SLSIERPTYTNLNR,TVGGGDDAFNTFFSETGAGK	tubulin alpha-2/alpha-4 chain	0,6263	0,58585	0,99211	0,03241734	0,02644344	0,28053259
P93025	AT5G58140.2	1	FWNLFHLQPMR	phototropin 2	0,51024	0,54251	1,0632	0,03313769	0,02168291	0,36785181
Q94JY5		3	NVIHGSDSVESAR,IIGATNPAASEPGTIR,HYEDLSSK	Nucleoside diphosphate kinase 1	1,2296	1,3314	1,4072	0,25875065	0,04449242	0,11328473
Q9LF61	AT5G17630.1	1	NPVRPLNALGSAIAIFGTFLYSQATAK	Nucleotide/sugar transporter family protein	0,33468	1,3667	4,0836	0,00630893	0,09424463	0,0008235
Q9LN62	AT1G19360.1	1	TNPTILPDESINPR	Nucleotide-diphospho-sugar transferase family protein	1,5717	1,406	0,89456	0,07142542	0,08169761	0,28589765
O22224	AT2G41620.1	1	QLLTEILLR	Nup93/Nic96 nucleoporin interacting component-containing pro	0,49235	0,92838	1,8856	0,02843003	0,32362958	0,03184562
Q93VB0	AT2G40660.1	1	EELSTLFAPIPVK	OB-fold nucleic acid binding domain-containing protein	1,0415	1,2443	1,1947	0,40753751	0,14881932	0,20917653
Q9SQR1	AT3G04010.1	1	SDIIQEMTQIVNFLAQNK	O-Glycosyl hydrolases family 17 protein	1,348	0,82964	0,63032	0,17677818	0,16177995	0,07249416
Q9LKA6	AT3G14990.3	2	LVAEVLLDEVAEK,LAATCATAVESR	class I glutamine amidotransferase domain-containing protein	0,51174	0,43749	0,85491	0,03358934	0,00848774	0,23198118
P46312	AT4G30950.1	1	QIGEDLPENVTLK	omega-6 fatty acid desaturase	0,94171	1,3954	1,4817	0,37308258	0,08506077	0,08303118
Q9FEC1	AT1G34000.1	2	ILLSNFGILDLE,EPQKPVPPSQPSSSPPPSPPPQK	one-helix protein 2	1,0921	1,0581	0,96889	0,32790447	0,35677107	0,42700519
Q1H537	AT5G18660.1	1	INQVLPIGGPGK	Rossmann-fold NAD(P)-binding domain-containing protein	1,9528	1,9436	0,99527	0,03358934	0,0174544	0,4876075
Q9CAU9	AT3G04830.1	1	LNLLTSALR	prenylyltransferase-like protein	0,51415	0,44385	0,86328	0,03399722	0,00931231	0,24165941
Q9SYB5	AT1G61790.1	2	LFFCEIEFSK,LAESMAEFVEQR	OST3 and OST6 domain-containing protein	1,0212	1,7347	1,6987	0,44832611	0,02828489	0,04731124
Q9C5J8	AT5G19620.1	2	GLFGIVSDIDTLSGGIVR	outer envelope protein	0,92294	2,0374	1,8343	0,18621086	0,00367439	0,01922562
Q93W02	AT5G24690.1	1	FDLQNTLQK	uncharacterized protein	1,9446	2,8863	1,4843	0,03399722	0,00293771	0,08303118
Q9FFG3	AT5G05520.1	4	VYLSTQDWLK,GYSFISTSQIGGLAPDSR,QATTLQELLK,SSVGAGIVVPTSLFR	outer membrane OMP85 family protein	0,67808	0,91711	1,3525	0,09579231	0,30336054	0,12394942
Q8LGH9	AT2G28900.1	3	NAMLAGAATGAVLSAVGK,PSSTFSGTVSTPK,IGAVGVTK	outer plastid envelope protein 16-1	0,74441	1,4241	1,5765	0,07358752	0,03074754	0,32009677
P23321	AT5G66570.1	6	GDEEELVK,LTYTLDEIEGPFEVASDGSVNFK,IQGVWYGQLE,QLDASGKPDSFTGK,GTGTANQCPTIDGGSE	oxygen-evolving enhancer protein 1-1	1,1301	1,0257	0,88636	0,15727102	0,27108039	0,24255511
Q9S841	AT3G50820.1	6	GTGTANQCPTIDGGSETFSFK,QLEASGKPESFSGK,LTYTLDEIEGPFEVGSDGSVK,IQGVWYGQIE	oxygen-evolving enhancer protein 1-2	1,217	1,0477	0,90705	0,13062538	0,34941796	0,15355836
Q42029	AT1G06680.1	4	HQLITATVNGGK,FEDNFDATSNLNVMVTPTDK,EIEYPGQVLR,SITDYGSPEEFLSQVNYLLGK,TNTDFLPYN	oxygen-evolving enhancer protein 2-1	1,3791	1,1705	0,91567	0,14833887	0,15939987	0,36629974
Q9XFT3	AT4G21280.2	3	YDLNTIISSKPK,YYAETVSALNEVLAK,LFDTIDNLDYAAK,DIINVKPLIDR	oxygen-evolving enhancer protein 3-1	0,98699	0,74524	0,77437	0,36096703	0,17457843	0,27589151
Q8LE12		7	WYEIASFPSR,AVEEGYDISK,SYLWILSR,GFIEGSAYK,TAQMEEETYK,GVWWFK,TPQSDTPPESNTAPEDSI	outer membrane lipoprotein-like	1,8569	2,8972	1,5384	0,03418244	0,00271908	0,07809191
Q3E9P5	AT4G37070.4	3		patatin-like protein	0,9689	0,90433	0,93336	0,4245653	0,28097411	0,35485881
O23179	AT4G37070.2	1	GIIAGVILAFLEK	patatin-like protein	0,67617	0,57939	0,85687	0,09425809	0,02907051	0,23502215
Q9LSP5	AT3G17020.1	3	ALSWAIDNVVR,YALKPDAETLDIVNTAAR	universal stress protein (USP) family protein	1,9398	2,4321	1,2538	0,03418244	0,00636851	0,16437754
Q56Z59	AT1G72160.1	9	QTVEIIIYEK,AEFVPEEK,EGHPVCYNVYGEFQNK,IDELVEEDLVDDLDK,LSDLSNSEK,EALDNHQFTNTPEE\	patellin-3	1,2703	0,9509	0,76682	0,37451204	0,30953756	0,24929874
Q56ZI2	AT1G22530.1	19	VGLTDEPVITDSFK,AFAADQEEETK,SAETIFK,YVAPEVVPVK,NALAELK,VVSEVAVPETEVTAVK,EFTAPP	patellin-4	0,75661	0,71434	0,98453	0,13425726	0,14624026	0,34755475
Q940J8	AT4G19410.1	2	TPLFVINAAFDSWQIK,VVALHGSAK	Pectinacetylesterase family protein	0,98371	1,1653	1,1846	0,45852089	0,21011354	0,21796349
O49006	AT3G14310.1	3	ADATVAADGSGTFK,DITFQNTAGPSK,TDPNQNTGIVIQK,TLISSAITNQETCLDGFSHDDADK,EYSNTGAG	pectinesterase 3	1,5233	1,0212	0,70265	0,24842803	0,49591147	0,2413593
Q8L790		3	DQIGSFYYFPSLGFHK,LASSAGQVDGK,IPVPFPDPAGDTTVLIGDWYK	pectinesterase, putative	1,0113	1,1149	1,1025	0,47056993	0,27017167	0,30767393
Q9LG23	AT1G55890.1	1	YLVGQTTLQQLVDELVK	pentatricopeptide repeat-containing protein	1,3778	2,895	2,1012	0,12161525	0,00290283	0,02145069
Q9LK57	AT3G13160.1	3	LDEAITWYK,LLVDEAVLQEVVDALVK	pentatricopeptide repeat-containing protein	1,2574	2,5782	2,5714	0,11570538	0,00983023	0,06464314
Q8LEZ4	AT1G19520.2	1	VDWIALINQLR	pentatricopeptide repeat-containing protein	1,0219	3,0859	3,0196	0,44693534	0,00243954	0,00499103
P35614	AT3G26618.1	1	YFEEISQDTGK	peptide chain release factor eRF subunit 1	1,0491	1,167	1,1124	0,3931149	0,20850578	0,29273525
Q9M390	AT3G54140.1	3	IFQVIVAAFR,LCSVTQVEELK,AAVESQSDSIK	peptide transporter PTR1	0,72547	0,7068	0,97426	0,12161525	0,07912739	0,43816765
P46032	AT2G02040.1	5	LAYYGIAGNLITYLTTK,AAVISEEESK,ISQVVVASFR,LYAEDGSVDFNGNPPLK	peptide transporter PTR2	1,0182	1,4395	1,3181	0,22317613	0,45179792	0,23559881
Q38900	AT2G16600.1	1	HVVFGQVVEGLNVVR	Peptidyl-prolyl cis-trans isomerase CYP19-1	1,2042	1,9739	1,6391	0,21268017	0,01649625	0,0555293
Q9ASS6	AT5G13120.1	1	IVIGLYGDDVPQTVENFR	Peptidyl-prolyl cis-trans isomerase CYP20-2	1,0231	1,2772	1,3638	0,23630779	0,08707745	0,21692022
P34791	AT3G62030.1	1	IYACGELPLDA,IVMGLFGEVVPK	Peptidyl-prolyl cis-trans isomerase CYP20-3	1,0457	0,91878	0,89238	0,25489378	0,13214134	0,29926809
Q9SSA5	AT3G01480.1	1	TPFYGSTLEELGLYK	peptidyl-prolyl cis-trans isomerase CYP38	1,1169	1,2819	1,1477	0,29634953	0,12933635	0,25206228
Q96520	AT1G71695.1	8	QQAFVVINNLR,LYPNQDPTMNQFFANSLK,YYVDLMNR,DSVVLSGGPDYAVPLGR,SPDVFDNK,DIGLAA	peroxidase 12	1,0535	0,8833	0,84335	0,30717238	0,19358982	0,13614308
Q0WLG9	AT2G38380.1	4	TSDLVALSGGHTFGR,GLIQSDQELFSTPGADTIPLVNQYSSDMSVFFR,GFNVIDR	peroxidase 22	1,2334	1,6168	1,3153	0,19991482	0,02416521	0,08429118
O80912	AT2G38390.1	1	TCPPIFNIIGDTIVNELR	peroxidase 23	0,59411	4,4749	7,9267	0,04518813	0,00036465	1,58E-05
B9DHE0	AT3G32980.1	7	MGNITPLTGTQGQIR,DSLQAFFALANTNLPAPFFTLPQLK,AAVETACPR,TVSCADILTIAAQQAVNLAGGPS	peroxidase 32	1,2455	1,336	1,1992	0,11609178	0,12530114	0,33277448
Q41932	AT4G05180.1	2	YDLNTVISAKPK,LFQTIDNLDYAAR	oxygen-evolving enhancer protein 3-2	0,51641	0,48889	0,94669	0,03424101	0,01384807	0,37789765
Q9LDN9	AT4G08770.1	2	TCPQVFDIATTTIVNALR,TVSCADLLAIAAQESVVLAGGPSWR	peroxidase 37	2,1139	9,5435	4,5147	0,02437822	4,34E-06	0,00048373
Q96522	AT4G30170.1	1	STVNSFANSEGAFR	peroxidase 45	0,69944	1,1114	1,589	0,10834393	0,2742757	0,06355894
Q53YQ5	AT5G17820.1	7	GCDASLLIDSTNSEK,VGFYSQSCPQAETIVR,DSVALAGGPSYSIPTGR,NSATAALDQSSPLR,FGVTPTVTAAL	peroxidase 57	0,38298	0,78979	2,0622	0,00989442	0,13614272	0,02332326
Q96511	AT5G64100.1	4	SVVQSHVR,ISQASDVNLPGPSDSVAK,VVLQSDLVLWK,TLNTLDLVTLVGGHTIGTAGCGLVR,DAVVLTGG	peroxidase 69	0,26854	0,73974	2,8431	0,00119159	0,09601886	0,00380368
Q9FXT6	AT5G62810.1	1	AASAAASDVAR	peroxin 14	1,4265	1,9591	1,5477	0,11932159	0,01662694	0,10264234
Q9XEX2	AT1G65980.1	1	FALLLDDLK	peroxiredoxin-2B	1,2423	1,2548	1,0351	0,23142653	0,11094188	0,27726892
Q9LTV6	AT3G12800.1	1	QVLDDAVSALR	peroxisomal 2,4-dienoyl-CoA reductase	1,6205	3,1011	1,9137	0,06266339	0,00243954	0,03075562
O65202	AT4G16760.1	2	AEDWLNPDVVLEAFEAR	peroxisomal acyl-coenzyme A oxidase 1	2,3259	4,653	2,0005	0,01630164	0,00033184	0,02635736
Q9FZF1	AT1G47750.1	1	IILASSLIPETR	peroxisomal membrane protein 11A	9,9546	1,9273	0,19361	1,43E-05	0,01800598	0,00020764
Q9LQ73	AT1G01820.1	2	FLSDGQPGTAQNVDK,FVNDLHALISPVPK	peroxisomal membrane protein 11C	1,0791	1,2254	1,2106	0,19663579	0,11664798	0,35743897
O80845	AT2G45740.3	4	VTGAFGFITSIISCYQLLPTRPK,YQDEDYR,FVNDLHGLISPVPK,FLSGGQPGTAQNVDK	peroxisomal membrane protein 11D	0,94995	1,2422	1,2263	0,17906573	0,24306048	0,30883009
Q84JW1	AT3G61070.3	2	AELALIVLYLNK,FLSGGQPGTAQTVDK	peroxisomal membrane protein 11E	1,2626	1,9172	1,4958	0,21721359	0,03548118	0,11243933
Q8H112	AT4G22890.3	2	IDGSDIVSEGPR,CTNCGTAMVYDSGSR	PGR5-like protein 1A	1,1954	0,85629	0,81528	0,11932159	0,02907051	0,17153826
Q9STL8	AT3G48380.1	1	SLQTIISWFR	putative Ufm1-specific protease	1,9345	3,0496	1,5764	0,03424101	0,0024715	0,06567516
Q9FMU6	AT5G14040.1	17	IGMVGLFTR,GIEMYSPAFYAACTFGGILSCGLTHMTVTPLDLVK,GWVPTLLGYSAQGACK,TYSDLAGPEYT/	phosphate transporter 3	1,2612	1,6211	1,2712	0,19991482	0,14602234	0,44412411
Q9C5G5	AT3G51460.1	2	SIFGVVGMLK,LSILYEQIEGFLEK	phosphatidylinositol-4-phosphate phosphatase RHD4	1,0261	1,4152	1,3046	0,27571203	0,3425837	0,36785181
A4GNA8	AT4G25970.1	4	SVSADDFAGIALLTLIGAEMK,GLLGNDVQSDAFLDGSLVIFR,FVNVSGSLYTVNPIAVNSK	phosphatidylserine decarboxylase 3	1,1627	0,63821	0,54893	0,24242069	0,04737346	0,03588478
P29976	AT4G39980.1	1	AFDSILAEVR	phospho-2-dehydro-3-deoxyheptonate aldolase 1	1,1231	1,5781	1,4051	0,28995258	0,04558104	0,1055051
Q6ZY51	AT5G26570.1	1	LGSVGFFLER	phosphoglucan, water dikinase	0,86071	0,95616	1,1109	0,24294789	0,38178267	0,29405411
Q8H103	AT4G24620.1	1	DGVHNFFATFIEVLR	phosphoglucose isomerase 1	0,73329	0,99529	1,3573	0,12801913	0,48603608	0,12230716
Q8LGJ6		2	ALDAGIVAQAALDVFTK	Phosphoglycerate dehydrogenase-like protein	0,69577	1,1197	1,6093	0,1059528	0,26402898	0,05963097
Q9SAJ4	AT1G79550.2	2	YSLKPLVPR,LVAGLPEGGVLLLENVR,VDLNVPLDDNSNITDDTR	phosphoglycerate kinase	1,1426	0,9051	0,77508	0,36319769	0,13712719	0,12438478
Q9LD57	AT3G12780.1	3	IVPASGIEDGWMGLDIGPDSIK,SVGDLTSADLK,ADDCIGPEVESLVASLPEGGVLLLENVR,FAAGTEAIANK,I	phosphoglycerate kinase 1	0,8471	0,5607	0,67054	0,19507833	0,01878338	0,06904298
B9DFU6	AT3G08590.2	1	TILDAIEQVGGIYVVTADHGNAEDMVK	Phosphoglycerate mutase, 2,3-bisphosphoglycerate-independen	2,848	6,8565	2,4075	0,00649404	3,80E-05	0,01356674
Q7XZU1	AT5G20840.1	1	YGVPIIILNLIK	Phosphoinositide phosphatase family protein	0,58164	0,7497	1,2889	0,04832222	0,10717064	0,14695659
Q39033	AT3G08510.2	6	VAEMVTEIFGEILFTPPVGESLK,YTASEAPR,FTQHNLLR,KPDLLLK,DDFGGQTCLPVWELSEGIR,SGSDSDIF	phosphoinositide phospholipase C 2	1,1144	0,91763	0,78162	0,31077465	0,1307783	0,10264234
Q9FJB9	AT5G53650.1	1	ISTLESLR	uncharacterized protein	1,92	2,4855	1,2945	0,03514917	0,00590502	0,1451596
Q38882	AT3G15730.1	11	SIDGGAAAGFPESPEAAAEAGLVSGK,DVIQALR,DDNPIGATLIGR,VLLLVWDDR,AQGLEEDPR,YPIGVASE	phospholipase D alpha 1	0,77448	0,84033	1,0945	0,37451204	0,07754485	0,12815403
Q9SSQ9	AT1G52570.1	1	VILLVWDDR	phospholipase D alpha 2	0,78564	0,85975	1,1082	0,18321838	0,09573237	0,31972762
Q9T053	AT4G11850.1	2	TVIVDAEAAQNR,KPPQPNANANAAQVQALK	phospholipase D gamma 1	0,61411	1,818	2,9603	0,06099168	0,02319918	0,00556307
P52032	AT2G25080.1	1	SNAGGFLGGLIK	phospholipid hydroperoxide glutathione peroxidase 1	0,75534	0,8623	1,1416	0,14224503	0,21650168	0,25837114

Q9FNA9	AT5G13640.1	1	AIAPGFLDTDIFR	phospholipid:diacylglycerol acyltransferase 1	0,71966	1,0436	1,4501	0,11932159	0,38634643	0,09091872
Q8L9T0		1	LSFDVWDVK	unknown	0,52193	0,31669	0,60677	0,03514917	0,00229085	0,05476911
P25697	AT1G32060.1	12	GGNPDSNTLISDTTTVICLDDYHSLDR,LTSVFGGAAKPPK,QYADAVIEVLPTTLIPDDNEGK,LDELIYVESHLS	phosphoribulokinase	0,59136	0,67578	1,1064	0,23630779	0,4818143	0,23682267
Q547D7		1	SFNQLIETLIDR	phosphorybosyl anthranilate transferase 1	1,3177	1,3014	0,98757	0,14408762	0,12133318	0,46793219
Q94K59	AT4G24350.1	3	QITNAGLWDWLNPDK,GGPYIGLVTVIATEENAFLR	phosphorylase family protein	0,71509	0,89064	1,2455	0,11723348	0,26070743	0,17026467
Q9ZV56	AT2G32260.1	1	MGTAIVDSIQER	phosphorylcholine cytidylyltransferase	1,1492	1,3781	1,1992	0,25842729	0,09060668	0,20537949
P56788	ATCG00520.1	1	AAELAYFLR	photosystem I assembly protein Ycf4	1,0901	1,7679	1,6217	0,33033186	0,02593261	0,05824542
P56766	ATCG00350.1	9	YNDLLDR,ALSIIQGR,AVGVTHYLLGGIATTWAFFLAR,ANLGFR,GVLFAR,DLLAQLYPSFAEGATPFFTLNW	photosystem I P700 chlorophyll a apoprotein A1	1,1822	0,92591	0,86289	0,30314377	0,23940646	0,1573503
P56767	ATCG00340.1	15	TNFGIGHSIK,IWFGIATAHDFESHDDITEER,GYWQELIETLAWAHER,TPLANLIR,GGALGPVNIAYSGVYQW	photosystem I P700 chlorophyll a apoprotein A2	1,2389	0,87257	0,80594	0,22642083	0,1854632	0,10388456
Q9S7H1	AT4G02770.1	8	TDSSAAAAAAPATK,EGVGLNMR,AQVEEFYVITWNSPK,VFPNGEVQYLHPK,EQIFEMPTGGAAIMR,ITYQ	photosystem I reaction center subunit II-1	0,84762	0,78769	1,0161	0,42619086	0,12309784	0,12852101
Q9SHE8	AT1G31330.1	7	GFIWPVAAYR,YGLLCGSDGLPHLIVNGDQR,EIIIDVPLASR,SYLIAISGEK,EFLNGDLIAK,LYAPESAPALALN	photosystem I reaction center subunit III	1,5266	0,91415	0,56751	0,04040215	0,46095579	0,03345105
Q9S831	AT4G28750.1	2	VNYANISTNNYALDEVEEVAA,DSPAAAAAPDGATATKPKPPPIGPK	photosystem I reaction center subunit IV A	3,4944	1,6199	0,46358	0,00296118	0,04031805	0,01994209
Q9S714	AT2G20260.1	5	VNYANISTNNYALDEVEEVK,AKPPPIGPK,AAEDTPPATASSDSSSTTAAAAPAK	photosystem I reaction center subunit IV B	1,8264	1,4596	1,107	0,36417609	0,16863695	0,14369822
P49107	AT5G64040.1	2	FPENFTGCQDLAK,VPFISEDIALECEGK	photosystem I reaction center subunit N	1,7317	0,81441	0,46671	0,04661118	0,24306048	0,02638708
Q9S7N7	AT1G55670.1	3	FVFFNFQR,THFEAGDDR	photosystem I reaction center subunit V	1,4848	1,814	1,1	0,13960785	0,32795148	0,17419534
Q0WWU6	AT3G16140.1	2	FFETFAAPFTK,FLILGGGSLLTYVSATSTGEVLPIK	photosystem I reaction center subunit VI-1	1,4493	0,96125	0,70488	0,14158087	0,34770623	0,10404583
Q9SUI6	AT1G52230.1	1	FLILGGGSLLTYVSANSTGDVLPIK	photosystem I reaction center subunit VI-2	0,86006	0,79581	0,92529	0,24242069	0,14149413	0,33964666
Q9SUI4	AT4G12800.1	5	GVEVGLAHGFFLVGPFVK,QPDQLQTADGWAK,EGEPSIAPSLTLTGR,TAVNPLLR	photosystem I reaction center subunit XI	1,2874	1,0677	0,92772	0,24318492	0,18433221	0,11195408
P62090	ATCG01060.1	4	ACPTDVLEMIPWDGCK,IYDTCIGCTQCVR,CESACPTDFLSVR,VYLWHETTR	photosystem I subunit VII	1,7562	2,1518	1,2987	0,28379817	0,38748556	0,22571128
Q9XF91	AT1G44575.1	8	AVIPPGK,ANELFVGR,VEDGIFGTSGGIGFTK,FITDDGEES,LAQLGIAFSLIGEIITGK,VAMIGFAASLLGEALT(	photosystem II 22 kDa protein	2,1022	1,9335	1,0768	0,42002701	0,0693958	0,07814145
P56778	ATCG00280.1	3	APWLEPLR,ITNLTLSPSVIFGYLLK,SAEYMTHAPLGSLNSVGGVATEINAVNYVSPR	photosystem II 44 kDa protein	1,1075	0,61379	0,51204	0,35149027	0,00342716	0,01057435
P56777	ATCG00680.1	15	VHTVVLNDPGR,VSAGLAENQSLSEAWAK,YQWDQGYFQQEIYR,MPTFFETFPVVLVDGDGIVR,DVFAGID	photosystem II 47 kDa protein	1,1906	1,049	0,86473	0,3876651	0,33799264	0,27772461
Q9S7J7	AT2G05070.1	2	WAMLGALGCTFPEILSK,IGGGPLGEGLDPLYPGGAFDPLNLAEDPEAFSELK,GPIENLFDHLADPVANNAW	photosystem II light harvesting complex protein 2.2	2,1824	1,9734	1,0577	0,11568153	0,047496	0,28894266
P83755	ATCG00020.1	10	ETTENESANEGYR,FGQEEETYNIVAAHGYFGR,ESESLWGR,VINTWADIINR,EWELSFR,ANLGMEVMHER	photosystem II protein D1	1,2405	1,1378	0,90372	0,29965193	0,38317006	0,36371306
P56761	ATCG00270.1	10	DLFDIMDDWLR,WCQLGGLWAFVALHGAFALIGFMLR,NILLNEGIR,FWSQIFGVAFSNK,AYDFVSQEIR,Q	photosystem II protein D2	1,1643	1,0311	0,87875	0,40683029	0,31995215	0,27742113
P60129	ATCG00560.1	1	TQSNPNEQSVELNR	photosystem II protein L	1,3004	1,0543	0,81074	0,15102512	0,36465123	0,17723399
Q9LR64	AT1G03600.1	2	DIYSALNAVSGHYVSFGPTAPIPAK,ILEEMETAEK	photosystem II Psb27 protein	1,6168	1,4312	0,95083	0,04823352	0,32702643	0,06053473
O82660	AT5G23120.1	13	SAEMVTDEGAIYVTSNR,AAIQETVSATLNR,SIPSAEEEDFNYR,GTGITEEFEEVPVQSR,QTLLETK,IPLSSQLI	photosystem II stability/assembly factor HCF136	1,1484	1,1165	0,99112	0,22069077	0,16604258	0,44368492
P27202	AT1G79040.1	1	YGANVDGYSPIYNENEWSASGDVYK	photosystem II subunit R	0,73622	1,006	1,3664	0,12948732	0,4818143	0,11842417
A8MS49	AT5G58140.3	2	ATATNVDEAVR,GINWPLIR,QLINTLLNR	phototropin 2	0,74253	0,89115	1,1956	0,15102512	0,31930478	0,2004875
Q8H0W8		1	SAPILQLIR	Unknown protein	1,9158	2,1272	1,1104	0,03514917	0,01183842	0,2940748
Q94EI1		7	VEDGIFGTSGGIGFTK,FITDDGEES,LAQLGIAFSLIGEIITGK,VAMIGFAASLLGEALTGK,FVDDPPTGLEK	At1g44575/T18F15	0,52207	0,55254	1,0584	0,03514917	0,02357655	0,37531276
Q07356	AT4G14210.1	2	VVIAGAGLAGLSTAK,SLGGEVQLNSR	phytoene dehydrogenase	1,3511	1,8628	1,3787	0,13242562	0,02067516	0,11454667
Q2V3F7	AT4G23470.3	2	CPQLCLATEVFCCFANSVASTR,FLLQDEFQIQTTK	PLAC8 family protein	1,1527	1,5522	1,3466	0,25416001	0,04947189	0,12567625
Q9FVQ1	AT1G48920.1	4	GERPAFTPQSGNFR,GFGHVEFASSEEAQK,SSDVEMVDAEK,GFDASLSEDDIK	nucleolin	1,781	1,786	0,98905	0,03655796	0,01434903	0,35685866
O80796	AT1G65260.1	2	GVVDNLVSNTR,SFADNATALK	plastid transcriptionally active 4	0,95979	0,83778	0,87485	0,33667925	0,34558619	0,24617869
Q56ZZ7	AT5G16150.3	2	DLGIAENTVLQGWIVSSLLAGATVGSFTGGALADK,SLEEIELALTSGA	Plastidic glucose transporter 4	0,82066	0,6285	0,75127	0,2069276	0,00787023	0,03075562
P42699	AT1G20340.1	2	NNAGYPHNVVFDEDEIPSGVDVAK	Plastocyanin major isoform	1,6432	1,8776	1,1426	0,05996128	0,02029567	0,25750992
Q9FJC9	AT5G53540.1	1	LQPAIIFIDEVDSFLGQR	P-loop containing nucleoside triphosphate hydrolase-like proteir	1,2118	1,4859	1,2261	0,20653755	0,06193731	0,18310043
Q9FXA2	AT1G49760.2	2	GYGFVQYDTDEAAQGAIDK,NLSESLSDEELNK	poly(A) binding protein 8	0,69037	1,1367	1,6466	0,10215749	0,24306048	0,05485971
O80915	AT2G38360.1	1	NFINQITETVK	PRA1 family protein B4	0,99763	1,7779	1,7822	0,49496207	0,02547932	0,03854945
P57681	AT5G63910.1	3	MSSFIENTVDNFLK,LSEAQLSPLLVNELVTVITR	prenylcysteine oxidase	1,0563	1,8755	1,7756	0,38180254	0,02040688	0,03932243
Q9SRM4	AT3G11200.1	1	LFSLINDLPTLFDVVTGR	alfin-like 2 protein	1,8897	3,0985	1,6397	0,03706326	0,00243954	0,0555293
Q38885	AT2G18710.1	5	LLGFLALSR,LGNGTSLLIFTSIISYLPASFGR,EAFVGNLDQNSILSTLDTFSGGGIGR,GVAAAIEDSSIDFGDFFK,	Preprotein translocase subunit secY	1,3532	1,919	1,4639	0,09779852	0,06153477	0,46388171
C0LGG8		1	GQSTFSSVSER	Probable LRR receptor-like serine/threonine-protein kinase At1g	1,3734	1,2737	0,98533	0,16002849	0,13401989	0,47047242
Q9ZNT7	AT1G03860.3	3	VYLSSNDLLLNLQEMNLEPK,LPQIYR,VPNIPGAPALSALLK,EIAQTIAQSANK	prohibitin 2	1,3625	2,3235	1,6453	0,11445402	0,00799285	0,05550763
O04331	AT5G40770.1	4	VLSRPEVSR,AAFGLGTAATVLNTSLFTVDGGER,SPNVAYLPGGQSMLFALNR	prohibitin 3	1,3167	2,5105	1,7584	0,10965279	0,00583395	0,04132934
Q9LK25	AT3G27280.2	1	AVVANFNADQLLTERPQVSALVR	prohibitin 4	1,8369	6,69	3,5219	0,04386127	7,19E-05	0,00264551
Q9SIL6	AT2G20530.2	3	SAQLIGQAIANNQAFLTLR,AKPYLVESTSGSR	prohibitin 6	1,4166	5,0062	3,1314	0,1143373	0,00322512	0,02638708
Q8LA39		3	AANFNVALDDVSITNLTFGK,ARPYLVESTSGSR,EIAQTIANSANK	prohibitin-like protein	1,6836	5,1604	2,8862	0,05725506	0,00036465	0,00775371
Q39221	AT2G01470.1	4	VDLNTNSLSEQPLGR,EDNQAGESEEVIK,GGSIITCDTK,LLAIGTLEGDVLILESTR	prolactin regulatory element-binding protein	1,0519	1,2965	1,0999	0,31014605	0,32795148	0,43108996
Q9FL44	AT5G07020.1	1	AVDYSGPSLSYYINK	proline-rich family protein	1,3075	0,66727	0,51033	0,14833887	0,05915108	0,0277367
Q9M1R2	AT3G62120.2	5	LILAPWCDEEEVER,DLENDQVR,IYEEYLAVPVVK,AEADEEVLQILELYR,NCYFPLFVSPGVLEK	prolyl-tRNA synthetase	1,1707	1,0905	1,1026	0,48810215	0,29413749	0,32296136
Q42044	AT2G45180.1	1	TPCCTLLQGLANLEAAVCLCTALK	protease inhibitor/seed storage/lipid transfer protein (LTP) famil	1,115	0,62415	0,5684	0,47794873	0,0194921	0,03225315
Q8W4A0	AT3G02200.2	1	ELFLAIANVLK	Proteasome component (PCI) domain protein	0,72407	1,4385	1,9867	0,1203441	0,07333292	0,0266429
O23712	AT1G47250.1	1	SSLCTVSVLGVDEPFHFLDQESIQK	proteasome subunit alpha type-1-B	0,89616	0,22988	0,25652	0,29763493	0,00044965	0,00111953
Q8L4A7	AT1G79210.3	2	LVQIEHALTAVGSGQTSLGIK	proteasome subunit alpha type-2-B	1,001	1,4272	1,4258	0,49784913	0,07596297	0,09913303
O23715	AT2G27020.1	2	AVDNSGTVVGIK,VPDDLLEEAK	proteasome subunit alpha type-3	0,95457	1,4616	1,5311	0,39554166	0,06806654	0,07417003
O81148	AT3G22110.1	5	AAAVGANNQAAQSILK,IDDHVACAVAGIMSDANILINTAR,HGVTQPAAETS,LELAEVYLTPSK,LYQVEYAM	proteasome subunit alpha type-4	1,0556	1,3438	1,3436	0,36319769	0,04134429	0,04504245
O81149	AT1G53850.2	2	DLSLQEAETIAVSILK	proteasome subunit alpha type-5-A	0,98335	2,0401	2,0746	0,45803923	0,01401529	0,02245587
Q42134	AT3G14290.1	2	DITLQEAETIAVSILK	proteasome subunit alpha type-5-B	0,96939	1,86	1,9187	0,4256023	0,02080735	0,03067041
P30186	AT3G51260.1	2	IPALYQTDPSGTFSAWK,LTLEDPVTVEYITR	proteasome subunit alpha type-7-A	0,80405	1,2031	1,4963	0,18463535	0,17718645	0,07939051
P42742	AT3G60820.3	4	HLIYQHQHNK,GCVFTYDAVGSYER,QDSNTPLSEAEAVDLVK,AVLSSSGFQADVK	proteasome subunit beta type-1	0,87389	1,6521	1,8905	0,26346102	0,03625266	0,03184562
Q9XI05	AT1G21720.1	1	VFIGLSGLATDVQTLYQR	proteasome subunit beta type-3-A	0,96396	2,0878	2,1659	0,41535462	0,01255961	0,01957823
Q7DLR9	AT1G56450.1	1	FNPLWNTLVLGGVK	proteasome subunit beta type-4	1,2064	1,4233	1,2583	0,35640931	0,03565511	0,07150049
Q2V3D4	AT4G31300.2	2	SGSAADSQVVSDYVR,ITQLTDNVYVCR	proteasome subunit beta type-6	0,89682	1,6903	1,8848	0,29871937	0,03209287	0,03184562
O04379	AT1G48410.1	5	QSDAPQEALQVLDIVLR,SGNILPGTVVDSK,ELLIAFR,LFAQNHNDR	protein argonaute	0,62404	1,4771	2,367	0,06454334	0,06380264	0,01398742
Q9FZ33	AT1G54990.1	1	SGELADYISEFVSLLPK	protein auxin RESPONSE 4	0,64132	1,8659	2,9095	0,07413148	0,02063113	0,00617974
Q8LDS7	AT1G48440.1	1	LSCSSEVCTATER	protein B-cell receptor-associated 31-like protein	0,96635	1,3503	1,4425	0,44298898	0,09935711	0,13380921
O65605	AT4G25570.1	1	GGLAWEATNK	cytochrome b561/ferric reductase transmembrane family prote	0,53011	0,46257	0,8726	0,03733465	0,01107518	0,25387857
Q9SRG3	AT1G77510.1	2	LFKPFDELFVDSK,LDATANDIPSDTFDVK	protein disulfide-isomerase 2	1,4718	1,7668	1,2004	0,09614373	0,02599306	0,20402169
Q9XI01	AT1G21750.1	5	VVVSDSLDDIVLNSGK,DFDGEALEK,IDASEETNR,VVVVGIFPK,AASALSSNVPPVVLAK	protein disulfide-isomerase A1	1,4007	1,7938	1,3282	0,172787	0,0325878	0,12929979
O48773	AT2G32920.1	2	VQGFPTILVFGPDK,GVATVAAIDADAHQSAAQDYGIK,SAFELQHLLEFVK	protein disulfide-isomerase A6	1,4667	1,7661	1,254	0,16916149	0,03089351	0,12730146
O22263	AT2G47470.1	2	DLDDFVSFINEK,AGIVESLDALVK	protein disulfide-isomerase A6	1,2272	1,3086	1,2173	0,35896271	0,13675276	0,12320195
A8MSC5	AT2G47470.4	1	AGIVESLDALVK	protein disulfide-isomerase A6	1,0672	1,4251	1,3353	0,36464091	0,07648908	0,12852101
Q9FE06	AT5G64260.1	2	GGSSTLVVGK,DVASSAAVPSVASWWK	protein EXORDIUM like 2	0,75373	1,3648	1,8108	0,14101618	0,09496392	0,03633011
Q0WUY1	AT1G04280.1	1	VSEEGTITEK	protein H1flk	1,4764	2,1034	1,4247	0,09523046	0,012231	0,09913303
O65719	AT3G09440.2	2	FTDSSVQSDIK,VQQLLVDFFNGK,EIAEAYLGTTIK	protein heat shock protein 70-3	1,0659	1,3892	1,3128	0,35603209	0,09571385	0,15691705
Q9LZV4	AT5G01920.1	1	LGEGSFGVVYAGVLLPK	protein kinase family protein	1,5525	1,1872	0,7647	0,0753558	0,19026959	0,13880363
O22100	AT3G63260.1	3	VLDWGEDGYATPAETTALR,DVIQLALDLAR,LLEAIDTSK,IADFGVAR	protein kinase family protein	1,2915	1,1416	0,9279	0,12549222	0,16863695	0,27410059
Q9M1Q2	AT3G62220.1	1	ANPHVIHR	protein kinase-domain containing protein	1,7029	2,2239	1,306	0,05024148	0,00976953	0,13987712
Q9LQG5	AT1G35620.1	3	FVAPDVAVLESDSTVK,SLGQVEEPTGVR,LNPELDAAAPILAK	protein PDI-like 5-2	0,8213	0,89552	1,0904	0,20219543	0,26763162	0,32439444
Q9XGR4	AT5G44090.1	1	LPSFLSSVLFR	protein phosphatase 2 (formerly 2A), regulatory subunit B''	0,61191	0,65266	1,0666	0,06099168	0,05301425	0,36358313
Q0WW19	AT1G17720.1	2	SFFTEIIASISDIK	Protein phosphatase 2A, regulatory subunit PR55	0,87873	1,1333	1,2722	0,40157837	0,11327618	0,11537043

Q9STF2	AT3G46780.1	8	IASLVADIFANTAVAENK,VYADAIAR,EAEAASLAEDAQQK,VAQTDVAYTLIK,AGVPDLGAAQDLAR,ADAV	protein plastid transcriptionally active 16	1,5029	1,0442	0,68641	0,09614373	0,44412679	0,07873399
Q9SL05	AT2G05620.1	1	GLFAPLVVVTR	protein PROTON GRADIENT REGULATION 5	1,0812	1,9951	1,8245	0,33689661	0,01758657	0,03801615
O65449	AT4G22010.1	7	IPVPFSPPDGDFWLLAGDWYK,NSWQDGVVGTTCPIPPGK,ITYGDIYPLGVK,GWGGNTFTVQPGK,STVQ\	protein SKU5 similar 4	0,93096	0,97611	1,0485	0,35559811	0,4310875	0,39383438
Q9SKT0	AT2G20890.1	5	RPIPSIYNTVLQELIVQQHLMR,LLELASATDPTVLDK,AYIEALNEDPK,LCASLNINK,SQTSASLVDFSSK,FFA\	protein THYLAKOID FORMATION 1	1,1116	1,1466	1,0484	0,33467429	0,20427284	0,34337213
Q9STE8	AT3G46740.1	4	LGNVVEGNTQVPVVR,TGQGSSYGAGVK,TVFQVDQGLGIGSK,EVVCEVVEGDITQLVIQFQDK,LLPSGGIS	protein TOC75-3	1,272	2,0166	1,3312	0,10113346	0,02204311	0,15800496
Q9LIC2	AT3G13772.1	1	NLIQGNTVPQEVEQGK	protein transmembrane nine 7	0,66069	0,7555	1,1435	0,08384724	0,11094188	0,25679238
Q7X659	AT2G17790.1	4	VEALFELIK,GVPQITVESVESLIK,AQVTALQLIIGTLQR	protein VPS35A	0,9276	1,6821	1,8134	0,34845914	0,0325878	0,03633011
B9DG36	AT4G27440.2	2	TETPLDVLVCNAAVYFPTAK,QFVDNFR	protochlorophyllide reductase B	1,2128	0,88152	0,76615	0,21721359	0,12693655	0,07196314
Q2V4R1	AT1G03630.2	3	LAQVVSDPSLGK,YHEETGVTFASLYPGCIATTGLFR,QFVENFR	protochlorophyllide reductase C	1,0467	0,81546	0,78201	0,47275753	0,1290002	0,15691705
O23144	AT4G27500.1	4	EEPVSAPKPDATVAQNTEK,SELFDLLDPLK,FISLWCSK,EEKPVDAATAK	proton pump interactor 1	0,87478	1,5529	1,66	0,34264664	0,05546255	0,0555293
Q5M730	AT4G01690.1	9	TECLIDGELK,SSLTSSGYEGLFLGGNYVAGVALGR,LTDLPFFDLMSIGGK,SEGELVEAVDR,HPDAAPNLIVTE	protoporphyrinogen oxidase	1,6785	1,1753	0,71601	0,03241734	0,14624026	0,06483965
Q8S9J1	AT5G14220.1	5	FQILLEPFLWK,GLNVTVFEADGR,VSDASAEESVSEFFQR,RPLEGFGVLIPSK,VAVVGAGVSGLAAAYK	protoporphyrinogen oxidase	1,2396	1,6328	1,3171	0,18621086	0,03847704	0,13576487
O22826	AT2G43770.1	1	IFTGGVDNDVK	Prp8 binding protein	0,82938	0,94282	1,1368	0,21184587	0,35149375	0,26287732
P82538	AT3G55330.1	1	TTLIDASEHDVDGK	PsbP-like protein 1	1,3038	1,7627	1,3519	0,1497995	0,02619155	0,12396969
Q8LCR7		6	WLAYGEIINGR,FAMLGAAGAIAPEILGK,GLAGSGNPAYPGGPFFNPLGFGK,LQDWYNPGSMGK	PSI type III chlorophyll a/b-binding protein, putative	1,2725	0,86005	0,68607	0,19991482	0,27006484	0,12117762
Q9C508	AT1G57990.1	2	EDSDQDLQYR,IGTLIATVVALGSYFYTLHK	purine permease 18	0,62772	1,0844	1,6182	0,0390965	0,16249493	0,07791112
O04309	AT3G16470.2	20	IVGFYGQAGEYLYK,VYVGQGDSGVVYVK,QTLLGTEEFVVDPEDYITSVK,LFGSPIEIVTALIFK,TSIPYGLEGG	myrosinase-binding-like protein	0,62001	0,34403	0,56779	0,0374089	0,00536658	0,12730146
Q9LMI0	AT1G06410.1	1	AVLVQIVNPAR	putative alpha,alpha-trehalose-phosphate synthase [UDP-formir	0,77639	0,7474	0,96265	0,15784004	0,10533992	0,41363758
Q9CAE1		3	EPTFIPVVVGLLDSSGK,SSSLAR	putative aminopeptidase	1,1947	1,4757	1,2352	0,21841012	0,06404894	0,17651228
Q940V0	AT2G04350,2	2	HSEFAELCEK,MLWDALVFK,LLSEPWTPESGLVTAALK	putative AMP-binding protein	1,7513	2,7196	1,5529	0,04581505	0	0
Q8GX78	AT4G00370.1	1	LGLPFLLVVR	putative anion transporter 2	1,0768	2,4774	2,1702	0,40270994	0,01057178	0,02145069
Q39196	AT4G00430.1	1	EPPPAPLFEPGELSSWSFYR	putative aquaporin PIP1-4	0,71257	0,36079	0,50633	0,11568153	0,00335257	0,02747498
Q8LAA6	AT4G23400.1	1	QPIGTAAQTESK	putative aquaporin PIP1-5	1,4983	0,91448	0,6729	0,10293836	0,11488251	0,03146056
Q9FF53	AT5G60660.1	1	DLDVNESGPPAAR	putative aquaporin PIP2-4	3,8444	0,47942	0,12471	0,00186276	0,01259507	1,58E-05
Q9ZV07	AT2G39010.1	2	VFQSTYYNR,SFGAAVIYNNQK	putative aquaporin PIP2-6	0,84169	0,76166	0,90374	0,22007147	0,10904609	0,29002757
Q41975	AT4G17340.1	1	IEIGSVGDSFSVASLK	putative aquaporin TIP2-2	1,037	0,21366	0,19154	0,21696531	0,00085606	0,00046994
Q3EBW9	AT2G21870.2	4	FQAAVDILR,EAARPTFK,ITLDPEDPAAVK,AYLLK	putative ATP synthase subunit	1,1798	1,6474	1,3963	0,22812793	0,03674982	0,10811884
Q9SJ12	AT2G21870.1	8	FQAAVDILR,EAARPTFK,YEENLELSMAK,AYLLK,GLTDELGAEAMMFEALEK,AQLDELK,GVFFDIK,ADMF	putative ATP synthase subunit	1,0309	1,2885	1,2499	0,42619086	0,12620998	0,1671942
Q9LUJ7	AT3G22640.1	1	EVLSTSFNVPEELLGR	cupin family protein	0,53207	0,53538	1,0062	0,0374089	0,02063113	0,48434743
Q9SGZ5	AT1G78060.1	8	NAVYHQGCDSVACSNAAIDQAVAIAK,AATSFPQVILTAASFDSYEWFR,IGSIIWAGYPGEAGGIAISEIIFGDH	putative beta-D-xylosidase 7	1,2227	1,2397	1,1217	0,11867247	0,32702643	0,14425372
O49499	AT4G34050.1	1	EGPALPVLDEIVADEK	putative caffeoyl-CoA O-methyltransferase	1,1474	1,5789	1,3761	0,26015474	0,04557805	0,11537043
Q9LE22	AT1G18210.2	1	ISVLELGGVFK	putative calcium-binding protein CML27	1,3398	1,9306	1,3466	0,22851155	0,11310742	0,28589765
Q9FYE4	AT5G04170.1	1	EFTALFYSLQNWR	putative calcium-binding protein CML50	1,1293	1,0704	0,96962	0,42808857	0,3056281	0,37739022
Q8LC80		2	SGSLFDNVLICDDPDYAK,YVGLELWQVK	putative calcium-binding protein, calreticulin	0,85289	1,1414	1,3383	0,23526536	0,23873136	0,12852101
Q9CAY2	AT3G11450.1	1	FYNFWYAFK	putative cell division related protein	0,93921	1,4116	1,503	0,36922949	0,0801249	0,07866686
O22842	AT2G43610.1	1	YCSPSTTYPCQPGK	putative chitinase	0,3479	1,5321	4,4039	0,00649404	0,05301425	0,00054729
P42734	AT4G39330.1	2	LIALGLPEKPLELPMFPLVLGR,VTVISSSSTK	putative cinnamyl alcohol dehydrogenase 9	0,91032	0,7759	0,85233	0,31825971	0,12620998	0,22896149
Q8LB43		2	EIITGNEVALDLVQVFLH	putative cytochrome P450 monooxygenase	0,94052	1,2111	1,2877	0,37103852	0,17174434	0,14773663
Q93ZX2		1	EGSPVNLGEQIFLSIFNVVTR	putative cytochrome P450 protein	0,96806	1,9486	2,0129	0,42303608	0,01738877	0,02532622
Q8LF35		1	SVFGGQLVLR	putative ER lumen protein retaining receptor	0,79108	0,90842	1,1483	0,17206879	0,2878086	0,25169131
Q9SCY3	AT4G39710.1	1	FADGTLFDSSYK	putative FKBP-type peptidyl-prolyl cis-trans isomerase 4	1,347	1,345	0,99854	0,13337707	0,10190205	0,49515082
Q9LDY5	AT1G18170.1	2	GQVQGTGQVFVDTFGTK,IEVSQEVANTR	putative FKBP-type peptidyl-prolyl cis-trans isomerase 5	0,68914	0,80887	1,1737	0,10169012	0,15438148	0,22896149
Q9FJ62	AT5G55480.1	3	SAAFDGNSYGISTVK,SYIWPIESQYLLPR,FVSLSDFLNLAK	putative glycerophosphoryl diester phosphodiesterase 1	0,43781	1,1378	2,5947	0,01692281	0,48801611	0,01568383
Q9SZ11	AT4G26690.1	6	TVLIDFISSPEVNFFK,LSVFPVAQSFITTQTNVVEK,DGASGDYPGCTDLAYK,SPWPTLTGDPPLVIAR,LFSLSD	putative glycerophosphoryl diester phosphodiesterase 2	0,62028	1,0897	1,8187	0,06587546	0,2972604	0,08876987
Q7Y208	AT1G66970.1	1	LISLSQFLDLAK	putative glycerophosphoryl diester phosphodiesterase 3	0,57097	1,0341	1,811	0,04661118	0,40771676	0,03633011
Q8H153		15	TPKPIAYFEYEFFTK,VWSYSTLATSTAWDVTYTAFTNADTDGNDECR,CLGFFYNR,GLLGWDETCK,VVNEG	putative glycoprotein (EP1)	1,4235	1,1683	0,83255	0,05198642	0,0471138	0,34669332
Q8H158		4	GIEVLYMVDAIDEYAIGQLK,APFDLFDTK,EGQNDIFYITGESK,EDQLEYLEER,SGDELTSLK,GIVDSEDLPLNI	putative heat shock protein 81-2 (HSP81-2)	0,85497	1,435	1,6487	0,15554632	0,29797719	0,10264234
Q9M8R8	AT3G02760.1	1	AFSIIQNVFK	putative histidyl tRNA synthetase	1,2879	1,7196	1,3352	0,15784004	0,02947581	0,12852101
Q7FLV1		1	GLPLGSGLGSSAASAAAAAVAVNEIFGR	putative homoserine kinase HSK	0,85262	0,92964	1,0903	0,23526536	0,32616632	0,32439444
Q8GZ46		3	STASQVIAK,VVCEATLSPEVK,QALPGLVPLLLETLLK	putative importin beta	1,7266	1,367	0,85863	0,20358135	0,28416113	0,30818781
Q9M8T0	AT3G02880.1	9	SSNILLSDSYEAK,VSDYGLAPIISSTSAPNR,SPTHQQLNEEGVDLPR,ALLAVR,TPLNWETR,AGIALGAAR,LL\	putative inactive receptor kinase	1,2531	1,3645	1,0678	0,39893743	0,29170312	0,25156677
C0LGT4	AT5G16590.1	1	SIEAAPVPTSSAAVAK	putative inactive receptor kinase	0,56514	0,85822	1,5186	0,04568662	0,21011354	0,07594912
Q8W593	AT1G67280.1	1	SVFVDNIDFLK	putative lactoylglutathione lyase, chloroplast	1,3629	1,1219	0,82318	0,12801913	0,26200924	0,191671
Q84WT1		2	LGFIENFSWYDAGSR,WADLIKPGSVDIEPK	putative light-harvesting chlorophyll a/b binding protein	1,2543	1,2029	1,031	0,23630779	0,17644154	0,43346509
Q94BN4		4	NIWANDLAASLITLQSLEGVVGK,SFDLLSLLPK,ALGVETVPVLVGPVSYLLLSK	putative methionine synthase	0,91106	1,1425	1,2541	0,31991806	0,23746354	0,16437754
B9DFI7	AT1G26850.2	4	LVDHEDGPLVPEK,AIQNWIQYEGDVFR,ISSGSISGVTVDAYEDDNR,GVPAVIGVLGTIK,GYVTPFSWPK	putative methyltransferase PMT2	0,78137	1,1046	1,2662	0,311508	0,14609819	0,11447098
Q6NPR7	AT1G29470.2	2	VILDVGCGVASFGGYLFDR,TYDLLHADHLFSSLK	putative methyltransferase PMT24	1,0502	1,3554	1,2907	0,39151124	0,09819711	0,14587407
Q940J9	AT1G04430.2	3	TYDLLHAWSIFSDIK,DGLLLLELDR,ILRPTGFVIIR	putative methyltransferase PMT8	0,94878	0,81792	0,98983	0,43748202	0,3731268	0,4440009
Q0WWT6		22	IPTAELFAR,DIAISAIGPIQDLPDYNK,IDAVDASTVK,VLDSNVNQALDVLADILQNSK,TILGPAQNVK,VAINE	putative mitochondrial processing peptidase	1,5346	1,5706	1,0775	0,48810215	0,12622377	0,1451596
Q9ZU25	AT1G51980.1	12	AIELAAK,EQMSYTIDALK,VAEPLTSDLPNVPPQLAPK,NPMGFLLEAIHSAGYSGPLASPLYAPESALDR,LNG	putative mitochondrial-processing peptidase subunit alpha-1	0,84438	1,0418	1,2085	0,43640902	0,12251207	0,12974288
Q84WD8		1	FSFGESTSR	putative Na+ dependent ileal bile acid transporter	1,3065	2,8675	2,1949	0,14877313	0,00296739	0,01900348
Q9SKC9	AT2G02050.1	1	QAEFYLPWK	putative NADH-ubiquinone oxidoreductase B18 subunit	0,78224	0,68452	0,87507	0,16337716	0,06809682	0,25750992
Q8VXZ3		1	LASGEVFGPDQPIALK	putative NADP-dependent malate dehydrogenase	1,0348	1,1518	1,1131	0,42004279	0,22601448	0,29184272
Q8LCL4		1	GPTLEDGGEDESSDGFVR	putative non-green plastid inner envelope membrane protein	0,79691	1,2607	1,582	0,17725625	0,13893232	0,06464314
Q9MAB3	AT3G05060.1	1	LDFSEILADEIEADLK	putative nucleolar protein 5-2	1,6102	1,8211	1,131	0,06347689	0,0230881	0,27044118
O65782	AT4G31500.1	5	ELGFGQYTAYYR,AADQSGTVDLSELLLSFTNCVVCR,DTAAWGDNPNEFIPER,FDWSLPK,LAVISSAELAK	cytochrome P450 83B1	1,879	2,4696	1,3144	0,0374089	0,00603337	0,13614308
B9DHI6	AT3G14420.2	2	DVQWLQTITK,NVVEQLVR	putative peroxisomal (S)-2-hydroxy-acid oxidase 2	0,85145	0,71007	0,83878	0,17452586	0,08050429	0,28320703
Q8L9P5		1	GSNNVTALETFLLGAVAK	putative peroxisomal membrane carrier protein	1,6519	3,5388	2,1423	0,05861778	0,0012406	0,02006469
Q9ZVA2	AT1G78830.1	7	GMCNACPTPK,FVWQSFDHPTDTLLTGQSLK,GVTGFQILPNGNIVLHDK,CLLAPLLGTLIK,VVNEGEFGEYI	curculin-like (mannose-binding) lectin-like protein	1,9959	1,5256	0,77395	0,0377867	0,0307626	0,31160325
Q9FDY5		2	VNYANISTNNYALDEVEEVAA,DSPAAAAAPDGATATKPKPPPIGPK	putative photosystem I subunit PSI-E	0,89089	0,87828	1,0761	0,09943575	0,11664798	0,2908149
O81439	AT4G04020.1	3	AIESVEETER,AEISELITQLESK,VFASSSTVSVADK,SLADSLYGTDR	putative plastid-lipid-associated protein 1	1,3289	1,1587	0,88793	0,22666054	0,23873136	0,42006128
Q8LAP6	AT1G51110.1	7	VLEGLEGIQNPTDSDLIEGR,GFDNIIFVHIR,FSDFIGELK,LIDALIGIQGR,GWLDTTYLSPSGNLR,TFTGVDVF	putative plastid-lipid-associated protein 12	1,1214	1,2735	1,1542	0,16588096	0,08496008	0,31633523
O49629	AT4G22240.1	3	GLSASSETR,FAGPLGTNSISTNAK,IDLNPIR,AEIGDLITQLESK	putative plastid-lipid-associated protein 2	1,2671	2,0444	1,5418	0,36165493	0,1854632	0,15355836
O82291	AT2G35490.1	4	GDGGLFVLAR,QSLNPLQDVAANISR,CLADSVYGTELGFK,GSSWLLTTYLDK	putative plastid-lipid-associated protein 3	1,035	1,1398	1,1169	0,41647555	0,4091523	0,35548218
Q9LU85	AT3G26070.1	3	ILGFIPIK,GNLFILK,SITNYQSINVDTLK,GATASPDDQLR,QELLEAIEPLER	putative plastid-lipid-associated protein 4	1,3506	2,1113	1,5586	0,12801913	0,01112212	0,06464314
Q96292	AT3G18780.2	1	AEYDEAGPGIVHR	actin 2	0,53391	0,44876	0,84052	0,03788164	0,00968629	0,21347954
Q941D3	AT5G19940.1	3	GSLFVFR,TGPDDLISTLLSK,EVAQVAGELQK	putative plastid-lipid-associated protein 8	1,1643	1,1216	0,96332	0,2412962	0,26222725	0,41453009
Q8RXR5		1	GYGFVQYANEESAQK	putative poly(A)-binding protein	0,68801	1,5583	2,2649	0,10089738	0,04881936	0,01658271
Q56YB8		1	LGFFLLSEESLLQVLLEIQQNPR,SLNLICAAEDK	putative protein	4,2374	1,3025	0,64288	0,0128681	0,29014286	0,01734302
Q9SU21	AT4G12650.1	3	AEAIQFPVR,ITFTYEVEFVK,MILDNLPALR	putative protein	1,5194	2,7137	1,6201	0,21322366	0,08084458	0,23023138
Q9LYM7		1	LAIEEAISITTTLIAQYEK	putative protein	1,4435	0,20618	0,14283	0,10367477	0,00029818	3,25E-05
Q9LY25		9	TNVATVLLEAGLAK,VPSLEDDFGPEAGEYLHTVTLGSGK,ELLQLEELAK,IAEAHLLEQAER,FMDFGSVFLPS/	putative protein	1,084	1,5961	2,0293	0,07474441	0,01649625	0,00468134
Q9LYD6		13	VLQAPGSSIIYVLGFLHSSEAVVYQIDSK,LVNDLNNVLLR,QLLIGTIGDQILALDK,DAIDGVGIALGK,TPISDLV	putative protein	0,99823	1,4919	1,511	0,47617584	0,09131721	0,11195408
Q67XV5		2	GGPYIGLVTVIATEENAFLR	putative protein	0,99522	0,74419	0,74777	0,48810215	0,10250324	0,12852101

Q9LY69		1	IPGVISASSFDGK,TLVLALATGNK	putative protein	0,97844	1,0392	1,026	0,44539078	0,19026959	0,23990571
Q9M1Z6		1	AAVIAIDLLR	putative protein	0,94379	0,93196	0,98747	0,37591169	0,32903377	0,46793219
Q9SVF0		8	IEQLILATVPDPK,FFDTHLSPFYR,APSIVTDENILLLFDIQQK,IQSLCPTISGNVCCTETQFDTLR,VLNCPYASPS	putative protein	0,91142	1,0543	1,1379	0,37451204	0,26260914	0,21358058
Q9LEV5		1	HEYEGEWSEK	putative protein	0,83098	0,82611	0,99414	0,21322366	0,17190195	0,48516499
Q681I7		1	LIAQNVPWTSTPEDIR	putative protein	0,704	0,53403	0,75856	0,11049341	0,02052223	0,13546214
Q9SVW4	AT4G18070.4	2	ATEIAAEVALAFDEIR	putative protein	0,59897	0,20811	0,34745	0,05507078	0,0003044	0,00626206
Q9SU20		1	ELYQVNMILDNLPALR	putative protein (fragment)	1,0123	1,3181	1,3021	0,46816001	0,11310742	0,14186479
Q8LB07		5	VILASNR,ASELGFFGF,GQQGSYPIK,IIEVDNNVR,FSGNFFVNLLGQWK,GAEFEGAVIALFHMLITK,GTVMI	putative protein transport protein SEC61 alpha subunit	1,3211	1,8715	1,3545	0,14602554	0,04919945	0,21763622
Q0WNI9		1	LTIFDIIK	putative pyrophosphate-dependent phosphofructokinase alpha	1,1188	0,90123	0,80556	0,29525989	0,27632132	0,17307293
Q9SYP2	AT1G20950.1	2	ISTQLSPWSSALFEFLPPFIK,STLLGFLGGSEGLFAQK	putative pyrophosphate-dependent phosphofructokinase alpha	0,65401	0,87798	1,3425	0,08067319	0,24097596	0,12730146
Q9SV68	AT4G13010.1	11	AENLEFMVNLVK,TPEGAALK,RPQEVGAAEAAALPVAGLTALQALTNPAGLK,LANAHVTATCGAR,SNEVCI	putative quinone-oxidoreductase-like protein	0,92451	0,91783	1,0084	0,32416414	0,37834936	0,40289158
Q9AST9	AT1G73110.1	1	IPLILGIWGGK	putative Rubisco activase 2	0,78087	0,54527	0,69828	0,16227175	0,02220782	0,09745137
Q8LA30		3	FFSLLPGEVILASLDGFNK,IVQGENVVAVLASVSDEIQWPLTK	putative senescence-associated protein 12	0,71394	0,50934	0,71342	0,1162738	0,01662694	0,10708804
Q8LC49		2	LVPAGEETELLVGLK,VGVMGIR	putative signal sequence receptor, alpha subunit (SSR-alpha)	0,98202	1,8132	1,8464	0,45471935	0,02346718	0,03423451
Q9FMF9	AT5G64270.1	2	AIGPQDVLATLLNNLK	putative splicing factor	0,79124	1,0946	1,3834	0,17210361	0,29702913	0,113176
Q8LAP5		5	SFDVNKPGYEVDEIK,LQLTAPVCTSK,IDLIQENVAINQHEAIQK,NFVSPPNMIVIR	putative translation initiation factor eIF-2, gamma subunit	0,92872	1,275	1,3488	0,29409355	0,37834936	0,22896149
Q9C6M1	AT1G25520.1	1	AFLTQFFSPIFLK	putative transmembrane protein	1,2077	3,1289	2,4889	0,45449884	0,00639157	0,01398742
Q9SYP1	AT1G20960.2	1	QLGGQPPSIVSGAADEILAVLK	putative U5 small nuclear ribonucleoprotein helicase	1,2227	1,1987	0,98035	0,19877653	0,18057928	0,45058148
Q9FVU8		1	ILFLDVIFPLSLEK	putative UDP-glucose:glycoprotein glucosyltransferase	1,6317	1,7893	1,0966	0,06099168	0,02478731	0,31472934
Q9SIV9	AT2G16430.2	5	YTPQYQWLEEEFPK,VSNIAYNVVNGICTPVK,AVIVSWVTQEAK,NHDGYAVEGDR,YYYVLGVGQTER	purple acid phosphatase 10	1,8662	1,7018	0,91191	0,03827781	0,03089351	0,31472934
Q9M9P3	AT3G03250.1	3	LDTFLSQGK,MTPVSQDVAETK,ASSDLLLVQSDLYTLVDGFVTR,IQTPTDEIVVPYEK	putative UTP--glucose-1-phosphate uridylyltransferase 2	0,95865	0,9227	1,0847	0,13472514	0,47382238	0,1313393
O04314	AT3G16420.3	17	VYVGQAQDGISAVK,NGQPEQAPLR,VLPADPFVINHPDEHLVSVEGWYSPEGIIQGIK,TSDVIGSDEGTHFTI	PYK10-binding protein 1	1,1052	1,163	1,0981	0,475579	0,24939841	0,28192356
Q56ZN6	AT1G78920.2	1	TAQEVVNEVR	pyrophosphate-energized membrane proton pump 2	1,1024	1,2487	1,1328	0,3146579	0,14609819	0,26833844
P31414	AT1G15690.1	19	GFAIGSAALVSLALFGAFVSR,YIEAGVSEHAK,AFIVAFR,AAVIGDTIGDPLK,IATYANAR,AADVGADLVGK,LI	Pyrophosphate-energized vacuolar membrane proton pump 1	1,0425	1,2889	1,2595	0,39110214	0,29340129	0,40324673
Q9LTB2	AT5G49810.1	1	IPFLAYLASVLK	methionine S-methyltransferase	0,53697	0,55263	1,0292	0,0385024	0,02357655	0,43301638
P52901	AT1G59900.1	1	SVESSSQELLDFFR,LVLSHDLATEK	pyruvate dehydrogenase complex E1 alpha subunit	1,2709	1,6395	1,1738	0,16337716	0,11819001	0,44580635
Q8H1Y0	AT1G24180.1	2	SVETSSEEILAFFR,EVDDAVAQAK	pyruvate dehydrogenase E1 component subunit alpha-2	1,1193	2,1203	1,7205	0,22012589	0,01651631	0,05407754
Q38799	AT5G50850.1	4	VLAPYSAEDAR,SNYMSAGQINVPIVFR,LAEEGISAEVINLR,VFVMGEEVGQYQGAYK	pyruvate dehydrogenase E1 component subunit beta	1,6691	2,1658	1,3469	0,08330194	0,00693155	0,0680739
Q9C8P0	AT1G34430.1	3	NQMQVNVTADHR,HPVVNSSCR,NMVESLGVPTFR	pyruvate dehydrogenase E2 component	0,91246	1,803	1,976	0,32194046	0,02384151	0,02747498
Q94KE3	AT3G52990.1	1	IENVEGLTHFDEILQEADGIILSR	pyruvate kinase	1,7218	3,9834	2,3135	0,04823352	0,00069591	0,01568383
Q9FM97	AT5G56350.1	1	ATLIMVLTR	pyruvate kinase	1,2377	1,303	1,0527	0,18736338	0,12035695	0,38489721
O22773	AT4G02530.1	4	WTTLTGLLEQLK,VGQAIENNDLPAAGLVLGK,LSTSPEENTEVEAFNSSLASLITSVNK,GYLQDLVYK	chloroplast thylakoid lumen protein	1,5287	2,0532	1,5361	0,0385024	0,01987707	0,46697019
Q9FK25	AT5G54160.1	3	VILAECILPETPDSSLSTK,VVCDAFGVNLIELLK,SALELDLLEIMAK	Quercetin 3-O-methyltransferase 1	1,5236	1,7539	1,1511	0,08154038	0,02680505	0,24929874
O23207	AT4G36750.1	3	TTATNTGGTTTPAITTTAAK,VPETLSQEVVEQMK,DLEIPEITAAELTAADGFLFGFPTR,GGSPYGAGVFAGD(	Quinone reductase family protein	1,3489	0,80495	0,62753	0,20006354	0,15804466	0,07873399
O24653	AT3G59920.1	6	FQGTSPYIYPLYGLGELPQAFAR,VIGVTSEGETAK,IVCDPSYLPNK,APEHLGASR,TLIHTDVTK	RAB GDP dissociation inhibitor 2	0,93283	1,3726	1,4469	0,16254974	0,19286738	0,07633634
Q9LXC0	AT5G09550.1	1	SPLMGLFEK	RAB GDP-dissociation inhibitor	1,3549	1,4567	1,0751	0,13062538	0,06898957	0,34841671
Q9SEH3	AT4G17530.1	1	AFADELGIPFLETSAK	RAB GTPase homolog 1C	1,6558	1,9329	1,1595	0,0563861	0,02365712	0,33079195
Q9SN35	AT4G18800.1	1	HLVAVQTEDAK	RAB GTPase homolog A1D	0,76889	1,4594	1,898	0,15102512	0,06833032	0,03158833
O49513	AT4G18430.1	1	AQLWDTAGQER	RAB GTPase homolog A1E	1,0381	1,0251	1,0551	0,3767835	0,40744273	0,31708406
P92963	AT4G17170.1	1	GAAGALLVYDITR,AVSTEEGEQFAK	RAB GTPase homolog B1C	0,92184	1,0189	1,1062	0,38846712	0,37107882	0,30218748
Q9FK99	AT5G39620,1	1	IILLGDSGVGK	RAB GTPase homolog G1	1,2063	0,92602	0,79045	0,21184587	0	0
Q9XI98	AT1G49300.2	1	GADCCVLVYDVNSAK	RAB GTPase homolog G3E	1,0397	1,0297	0,99035	0,4105179	0,41683144	0,47551442
O49841	AT5G03530.1	1	ILLIGDSGVGK	RAB GTPase-C2A	1,2063	0,92602	0,76768	0,21184587	0,31921491	0,14159103
Q9SF92	AT3G09910.3	1	ILLIGDSGVGK	RAB GTPase-like protein C2B	0,94281	0,74524	0,79045	0,37451204	0,10345363	0,15788845
Q9LS94	AT3G18820.1	1	VGTNVEEAFQCIAK	Ras-related protein Rab-7A	0,98337	0,83991	0,91627	0,16254974	0,1662079	0,39109031
Q9FJF1	AT5G59840.1	1	NIEQHASDNVNK	Ras-related protein Rab-8A	1,5416	1,0158	0,65888	0,07772994	0,45436924	0,07599149
Q9FK68	AT5G45750.1	1	HLVAVQTEDAK	Ras-related protein RABA1c	0,77083	0,91022	1,1808	0,15282694	0,29114832	0,22201574
O04486	AT1G09630.1	1	EGQTIDVAATSESNAK	Ras-related protein RABA2a	0,72685	1,0768	1,4815	0,12230158	0,32406999	0,08303118
Q9C4Z6	AT1G48630.1	2	NTLAGHSGYLNTVAVSPDGSLCASGGK,LWDLATGESTR	receptor for activated C kinase 1B	0,90267	2,4132	2,6734	0,30758306	0,00646224	0,00844301
Q9LV28	AT3G18130.1	3	LWDLATGETTR,NSLVGHSGYLNTVAVSPDGSLCASGGK,AHTDIVTAIATPIDNSDIIVTASR	receptor for activated C kinase 1C	0,937	1,9645	2,0965	0,36464091	0,01662694	0,02145069
Q9SN38	AT4G18760.1	3	LPDSLHSFTAVSSFLR,NTISGDIPDSIGDLISLK,GSIPTSITLLSNLK	receptor like protein 51	0,67344	0,54272	0,80589	0,09254201	0,02168291	0,17307293
Q9SCZ4	AT3G51550.1	2	YPTGTPTYVAPVDVYSTAR,LNVGGNDISPSADTGLYR	receptor-like protein kinase FERONIA	0,89684	0,92632	1,0691	0,30758306	0,38074277	0,37380535
Q9SSB5	AT1G53750.1	1	FDDGVGGDNEVQR	regulatory particle triple-A 1A	1,332	1,7227	1,3239	0,14224503	0,074121	0,33358684
Q9SEI4	AT5G58290.1	2	ENAPAIIFIDEVDAIATAR,FDAQTGADR	regulatory particle triple-A ATPase 3	0,77765	1,1501	1,5739	0,06856897	0,30054528	0,0445388
O80837	AT2G45820.1	2	VDVESPAVLAPAK,EPTPAPVEVADEK,AAVEAQLR	Remorin	0,87358	0,70518	0,79596	0,41210417	0,14602234	0,20220305
Q9M2D8	AT3G61260.1	1	AAVEAQLK	remorin-like protein	1,0136	0,66867	0,65969	0,46504043	0,0599805	0,07633634
B9DFM8	AT4G20830.1	3	ASVVALFLGR,IGLVFNPYGGK,TLNFLLK	Reticuline oxidase-like protein	1,2952	1,2415	0,98508	0,17793438	0,15733402	0,45518062
Q42586	AT3G54470.1	1	DQTCLIIEDLVTSGASVLETAAPLR	orotidine 5'-phosphate decarboxylase	0,53952	0,48343	0,89604	0,0390965	0,01319151	0,28820503
Q9SH59	AT1G64090.1	2	GAFAFIK,VDDFGEK	reticulon-like protein B3	1,1232	1,0876	0,9683	0,28995258	0,30624202	0,42538439
O82352	AT2G46170.1	1	TSPQIPEIHVPEEAFLVVASSLR	reticulon-like protein B5	0,99621	1,2555	1,2603	0,49105726	0,14192721	0,16012956
Q9SS37	AT3G10260.2	1	GLLFLQDLACK,SADVLLWR	reticulon-like protein B8	1,0211	0,67591	0,63859	0,3847187	0,00639157	0,01568383
Q39099	AT2G06850.1	5	TGQPAILQTNVFTGGK,DLDAEQWR,GFHIDGCQASVEAK,WTIYNYCTDR,IYLWFDPSK,TNWANAPFVAS	xyloglucan endotransglucosylase/hydrolase protein 4	0,53895	0,36719	0,68338	0,0390965	0,00558934	0,12344714
Q8LB19	AT5G15650.1	4	NLLCPSSPFFFNTLYDPYR,GIFWQEEIIPFFQNAK,IHVPEGYDYELYNR	reversibly glycosylated polypeptide 2	1,6532	1,8371	1,4138	0,49784913	0,013676	0,02369746
Q0WWT7	AT4G24750.1	4	LAGIGGFSEFLGWTDQQR,GSTWVPIFDNDDNLDAGTLSK,DSELIVACQK	rhodanese homology domain-containing protein	1,0139	0,91297	0,90047	0,46447374	0,29610372	0,29405411
Q8RY72	AT3G08920.1	2	VSEGDFPEIEGTEELR,LLVLLPSFGQK	rhodanese-like domain-containing protein	1,3844	2,0322	1,468	0,11940032	0,01421162	0,08601586
O48529	AT2G42220.1	4	TVHNNFSGLFFGLPFTK,SCSHIPLFIYNEDNDIGTIIK,LLLVCQEGLR,LEEAGYENIACVTSGLQSVKPGTFESV(	rhodanese-like domain-containing protein	1,2632	2,0261	1,5333	0,28995258	0,05630528	0,12540988
Q9T092	AT4G27700.1	5	NVFHLEGGIYTWGK,IIVACSSAGTMKPTQNLPEGQQSR,SLIAAYLLVLNGYK,EWTAWDIAR,ENNFVILDVF	rhodanese-like domain-containing protein	1,0497	1,6961	1,6721	0,25128951	0,07495033	0,17416837
Q9SFX3	AT1G76400.1	5	FLGNNLAYLSVAPSEGK,GDTLTLEVVAAFTNVLQPFPEK,TFFTIGYGLPLTDFLFASEGK,VVNSGSESVSEFAL	Ribophorin I	1,1	1,6413	1,5565	0,33667925	0,12530114	0,1072668
Q9ZUA0	AT2G01720.1	5	NLAMVQALATTGK,TYLPLDVKPTEQPDAPNDTGYYR,VESFTSIEPANR,YSYLDIVGR,AFLTLK	Ribophorin I	0,84223	1,3114	1,579	0,39394212	0,09077271	0,09313267
Q93Z16	AT4G21150.3	16	VLQSSSSTLK,GLTSFAASESTGLNLPGDK,VYITEAQTK,SVDASQGPISTTASVIR,VPIAATGAISIENAEIAVLDS	ribophorin II (RPN2) family protein	1,0197	1,1784	1,1296	0,33467429	0,04259255	0,08383041
Q680A5	AT2G42910.1	3	FYFADQVLPLFETGIPLLTK,VIVAFPDDGAWK	ribose-phosphate pyrophosphokinase 4	0,71271	1,4911	2,0922	0,11568153	0,06091412	0,02145069
Q8RWT4	AT2G42710.1	1	AAGADVVGGLELIEEILK	ribosomal protein .1/L10 family protein	1,6141	3,269	2,5137	0,14408762	0,00185623	0,01197314
P56791	ATCG01310.1	2	IVTIEYDPNR,GAIIGDTIVSGTEVPIK	ribosomal protein L2	0,81698	0,5466	0,69761	0,10815991	0,01895266	0,12993994
P56796	ATCG00640.1	1	HTIHGEIK	ribosomal protein L33	0,86486	1,1429	1,3214	0,24982453	0,23718685	0,13380921
P56802	ATCG00750.1	1	SGILLSFVR	ribosomal protein S11	0,84696	0,44429	0,52457	0,22760233	0,00931231	0,03104402
P56807	ATCG00650.1	1	ILSLLPFLNNQK	ribosomal protein S18	1,1522	1,1137	0,96656	0,25460184	0,27146028	0,42129325
P56797	ATCG00160.1	2	AGVHFGHGTR,FLSEACDLVFDAASR	ribosomal protein S2	0,84904	0,56353	0,66373	0,23033777	0,02569306	0,07811348
P56798	ATCG00800.1	4	VPLQTIEAK,ISNPYGDPNILAEFIAGQLK,AIELTEQANTK,LNIAITR	ribosomal protein S3	0,78432	0,70933	1,0469	0,21184587	0,02080735	0,07150049
Q9M1X0	AT3G63190.1	1	AIVNSDLGVTPNNDGDVIR	ribosome-recycling factor	1,5873	1,7949	1,1308	0,06789459	0,0244066	0,27044118
P10795	AT1G67090.1	4	EHGNSPGYYDGR,QVQCISFIAYKPPSFTG,EYPNAFIR	ribulose bisphosphate carboxylase small chain 1A	0,76703	0,80132	1,4702	0,36226386	0,46522724	0,37598399
P10796	AT5G38430.1	2	QVQCISFIAYKPPSFTDA,FETLSYLPPLDTDVELAK	ribulose bisphosphate carboxylase small chain 1B	1,1164	0,68808	0,61933	0,29525989	0,08538335	0,06904298
P10797	AT5G38420.1	2	FETLSYLPDLSDVELAK,QVQCISFIAYKPPSFTEA	ribulose bisphosphate carboxylase small chain 2B	0,64507	0,33385	0,51754	0,07559385	0,00264297	0,0297318
Q3ECU4	AT1G48600.2	2	SAETPSPEFAEYIK,AGEVIALDFIESAIQK	putative phosphoethanolamine N-methyltransferase 2	0,58221	0,56281	0,97105	0,0390965	0,02319918	0,46230043
Q3EBJ5	AT2G39730.3	10	SFQCELVMAK,VPIICTGNDFSTLYAPLIR,GLAYDTSDDQQDITR,LVVHITK,FYWAPTR,NFLTLPNIK,VPLILG	ribulose bisphosphate carboxylase/oxygenase activase	0,63583	0,56303	0,90455	0,0753558	0,03444647	0,35101078

O03042	ATCG00490.1	28	DTDILAAFR,GGLDFTK,TFQGPPHGIQVER,ALAALR,DDENVNSQPFMR,LEDLR,SQAETGEIK,DNGLLLHIH	ribulose-1,5-bisphosphate carboxylase/oxygenase large subunit	0,74014	0,51193	0,70294	0,18917409	0,03089351	0,11455509
Q9C9I7	AT1G71500.1	9	AAVIGFLLLLDFELLTGK,AQPGLTATNVNVDEVR,GTGFLDFLYSASDAFK,LTQDGCIVCPSTDSTFDLR,VVIC	Rieske (2Fe-2S) domain-containing protein	1,1859	0,94685	0,79704	0,30148919	0,20850578	0,14369822
Q39209		2	VYVGNLPWDVDNGR,LEQLFSEHGK,AEFPEPSEEAK	RNA binding protein	1,397	0,89128	0,65074	0,1681456	0,2595879	0,09816721
Q9SA52	AT1G09340.1	18	QLPGESDQDFADFSSK,SSLSAEGFDVVYDINGR,SDILPHCEEDAVDPK,DLATAFLNVLGNEK,LETESLLQSK,	RNA binding protein	0,77201	0,5659	0,70791	0,16588096	0,09223695	0,34555977
Q2V4R5	AT1G01080.2	1	IAIASLDGTEVGGR	RNA recognition motif-containing protein	1,9754	1,2673	0,64157	0,03241734	0,1357703	0,06904298
O82299	AT2G35410.1	2	EEAENAITK,SPNDLPSPAPGDTR	RNA-binding (RRM/RBD/RNP motifs) family protein	1,0365	0,72589	0,76843	0,30758306	0,10935482	0,2064367
P93042	AT3G13870.1	5	ALDAGEGFSSSAK,STLLNHLFGTNFR,GCEEAVIEQAK,LLQLVQPTFQDVLGHLR,NETEYTVTQAISAQEANI	Root hair defective 3 GTP-binding protein RHD3	1,2862	1,5121	1,1741	0,18146812	0,07190364	0,24204577
Q94B78	AT4G33010.1	7	GNADVQNNVLK,GNINIEEVR,LGVAEVQELPFFDTVK,ATSNICTAQALLANMAAMYAVYHGPAGLK,IIGIS\	glycine dehydrogenase [decarboxylating] 2	0,44161	0,41375	0,87187	0,03918435	0,01851274	0,40957572
Q93VH5	AT5G10730.1	4	FVYISAADFGLANYLLR,DALEGVTSVISCVGGFGSNSYMYK,FAYGGIILRPGFIYGTR,SSLQESWASR	Rossmann-fold NAD(P)-binding domain-containing protein	1,6092	1,6517	1,0264	0,06347689	0,03625266	0,43816765
Q8H0U5	AT3G18890.1	7	AGSLVQSVK,VNNFILVTSLGTNK,RPYVPPPK,IVEVVAETTAPLTPIEK,LQNTDEGTQPVEK,EEDLVFVAGAT	Rossmann-fold NAD(P)-binding domain-containing protein	1,434	1,4904	1,0442	0,0822534	0,05301688	0,48278577
O65502	AT4G35250.1	10	YLQDYFSNILK,AWTTQEVITLCER,LLTFAGPR,ALDEGYDVR,LCGFMQGLIGQYAVPILEEK,LTLIALR,FFQW	Rossmann-fold NAD(P)-binding domain-containing protein	1,1492	1,2619	1,1176	0,17677818	0,3081625	0,24275995
Q8L9T6	AT4G23430.2	3	YFNPYLAVAVGAVAK,LCNVLHANELTK	Rossmann-fold NAD(P)-binding domain-containing protein	1,0398	0,89031	0,85624	0,4105179	0,26037722	0,23398863
Q9SZ91	AT4G09750.1	1	TSEQGADTIVWLALQPK	Rossmann-fold NAD(P)-binding domain-containing protein	0,52416	0,76348	1,4566	0,03554246	0,1167307	0,08876987
Q9FEF8	AT5G52470.1	1	ILALNASFFLK	rRNA 2'-O-methyltransferase fibrillarin 1	0,44275	0,84577	1,9103	0,01870364	0,19494297	0,03075562
Q94AH9	AT4G25630.1	1	ILALNASYFLK	rRNA 2'-O-methyltransferase fibrillarin 2	0,84973	2,2301	2,936	0,37591169	0,00320596	0,00848737
Q9FYF7	AT1G67360.2	3	FFVVTNSVK,AAFNYAATEYK,VEGAVTTVVTPVYQK	Rubber elongation factor protein (REF)	1,5781	4,4748	2,8356	0,07029693	0,00038756	0,00652833
B9DFS9	AT1G55490.2	1	ETSTIVGDGSTQDAVK	RuBisCO large subunit-binding protein subunit beta	1,4151	1,2593	0,89122	0,11609178	0,11310742	0,36785181
Q9FFJ2	AT5G17170.1	3	QGGGVVITGVDGGGNAAK,SFDEQPDTYVCPQCIAPK,ATHICLDCGFIYTLPK	rubredoxin family protein	1,7345	1,0391	0,5991	0,04768595	0,39518157	0,0510789
Q9SLI4	AT1G54500.1	3	YDESAGDPSYPIPPGFQFDK,CPTCGAAQSFFESK,FAVLNTGIYECR	rubredoxin-like protein	1,0066	1,083	1,0754	0,23365741	0,29797719	0,35067786
Q94BZ0	AT1G50450.1	3	LGENSEFSQVDINDAK,DVYLLNLPEVR,FSYYTAGTGGAGPTILATSFLLLGEEVTAYK	Saccharopine dehydrogenase	1,4881	1,4068	0,94538	0,09182467	0,08150801	0,37602812
Q8LGI2	AT5G39410.1	9	GPSEEEVESATFK,SLALAGR,SLFGIFR,IAGNFGTYESAVLGVANAEK,LPSADAVVVR,GYSEESLASQGETKPI	Saccharopine dehydrogenase	1,1753	1,3233	1,1342	0,28959084	0,12897321	0,25850568
Q52T38	AT5G20350.1	1	LLLFLDAYR	S-acyltransferase TIP1	1,3228	2,2694	1,7156	0,14224503	0,00890638	0,04560558
Q5D6C4		1	TGQVPDPTSTDNPEFQIVLSIIK,TEFGPSQPFK	S-adenosyl-L-homocysteine hydrolase 1 mutant	1,4778	1,0716	0,80728	0,49496207	0,43662031	0,45058148
Q5D6C3		2	LVGVSEETTTGVK,ALDWGPGGGPDLIVDDGGDATLLIHEGVK	S-adenosyl-L-homocysteine hydrolase 1 mutant	0,80525	0,50562	0,6279	0,18561901	0,01634816	0,06300224
Q9SJL8	AT2G36880.2	2	TQVTVEYK,SVVAAGLAR	S-adenosylmethionine synthase 3	1,2616	1,333	1,0565	0,17297932	0,10735207	0,37789765
Q9LUT2	AT3G17390.1	2	SIVASGLAR,ANVDYEQIVR	S-adenosylmethionine synthase 4	1,146	1,2502	1,1106	0,3299506	0,15211344	0,26280471
Q9SI32	AT2G04940.1	1	RPFVASITDALGNELFR	scramblase-related protein	1,1469	1,9546	1,7042	0,26069976	0,01707463	0,04671641
Q9SFU0	AT3G07100.1	1	FLPLYGLAITK	sec24-like transport protein	0,59414	0,7144	1,2024	0,0523846	0,08354731	0,20220305
Q9LPC5	AT1G01960.1	1	APLVVAAIQTLGNMGESLFK	SEC7-like guanine nucleotide exchange protein	0,55477	0,78643	1,4176	0,04293598	0,13359262	0,10188027
Q9M5P2	AT1G61250.1	3	LSPLPPEPVGFDYGR,YDPNPFAEEEEVNPFANAR,AGIVIEVK	secretory carrier-associated membrane protein 3	0,94134	0,94413	1,003	0,3724232	0,35467868	0,4918964
Q9LSE4	AT3G26710.1	1	QVAGEILSFFTR	cofactor assembly of complex C	1,8275	2,401	1,3138	0,04044006	0,00663084	0,13614308
Q39218	AT3G13110.1	1	LSNLNLPSNTLFDLFSGVLQGNPDIVESVK	serine acetyltransferase 3	0,95348	1,5469	1,6224	0,3936627	0,05048437	0,05824542
Q3E923	AT5G26780.1	1	LDENTGYIDYDQLEK	serine hydroxymethyltransferase 2	2,9234	5,9935	2,0502	0,00641132	7,73E-05	0,02371304
O23254	AT4G13930.1	1	LLICGGSAYPR	serine hydroxymethyltransferase 4	0,84071	0,8585	1,0212	0,2218635	0,21044256	0,44849848
Q94IB8	AT4G36480.2	1	VVLFGVPIR	serine palmitoyltransferase	1,0997	1,3935	1,2672	0,31679829	0,08542725	0,15741367
O23601		3	EPPQSTPAVK,NLFDETTLYDK,WINGTWDLK	hypothetical protein	1,8203	1,6673	0,91593	0,04125134	0,03444005	0,32227833
A1L4Y1	AT3G10350.1	1	LLSLPDFLDASIGK	anion-transporting ATPase family protein	0,5503	0,57398	1,043	0,04139894	0,02758077	0,40522387
Q56YA5	AT2G13360.2	10	HHLFVPGPVNIPEPVIR,YNLSLGLGLNK,LGTYWPYTPSIQLLYGLR,VFFDWNDYLK,ALSLPTGLGIVCASPK,I	serine--glyoxylate aminotransferase	0,62398	0,47993	0,71767	0,0507979	0,00692255	0,1072668
Q39230	AT5G27470.1	2	LPYQIVSIVSGALNDAAAK,FASVEIVDEIIK	seryl-tRNA synthetase	1,1617	1,0799	0,92958	0,24294789	0,31921491	0,34755475
P58684	AT2G39960.1	1	DGVLVEGLFWK	signal peptidase complex subunit 2	0,72145	0,70294	0,97434	0,11932159	0,077212	0,43816765
Q9SSR2	AT1G52600.1	1	ENTGEVDVLTK	signal peptidase, endoplasmic reticulum-type	1,3786	1,4497	1,0516	0,12161525	0,07071045	0,38674952
Q9C9C0	AT1G73990.1	2	NAWEIFVVK,FNSGLSLPQLSENFVK	signal peptide peptidase	1,0009	1,7308	1,6694	0,28224382	0,01758657	0,04560558
O65253		2	IEALIQFILEQGASK,LNLVFTLLSK	similar to aminoacyl-tRNA synthetases	1,4792	1,4932	1,1028	0,18877558	0,08232493	0,26397186
P93818		1	YGLTPVELALGFVR	Similar to Babesia aldo-keto reductase (gb|M93122)	1,0593	1,2916	1,2193	0,37599422	0,12530114	0,18838476
O64796		1	LPFTIDIPFISGIK	Similar to mannosyl-oligosaccharide glucosidase gb|X87237 fror	0,90237	1,3086	1,4502	0,30758306	0,11738917	0,09091872
O04471		1	LFLLGIGPALR	Similar to Saccharomyces hypothetical protein P9642.2 (gb|U4C	1,7783	2,7853	1,5347	0,06099168	0,00692025	0,08876987
O80977	AT2G14740.2	1	VCECPTVDGVQFK	vacuolar-sorting receptor 3	0,55033	0,58601	1,0648	0,04139894	0,0307626	0,36629974
Q9XIR5		1	NPEISSLVPTLLLALTDPNEYTR,NPDTISQISDLLSPLIQLVK,VLPLIIPILSK	similar to translational activator	1,1819	2,0272	1,6786	0,17691773	0,00931231	0,03772105
B3H5K3	AT5G47690.3	1	VVGLVGELFSLPGR,AGVDDLLGILK	sister chromatid cohesion protein PDS5	0,93942	1,499	1,4467	0,20343289	0,3686857	0,1527779
Q93VC7	AT5G30510.1	7	FVEVDEEQTK,MFEDAYER,IAQAEAMAR,SSAYLSVEQACIHR,VSDIATVLQPGDTLK,GGLVALVEGLR,NIQ	small subunit ribosomal protein S1	1,3059	0,6868	0,53337	0,23819306	0,0631033	0,04475628
Q42343		1	TLGLVLLR	SnRNP	0,5422	1,3284	2,4499	0,0390965	0,10904609	0,01225561
Q1EBV7	AT2G26900.1	1	FSFGESTSR	Sodium bile acid symporter-like protein	1,012	1,404	1,3873	0,46816001	0,08227413	0,11195408
Q0WVZ5	AT5G27150.1	1	AAFVFPLSFLSNLAK	sodium/hydrogen exchanger 1	1,018	1,0652	1,0463	0,45504834	0,34358374	0,39884546
Q8VZ90	AT5G20140.1	2	FCSHLDLWDSIK,NNDYFSLEGLVDVFK	SOUL heme-binding protein	1,4391	1,2927	0,89825	0,10545331	0,12502722	0,29173895
Q9LVW0	AT5G54100.1	2	LASYGVENPIYAVMQLAQTTMR	SPFH/Band 7/PHB domain-containing membrane-associated prc	2,557	23,724	9,2778	0,0106699	7,50E-09	6,88E-06
Q9C9A4		1	SVLLAPSEIVDVLVDFSK	spore coat protein-like protein	0,88046	1,0888	1,2366	0,27394655	0,30531068	0,17553034
P53799	AT4G34640.1	2	SFSLVIQQLNTELR,YPDDIYPLLK	Squalene synthase	1,3455	2,1026	1,5627	0,13337707	0,012231	0,06850123
O81814		2	SLDLTIISAEDLK,LTVDDAAAR	src2-like protein	1,4558	2,7045	1,8577	0,10012196	0,00367439	0,03345105
Q9SUQ9	AT4G23680.1	1	VYDVVYQFIPK	SRPBCC ligand-binding domain-containing protein	1,4619	1,7054	1,1666	0,09906164	0,03084205	0,235489
Q9SUR0	AT4G23670.1	4	SENHVFADAIGHHIQNVVVHEGEHDSHGSIR,NDDFPEPSGYMK,VFDIIYEFIPK,GLDGHVMEHLK	SRPBCC ligand-binding domain-containing protein	0,86038	0,50683	0,73661	0,20302221	0,04281202	0,13576487
P94111	AT1G74020.1	7	FLDGLDVDPTTGVVYFTSFSSR,SGPEAFAFDSTGK,AGSSEDFTNSVSNPDNIK,IGSTGNFWVASVVNK,ITDS	strictosidine synthase 1	0,88292	1,606	2,0762	0,11399376	0,03097873	0,2004875
Q9M1J6	AT3G57020.1	5	SFLITCESGTSLVHR,ISGETGEVLELLEDK,LWFGSVYWPAVWVLDR,TIPIPVAGPESIEFDPK,GEGPYAAVVD	strictosidine synthase family protein	0,83234	0,54962	0,66033	0,21404153	0,02317759	0,07653079
Q9SD07	AT3G51420.1	3	FLTGAEFIGVGLLNNPEDIAYHK,YDGDGHYWIALISEVTTSWK,LTDAVTVADNGVLYFTDASSK	strictosidine synthase-like 4 protein	1,1048	0,51773	0,46862	0,311508	0,01792803	0,02029856
O04485		1	SPLSVNEIIYALPMLTILTNK	Strong similarity to S. pombe leucyl-tRNA synthetase (gb|Z7310(	1,0928	3,0926	2,83	0,32669256	0,00243954	0,00652833
Q9ZUF6	AT2G05920.1	6	GVFVSCSAGNSGPTR,EVTNVGAASSVYK,GTVLDVKPSPVVAAFSSR,DVDGHGTHTSTTAAGSAVR,DAGGI	Subtilase-like protein	1,426	2,0442	1,4289	0,11932159	0,02593261	0,1545229
Q84WS0	AT1G01900.1	1	DAQGHGTHTASTAAGDIVPK	subtilase-like protein	0,87125	1,2038	1,3817	0,25973442	0,17661713	0,11381838
P53492	AT5G09810.1	1	LAYVALDYEQELETAK	actin 7	0,6687	0,3989	0,63127	0,04205447	0,00389786	0,08383041
O82663	AT5G66760.1	12	NSNGSLPTSTIR,TQETLEEGCQLIDK,HTLGYWEDEK,TGHALLHTLYGQAMK,AVIELENYGLPFSR,AGLPLQ	succinate dehydrogenase [ubiquinone] flavoprotein subunit 1	1,5207	2,6216	1,8533	0,16029952	0,00603337	0,03052763
Q9XFM6	AT5G52240.1	1	SDDAPAETVLK	membrane steroid-binding protein 1	0,55566	0,53678	0,96602	0,04306948	0,02067516	0,42018922
Q8LAD2	AT5G23250.1	1	FFVDPQTEGIVLIGEIGGTAEEDAAALIK	Succinyl-CoA ligase [GDP-forming] subunit alpha-2	0,89004	1,1767	1,322	0,28995258	0,19996677	0,13377983
O82662	AT2G20420.1	1	AILVNIFGGIMK	Succinyl-CoA ligase [GDP-forming] subunit beta	1,2756	3,0128	2,3619	0,16526184	0,00259189	0,01400248
Q39232	AT1G71880.1	1	AFLADLAAGDAK	sucrose transport protein SUC1	1,3767	2,8235	2,0509	0,12191578	0,00319329	0,02371304
Q39231	AT1G22710.1	2	EVYGGNSDATATAASK,ASNVPFFGEIFGAFK	sucrose transport protein SUC2	0,76892	0,87787	1,1417	0,15102512	0,24095013	0,25837114
Q39228	AT3G19930.1	1	AGGFVSQTPGVR	sugar transport protein 4	1,1005	1,4923	1,356	0,31578971	0,06064588	0,12294256
P23586	AT1G11260.1	2	PAGGFVVGDGQK,GVDDVSQEFDDLVAASK	sugar transporter 1	0,91789	0,33984	0,37024	0,33099788	0,00271908	0,00824639
Q93YP9	AT1G19450.1	1	TLEEIQALFR	sugar transporter ERD6-like 4	1,0481	1,0345	0,9877	0,28627108	0,27557623	0,46388171
Q9FRL3	AT1G75220.1	1	TLEELQSLFR	sugar transporter ERD6-like 6	1,1013	1,1233	1,0273	0,24063742	0,24522237	0,43784546
Q9FY46	AT5G13550.1	1	TLLPTSALITGVAILESVGIAK	sulfate transporter 4.1	0,65633	0,80852	1,2319	0,08154038	0,15404108	0,1782611
Q9LZ66	AT5G04590.1	1	ITPNQNIVLCDIK	sulfite reductase	1,5562	1,9063	1,247	0,07123949	0,01961879	0,20293002
Q9SKR2	AT2G20990.1	9	TLVVPILDPAK,RPVGIVHVK,FLEYMWPYLDK,AFTLELR,DPQTQVLEFSVYDWEQVGNPEK,GFEETQAVQI	synaptotagmin A	0,79067	0,82378	1,0614	0,25951247	0,26555968	0,43816765
Q3E7R5	AT3G11820.2	1	GGTDQLQTAR	syntaxin-121	1,3225	1,271	0,96108	0,14224503	0,13387106	0,41046922
Q9ZSD4	AT3G11820.1	1	VLDTINEIQER,GGTDQLQTAR	syntaxin-121	1,2447	1,3057	1,1403	0,2218635	0,11197347	0,29277568
Q8VZU2	AT5G08080.1	1	ENIQQEYR	syntaxin-132	1,2971	0,93625	0,72183	0,1528316	0,33893255	0,11243933
Q9SF29	AT3G09740.1	1	LYGAFETQIETALEK	syntaxin-71	0,65646	0,58017	0,88379	0,08154038	0,02913383	0,27013697

Q9LN91		1	IEDILPFFPDFALIDDFK	T12C24.2	0,54641	1,1935	2,1843	0,04040215	0,18509878	0,01919518
Q9MAM6		5	EEGLELYEDMILGR,QSTNNTSLLITK,DHVHALSK,GFGIGPDGR,LPIIFVVENNLWAIGMSHLR,GHSLADPDI	T25K16.8	0,88173	0,61614	0,75777	0,09951543	0,04557805	0,33658501
Q5E917	AT3G20050.1	4	IACLDFNLQK,AGANVILTTK,LISGDSDFFANLVVEAVLSVK,EQLAIAEFADALLIIPK,TLESNTVVAGGGAVES	T-complex protein 1 subunit alpha	0,85185	0,89285	1,0892	0,08453135	0,06353359	0,44656466
Q0WRG9	AT5G56500.2	1	DIISILEDAIK	TCP-1/cpn60 chaperonin family protein	1,0672	1,2328	1,2166	0,33941417	0,1854632	0,30776669
Q9SF16	AT3G11830.1	7	DSFLVDGVAFK,SQDSEVGDGTTTVVLLAAEFLK,LAIGDLATQYFADR,VAEEDLNR,VAAAAGGTVQTSVNN	TCP-1/cpn60 chaperonin family protein	1,0178	1,1542	1,2007	0,35171676	0,12253519	0,10811884
Q9LV21	AT3G18190.1	3	VVSQYSTLLAPLAVDAVLSVIDPEKPEIVDLR,GSNQLVLDEAER,ATGCNVLLIQK	TCP-1/cpn60 chaperonin family protein	1,0001	0,90777	0,90769	0,49967239	0,28672085	0,30776669
Q84WV1	AT5G26360.1	1	VPGGQFEDSEVLK	TCP-1/cpn60 chaperonin family protein	0,9026	1,4628	1,6206	0,30758306	0,06786834	0,05825772
Q94K05	AT3G03960.1	4	GTVLIHSAEQLENYAK,TLADNAGLNAMEIIAALYTGHGSGNTK,YAESFEFVPK,NPTNFNVDNVR	TCP-1/cpn60 chaperonin family protein	0,84274	1,9111	2,2677	0,22262747	0,01878338	0,01658271
Q9SVU4	AT4G28770.1	1	DLPYDPTGELNSLR	Tetraspanin family protein	1,0848	1,1725	1,0809	0,33667925	0,20362871	0,33958309
Q9M1E7	AT3G45600.1	2	AGVLGSLK,IDNDEPAGEAR	tetraspanin3	0,84561	0,69869	0,80915	0,23526536	0,06795903	0,16928349
Q8S8Q6	AT2G23810.1	2	AGLLDNVK,LGDYSTWLQK	tetraspanin8	1,6125	1,2728	0,78931	0,06347689	0,13279871	0,15741367
Q9FHY8	AT5G41950.1	1	AIYNLGTVLYGLAEDTLR	tetratricopeptide repeat domain-containing protein	0,96123	1,2858	1,3379	0,3847187	0,1318416	0,12673697
Q8VZM1	AT1G80410.1	5	VLEAERPSISQLQNK,HEDSLVHR,LGAENPDSHR	tetratricopeptide repeat-containing protein	1,173	1,7854	1,7272	0,172787	0,02286788	0,01568383
Q9FI37	AT5G48010.2	2	SFLQDLFWK,SQITENPR	thalianol synthase 1	1,641	1,2943	0,78868	0,0599686	0,12420678	0,15739971
Q38814	AT5G54770.1	1	LFNAVAAEDLIVK	thiazole biosynthetic enzyme	0,91422	0,48414	0,52957	0,32488037	0,0132838	0,03184562
Q940I2	AT5G03880.1	1	YGDGTVPLSLSLGALTAITAGFAMIGR	thioredoxin family protein	1,7903	0,23271	0,12999	0,04306948	0,00047295	1,94E-05
Q42403	AT5G42980.1	2	HLDVVFFK,FIAPVFADLAK	thioredoxin H3	1,2857	1,5344	1,2484	0,42165346	0,0928153	0,13180052
Q39241	AT1G45145.1	1	IDVDELQAVAQEFK	thioredoxin H5	0,75754	1,0225	1,3328	0,11568153	0,31791102	0,14369822
Q9SEU6	AT3G15360.1	1	INTDESPNTANR	thioredoxin M4	0,58728	0,948	1,7298	0,04741018	0,24575719	0,07359494
Q39242		1	FGTTIFTETVNK	Thioredoxin reductase 2	1,2326	1,2616	1,0235	0,18992425	0,13839266	0,44412223
Q94CD9		6	IIYTSSFFALGSTDGSVANENQVHNER,LPGYIGSGTDR,MALNAASEGVPIILLYPGVIFGPGK,LPLISPPTVTVL	unknown protein	1,7983	2,4286	1,3505	0,04306948	0,00639157	0,12438478
O23166	AT4G37200.1	1	EGNEEGNVVGR	thioredoxin-like protein HCF164	1,102	1,2118	1,0996	0,3146579	0,17124124	0,31082125
O04630	AT5G26830.1	6	SIEDLLEEFK,EAQPFER,NEASGALSGLTR,GEVQGVLEFIDYVYK,WPFWISPR	threonyl-tRNA synthetase	1,7396	1,8202	1,1428	0,11810843	0,00692025	0,04671641
Q9M024	AT5G01500.1	1	TQSSLLTAVVAAAIATGTCYPLDTIR	thylakoid ADP,ATP carrier protein	2,2904	5,0019	2,1839	0,01746757	0,00025441	0,01919518
Q9ZVL6	AT1G54780.1	7	LVAAIDGQPDPGGPTVK,ADAFEYADQVLEK,LNFITVR,WYPSIEEGNNK,EGAITGGPAFIEAVGENILDATVS	thylakoid lumen 18.3 kDa protein	1,2604	1,6419	1,2469	0,27231812	0,31761073	0,38652388
Q0WLK8		1	DVILNNLALSDVELQDLIAGADK	Thylakoid lumenal 19 kDa protein, chloroplastic	2,0873	1,218	0,58352	0,02516926	0,16688934	0,04581427
Q8LCA1	AT2G46820.2	4	NLVFKPDR,TAQEAWEK,ATTEVGEAPATTTEAETTELPEIVK	thylakoid membrane phosphoprotein	1,72	2,5188	1,468	0,06347689	0,00632404	0,07791112
Q9M158	AT4G01050.1	6	QVDEFLNTK,GWLNSSLPWIEPK,AVSTVYNGEDKPGFLK,LGTDDNAQLLDIR,FDGNSELVAELVALNGFK,A	thylakoid rhodanese-like protein	1,2998	1,6677	1,2709	0,18621086	0,05672548	0,18869288
Q9SZB2	AT4G33350.1	1	VVPITLDQVYLLK	Tic22-like protein	1,3633	1,0651	0,78125	0,12801913	0,34358374	0,15236678
Q2V409	AT2G42210.2	3	GSITGFGAGTIYGTILATWK,DFYNGAIGGFVAGASVLGYR,SIPTAIAAGATLAVTSALIDSGGQTTR,NVALPGI	Tim17 domain-containing protein	1,0244	1,0126	0,9492	0,39151124	0,12372323	0,17723399
Q9C5W7	AT1G32400.3	1	TGNFDTIYNFLR	tobamovirus multiplication 2A protein	0,82162	1,1495	1,3991	0,20230136	0,22817556	0,10738259
Q3E7E9	AT5G44920.2	1	IALAIFSK	Toll-Interleukin-Resistance domain-containing protein	1,0705	1,2483	1,166	0,36165493	0,14624026	0,23559881
Q8GXY6	AT1G05270.1	1	IISSVAVAVAGTAIVSGILLSR	TraB-like protein	3,2381	0,32631	0,10077	0,00444389	0,00244126	4,87E-06
Q9LTQ5	AT3G20370.1	13	GYLYDGDHCEFGVDVTMPSLYEK,DRPPSSYSLK,NDVLMVQVEMEAISSTK,TMWGFSQVLPIDTFK,SWNI	TRAF-like family protein	0,83349	1,3029	1,556	0,20275682	0,20835587	0,40775179
O81493	AT5G26260.1	5	SDYGVGVNNIILMSELK,YLSVTDGTVK,QLYSAAWYPIR,GEPYESSIFEAAGYK,GGINNHISLYAR,VTFISNPP	TRAF-like family protein	0,63298	1,4836	2,2147	0,03425878	0,12620998	0,0790075
Q9SGS4	AT1G76080.1	2	DMNVIEVPTFLFIR,SMSETVVFAR	thioredoxin-like protein CDSP32	0,55624	0,46211	0,83077	0,04306948	0,01107518	0,20167013
P92994	AT2G30490.1	1	NVVFDIFTGK	trans-cinnamate 4-monooxygenase	1,3467	2,0108	1,4931	0,13337707	0,0148253	0,08016342
Q9C5S7		3	NYINLAQIHASENSK,SFEQIEVER,IEEHLSR	transcription factor APFI	1,4992	2,829	2,0111	0,18621086	0,00341236	0,01797573
Q9SKZ1	AT2G32080.1	2	LFYFDLK,SSIILPLSGLK,AVTGGGGGGGGSDVELVSK	transcription factor Pur-alpha 1	1,552	1,1142	0,74811	0,09614373	0,26260914	0,13614308
Q8RWV0	AT3G60750.1	11	SIITGELPAGWEK,ALPTYTPESPGDATR,TVTDKPTLIK,LPHLPGTSIEGVEK,VSIEAASTFGWGK,SIGINSFGA	Transketolase	0,75957	0,73524	0,92749	0,21936801	0,09391219	0,25274925
Q38884	AT2G46280.2	2	LITGSADQTAK,IAEDPEEQDAESVLVLHCPDGK	translation initiation factor 3 subunit I	1,0777	1,4122	1,3453	0,26346102	0,13279871	0,08876987
P41377	AT1G54270.1	1	ALGDYQGVK	translation initiation factor 4A-2	1,3868	2,1011	1,515	0,11932159	0,012231	0,07647713
Q3E987	AT5G20920.2	2	SPDTILSK,ENNPELAGDR	translation initiation factor eIF-2 beta subunit	0,83238	0,76896	0,92381	0,21404153	0,12138972	0,33658501
Q9LD55	AT4G11420.1	3	DRPDLSAPESLQLYLDTR,TVLEILR,GVLSCASQEVK,SALLSELVSK,IYQTIR,DLQLIASSVVLAALSIPPFDR	translation initiation factor eIF-3 subunit 10	1,2676	1,6964	1,456	0,10345539	0,004953	0,03990795
Q9C5Z2	AT1G10840.1	2	NFSWMDIFEEIPIK,VVQIEGLAVLK	translation initiation factor eIF-3 subunit 3	0,65235	1,2309	1,8868	0,08023104	0,15790714	0,03184562
Q9C5Z3	AT3G57290.1	2	WIVNLIR,YQIGPDQIEALYQYAK	translation initiation factor eIF-3 subunit 6	1,0252	1,0528	1,0269	0,43915522	0,36742623	0,43784546
O49160	AT3G56150.2	6	YSNIVVDDTVEPDENETSKPTDYDGK,ELLAQGVSQSR,TLVMLEDFLNEALANK,VWGNLVAFLER,CVLNMI	translation initiation factor eIF-3 subunit 8	0,959	1,4085	1,5373	0,35339165	0,07645503	0,13180052
Q93VP3	AT1G26630.1	4	VDYQLIDITEDGFVSLLTDSGGTK,TYPQSAGNIR,CHFVAIDIFTAK	translation initiation factor eIF-5A	0,57058	1,2097	2,1202	0,04661118	0,17239492	0,02060502
P31265	AT3G16640.1	2	LSEEDQAVFK,GFIAYIK,VVDIVDTFR	translationally-controlled tumor protein-like protein	1,443	1,3843	1,0334	0,15606633	0,10082244	0,40431654
O81283	AT4G02510.1	2	SIRPEATNDK,SQGDSAYGANLEVR	translocase of chloroplast 159	1,2247	1,8834	1,3852	0,30681929	0,14149413	0,2685707
O23680	AT1G02280.2	1	LIPLIIGAQYLIVK	translocase of chloroplast 33	0,95996	0,6296	0,65586	0,40723125	0,04450111	0,07464304
Q38906	AT5G05000.3	1	QVVGAITDAFGK	translocase of chloroplast 34	0,77898	0,86994	1,1168	0,160455	0,22817556	0,28701334
Q9SHU7	AT2G15290.1	1	SVTVPNSESVVVPK	translocon at inner membrane of chloroplasts 21	1,262	2,0021	1,5864	0,172787	0,01516721	0,06399897
Q9SK50	AT2G24820.1	9	SPLVSVLPQWFWHQNACK,AVLLGSAALCSAAAYTCLR,VPPVVEHAPAGLIAALSASYPAK,NVPEDAPLGLT	translocon at the inner envelope membrane of chloroplasts 55-I	1,592	1,8361	1,0772	0,13134162	0,099675	0,49515082
Q94BY3	AT5G14030.4	2	LDAGGILSHSIELEAK,GVFYGAPAVVTFR	translocon-associated protein subunit beta	0,92431	1,1672	1,2234	0,42621916	0,15247118	0,15355836
Q940G0	AT1G10950.1	5	NWSGAPNNPCR,WGGLGEVLGGNELIDSEIAIK,SVICPLELDEAK,HVLYTHK,EDDDLESLER	transmembrane nine 1	1,1183	1,5071	1,282	0,1996738	0,03851025	0,12852101
Q94A32	AT3G43520.1	1	AVEVEPTIDYGGGGGIGGDK	transmembrane protein 14C	0,60987	0,61327	0,99523	0,06347689	0,05462372	0,37789765
Q93V66	AT3G57280.1	3	ESITEPVEEDVATQPIR,LKPSATSPSY,TSKPYSTVDETATNK	Transmembrane proteins 14C	0,90488	1,3203	1,4591	0,31147744	0,11216109	0,08876987
Q9ZVH7	AT2G38550.1	1	GPTLEDGGEDESSDGFVR	Transmembrane proteins 14C	0,5046	0,75619	1,4986	0,03199499	0,11129187	0,0791888
O23275		1	IVALVGGPCTEGPGTIISK	transport protein	0,6198	0,71351	1,1512	0,06347689	0,08288761	0,24929874
Q8H2D6		2	LLDFLEIASQQLSANLNR	transportin	0,88138	1,0178	1,137	0,22642083	0,20080233	0,48527501
Q8H0U4	AT2G16950.2	1	LLDFLEIASQQLSANLNR	transportin 1	0,67945	1,6689	2,4562	0,09614373	0,03426426	0,01225561
Q8L7S1	AT1G45201.1	13	SFILQFWK,IELDEK,SSATYTSFIGCSDR,TSSTNPDLIVVSFR,FVYNNDVVPR,INVGSIEYK,ENISLLHQYAYYTI	triacylglycerol lipase-like 1 protein	0,72468	1,0632	1,8314	0,18992425	0,11869583	0,05963097
Q8L4R0	AT1G19800.3	1	VLGADPIDYLITPR	trigalactosyldiacylglycerol 1	1,5554	1,902	1,2229	0,07507334	0,01909412	0,18571584
Q9LTR2	AT3G20320.1	2	NVESISSDILGFTGDEATR,SIYTLVYTLK	trigalactosyldiacylglycerol2	1,2206	1,8098	1,4827	0,19994416	0,02357655	0,08303118
Q8S9L5	AT5G55220.1	2	VTETVQANSSVK,LNEDQLASLSSQK,LLPGFLDAIIGIR	trigger factor type chaperone family protein	1,0287	0,77232	0,7757	0,41601691	0,13179251	0,17836987
P48491	AT3G55440.1	4	AILNESSEFVGDK,NVSADVAATTR,IIYGGSVNGGNCK,SDFFVAAQNCWVK,VIACVGETLEER,EAGSTMDV	triosephosphate isomerase	1,3613	1,3322	1,0044	0,07253728	0,03089351	0,33025215
Q9SKP6	AT2G21170.1	2	AFADAVPSWDNIVVAYEPVWAIGTGK,TFDVCFAQLK	triosephosphate isomerase	1,3456	1,7793	1,3223	0,13337707	0,02539608	0,13375719
Q9SHJ6	AT1G06530.1	2	VVALESEIVELQK,TEGEEATAEAEK	tropomyosin-related protein	1,2387	1,5917	1,2521	0,47196645	0,19996677	0,236846
O80852	AT2G30860.1	2	QPAYLALQPFGTVPAVVDGDYK,LAGVLDVYEAHLSK	glutathione S-transferase	1,7945	2,1235	1,1833	0,04306948	0,01183842	0,21925365
P20363	AT5G19780.1	4	SLDIERPTYTNLNR,LISQIISSLTTSLR	tubulin alpha-3/alpha-5 chain	0,80571	0,69757	0,89015	0,11207126	0,05241017	0,33321787
P12411	AT1G75780.1	1	GHYTEGAELIDAVLDVVR	tubulin beta	1,3876	0,78015	0,56222	0,11932159	0,12933635	0,03896583
Q9SAF5	AT1G13210.1	1	LPLSITQLLLR	phospholipid-translocating ATPase	1,7937	1,8473	1,0299	0,04306948	0,02156717	0,43108996
P24636	AT5G44340.1	1	IDVYFNEASGGK	tubulin beta-4 chain	0,83351	1,1512	1,223	0,08439007	0,05462372	0,48109254
Q8L7S8	AT5G26742.2	4	TILSDLITVYAK,SVTGFLSDLYR,GWVTLQLIR,IFLIADDR	DEAD-box ATP-dependent RNA helicase 3	0,55758	0,53529	0,96003	0,04306948	0,02063113	0,40775179
Q9FLT0	AT5G61780.1	6	INQISAVVEYVLSGHR,TNAGTYLLEAGLAK,VSPYIAELEQLEEQAQQEGFGR,FYVQTVGDQK,DAGEYLHTV	TUDOR-SN protein 2	0,91823	1,369	2,0316	0,04306948	0,02566376	0,00380368
B9DFD5	AT4G03560.1	4	EEFGLIFDELDDTR,YLLLAR,YYFIFTR,AAALVDLAEDGIGLPVEILDQSSFGESAR	two pore calcium channel protein 1	0,91243	1,0899	1,1246	0,09332549	0,04062558	0,32323666
P43333	AT1G09760.1	4	LVNLVEIDPLASIPK,LENFPYLNR,VVAPTAEQILAIK,LTADLIWK	U2 small nuclear ribonucleoprotein A'	0,91313	1,1406	1,2491	0,3231144	0,23940646	0,16725321
Q39192	AT3G50500.1	1	IFVADPATR	serine/threonine-protein kinase SRK2D	0,55803	0,38964	0,69825	0,04306948	0,00489045	0,09745137
Q9LK29	AT3G27240.1	4	MLNDEAVEYEDGVPATEAQMGK,FPQPYANESAAR,HNGPNYVFALLTGYR,DIVSFLAWAAEPEMEER	ubiquinol-cytochrome c reductase cytochrome c1 subunit	0,92697	1,2151	1,3108	0,34725117	0,16863695	0,13736454
Q9FKS5	AT5G40810.1	1	DVVSFLSWAAEPEMEER	ubiquinol-cytochrome c reductase cytochrome c1 subunit	0,65924	1,1054	1,6768	0,08330194	0,28150925	0,04995052
Q9LYR2	AT5G13440.1	1	LANSVDVGSLR	Ubiquinol-cytochrome C reductase iron-sulfur subunit	0,78842	1,0134	1,2854	0,16916149	0,46065137	0,14943771
Q9SUU5	AT4G32470.1	2	GYLQDMLALVER	ubiquinol-cytochrome c reductase subunit 7	0,87336	0,95979	1,099	0,26317249	0,38889733	0,31160325
Q9LXJ2	AT3G52730.1	1	YEDISVLGQRPVEE	ubiquinol-cytochrome c reductase subunit 9	1,3155	1,4588	1,109	0,14481507	0,06838838	0,29614912

Q9LYR3		3	GPAPYNLEVPTYSFLEENK,AFAYFVLSGGR,LANSVDVGSLR	ubiquinol--cytochrome-c reductase-like protein	1,1695	1,7187	1,4696	0,2365211	0,02949463	0,08548837
P59233	AT3G62250.1	1	LAVLQFYK	ubiquitin-40S ribosomal protein S27a-3	0,83342	1,867	2,2402	0,21554938	0,02063113	0,01740826
Q9FZ48	AT1G16890.2	2	SNEAEAVETAK,ICLDILK	ubiquitin-conjugating enzyme E2 36	0,57982	1,6608	3,0504	0,08067319	0,13262018	0,02836906
Q9LM76	AT1G20780.1	1	VVEAALAALSSLLEDGLDVEK	U-box domain-containing protein 44	1,0482	0,69473	0,6628	0,39433221	0,0731004	0,07799316
O80517	AT2G44790.1	6	YGSSHTVDVVDK,TVGINYFICSTPGHCR,AGYDGCDASSSTENHSDGDTK	uclacyanin 2	1,3435	1,4259	1,1264	0,11932159	0,08279093	0,47008771
Q96316	AT3G60280.1	1	VGDTLEFVYGLSHSVSVVDK	uclacyanin 3	0,81921	0,61328	0,74144	0,18329251	0,00802105	0,03363603
O49607	AT4G34980.1	1	DSGCLDSDILAAFDAAVR	subtilisin-like serine protease 2	1,7715	1,8068	1,0199	0,04550156	0,02365712	0,45060381
Q9C5H6	AT3G59360.2	1	NNVLLAVPALLYAINNYLK	UDP-galactose transporter 6	1,1837	6,6254	5,5974	0,22481583	4,52E-05	0,00012856
O23649		2	DFVYLLDNVPHDWLFPR,MTEIIVEALQR	UDP-glucose:sterol glucosyltransferase	2,4789	1,5141	0,61079	0,0126573	0,05630528	0,05583941
Q9SX77	AT1G47420.1	4	VTVLGTSGLSGSYVEQR,AAEAVEEFGGILTSIK,DLMAADWAELPSAVVK	succinate dehydrogenase 5	1,7168	1,9105	1,1213	0,04572858	0,01758657	0,31472934
Q9LFX8	AT1G27090.1	2	SKPAVVILIDELEK,APLSAAVTEEISLATQLNR	uncharacterized glycine-rich protein	0,54175	1,2297	2,27	0,0390965	0,15817724	0,01658271
Q94EG9	AT1G55910.1	1	TQNDLELQGEDK	zinc transporter 11	1,7651	1,7328	0,98174	0,04575845	0,02844818	0,45342888
A8MR14	AT1G64650.2	1	NAASAIASDEK	general substrate transporter-like protein	0,56728	0,47564	0,83847	0,04581505	0,012231	0,21118782
Q94A28	AT4G26970.1	1	EGVTATDLVLTVTQILR	aconitase 2	1,7543	2,4212	1,3801	0,04661118	0,00639157	0,11443023
Q9M7Z1	AT3G06850.2	1	FGSPLLNLPEVAIIALGR	2-oxoisovalerate dehydrogenase E2 component (dihydrolipoyl t	1,7488	2,0085	1,1485	0,04672786	0,01489476	0,25160888
Q9C5M3	AT1G78970.2	2	EFVECTSSVIQALDLFR,FVGPITPLILLLR	lupeol synthase 1	0,57192	0,45729	0,79957	0,04672786	0,01050728	0,16664956
Q9ZV49	AT2G18690.1	1	FSFLNVVK,DVVAILNESR	uncharacterized protein	1,815	1,6017	0,96295	0,12245672	0,05048437	0,27742113
Q9M1X3	AT3G63160.1	1	SGGEVNFPK	uncharacterized protein	1,7931	2,3293	1,2423	0,08067319	0,04514995	0,41917244
O49636	AT4G22310.1	1	WGISIANIADFAKPPEK	uncharacterized protein	1,7871	3,4205	1,914	0,04337302	0,00142593	0,03075562
Q8H0X5	AT5G16660.1	1	SVLNEEDEYGFEKPK	uncharacterized protein	1,7207	2,9726	1,7276	0,04823352	0,00269651	0,04475628
Q9LPD7	AT1G44920.1	2	VALLAGGDVLALLIFSAIGR,SWIVGTPLGIIIR	uncharacterized protein	1,7008	1,6044	0,9433	0,05045175	0,04253299	0,37223071
Q9C803	AT1G33490.1	1	ISEPLIQILR	uncharacterized protein	1,6846	3,0753	1,8255	0,0523846	0,00243954	0,0355902
Q8LDV3	AT4G13200.1	3	IESTVGEVLSTIGK,SVLDAFFLGK,ALAEVINER	uncharacterized protein	1,604	2,1065	1,3133	0,06440019	0,012231	0,13614308
Q9LHA3	AT3G28720.1	1	IPVYFEDTLVVQFIHVYGSEVK	uncharacterized protein	1,5688	0,49321	0,31439	0,07214592	0,01434903	0,00380368
Q8L9M8	AT1G33810.1	1	IFTQFFDPEGIANAQK	uncharacterized protein	1,5449	1,5535	1,0056	0,07711156	0,04941378	0,48558152
Q8S8F1	AT2G36885.1	2	YSDDIEEK,SVFGPVVEIVK	uncharacterized protein	1,5177	3,7543	2,5443	0,13062538	0,00134791	0,01050095
Q941A6	AT1G08480.1	3	LSFFENYTR,FMEWWER,EAVTFGVTGAALGAVSTAAFAWK	uncharacterized protein	1,509	1,5431	0,974	0,08154038	0,14986913	0,18261312
Q949R9	AT5G20090.2	2	FAWMVQPR,FQAFLNSPIGPK	uncharacterized protein	1,4732	1,9397	1,3025	0,09614373	0,14624026	0,22275931
Q8LDD3	AT4G38100.1	1	DGVVVAAEEK	uncharacterized protein	1,4665	1,4593	0,99511	0,09785025	0,06833032	0,48736986
Q9LTX0	AT3G12650.1	1	DLSALGNETR	uncharacterized protein	1,3926	1,5815	1,1357	0,11867247	0,04525952	0,26397186
Q8VZM7	AT5G02940.1	1	ESILLLGWR	uncharacterized protein	1,3452	1,2831	0,95383	0,13337707	0,12897147	0,39385206
Q8RWI0	AT1G54520.1	1	LAESSDTSTPEGLSYVLTEATLALLR	uncharacterized protein	1,3332	1,6657	1,2493	0,13835208	0,03454204	0,16725321
Q9ZPZ4	AT1G09310.1	1	LVSYGTEVTAIVETGK	uncharacterized protein	1,2887	0,99265	0,79954	0,23911415	0,46478545	0,24654974
Q9FKA5	AT5G39570.1	2	SEEQEEGSYR,SEEQEEGYR	uncharacterized protein	1,2857	0,61357	0,50047	0,46625775	0,02357655	0,03801615
Q8L9R6	AT1G68680.1	2	FLVPLIYAPALPLIR	uncharacterized protein	1,2571	1,6862	1,3413	0,17669977	0,03240953	0,12730146
Q9C7F9	AT1G27930.1	1	LAPANFLVFGLGR	uncharacterized protein	1,2249	1,582	1,2916	0,19663579	0,04523822	0,14562255
Q8S8R9	AT2G34585.1	1	SLNPDYDPDDQDVDSSSDVR	uncharacterized protein	1,1941	1,5337	1,3931	0,31684455	0,05301425	0,10920229
Q9SN96	AT5G59613.2	2	LFDPWPVFFK,LTAGLTEEDAK	uncharacterized protein	1,1755	2,6927	2,313	0,17134952	0,00367439	0,02006469
Q94C78	AT3G51140.1	1	VVNFVFER	uncharacterized protein	1,1441	1,749	1,5287	0,26346102	0,02701153	0,07421815
Q9S791	AT1G70770.2	5	IDPLNLEAFLVEASESYASQPEIQLMR,ALSGVSSVQFPWVK,DLILQLVEK,EVALAGAPGSK,LAGEGNPVLAK	uncharacterized protein	1,1418	1,8446	1,6231	0,19251025	0,01894149	0,0700569
O82251	AT2G47840,1	4	VISIASYALPFFNSLQYGR,LGLLFEPIFPILNLYR,FLFAQYPR	uncharacterized protein	1,1353	2,7703	2,4402	0,274315	0	0
Q9SZV4	AT4G30010.1	1	VTELPGYIK	uncharacterized protein	1,1342	2,4587	2,1679	0,27549839	0,00614667	0,01957823
Q9M898	AT3G02420.1	2	FALIPILCR,YAPFLNTPVSAVQR	uncharacterized protein	1,0997	1,8656	1,6964	0,31679829	0,02063113	0,04763299
Q9FM77	AT5G55610.1	1	TASEDDDDFLNNAAEENR	uncharacterized protein	1,067	1,4339	1,3439	0,36464091	0,07432637	0,1272131
Q9C9Z2	AT3G08640.1	2	YQTLNGIEFLLAK,VAADPQFPFK	uncharacterized protein	1,0628	1,9728	1,8562	0,37157152	0,01649625	0,03345105
Q9FF91	AT5G23890.1	1	GAAADVEQGTVQVSDK	uncharacterized protein	1,0547	2,1331	2,0226	0,38437408	0,01174781	0,02490532
Q8VYY8	AT2G43630.1	1	AIINTPTWYDSPEK	uncharacterized protein	1,0538	1,3696	1,2997	0,3847187	0,09314922	0,14359696
Q38842	AT4G00860.1	1	LFGGTTPGTITNK	uncharacterized protein	1,0394	2,1236	2,0431	0,4109947	0,01183842	0,02416558
Q9SU27	AT4G12590.1	2	GLFSLILGDENAIDDTQR,DGLDIIQHEWALPR	uncharacterized protein	1,0277	1,5358	1,5927	0,46382572	0,07226174	0,09959821
Q9M292	AT3G44330.1	5	IFADDSSLAVNPFYVR,LFSVLYSNPK,GLDDLISLFR,IASPTITNIQGWLPGLR,NDALGQQATATTGGYK,QLV	uncharacterized protein	1,015	1,254	1,2173	0,31077465	0,16441248	0,30423363
Q8RWC3	AT3G19340.1	2	LGIVAR,LTIQEPTFDR	uncharacterized protein	0,99169	0,80457	0,81176	0,47999997	0,14940749	0,17723399
Q8L604	AT1G65230.1	1	SILAAVSGDLIVK	uncharacterized protein	0,98463	0,49427	0,50199	0,46022159	0,01452618	0,02638708
Q9FG71	AT5G55940.1	2	EPSANVVLSDYISSEK,YFPQAPILLLNNK	uncharacterized protein	0,93314	1,3281	1,4059	0,3847187	0,10576311	0,10404583
Q9LYG2	AT5G11680.1	1	EGGCGTFVPLFLNLISSVR	uncharacterized protein	0,89743	1,0726	1,1952	0,29970228	0,3296146	0,20888524
Q93Z11	AT5G37360.1	4	FGELQVAVTPEK,LPELEILLTDGAR,AWEQADTSTSR,LDLNDAAFDLSNGPVIDECGQELGETLLNLSR	uncharacterized protein	0,86662	0,70713	0,81596	0,2523643	0,0791647	0,1833606
Q0WRM9	AT1G09330.1	2	FESLDQESLAR,WWNEINDLGESVWK	uncharacterized protein	0,86314	0,92791	1,075	0,24687717	0,32290855	0,34841671
Q94F20	AT5G25460.1	1	GSMVVEAFAGK	uncharacterized protein	0,8549	0,36093	0,4222	0,23646531	0,00335257	0,01398742
Q9SFB1	AT3G08030.1	2	VPHTSVGGGHVK	uncharacterized protein	0,7933	1,1422	1,4771	0,10664388	0,19358982	0,04731124
Q8GXB1	AT2G17695.3	1	EVLPWVMLPLQVVYVDESR	uncharacterized protein	0,76193	1,1275	1,4798	0,14602554	0,25408752	0,08318031
Q8GXK1	AT4G33625.1	2	TEEIEESPAGPSSGSAK,ADPFLVVCR	uncharacterized protein	0,75856	0,5843	0,77028	0,14408762	0,03036783	0,14369822
Q9M2Y6	AT3G49720.2	2	VTGDYSCTAEVQR,SFSLVIVSDALDYLSPK	uncharacterized protein	0,74224	1,1888	1,6016	0,13337707	0,18932684	0,06076306
Q8LAR0	AT4G32460.2	1	NDDFSSLCGPVIDDVK	uncharacterized protein	0,74175	0,55733	0,75137	0,13337707	0,02440769	0,13010259
Q8GSG8	AT5G01010.1	1	YLVGVPQVLLDFIR	uncharacterized protein	0,74085	1,0188	1,3752	0,13264411	0,44591935	0,11577586
O80503	AT2G44640.1	4	SPISADVFGSACYTFQK,IVAWYSPK,EGNTEEEDKPVFLPYDLR,LNLIFQQQVAGPIVFK	uncharacterized protein	0,73167	0,84582	1,156	0,1268856	0,19494297	0,24368858
Q9FF88	AT5G23920.1	1	IFALLDWVK	uncharacterized protein	0,71812	0,89783	1,1022	0,10942681	0,0428636	0,27837031
Q93XX1	AT5G55960.1	2	TSLSTVDSQAR,VFSSSASVVGGGAK	uncharacterized protein	0,63592	0,81003	1,2738	0,07142542	0,15530305	0,1545229
Q84W12	AT4G16450.2	1	VVGNFSTLDYLR	uncharacterized protein	0,58881	0,46477	0,78934	0,05070211	0,01126596	0,15741367
Q8W553	AT5G35180.1	1	TIPYALLLQVAGLK	uncharacterized protein	0,57569	0,68273	1,1859	0,04754354	0,06741156	0,21703109
Q9XI93	AT1G13930.3	1	VVAEAAQAAAR	uncharacterized protein	0,53415	0,47264	1,018	0,04700634	0,01758657	0,29405411
Q9LFY3	AT1G27030.1	1	LVIAEVLVDELSPESQGIIR	uncharacterized protein	0,52108	0,79569	1,527	0,03514917	0,14149413	0,07439308
Q7Y1W1	AT5G01590.1	1	QFLFGLWGFR	uncharacterized protein	0,44757	1,83	4,0889	0,01908925	0,02244204	0,0008235
Q9ZU97	AT2G01690.1	1	IEALNWISTLLNK	uncharacterized protein	0,36285	1,0444	2,8784	0,00736139	0,3845941	0,00626206
Q9LYE7	AT5G11420.1	2	AIPSWELSGFVEYIK,ESAIAQVAR	uncharacterized protein	0,36003	0,2067	0,57718	0,00630893	0,00025441	0,04995052
Q38845	AT1G25490.1	3	DSTIEHLLPIFLSLLK,ILPVIVNFSQDK,LAIIEYVPLLASQLGIGFFDDK,ENDLVESFVPLVK	serine/threonine-protein phosphatase 2A regulatory subunit A a	1,1601	0,79348	0,95857	0,04741018	0,02515116	0,44200576
O81845	AT3G54110.1	8	DFVGDVPLSK,NAIINAAELASYDQVK,ALWTGLGPNVAR,ILAGLTTGALGIMVANPTDLVK,IPGFTDNVVTH	uncoupling mitochondrial protein 1	1,5568	1,7493	1,1227	0,06252003	0,02151949	0,26059071
Q9M328	AT3G53990.1	2	IIMGSVSSFVIQHAPCPVTVVK,SGSPLIPLAEFR	universal stress protein (USP) family protein	2,0188	1,082	0,53595	0,02909831	0,31597334	0,03307014
Q9M0X9	AT4G05160.1	2	GFDLPVVLLGSK,IIISVNQLFDK	4-coumarate--CoA ligase-like 7	1,7386	1,8537	1,0662	0,04741018	0,02119669	0,36371306
Q8LG09		1	ILLSNFGILDLE	unknown	1,5146	1,5214	1,0044	0,08361268	0,05501971	0,48836072
Q8LFF4		1	ELPLALNAFEEVLLFDPNNK	unknown	1,2053	1,3999	1,1368	0,2218635	0,14538159	0,38489721
Q8LBF2		2	WAVLYSASLLK,ISNEQQQNLTR	unknown	1,1262	1,573	1,3967	0,28627108	0,04644231	0,10811884
Q8LEY8		2	TVFLFEFPFYSR,GVYGNLPGWFK,TTSPLILFPR	unknown	0,89854	1,1793	1,3285	0,35682557	0,17644154	0,14531065
Q8LD22		3	IPVIGPLLIPPVNVK,AAVDPEFASGVIDVYR,FVYISAADFGVINNLIR	unknown	0,79385	1,1731	1,4778	0,17452586	0,20300003	0,08325311
Q8LFG2		1	FLSQFIPVFYIR	unknown	0,75022	0,89406	1,1917	0,13836381	0,26555968	0,21194888
Q8LAS5		5	WLPLFYVPSLVVLPLSVR,TSLPFLLSSTVLGYIVGSGLPSSIK,LVGLEPSLTVSILPR	unknown	0,69981	1,9422	2,0097	0,17831287	0,04934788	0,02844234

Q941L3	AT5G61900.3	1	LDEQQFLGEATCALSEIITK	calcium-dependent phospholipid-binding copine-like protein	1,7347	2,3296	1,3429	0,04768595	0,00774231	0,12730146
Q8LEX2		1	DCCAGFGQVIR	unknown	0,50342	1,099	2,183	0,03198338	0,29070864	0,01919518
O22126	AT2G45470.1	2	TFANLLVSSGVLK,FDLTTSTSGDEVILHTGVAPSR,TYESAVEK,LTQAEVVSLLEYHALAEYKPK	FASCICLIN-like arabinogalactan protein 8	0,87579	0,53196	0,6925	0,04803632	0,01107518	0,16725321
Q9FZ47	AT1G16880.1	2	ALIKPLQQVLANSLR	uridylyltransferase-related protein	0,5611	0,43187	0,74684	0,04823352	0,00802364	0,12852101
Q93VA8	AT1G76010.1	1	TVTIVELIK	Alba DNA/RNA-binding protein	1,7247	1,6361	0,94866	0,04823352	0,03811548	0,38201671
Q8GXZ8		5	LISNLSTNSVSSTS,AADSTSSSPSVASGDR,FTSFFGPNITAEVGK,ESCATPILTQVR,SAPILQLIR	unknown protein	1,5334	2,0004	1,3046	0,08023104	0,01518455	0,14080386
Q941K9		6	DLIAAGATAANQLR,FQNFIR,AGGIPDIIPEDQEGK,GVDSESFNPR,AADLTLVPSAAIGK,VPLSLALSPR	unknown protein	1,5264	1,687	1,1032	0,06266339	0,01909412	0,22896149
Q67ZP4		1	TLGLVLLR	unknown protein	1,2786	1,2501	0,97908	0,16254974	0,14419946	0,4460835
O80821	AT2G41470.1	1	DVCYLYLLR	unknown protein	1,1128	0,65935	0,5925	0,30160883	0,05574351	0,04854766
Q9SSF3		3	QSLLYAVGPAGFFPSSPDFR,FQLTATLPR,FVTTTTINYNTGSQSVVK	unknown protein	1,0721	1,0504	0,97981	0,35896271	0,37274531	0,4497903
Q8L7P2		2	NTALSEVR,LLGLHK	unknown protein	1,0214	1,3262	1,2984	0,44797159	0,10986489	0,14369822
Q67Y15		1	DVCYLYLLR	unknown protein	0,88578	0,78367	0,88472	0,28320985	0,1318416	0,27044118
Q9M5K4	AT4G16155.1	6	VDSPASVTAQSVK,ALGVDILTGFGAVLGPQK,LAVHAHPTLSEVVDELFK,ALAENEGEGLAK,QGVADHASN	unknown protein	0,78714	0,75147	0,99008	0,24243794	0,14939195	0,35736084
Q8RWN9	AT3G13930.1	6	VEKPASAPEAK,IFASPLAR,ISVNDLVIK,ADVEDFLASGSK,VPALDYVDIPHTQIR,VIDGAIGAEWLK	dihydrolipoyllysine-residue acetyltransferase component 2 of py	1,7859	2,337	1,3526	0,04823352	0,00335257	0,05963097
Q9FHN0		1	YVYPVFTPSVVLFVAGTTAYGVLK	unnamed protein product	1,8278	0,64874	0,35493	0,04044006	0,05154991	0,00661528
Q9FIR6		1	AAELSILGLAAGTLQGSLSNVLAGK	unnamed protein product	1,2843	2,0214	1,5739	0,16029952	0,01456931	0,0659409
Q9FH68		2	LIILTFDR,SEDGAVLNPTDQVLASK	unnamed protein product	0,86833	0,92258	1,0549	0,26113057	0,28356771	0,41453009
Q9FNL5		1	SFILNQLLGR	unnamed protein product	0,7684	0,86825	1,1299	0,15102512	0,22601448	0,27044118
Q9LU53		1	ALALLEILQFR	unnamed protein product	0,59199	0,9032	1,5257	0,05198642	0,27994562	0,07461162
Q1EBW2	AT5G19550.1	3	VFNLAGLSVEYFR,EYIPIVGISDFNK,VTTVQCLSGTGSLR	aspartate aminotransferase	1,7205	2,192	1,2741	0,04823352	0,01046927	0,1545229
Q9SG80	AT3G10740.1	1	DAGTGSLLASLAEAAFLIGLEK	alpha-L-arabinofuranosidase 1	0,58384	0,56406	0,96613	0,0492606	0,0257728	0,42018922
O49048	AT1G77140.1	1	VVDGIAAVFLALK	vacuolar protein sorting-associated protein 45-like protein	0,59528	1,1887	1,9969	0,05277604	0,18932684	0,02638708
P93026	AT3G52850.1	1	LPTFVLIDR	vacuolar-sorting receptor 1	0,91838	1,234	1,3186	0,33667925	0,21001488	0,1580139
Q9SH90		4	LVTMQFLLPSMYK,FSGIASESVVSEK,DGGFQLLAK,LSSHLEK	unknown protein	1,7123	1,8655	1,0894	0,0492606	0,02063113	0,32531804
Q9ZRD6	AT5G58060.1	1	SAFSLLNQVLDEYQK	VAMP-like protein YKT61	0,55452	0,67777	1,2223	0,04293598	0,06404894	0,18622741
Q8LC74		1	SATVENLIEAGYHSWSNLLLR	vegetative storage protein-like	0,82062	0,79065	0,96347	0,20119711	0,13666172	0,4147151
Q8LAZ1		1	ANITTIGPLVLPASEQLLFLTSLIGR	very-long-chain fatty acid condensing enzyme CUT1	0,85721	1,4413	1,6813	0,23930232	0,07262151	0,04946517
Q9M003	AT3G60600.1	2	QISCSLYLTNK,FLLQGVIASPGVTAK	vesicle associated protein	0,52567	0,75289	1,4322	0,03609131	0,10904609	0,09745137
Q9ZTW3	AT1G04750.1	1	GNFTSIAAQCLQK	vesicle-associated membrane protein 721	1,0121	0,9647	0,95319	0,46816001	0,40150829	0,39247935
Q9M0Y8	AT4G04910.1	4	DGNIALNAIQR,LLEFIAIGPR,ITMEDFLHAIYEVQPAFGASTDDLER,FSNIISQTLMVLLK	vesicle-fusing ATPase	0,97167	1,5238	1,6512	0,2547768	0,02644344	0,02474635
Q9XI87	AT1G14000.1	1	GGLTPTTAVNFALDIAR	VH1-interacting kinase	0,80043	0,93885	1,1729	0,18059715	0,34358374	0,22914484
O80910	AT2G38410.1	1	VQLLALTLLETLVK	VHS and GAT domain-containing protein	0,85774	1,1717	1,356	0,23258573	0,32795148	0,15909481
Q9FJX3	AT5G67500.1	12	FSISTYSASGVALTSTALK,FSTNENTITVGGLYAIDHSTAVK,IDTDSSVLTTVTLTEILPSTK,YNAGISVTKPDACI	voltage dependent anion channel 2	1,4076	1,7962	1,2455	0,09040669	0,02050831	0,16366213
Q9FKM2	AT5G57490.1	3	LGLALALKP,GQNTIVDLK,DDFFFGDISTLYK	voltage dependent anion channel 4	1,0953	1,684	1,5375	0,3231144	0,03258199	0,07263455
P59227	AT4G38920.1	1	SGVGVASMGVMRPELVMK	V-type H+-transporting ATPase 16kDa proteolipid subunit	1,1932	1,1031	0,92448	0,21902323	0,28455228	0,33799488
Q9SJT7	AT2G21410.1	4	CLVGEGWSPVFAATEIQDALHR,FTTAFQEIVDAYGVAK,QSVIEESVVDPNSGEK	V-type H+-transporting ATPase subunit I	1,0196	0,82589	0,81004	0,45267591	0,17190195	0,17667834
Q8W4S4	AT4G39080.1	11	LGELEAELVEINANNDK,QAQMITEVSGR,FTSAIQEIVDAYGVAK,NILLQTIGDK,FAPFTFIFTANEDE,TTIDA	V-type H+-transporting ATPase subunit I	0,84745	0,82696	0,9862	0,36417609	0,44488957	0,32764524
O23654	AT1G78900.2	26	YSNSDAVVYVGCGER,EASIYTGITIAEYFR,EDDLNEIVQLVGK,ITYIAPAGQYSLK,LTTFEDDEK,NIIHFYNLAI	V-type proton ATPase catalytic subunit A	1,0097	0,93121	0,91031	0,29581586	0,32979842	0,40734824
P11574	AT1G76030.1	2	FTTVQFTGEVLK,TVSGVAGPLVILDK	V-type proton ATPase subunit B1	1,0506	1,2319	1,1726	0,39110214	0,1572751	0,2293484
Q9SZN1	AT4G38510.4	2	TVSGVAGPLVILEK,FVAQGAYDTR	V-type proton ATPase subunit B2	1,1645	1,4264	1,2187	0,2166652	0,03794099	0,11842417
Q3ED91	AT1G20260.1	3	FVMQGAYDTR,FTTVQFTGEVLK,TVSGVAGPLVILDK	V-type proton ATPase subunit B3	1,3401	0,92781	0,70662	0,18917409	0,32702643	0,13180052
Q9SDS7	AT1G12840.1	10	DSASSLWNR,LVQDQESLR,EVVDNIQSQVAK,VGTLDSLLALGDDLLK,DFEQSVEAQETR,LCDSTNSLYWK,	V-type proton ATPase subunit C	0,95073	1,1244	1,2006	0,41555899	0,10904609	0,15236678
Q0WWD3	AT3G58730.1	5	LENTISYIK,NDLTGLAR,TENIAGVK,GELDELER,VNALENVVKPK	V-type proton ATPase subunit D	1,251	1,2664	1,0123	0,17906573	0,13614272	0,46848158
Q9LJI5	AT3G28710.1	5	AYLEDFYK,LYSNFGLLYPYGHEELAICEDIDQVR,AGLLTTADYNNLCQCENLDDIK,AVNITINSIGTELTR,LGG	V-type proton ATPase subunit d1	1,042	1,0308	0,9892	0,40683029	0,4144451	0,47209125
Q9LHA4	AT3G28715.1	1	AYLEDFYNFCQK	V-type proton ATPase subunit d2	1,2935	1,1837	0,91511	0,15509285	0,19358982	0,32052402
Q39258	AT4G11150.1	4	ANEISVSAEEEFNIEK,VLQAQDDIVNAMK,DLLNVSR,SNDPHGLHCSGGVVLASR,LDVAFR,IVCENTLDAR	V-type proton ATPase subunit E1	1,294	1,4487	1,1181	0,15554632	0,0553681	0,22684353
Q9ZQX4	AT4G02620.1	4	FLVDSYNKPVPAILEIPSK,DDIAIILLSQYIANMIR,YLFSAESVSQR	V-type proton ATPase subunit F	0,94243	1,5267	1,6199	0,37451204	0,05405697	0,05825772
O82628	AT3G01390.2	2	DVVEMLLK,AQTEQDFQR	V-type proton ATPase subunit G1	1,6419	1,9263	1,1732	0,0599686	0,01800598	0,22896149
Q9SU63	AT3G48000.1	6	AGTVWVNCFDVFDAAIPFGGYK,VILGLAANSNLKPVTLELGGK,AVVTALNKPAWI,SPFIVFEDADIDK,TGE\	aldehyde dehydrogenase 2B4	1,7102	2,0569	1,2027	0,04954348	0,01354858	0,20220305
Q9SJL9	AT2G36870.1	1	EIIFLVDDIPIR	xyloglucan:xyloglucosyl transferase	1,5321	0,78485	0,51226	0,08032741	0,13246774	0,02815539
Q8LER3	AT4G37800.1	7	DFDIEGCPVPGPADCPANSK,APFYAYYK,DFHEYAISWNHLR,LDPSSGCGFASK,VNLWFDPSR,FQPMGVY	xyloglucan:xyloglucosyl transferase	0,73775	0,79534	1,0946	0,14557629	0,12620998	0,45253267
Q8LFA4		1	GTGGDPFGAATK	xylose isomerase	0,79602	0,50087	0,62921	0,17677818	0,01535117	0,06355894
P56786	ATCG01280.1	1	IFSILLLR	Ycf2	1,2059	2,078	1,7232	0,21184587	0,01280421	0,04504245
A8Y7S8		1	EADVDGDGQINYEEFVK	Z-box binding factor 3	1,3156	1,0505	0,79671	0,14408762	0,45887369	0,12852101
Q9FGC7	AT5G67030.1	1	YEGDLLVGADGIWSK	zeaxanthin epoxidase (ZEP) (ABA1)	1,0439	0,83768	0,80247	0,40306264	0,18496683	0,16978186
Q9SIL0		1	YGPFLADLQGCDFVR	Unknown protein	0,5859	0,58113	0,99186	0,04967535	0,02942552	0,48024433
Q9LW57	AT3G23400.1	7	LPDSFRPSSNPGTGDFEVTYVDDTMR,DFDNIAEVELGAPWPFPPLEATATLAHK,FELLGTCK,GLVASVDDLI	putative plastid-lipid-associated protein 6	1,4798	1,6173	1,0824	0,04967535	0,03674982	0,4195237
